# Effects of ligands on the migratory insertion of alkenes into rhodium–oxygen bonds†‡

**DOI:** 10.1039/d0sc04402d

**Published:** 2020-09-08

**Authors:** Casseday P. Richers, Sven Roediger, Victor Laserna, John F. Hartwig

**Affiliations:** Department of Chemistry, University of California, United States and Chemical Sciences Division, Lawrence Berkeley National Laboratory 1 Cyclotron Road Berkeley CA 94720 USA jhartwig@berkeley.edu; Department of Chemistry, University of Illinois, Urbana-Champaign 600 South Mathews Urbana IL 61801 USA

## Abstract

Migratory insertions of olefins into metal–oxygen bonds are elementary steps of important catalytic processes, but well characterised complexes that undergo this reaction are rare, and little information on the effects of ancillary ligands on such reactions has been gained. We report a series of alkoxo alkene complexes of rhodium(i) that contain a range of bidentate ligands and that undergo insertion of the alkene. Our results show that complexes containing less electron-donating ancillary ligands react faster than their counterparts containing more electron-donating ancillary ligands, and that complexes possessing ligands with larger bite angles react faster than those with smaller bite angles. External added ligands had several effects on the reactions, including an inhibition of olefin isomerisation in the product and acceleration of the displacement of the product from complexes of ancillary ligands with small bite angles. Complementary computational studies help elucidate the details of these insertion processes.

## Abstract 2

A series of diphosphine-ligated rhodium(i) alkoxo alkene complexes is reported and the migratory insertion of the alkene moiety into the rhodium–oxygen bond in these complexes was studied, revealing the effects of the ligand on the insertion process.

## Graphical abstract

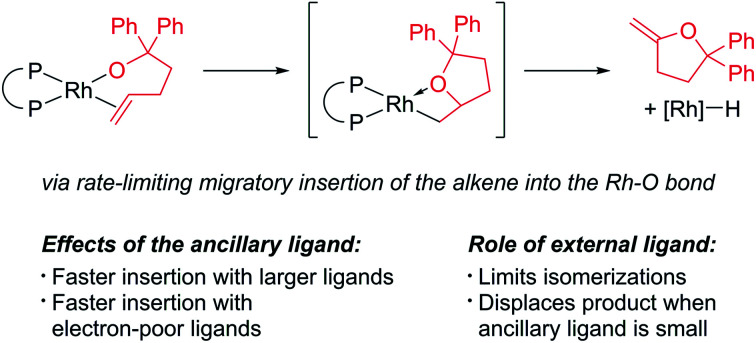

## Introduction

Migratory insertions are a fundamental process of organometallic chemistry that occur in a wide range of commonly practiced catalytic reactions.^1^ The most common classes of olefin insertions involve reactions of metal-hydride, -alkyl, or -aryl complexes, and these reactions are part of many catalytic processes, including hydrogenation,^2^ olefin polymerisation,^3–6^ and arene alkylation^7^ and vinylation.^8,9^ The migratory insertion of alkenes into metal–heteroatom bonds is much less common than insertions into metal–hydrogen and metal–carbon bonds,^1^ and information on the influence of the properties of the metal–ligand fragments on the rates of these reactions is limited. The pathways by which C–N bonds with alkenes form are emerging, and studies have begun to reveal the factors that dictate when this bond forms by insertion into a metal–nitrogen bond and when it forms by external attack on a bound alkene. These studies also have begun to reveal the factors that control the rates of the insertion processes.^10–12^ However, related information on the insertions of alkenes into metal–oxygen bonds is lacking.^13,14^

**Scheme 1:**
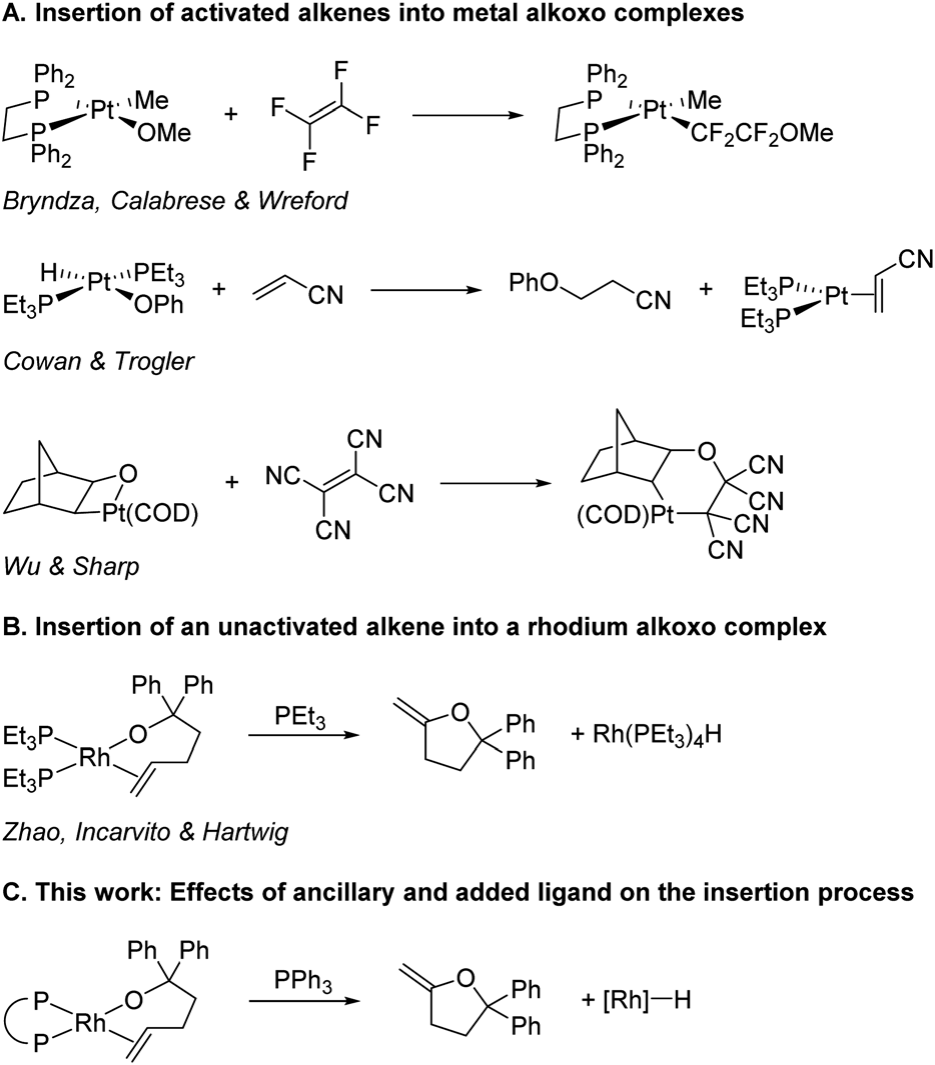
Insertion of alkenes into metal–alkoxo bonds.

The insertions of alkenes into metal–oxygen bonds has been demonstrated to occur during several important catalytic reactions,^15–26^ including the classic Wacker oxidation under conditions of low chloride concentration,^27–32^ but examples of discrete complexes that undergo insertions of olefins into metal–alkoxide bonds are rare and have occurred in most cases with activated alkenes. For example, tetrafluoroethene inserts into the platinum(ii)–methoxo bond of Pt(DPPE)(OMe)(Me) (Scheme 1A),^33^ acrylonitrile inserts into the platinum(ii)–phenoxo bond in Pt(PEt_3_)_2_(OPh)(H),^34^ and tetracyanoethene inserts into the Pt(II)–O bond of a platinaoxetane.^35^ Yet, the only direct observation of the insertion of an unactivated alkene into a transition-metal–alkoxo bond is the reaction of PEt_3_-ligated rhodium(i) alkoxo complexes that insert coordinated alkenes (Scheme 1B).^36^ No discrete complexes of any metal containing other types of ancillary ligands have been reported that undergo this elementary reaction.

We report a series of rhodium(i) alkoxo alkene complexes that contain a range of bidentate phosphine ligands and that undergo insertion of the alkene to form cyclised product. With these compounds, we have been able to reveal several effects of the ancillary diphosphine ligand and external ligand on the rate of the insertion of alkenes into the rhodium alkoxides. First, the steric properties of the ancillary ligand strongly affect the rate of the insertion, as well as the mechanism of the reaction after insertion; second, the electronic properties of the ligand affect the rate of insertion such that more electron-poor ligands lead to faster reactions than do more electron-rich ligands; third, free ligand (PPh_3_), which was added to bind the resulting Rh(i) hydride to form a stable product, appears to promote dissociation of the product from the metal. Experimental and computational data imply that the insertion of the alkene into the rhodium–alkoxo bond is endothermic and is driven by displacement of the organic product by PPh_3_ after β-hydride elimination when the steric congestion around the metal center is low. In contrast, product dissociation occurs without prior association of PPh_3_ when the steric congestion around the metal is higher.

## Results and discussion

### Synthesis and insertion reactions of rhodium alkoxo olefin complexes

**Fig. 1:**
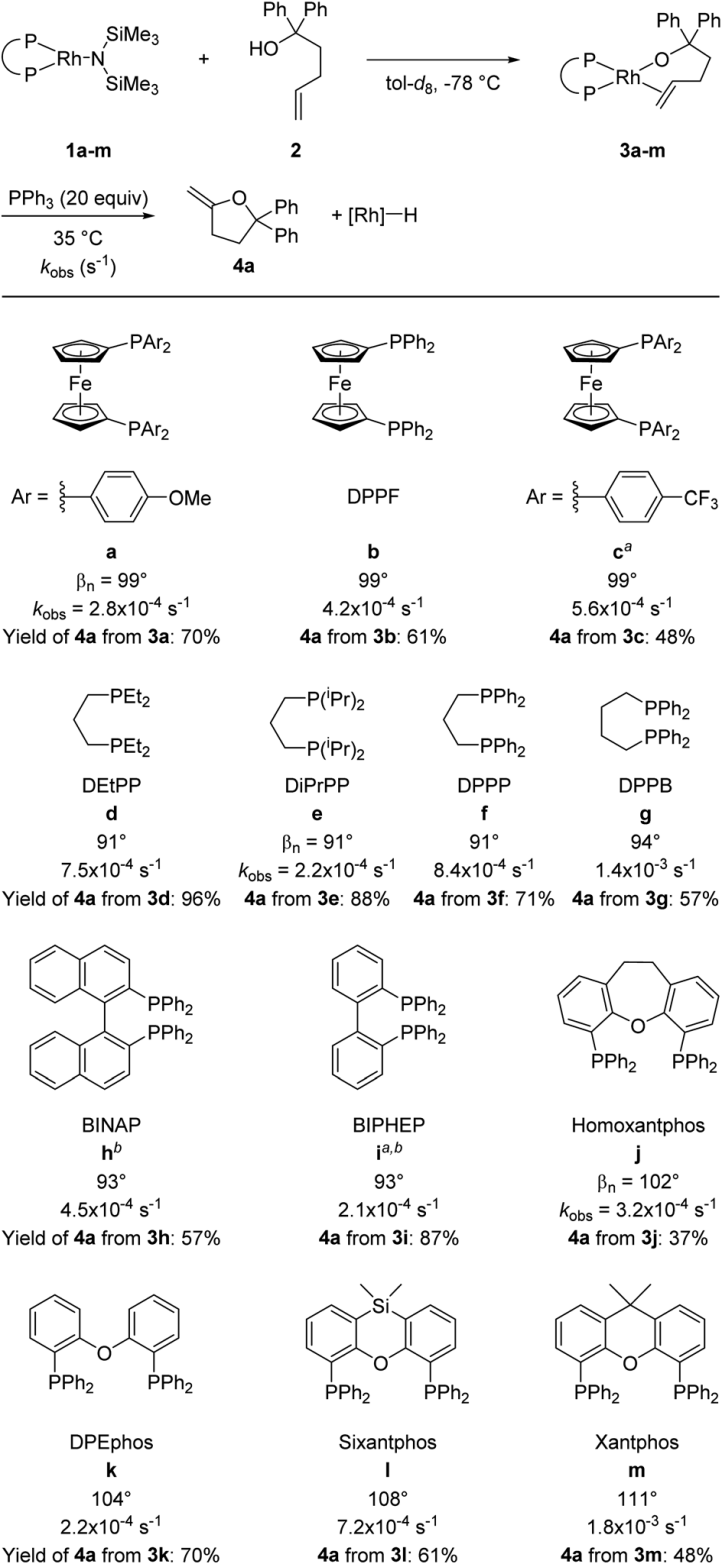
Synthesis and reaction of rhodium alkoxo alkene complexes **3a–m**. Rate constants refer to the reaction of alkoxo–alkene complexes **3a–m** at 35 °C in tol-d_8_ with 20 equiv. of added PPh_3_. Bite angles are those from published work.^37,38^ Yields were determined by ^1^H NMR spectroscopy using 1,3,5-trimethoxybenzene (TMB) as internal standard. ^*a*^THF-*d*_8_ used as solvent; ^*b*^0 °C was used as the reaction temperature.

A series of alkoxorhodium complexes shown in Fig. 1 was generated at low temperature. Addition of alcohol **2** to rhodium(i) silylamido diphosphine complexes **1a–m** (see ESI‡ for the synthesis and characterisation of **1a–m**) at −78 °C quantitatively generated alkoxo alkene complexes **3a–m**. Complexes **3a–m** were characterised *in situ* by ^1^H and ^31^P{^1^H} NMR spectroscopy at −40 °C. This set of complexes contain a series of diphosphine ligands with varying backbones connecting the phosphorus atoms and substituents on the phosphorus atoms that alter their structural and electronic properties.

**Scheme 2:**
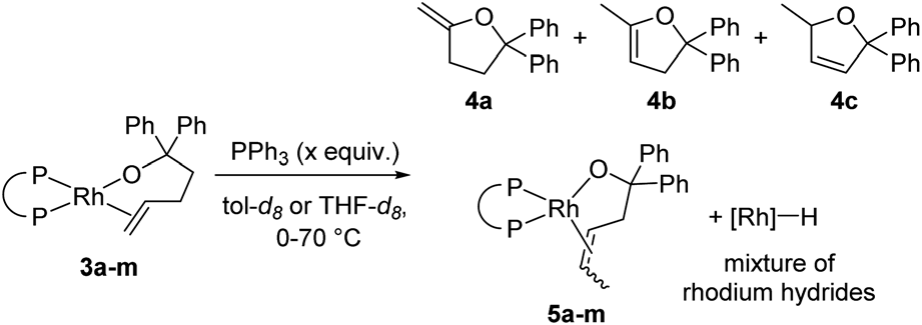
Products of the reaction of alkoxo alkene complexes **3a–m**.

Warming the alkoxo alkene complexes **3a–m** led to the formation of furan products **4a–c**, isomerised alkoxo alkene complexes **5a–m**, and rhodium hydride species (Scheme 2). Methylenetetrahydrofuran **4a** is the direct product from insertion of the alkene moiety of **3a–m** into the rhodium–alkoxo bond and subsequent β-hydride elimination. Dihydrofurans **4b-c** are likely formed by reaction of rhodium hydride byproducts with the alkene moiety of **4a**.^15^ Isomerisation of the double bond in **3a–m** gave alkoxo alkene complexes **5a–m** with an internal alkene bound to rhodium. These complexes did not undergo alkene insertion. The combination of alkoxo alkene complex **3f** and an equivalent of [Rh(DPPP)(PPh_3_)H] yielded a mixture of products including 52% of the isomeric alkoxo alkene complex **5f**. This result indicates that rhodium hydride byproducts induce isomerisation of the complex of the terminal alkene to that of the internal alkene.

To occupy open coordination sites on the rhodium hydride species and thereby limit hydride-induced isomerisation reactions, reactions were run in the presence of free phosphine ligand. Triphenylphosphine was selected as the external ligand because it prevented isomerisation without reacting with alkoxo alkene complexes **3a–m** (see Fig. S97‡). With 1–20 equivalents of PPh_3_ added to the reaction of DPPP-ligated complex **3f**, the amount of isomerised furans was reduced, and the amount of methylene furan **4a** depended on the amount of added PPh_3_. Reactions conducted with higher concentrations of PPh_3_ formed less isomerised product than those conducted with lower concentrations of PPh_3_. The yield of **4a** was also higher from reactions with a more strongly binding added ligand, such as PEt_3_ or P(OPh)_3_, instead of PPh_3_ (see Table S3‡). Nevertheless, we conducted subsequent experiments with added PPh_3_ because it did not displace the ancillary ligands that bind more weakly and would not create the ambiguity of whether the reactions occurred by forming complexes of the monodentate added ligand, particularly PEt_3_.

Reactions of **3a–m** with 20 equivalents of PPh_3_ at 35 °C gave **4a** in 37–96% yields, with complexes **3d** and **3e** giving the highest yields of furan products (96% and 88% respectively). Broadly speaking, reactions of complexes containing more electron-rich diphosphines gave higher yields of **4a** than did those of complexes containing more electron-poor diphosphines, presumably due to the difference in activity of the rhodium hydride product toward isomerisation.

### Kinetic studies on the insertion reactions and effects of ancillary ligand properties on rates

Kinetic studies were conducted to elucidate the reaction mechanism and analyse the effects of ancillary and added ligands on the reaction. Initial investigations focused on the DPPF-ligated complex **3b**. Increasing the concentration of the rhodium complex by a factor of 3.8 did not change the observed rate constant for the decay of **3b** (4.2 × 10^−4^ s^−1^ at 0.041 M and 4.1 × 10^−4^ s^−1^ at 0.151 M). This result is consistent with a first-order dependence of the rate on the concentration of the alkoxo alkene complex **3b** and the observed exponential decay curves.

The effect of solvent polarity was evaluated by conducting reactions in toluene-*d*_8_ and in THF-*d*_8_ with 10 equivalents of PPh_3_ at 35 °C. In toluene-*d*_8_, the reaction occurred with a rate constant of 4.2 × 10^−4^ s^−1^ (*t*_1/2_ = 28 min), while in THF-*d*_8_, the reaction occurred with a rate constant of 3.5 × 10^−4^ s^−1^ (*t*_1/2_ = 33 min). Thus, the reaction is slightly (1.2×) faster in toluene-*d*_8_ than in THF-*d*_8_, but the difference in polarity of the solvent did not have a large effect on the rate. Toluene-*d*_8_ was used as solvent for reactions of all the complexes unless the solubility of the complex was too low. This lack of a significant solvent effect is consistent for a reaction pathway proceeding through migratory insertion of the alkene into the rhodium alkoxide and is inconsistent with a pathway involving dissociation of the rhodium–alkoxo bond and nucleophilic attack of the resulting alkoxide on the coordinated alkene moiety.

To determine the effect of added PPh_3_ on the reactions of **3a–m**, the decay of DPPF-ligated complex **3b** and DPPP-ligated complex **3f** were monitored at 35 °C in the presence of 1, 10, and 20 equivalents of PPh_3_. The rate constants for the decay of both **3b** and **3f** were indistinguishable from each other (**3b**: 4.1 ± 0.1 × 10^−4^ s^−1^; *t*_1/2_ = 30 min; **3f**: *k*_obs_ = 1.0 ± 0.1 × 10^−3^ s^−1^), indicating that the reactions of alkoxo alkene complexes **3b** and **3f** are zero order in added PPh_3_, respectively, when more than 1 equivalent of PPh_3_ is added.

Having confirmed that PPh_3_ is not interfering with the migratory insertion process, we analysed the effects of the ancillary ligands in **3a–m** on this reaction by comparing the rate constants for reaction of a series of complexes containing different ligands. The effect of the electronic properties of the ancillary ligands was revealed by comparing the rate constants for complexes **3a–c**, which contain three DPPF derivatives as ligands. The kinetic data (**3a** containing *p*-OMe ligand: *k*_obs_ = 2.8 × 10^−4^ s^−1^; **3b** containing *p*-H ligand: *k*_obs_ = 4.2 × 10^−4^ s^−1^; **3c** containing *p*-CF_3_ ligand: *k*_obs_ = 5.6 × 10^−4^ s^−1^) indicate that the less electron-rich alkoxo alkene complexes react faster than more electron-rich alkoxo alkene complexes.

Two studies describing the effect of electronic properties of an ancillary ligand on the rate of insertion into a metal–amido complex have been previously reported.^10,12^ Wolfe reported the intramolecular insertion of alkenes into bisphosphine-ligated palladium(ii)–amido bonds.^10^ The electronic effect of the ancillary ligand on this process was examined for complexes of the same three DPPF derivatives as in the study presented here. Complexes containing less electron-rich ancillary ligands underwent migratory insertion of the alkene into the metal–amido bond faster than complexes containing more electron-rich ligands. The similarity of the system studied by Wolfe and the system reported here allows a direct comparison of the electronic effects of these phosphines on the two insertion processes. The *p*-CF_3_ containing amido complex reacted more than five times faster than the analogous *p*-OMe containing complex, while the *p*-CF_3_ containing alkoxo complex reacted only two times faster than the analogous *p*-OMe containing complex. These olefin insertions into metal–alkoxo bonds in the current work are less sensitive to the electronic properties of the ligand than are the prior migratory insertions into metal–amido bonds.

Our group reported an additional migratory insertion of an alkene into a metal–amido bond in which the effect of the electronic properties of an ancillary LX-type ligand from cyclometalation of an aryl dialkyl phosphine at the ortho carbon of the aryl group was investigated.^12^ A substituent at the para position of the aryl ring was varied, and this single change in substituent from –CF_3_ to –OMe led to nearly an order of magnitude difference in the rate constant for the elementary step of insertion. While this reaction system is significantly different from the one reported here, it implies, together with the work of Wolfe,^10^ that the migratory insertion of alkenes into metal–alkoxo bonds are less sensitive to changes in the electronic properties of the ancillary ligands than the insertion of alkenes into metal–amido bonds. Together, these data begin to provide a general trend for migratory insertions into late metal–heteroatom bonds.^10,12,39^

Complexes containing diphosphines with biphenyl linkages between the two phosphorus atoms (**3h–i**) reacted faster than the structurally related DPPB-ligated complex **3g**, which has an alkyl linkage containing the same number of carbon atoms between the phosphorus atoms of the ancillary ligand as are present in the biaryl linkages of biaryl bisphosphines. The rates of reaction of the complexes of biphenyl diphosphines were even too fast to measure conveniently at 35 °C; thus, the rate constants for reaction of these two complexes were obtained at 0 °C (**3h**: *k*_obs_ = 4.5 × 10^−4^ s^−1^; **3i**: *k*_obs_ = 2.1 × 10^−4^ s^−1^). These high rates of reaction can be explained by the lower electron donation of the biaryl backbones in the ancillary ligands of complexes **3h–i** than of the ferrocenyl or aliphatic backbones in complexes **3a–g**.

The effect of the ligand bite angle was illustrated by comparing the rate constants for the structurally related complexes **3j–m** (**3j**: 3.2 × 10^−4^ s^−1^; **3k**: 2.2 × 10^−4^ s^−1^; **3l**: 7.2 × 10^−4^ s^−1^; **3m**: 18 × 10^−4^ s^−1^). The complexes containing the ligands with the largest bite angles in this set, sixantphos-ligated **3l** und xantphos-ligated **3m**, reacted faster than homoxantphos-ligated and DPEphos-ligated complexes **3j** and **3k**. Similarly, the observed rate constant for DPPB-ligated complex **3g** (14 × 10^−4^ s^−1^) was higher than the rate constant for DPPP-ligated complex **3f** (8.4 × 10^−4^ s^−1^). These results show that an increase in the size of the bite angle accelerates the insertion of alkenes into metal–alkoxo bonds. Analogous results were previously described for the insertion of alkenes into palladium(ii)–amido,^10,12^ palladium(ii)–methyl,^40^ and Group V metal–hydrido bonds.^41^ A larger bite angle likely increases the steric congestion of the alkene complexes prior to insertion and therefore makes insertion more feasible.

### Effect of free phosphine on the reaction rates

**Scheme 3:**
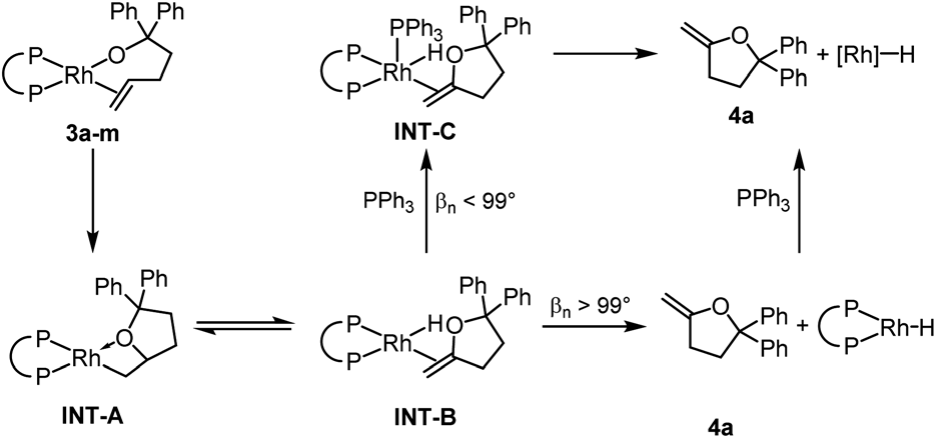
Proposed mechanism for the reaction of **3a–m** to form **4a**.

The relative rates for the reactions of DEtPP-ligated complex **3d** and DiPrPP-ligated complex **3e** initially appeared to contradict the steric effect deduced from analysing the effect of bite angle on the reactions of complexes **3j–m**. In the presence of 20 equivalents of PPh_3_, the less sterically hindered complex **3d** reacted faster (*k*_obs_ = 7.5 × 10^−4^ s^−1^ at 35 °C) than the more sterically hindered complex **3e** (*k*_obs_ = 2.2 × 10^−4^ s^−1^ at 35 °C). As discussed later in this paper, PPh_3_ appears to displace the product of insertion and β-hydrogen elimination from the hydrido alkene complex **INT-B** when the bite angle is small. Because this displacement reaction would be associative (Scheme 3), coordination of PPh_3_ to **INT-B** containing DEtPP would be faster than coordination to the more sterically congested complex containing DiPrPP, causing the reaction of DEtPP-ligated **3d** to be faster than reaction of DiPrPP-ligated **3e** when conducted in the presence of free PPh_3_. This proposal also implies that displacement of the product, rather than migratory insertion, is rate-limiting for the more hindered complex **3e** in the presence of PPh_3_.

To test this hypothesis, the rates for the reactions of complexes **3d-e** were measured in the absence of PPh_3_ to eliminate effects of the free ligand on product displacement. The reaction rates were too slow to be measured at 35 °C for both complexes, so the reactions were conducted at 70 °C. At this temperature, the more sterically congested complex **3e** (*k*_obs_ = 8.8 × 10^−4^ s^−1^ at 70 °C) reacted significantly faster than its less sterically congested counterpart **3d** (3.0 × 10^−4^ s^−1^ at 70 °C). This result is consistent with increased steric bulk on the ancillary ligands leading to faster rates of insertion and supports the hypothesis that increased steric hindrance of the ancillary ligand increases the rate of the insertion step, but retards the association of PPh_3_, thereby leading to slower reaction of **3e** than of **3d** in the presence of PPh_3_.

The large accelerating effect of PPh_3_ that was observed for the reactions of **3d-e** was further investigated by conducting the reactions of other complexes in the presence and absence of PPh_3_. Similar rate accelerating effects were observed for complexes **3f–i**, but the reaction was zero order in PPh_3_ in each case when more than 1 equivalent of PPh_3_ was added. In contrast, the rate constants for the reactions of complexes **3b** and **3m** were the same in the presence and absence of added PPh_3_. Thus, the reaction rates of rhodium diphosphine alkoxo alkene complexes containing ancillary ligands with natural bite angles greater than 99° were unaffected by added PPh_3_, whereas the reaction rates of rhodium diphosphine alkoxo alkene complexes containing ancillary ligands with bite angles smaller than 99° were faster in the presence of PPh_3_ than in the absence of this ligand, but zero order in PPh_3_ in the presence of 1–20 equivalents of PPh_3_.

We propose that **4a** dissociates from **INT-B** without prior association of PPh_3_ for complexes containing ancillary ligands with bite angles >99° as in **3b** and **3m** (Scheme 3). In complexes containing ancillary ligands with bite angles <99°, the steric congestion around the metal centre is smaller. Therefore, direct dissociation from **INT-B** is less favoured, and coordination of free PPh_3_ (**INT-C**) leads to displacement of methylene-tetrahydrofuran **4a**. Although the added PPh_3_ does not affect the rate of the reactions of the complexes containing ligands with bite angles greater than 99°, it does affect the products of the reaction. Binding of the external ligand to the resulting rhodium hydride species inhibits isomerisation of **4a** (*vide supra*).

To gain additional information on the role of PPh_3_, the rate constant for reaction of **3f** was measured in the presence of 0.1 to 1 equivalent of PPh_3_. In each reaction, the initial reaction rate was the same, but the rate of conversion sharply decreased (more than that of an exponential decay) during the reaction, and the time at which the rate decreased was shorter for reactions with lower initial concentrations PPh_3_ (see Table S1 and Fig. S2‡). These data indicate that PPh_3_ is consumed in the reaction and once no free PPh_3_ is present, the reaction rate is smaller, as described above for reactions conducted in the absence of PPh_3_.

### Computational studies on the insertion process

**Fig. 2:**
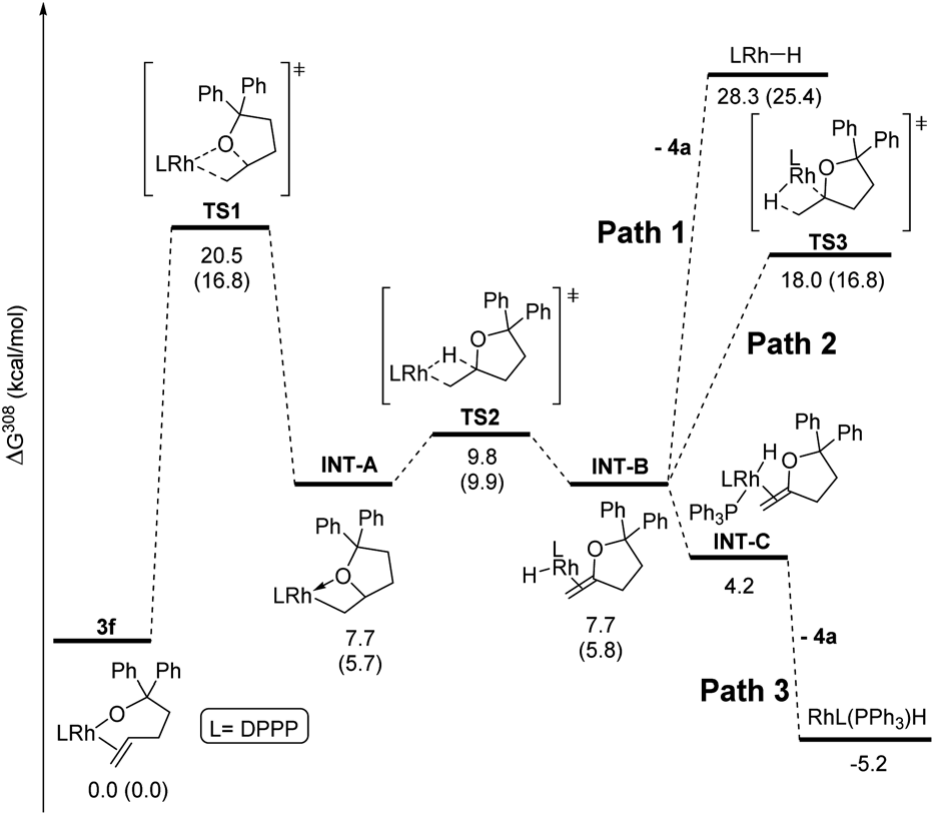
Calculated reaction pathway for the reaction of complex **3f**. Energies were calculated at the PBE0 level of theory. Additional energy values from single point calculations at the M06 level of theory are given in parentheses.

Computational studies were conducted with the Gaussian16 suite of programs^42^ to analyse the feasibility of the proposed mechanism. The pathway for the reaction of DPPP-ligated complex **3f** was calculated as a model for complexes containing ancillary ligands with bite angles smaller than 99° (Fig. 2). Geometries and energies were calculated at the PBE0-D3(BJ) level of theory^43–48^ using a mixed def2 basis set^49,50^ (see ESI‡ for details), including SMD solvent correction for toluene^51^ and thermal correction to 308 K. Additional single point calculations were performed for selected structures at the M06-D3/SDD/6-311+G(d,p) level of theory^52–57^ including the same thermal and solvent corrections. This combination of functional and basis set has been previously employed to study a rhodium(i)-catalysed reaction including a migratory insertion step.^58^

The reaction of the complex containing DPPP was chosen for computational studies because this reaction was one influenced by the presence of PPh_3_. The activation free energy for the migratory insertion of the alkene moiety in **3f** into the rhodium–alkoxo bond was calculated to be 20.5 kcal mol^−1^ (**TS1**). The free energy of the resulting rhodium alkyl complex **INT-A** is predicted to be 7.7 kcal mol^−1^ higher than that of **3f**. These values correspond well to those reported for the migratory insertion of ethylene and propene into rhodium hydroxo complexes.^59^ The free energy barrier for β-hydride elimination from **INT-A** is only 2.1 kcal mol^−1^ (**TS2**), and the free energy of the product (**INT-B**) is identical to that of **INT-A**. β-Hydride elimination, therefore, is predicted to be rapid and reversible.

If PPh_3_ is present in the reaction mixture, it can readily bind to the rhodium centre of **INT-B** to form **INT-C** (Path 3). The dissociation of methylenetetrahydrofuran **4a** from **INT-C** is predicted to be exergonic by 9.0 kcal mol^−1^. Thus, our calculations predict that migratory insertion would be expected to be the rate limiting step in this reaction, and this prediction is consistent with our kinetic data on the effect of added ligand on the rate of the overall process.

In the absence of PPh_3_, Path 3 cannot occur. Direct dissociation of **4a** from **INT-B** leads to the combination of rhodium hydride and alkene products that are computed, together, to lie 28.3 kcal mol^−1^ uphill of the starting complex (Path 1). This high energy makes isomerisation processes possible by reinsertion of the rhodium hydride moiety into the double bond of the alkene ligand (**TS3**, Path 2). Indeed, **4a** formed in only 13% yield when the reaction of **3f** was conducted without added PPh_3_, and internal alkene **4b** and the complex **5f** containing the isomerised alkoxo olefin were formed as the major products in 42% each (see ESI‡ for details).

The energy values obtained from a second single point calculation with the M06 functional were indistinguishable from the results that were calculated with the PBE0 functional. Most important, the energy of the transition state leading to the isomerised rhodium alkyl species **TS3** is predicted to be nearly identical to the energy of the transition state **TS1** for the initial migratory insertion.

### Characterisation of rhodium alkyl complexes generated in the absence of PPh_3_

The reaction of DPPP-ligated complex **3f** was monitored by ^31^P{^1^H} NMR spectroscopy in the absence of PPh_3_ at 35 °C. Complexes corresponding to two sets of doublets of doublets at 48.4 and 16.3 ppm, and 50.4 and 12.5 ppm accumulated. The coupling constants for these two species were nearly identical. Therefore, we assume that these complexes are either diastereomers or constitutional isomers in which the rhodium centre is bound to different carbon atoms on the product methyltetrahydrofuran scaffold. Similar ^31^P{^1^H} NMR signals were observed during the reaction of the BINAP-ligated complex **3h** (see ESI‡). In this case, the ^31^P{^1^H} NMR spectrum contained four sets of peaks, presumably corresponding to two sets of diastereomers resulting from the axial chirality of the BINAP ligand. For both ligands, the complexes accumulated over time when the reaction was monitored at 35 °C, but decayed after the reaction was run to completion at 70 °C. Thus, the observed complexes are potential intermediates to the final product **4a** or to the side products **4b-c** resulting from isomerisation observed in the absence of PPh_3_.

Information about the identity of these complexes was deduced from a series of NMR experiments. ^31^P{^1^H} DOSY NMR spectroscopy showed that the diffusion constants of the complexes are almost the same as the diffusion constants of the alkoxo alkene complexes **3f** and **5f**, suggesting that they are a similar size and likely monomeric. No hydrides of appropriate intensity were detected by ^1^H NMR spectroscopy at time points at which the complexes had accumulated, and no coupling between the small hydride peaks and the ^31^P{^1^H} NMR signals was detected by ^31^P{^1^H}-^1^H-HMBC NMR spectroscopy. Thus, the observed complexes are not hydrido alkene complexes like **INT-B**.

**Scheme 4:**
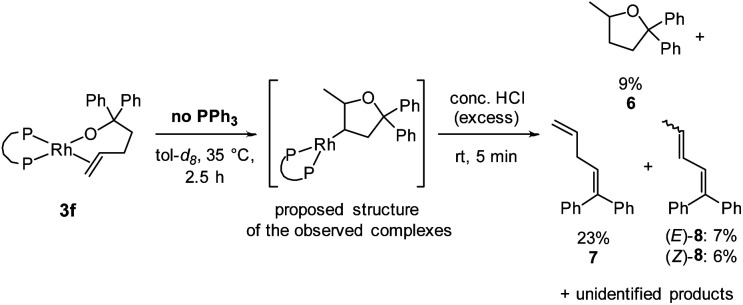
Formation of tetrahydrofuran **6** by quenching the reaction of alkoxo alkene complex **3f** with an excess of hydrochloric acid. Yields were determined by ^1^H NMR spectroscopy using TMB as internal standard. The proposed structure of the observed complexes is also shown.

To test whether the observed species are rhodium alkyl complexes, we added acid to the solution containing them. After generation of these species from complex **3f**, a large excess of concentrated hydrochloric acid was added. The complexes were fully consumed, and 5-methyl-2,2-diphenyltetrahydrofuran^60^ (**6**) was formed, albeit in 9% yield, as determined by ^1^H NMR spectroscopy using TMB as internal standard (Scheme 4). Alkenes **7** and **8** were identified as side products of this reaction in a combined yield of 36% among further unidentified side products. The formation of **7** and **8** can be explained by decomposition of complexes **3f** and **5f** in the presence of strong acid, respectively. The observation of tetrahydrofuran **6** as a product of this reaction suggests that protodemetalation of a rhodium–alkyl complex occurred. Rhodium–alkyl complexes are common intermediates but rarely observed and characterised in catalytic reactions.^61^ We attempted to determine the position at which the protodemetalation occurred by adding deuterated acid instead of protic acid, but the ^2^H NMR spectrum of the crude reaction mixture was too complex to determine the site of deuteration of the organic product. GC/HRMS analysis of the crude reaction mixture showed a product with the expected mass of **6-d1** which further confirms the hypothesis that the observed species are rhodium alkyl complexes that undergo demetalation in the presence of strong acid.

Further information about the connectivity between rhodium and the organic ligand in the observed complexes was instead deduced from a series of NMR experiments. No coupling to rhodium was observed in the ^13^C{^1^H} NMR spectrum when the terminal carbon of the double bond in alcohol **2** was labelled with ^13^C.^61^ This result indicates that the observed complex is not **INT-A**. In addition, monitoring the reaction of the alkoxo alkene complex from a variant of alcohol **2** containing deuterated phenyl groups by ^2^H NMR spectroscopy indicated the absence of rhodium–aryl interactions. Based on these experiments and the observation of two similar species in the reaction, it is likely that the complexes are the diastereomeric mixture of rhodium alkyl complexes in which the rhodium is bound to the 4-position of the methyltetrahydrofuran scaffold (see Scheme 4 for the structure and the ESI‡ for a more detailed discussion).^62^ This proposal that the observed complex contains rhodium bound to the 4-position of the tetrahydrofuryl unit is consistent with isomerisation processes dominating in the absence of added ligand when the bite angle of the ancillary ligand is small.

## Conclusions

The studies described here reveal the effects of bound ancillary and free external ligands on the first set of well-characterised complexes containing varied ancillary ligands that insert alkenes into rhodium–alkoxo bonds. Our results demonstrate that the insertion process is faster for complexes containing ancillary ligands that are more electron-poor and for which the bite angle is larger than for complexes with ancillary ligands that are more electron rich and for which the bite angle is smaller. Varying the electronic properties of the ancillary ligand appears to have a smaller effect on the rate of reaction than varying the steric properties of the ancillary ligand. The observed trends are similar to previously reported trends for the insertion of olefins into late transition metal–amido and –alkyl bond.^10,12,39,40^ Together, these results provide a comprehensive picture of the influences of ancillary ligands on migratory insertion processes of olefins.

Added ligand was found to affect the reaction in several ways. First, this external ligand affected the product distribution by limiting isomerisation processes caused by rhodium hydride byproducts. Second, the rates of reactions of complexes with bite angles smaller than 99° increased significantly in the presence of PPh_3_, but the rates of reactions of complexes containing ligands with bite angles greater than 99° were unaffected by the presence of PPh_3_. Computational results indicate that this difference in the effect of added PPh_3_ is because PPh_3_ changes the mechanism of product release from dissociative, in the absence of added ligand, to associative, in the presence of added ligand. The higher barrier for dissociation of the organic product than reinsertion of the rhodium hydride into the alkene bond enables isomerisation processes to dominate during reactions conducted without added PPh_3_. A rhodium complex that is likely an alkyl intermediate in this isomerisation process accumulated during reactions without added PPh_3_. We expect these findings to accelerate the design of new reactions involving migratory insertions of alkenes.

## Conflicts of interest

There are no conflicts to declare.

## Supplementary Material

SC-011-D0SC04402D-s001
