# Supplementary material for: Effects of ligands on the migratory insertion of alkenes into rhodium–oxygen bonds
Source: Chem Sci. 2020 Sep 8;11(38):10449–56. doi: 10.1039/d0sc04402d (PMC8162318; doi:10.1039/d0sc04402d)
Supplement: SC-011-D0SC04402D-s001 [file SC-011-D0SC04402D-s001.pdf]

Electronic Supplementary Material (ESI) for Chemical Science.
This journal is © The Royal Society of Chemistry 2020

Supporting Information for

Effects of Ancillary and External Ligands on the Insertion of Alkenes into Rhodium Oxygen Bonds.

Casseday P. Richers[a‡], Sven Roediger[b‡], Victor Laserna[b], John F. Hartwig[a,b*]

[a] *Department of Chemistry, University of Illinois, Urbana-Champaign, 600 South Matthews Avenue, Urbana, Illinois 61801, United States, and* [b] *Department of Chemistry, University of California, Berkeley, California 94720, United States*.

Table of Contents

Materials and Methods S2

Synthesis of Diphosphine Ligands S3

Synthesis of alkoxo alkene complexes **2a-2m** S13

Characterization of Rhodium Silyl Amido Complexes, **1a-m** and Alkoxo Alkene Complexes, **3a-m**. S15

Kinetic Data of Alkoxo Alkene Complexes S33

Spectroscopic Data........ S43

Discussion of the proposed structure of the reaction intermediates:........ S91

Computational data........ S93

References S108

Materials and Methods

All reactions were performed under an inert atmosphere in oven-dried glassware using standard Schlenk techniques or inside a nitrogen-filled glove box, unless otherwise noted. $[Rh(COE)_2Cl]_2$ was prepared according to literature procedures. $RhCl_3$, deuterated solvents, 1,3,5-trimethoxybenzene (TMB), PPh_3, and other commercially mono- and diphosphines were purchased from Aldrich, Acros, or Strem and were used as received. Solvents were dried by filtration through activated alumina or distilled from sodium benzophenone ketyls. NMR spectra were recorded on Bruker AVQ-400, AV-500, DRX-500, Neo-500, and AV-600 instruments. ^{1}H NMR chemical shifts (δ) are reported in ppm relative to the residual solvent signal. ^{31}P NMR chemical shifts were reported relative to an external H_3PO_4 (85% aqueous) sample. High resolution mass spectral data were obtained from the University of California, Berkeley Mass Spectrometry Laboratory.

Synthesis of Diphosphine Ligands

Synthesis of DPPF derivatives.

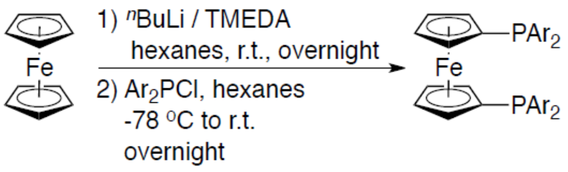

Synthesis of 1,1'-Bis[bis(4-methoxyphenyl)phosphino]ferrocene, (**a**).[1]

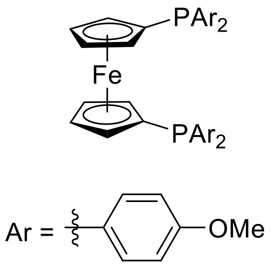

Ferrocene (1.0 g, 5.4 mmol, 1.0 equiv) was weighed in a 20 ml vial and transferred to a 100 ml Schlenk flask. The Schlenk flask was equipped with a magnetic stir bar, sealed with a septum, and connected to a Schlenk manifold. The flask was purged of air by successively applying vacuum and dry N_2 (3x), and then placed under an N_2 atmosphere. Dry pentane (30 ml) was added to the Schlenk flask via syringe. The resulting solution was stirred at room temperature. A solution of *n*BuLi in hexanes (7.4 ml, 1.6 M, 12 mmol, 2.2 equiv) was added slowly to the ferrocene solution. A solution of TMEDA (distilled from sodium, 1.8 ml, 1.4 g, 12 mmol, 2.2 equiv) in pentane (30 ml) was added slowly to the mixture of ferrocene and ^{n}BuLi. The resulting solution was stirred overnight at room temperature under an N_2 atmosphere. An orange solid precipitated. The solution was placed in a dry ice / acetone bath and cooled to -78 °C. Bis(4- methoxyphenyl)chlorophosphine, (4-MeO-C_6H_4)$_2$PCl (3.4 g, 12 mmol, 2.2 equiv), was weighed inside a glovebox in a 20 ml vial. The compound was dissolved in approximately 20 ml of dry THF and loaded into a 50 ml syringe. A rubber septum was placed on the needle of the syringe and the syringe was removed from the glovebox. The solution of (4-MeO-C_6H_4)$_2$PCl in THF was added slowly to the solution of 1,1'-dilithioferrocene / 2 TMEDA at -78 °C. The dry ice / acetone bath was removed, and the reaction was allowed to stir at room temperature overnight. The resulting solution was filtered through Celite into a second 250 ml Schlenk flask equipped with a magnetic stir bar and a Schlenk filter. The Schlenk filter was removed from the Schlenk flask under a high N_2 pressure and replaced with a rubber septum. The solvent was removed from the filtrate under vacuum. The residue was washed with pentane. A yellow solid was obtained (2.0 g, 3.0 mmol, 55 % yield). ^{1}H NMR (400 MHz, CD_2Cl_2): δ 7.25 (s, broad, 8H), 6.85 (d, J = 7 Hz, 8H), 4.27 (s, broad, 4H), 3.79 (s, broad, 4H) ppm. ^{13}C{^{1}H} NMR (101 MHz, CD_2Cl_2): δ 160.69, 135.19 (d, J = 21 Hz), 130.77 (d, J = 8 Hz), 114.22 (d, J = 8 Hz), 78.50 (d, J = 8 Hz), 74.00 (d, J = 15 Hz), 72.77, 55.65 ppm. ^{31}P{^{1}H} NMR (162.0 MHz, CD_2Cl_2): δ -20.51 ppm.

Synthesis of 1,1'-Bis[bis(4 trifluoromethylphenyl)phosphino]ferrocene, (**c**).[1]

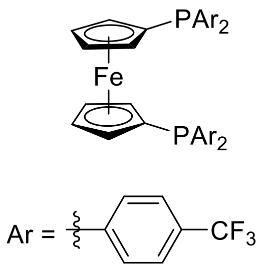

Ferrocene (1.8 g, 9.6 mmol, 1.0 equiv) was weighed in a 20 ml vial and transferred to a 100 ml Schlenk flask. The Schlenk flask was equipped with a magnetic stir bar, sealed with a septum, and connected to a Schlenk manifold (by a rubber hose). The flask was purged of air by successively applying vacuum and dry N2 (3x), and then placed under an N_2 atmosphere. Dry pentane (30 ml) was added to the Schlenk flask via syringe. The resulting solution was stirred at room temperature. A solution of *n*BuLi in hexanes (13.2 ml, 1.6 M, 21 mmol, 2.2 equiv) was added slowly to the ferrocene solution. A solution of TMEDA (distilled from sodium, 3.2 ml, 2.5 g, 21 mmol, 2.2 equiv) in pentane (30 ml) was added slowly to the mixture of ferrocene and *n*BuLi. The resulting solution was stirred overnight at room temperature under an N_2 atmosphere. An orange solid precipitated. The solution was placed in a dry ice / acetone bath and cooled to -78 °C. Bis(4- trifluoromethylphenyl)chlorophosphine, (4-CF_3-C_6H_4)$_2$PCl (7.6 g, 21 mmol, 2.2 equiv), was weighed inside a glovebox in a 20 ml vial. The compound was dissolved in approximately 20 ml of dry THF and loaded into a 50 ml syringe. A rubber septum was placed on the needle of the syringe and the syringe was removed from the glovebox. The solution of (4-CF_3-C_6H_4)$_2$PCl in THF was added slowly to the solution of 1,1'-dilithioferrocene / 2 TMEDA at -78 °C. The dry ice / acetone bath was removed, and the reaction was allowed to stir at room temperature overnight. The resulting solution was filtered through Celite into a second 250 ml Schlenk flask equipped with a magnetic stir bar and a Schlenk filter. The Schlenk filter was removed from the Schlenk flask under a high N_2 pressure and replaced with a rubber septum. The solvent was removed from the filtrate under vacuum. The residue was washed with pentane. A yellow solid was obtained (2.0 g, 3.2 mmol, 33 % yield). ^{1}H NMR (400 MHz, CD_2Cl_2): δ 7.57 (d, *J* = 8 Hz, 8H), 7.41 (t, *J* = 7 Hz, 8H), 4.35 (m, 4H), 4.01 (d *J* = 4 Hz, *J* = 2 Hz, 4H) ppm. ^{13}C{^{1}H} NMR (101 MHz, CD_2Cl_2): δ 143.90 (d, *J* = 14 Hz), 134.27 (d, *J* = 20 Hz), 131.16 (d, *J* = 33 Hz), 125.53 (dt, *J* = 8 Hz, *J* = 4 Hz), 75.36 (d, *J* = 8 Hz), 74.48 (d, *J* = 15 Hz), 73.36 (d, *J* = 4Hz). ^{31}P{^{1}H} NMR (162.0 MHz, CD_2Cl_2): δ -20.05 ppm. ^{19}F{^{1}H} NMR (376.5 MHz, CD_2Cl_2): δ -62.95 ppm.

Synthesis of 1,3-Bis(dialkyllphosphino)propane derivatives.

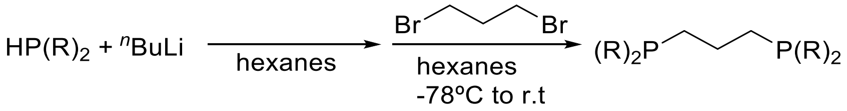

Synthesis of 1,3-Bis(diethylphosphino)propane, DEtPP (**d**).[2]

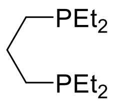

Diethylphosphine, Et_2PH, (1.0 g, 11 mmol, 2.0 equiv) was weighed inside a glovebox in a 3 ml vial. The compound was dissolved in 2 ml of pentane and transferred to a 50 ml Schlenk flask. Additional pentane (10 ml) was added to the flask. The flask was equipped with a magnetic stir bar. A solution of *n*BuLi in hexanes (1.6 M, 6.9 ml, 11 mmol, 2.0 equiv) was added to the solution of Et_2PH at room temperature. The flask was stoppered and sealed from the box atmosphere. The resulting solution was stirred overnight at room temperature. A white solid precipitated. The Schlenk flask was removed from the glovebox and connected to a Schlenk manifold by a rubber hose. The hose and neck of the Schlenk flask were purged of air by applying vacuum and N_2 successively three times. The Schlenk flask was then opened to an N_2 atmosphere and placed in a dry ice / acetone bath. 1,3-Dibromopropane (0.57 ml, 1.1 g, 5.5 mmol, 1.0 equiv) was loaded into a syringe and slowly added to the Et_2PLi solution at -78 °C. The reaction was slowly warmed to room temperature and stirred overnight. The product solution was filtered through Celite into a second 50 ml Schlenk flask, equipped with a magnetic stir bar and fitted with a Schlenk filter. The solid was washed with additional pentane (3 x 10 ml). The Schlenk filter was removed from the second Schlenk flask under a high N_2 pressure and replaced with a rubber septum. The pentane was removed from the filtrate under vacuum. A yellow oil was obtained. The crude product was distilled under vacuum to give a colorless oil (0.78 g, 3.5 mmol, 64% yield). ^1H NMR (400 MHz, C_6D_6): δ 1.57 (m, 2H), 1.37 (m, 4H), 1.24 (m, 8H), 0.98 (m, 12H) ppm. $^{13}C\{^1H\}$ NMR (101 MHz, C_6D_6): δ 28.94 (dd, $J^{C\text{-}P}$ = 15 Hz, $J^{C\text{-}P}$ = 10 Hz), 23.03 (t, $J^{C\text{-}P}$ = 15 Hz), 19.47 (d, $J^{C\text{-}P}$ = 13 Hz), 9.90 (d, $J^{C\text{-}P}$ = 13 Hz) ppm. $^{31}P\{^1H\}$ NMR (162 MHz, C_6D_6): δ -23.96 ppm.

Synthesis of 1,3-Bis(diisopropylphosphino)propane, DiPrPP (**e**).[3]

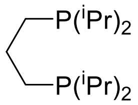

Diisopropylphosphine, *i*Pr_2PH (10 g of 10 % wt in hexanes, 1.0 g, 8.5 mmol, 2.0 equiv) was weighed inside a glovebox in a 3 ml vial. The compound was dissolved in 2 ml of pentane and transferred to a 50 ml Schlenk flask. Additional pentane (10 ml) was added to the flask. The flask was equipped with a magnetic stir bar. A solution of *n*BuLi in hexanes (1.6 M, 5.8 ml, 9.3 mmol, 1.1 equiv) was added to the solution of *i*Pr_2PH at room temperature. The flask was stoppered and sealed from the box atmosphere. The resulting solution was stirred overnight at room temperature. A white solid precipitated. The Schlenk flask was removed from the glovebox and connected to a Schlenk manifold by a rubber hose. The hose

and neck of the Schlenk flask were purged of air by applying vacuum and N_2 successively three times. The Schlenk flask was then opened to an N_2 atmosphere and placed in a dry ice / acetone bath. 1,3-Dibromopropane (0.43 ml, 0.86 g, 4.2 mmol, 1.0 equiv) was loaded into a syringe and slowly added to the $^iPr_2PLi$ solution at -78 °C. The reaction was slowly warmed to room temperature and stirred overnight. The product solution was filtered through Celite into a second 50 ml Schlenk flask, equipped with a magnetic stir bar and fitted with a Schlenk filter. The solid was washed with additional pentane (3 x 10 ml). The Schlenk filter was removed from the second Schlenk flask under a high N_2 pressure and replaced with a rubber septum. The pentane was removed from the filtrate under vacuum. A yellow oil was obtained. The crude product was distilled under vacuum to give a colorless oil (0.55 g, 2.0 mmol, 47% yield).

Lithium diisopropylphosphide was also prepared by the addition of chlorodiisopropylphosphine to lithium metal. Lithium metal (4.09 g, 589 mmol, 6.00 equiv) was weighed inside an argon-filled glovebox and loaded into a 250 ml Schlenk flask equipped with a magnetic stir bar. The flask was stoppered with a septum, sealed from the box atmosphere and removed from the glovebox. The Schlenk flask was connected to a Schlenk manifold by a rubber hose that was purged of air by applying vacuum and argon successively three times. The Schlenk flask was opened an argon atmosphere, charged with dry THF (100 ml), and placed in an ice bath. Chlorodiisopropylphosphine (15 g, 98 mmol, 1.0 equiv) was added slowly to the solution of lithium metal (the reaction is exothermic). The reaction was stirred overnight. The ice bath was allowed to warm to room temperature. The colorless solution became green. ($^{31}P\{^1H\}$ NMR: δ -4.75 ppm.). The heterogeneous solution was cannulated into a glass Schlenk filter connected to a second 250 ml Schlenk flask that had been purged of air and moisture. The Schlenk filter was replaced with a rubber septum under a high argon pressure, and the Schlenk flask was subsequently placed in a dry ice / acetone bath. 1,3- Dibromopropane (4.5 ml, 9.0 g, 44 mmol, 0.45 equiv) in dry THF (20 ml) was slowly added to the solution of lithium diisopropylphosphide at -78 °C. The resulting solution was stirred overnight. The dry ice / acetone bath was allowed to warm to room temperature. The crude product was concentrated under vacuum and transferred to a two neck round bottom flask, fitted with a short-path distillation apparatus and purged of air and moisture. The crude product was distilled under vacuum to yield a colorless, pyrophoric oil (11 g, 41 mmol, 83% yield). $^{31}P\{^1H\}$ NMR (162 MHz, THF-d_8): δ 2.37 ppm.

Synthesis of Homoxantphos

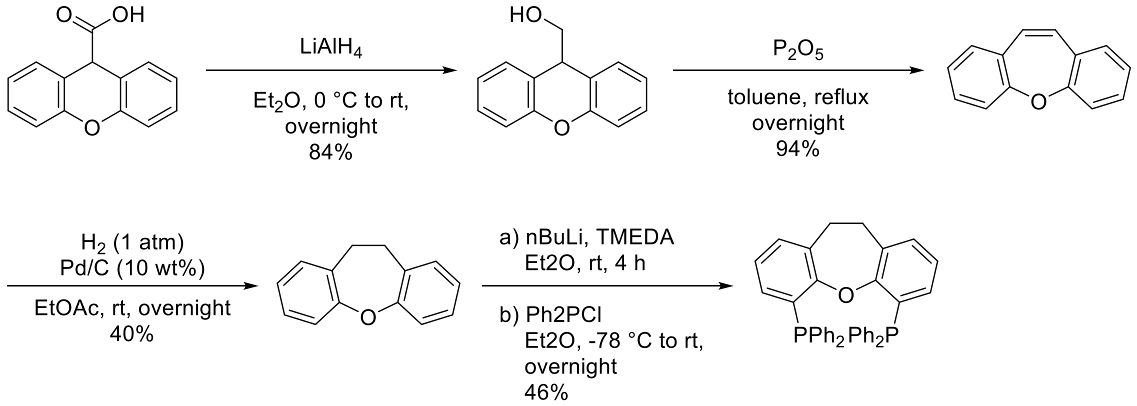

Synthesis of 9-Xanthylmethanol.[4]

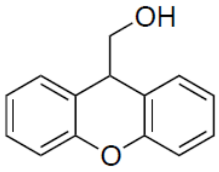

Lithium aluminum hydride, $LiAlH_4$ (3.8 g, 100 mmol, 3.0 equiv) was weighed inside a glovebox in a 20 ml vial, and transferred, as a solid, to a 500 ml three-neck round bottom flask. The flask was equipped with a magnetic stir bar, charged with 200 ml of dry Et_2O, and fitted with a 24/40-to-barb flow control adapter and two rubber septa. The three-neck flask was then removed from the glovebox and connected to a Schlenk manifold. The hose connecting the flask to the manifold was purged of air by successively applying vacuum and N_2 (3x), and the flask was opened to an N_2 atmosphere, which was continuously bled through a bubbler. The reaction vessel was placed in an ice bath. Xanthene-9-carboxylic acid (7.5 g, 33 mmol, 1.0 equiv) was weighed inside the glovebox in a 20 ml vial. The compound was dissolved in 30 ml of dry Et_2O and loaded into a 50 ml syringe. The syringe was stoppered with a rubber septum and removed from the glovebox. The solution of xanthene-9-carboxylic acid was slowly added to the solution of $LiAlH_4$ at 0 °C (caution: the reaction is vigorous and expels H_2 gas). The reaction was warmed to room temperature and then stirred overnight. The reaction was quenched by sequential addition of 10 ml H_2O, 10 ml 3 N NaOH(aq), and 10 ml H_2O and then filtered after all of the gray solid had become white. The filtered solid was washed with additional Et_2O (3 x 60 ml). The combined filtrates and washings were concentrated under vacuum to yield a white solid, which was recrystallized from cold pentane (5.9 g, 28 mmol, 84% yield). ^1H NMR (400 MHz, $CDCl_3$): δ 7.29 (triplet of doublets, overlapping signals, J = 8 Hz, J = 1 Hz, 2H), 7.27 (d, overlapping signals, J = 8 Hz, 2H), 7.17 (d, J = 8 Hz, 4H), 7.12 (triplet of doublets, J = 8 Hz, J = 1 Hz), 3.99 (t, J = 6 Hz, 1H), 3.61 (d, J = 5 Hz, 2H), 2.52 (s, 1H) ppm. $^{13}C\{^1H\}$ NMR (100.6 MHz, $CDCl_3$): δ 152.18, 129.15, 128.13, 123.16, 122.12, 116.45, 68.79, 41.99 ppm.

Synthesis of Dibenz[*b*,*f*]oxepine.[5]

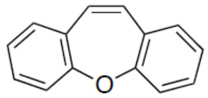

P_2O_5 (26.8 g, 189 mmol, 4.00 equiv) was weighed inside a glovebox in a 20 ml vial and transferred as a solid to a 500 ml three-neck round bottom flask, equipped with a magnetic stir bar, a 24/40-to-barb flow control adapter, and a reflux condenser. Dry toluene (250 ml) was added to the flask, and the flask was stoppered with two rubber septa and sealed from the box atmosphere. The three-neck flask was then removed from the glovebox and connected to a Schlenk manifold by a rubber hose. The hose was purged of O_2 by successively applying vacuum and N_2, and the flask was then opened to an N_2 atmosphere. 9- Xanthylmethanol (10 g, 47 mmol, 1.0 equiv) was weighed inside the glovebox in a 20 ml vial, dissolved in toluene (30 ml), and loaded into a 50 ml syringe. The syringe was stoppered with a rubber septum and removed from the glovebox. The solution of 9-xanthylmethanol was added to the solution of P_2O_5 at room temperature. The reaction was then heated to reflux and stirred overnight. The reaction was cooled to 0 °C, and water (100 ml) was added to the reaction vessel. The reagents were mixed thoroughly. The aqueous phase was separated from the organic phase and extracted with Et2O (3 x 100 ml). The organic fractions were combined and concentrated under vacuum. The product crystallized from a small amount of MeOH as a white solid (8.6 g, 44 mmol, 94% yield). ^{1}H NMR (600 MHz, $CDCl_3$): δ 7.26 (triplet of doublets, J = 8 Hz, J = 2 Hz, 2H), 7.14 (m, overlapping signals, 4H), 7.06 (triplet of doublets, J = 8 Hz, J = 2 Hz, 2H), 6.66 (s, 2H). ^{13}C{^{1}H} NMR (150.9 MHz, $CDCl_3$): δ 157.36, 130.59, 130.09, 129.94, 139.39, 124.99, 121.38.

Synthesis of 10,11-dihydrodibenzo[*b,f*]oxepine.[5]

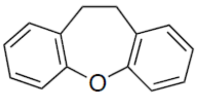

A 100 ml Schlenk flask, equipped with a magnetic stir bar, was charged with dibenz[*b*,*f*]oxepine (4.43 g, 22.8 mmol, 1.0 equiv). Palladium on carbon (10% wt, 1.6 g) was added to the flask as a solid. The flask was stoppered and connected to a Schlenk manifold by a rubber hose. The flask was then purged of O_2 by applying vacuum and N_2 successively three times, and then opened to N_2. The flask was charged with 50 ml of dry THF. A bubbler was connected to the Schlenk flask by a hose and needle, and the flask was then closed to N_2. A balloon filled with hydrogen and fitted with a syringe and 12 inch needle was connected to the flask. The tip of the needle was placed in the solution of dibenz[*b*,*f*]oxepine so that the solution could be sparged with H_2. This was repeated two more times. The bubbler was disconnected. A fourth balloon was filled with hydrogen and connected to the Schlenk flask through a needle. The reaction was stirred overnight at room temperature under an atmosphere of H_2. The balloon was removed. The reaction was diluted with EtOAc and filtered through Celite using a glass frit. The filtrate was collected and the solvent removed. The crude product was purified by column chromatography (eluent = 100% hexanes) to yield a colorless oil (1.8 g, 9.2 mmol. 40% yield). ^{1}H NMR (400 MHz, THF-d_8): δ 7.14-7.03 (overlapping signals, m, 6H), 6.94

(ddd, J = 8 Hz, J = 6 Hz, J = 2 Hz, 4H), 3.04 (s, 4H) ppm. $^{13}C\{^{1}H\}$ NMR (150.9 MHz, $CDCl_3$): δ 158.03, 132.72, 131.09, 127.99, 124.54, 121.63, 31.96 ppm.

Synthesis of 10,11-dihydro-4,5-bis(diphenylphosphino)dibenzo[*b,f*]oxepine, Homoxantphos (**k**).

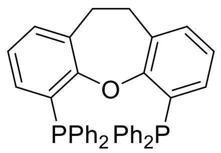

10,11-dihydrodibenzo[*b,f*]oxepine (2.02 g, 10.3 mmol, 1.00 equiv) was weighed in a 20 ml vial and transferred as a solid to a 250 ml Schlenk flask. The Schlenk flask was equipped with a magnetic stir bar and then connected to a Schlenk manifold by a rubber hose. The flask was purged of O_2 by successively applying vacuum and N_2, and then placed under an N_2 atmosphere. Dry Et_2O (80 ml) and TMEDA (distilled from sodium, 3.37 ml, 2.63 g, 22.6 mmol, 2.20 equiv) were added to the flask by syringe. The flask was placed in a dry ice / acetone bath, and a solution of *n*BuLi in hexanes (1.6 M, 14.2 ml, 23 mmol, 2.2 equiv) was slowly added to the solution of 10,11-dihydrodibenzo[*b,f*]oxepine and TMEDA by syringe. The reaction was warmed to room temperature and stirred overnight. The flask was then placed in a dry ice acetone / bath, and Ph_2PCl (4.1 ml, 5.0 g, 23 mmol, 2.2 equiv) was added to the reaction flask by syringe. The reaction was warmed to room temperature and stirred overnight. The solvent was removed under vacuum, and the resulting residue was dissolved in CH_2Cl_2. The CH_2Cl_2 solution was filtered through Celite into a second 250 ml Schlenk flask equipped with a magnetic stir bar. The collected solid was washed with additional CH_2Cl_2 (3 x 20 ml). The Schlenk filter was removed from the second Schlenk flask under a high N_2 pressure and replaced with a rubber septum. The solvent was removed under vacuum. The crude product was washed with pentane (2 x 30 ml) and dried under vacuum. A white solid was obtained (2.7 g, 4.8 mmol, 46 % yield). ^{1}H NMR (400 MHz, THF-d_8): δ 7.28-7.16 (overlapping signals, m, 12H), 7.15-7.10 (overlapping signals, m, 8H), 7.08 (dd, J = 8 Hz, J = 1 Hz, 2H), 6.82 (t, J = 8 Hz, 2H), 6.48 (dd, J = 8 Hz, J = 1 Hz, 2H), 3.07 (s, 4H) ppm. $^{31}P\{^{1}H\}$ NMR (161.9 MHz, THF-d_8): δ -16.39 ppm.

Synthesis of Sixantphos.

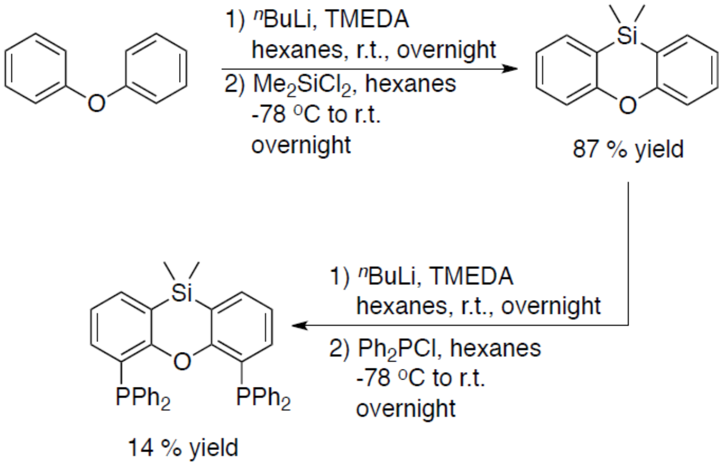

Synthesis of 10,10-Dimethylphenoxasilin.[6]

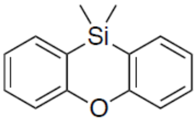

A 500 ml three-neck round bottom flask was equipped with a magnetic stir bar, a 24/40-to-barb flow control adapter, and two rubber septa. The flask was connected to a Schlenk manifold by a rubber hose and purged of O_2 and water by successively applying vacuum and N_2. The flask was then placed under an N_2 atmosphere and charged with dry pentane (300 ml), diphenyl ether (18.6 ml, 20.0 g, 0.118 mol, 1.00 equiv) and TMEDA (38.5 ml, 30.0 g, 0.258 mol, 2.20 equiv). A dry ice / acetone bath was prepared, and the flask was cooled to -78 °C. A solution of ^{n}BuLi in hexanes (1.6 M, 162 ml, 0.259 mol, 2.20 equiv) was added to the solution of diphenyl ether and TMEDA at -78 °C. The resulting reaction was warmed to room temperature and stirred overnight. A dark red solution formed. The reaction was cooled to -78 °C, and dichlorodimethylsilane (31.5 ml, 33.4 g, 0.259 mol, 2.20 equiv) was added. The reaction was warmed to room temperature and stirred overnight. Water (100 ml) was added to the reaction, and the organic and aqueous layers were separated. The aqueous phase was washed with Et_2O (3 x 100 ml). The organic fractions were combined and concentrated under vacuum. A dark red oil was obtained. The crude product was purified by column chromatography (eluent = 100% hexanes) to yield a white solid (23.1 g, 0.102 mol, 86.4 % yield). ^1H NMR (500 MHz, $CDCl_3$): δ 7.76 (dd, J = 7 Hz, J = 2 Hz, 2H), 7.64 (m, 2H), 7.46 (d, J = 8 Hz, 2H), 7.36 (td, J = 7 Hz, J = 1 Hz, 2H), 0.72 (s, 6H) ppm. $^{13}C\{^1H\}$ NMR (125.8 MHz, $CDCl_3$): δ 159.77, 134.05, 131.28, 122.74, 119.20, 118.15, -0.21 ppm.

Synthesis of Sixantphos (**I**).[6]

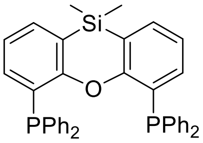

10,10-Dimethylphenoxasilin (3.56 g, 15.7 mmol, 1.00 equiv) was weighed in a 20 ml vial and transferred as a solid to a 250 ml Schlenk flask. The Schlenk flask was equipped with a magnetic stir bar and then connected to a Schlenk

manifold by a rubber hose. The flask was purged of O_2 by successively applying vacuum and N_2, and placed under an N_2 atmosphere. Dry Et_2O (80 ml) and TMEDA (distilled from sodium, 5.15 ml, 4.02 g, 34.6 mmol, 2.20 equiv) were added to the flask by syringe. The flask was placed in a dry ice / acetone bath, and a solution of *n*BuLi in hexanes (1.6 M, 21.6 ml, 34.6 mmol, 2.20 equiv) was slowly added to the solution of 10,10-dimethylphenoxasilin and TMEDA by syringe. The reaction was warmed to room temperature and stirred overnight. The flask was then placed in a dry ice acetone / bath, and Ph_2PCl (7.1 ml, 8.7 g, 39 mmol, 2.5 equiv) was added to the reaction flask by syringe. The reaction was warmed to room temperature and stirred overnight. The solvent was removed under vacuum, and the resulting residue was dissolved in CH_2Cl_2. The CH_2Cl_2 solution was filtered through Celite into a second 250 ml Schlenk flask equipped with a magnetic stir bar. The collected solid was washed with additional CH_2Cl_2 (3 x 20 ml). The Schlenk filter was removed from the second Schlenk flask under a high N_2 pressure and replaced with a rubber septum. The CH_2Cl_2 was removed under vacuum. The residue was dissolved in benzene and passed through a plug of silica gel. Crystallization from isopropanol afforded a white solid (1.3 g, 2.2 mmol, 14 % yield). ^1H NMR (400 MHz, $CDCl_3$): δ 7.52 (d, J = 7 Hz, 2H), 7.29 (overlapping signals, m, 12H), 7.22 (s, 8H), 7.03 (t, J = 7 Hz, 2H), 6.82 (d, J = 7 Hz, 2H), 0.57 (s, 6H) ppm. $^{31}P\{^1H\}$ NMR (202.2 MHz, $CDCl_3$): δ -17.62 ppm.

Synthesis of 1,1-diphenyl-4-penten-1-ol (**2**).

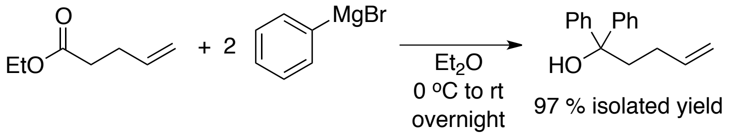

A 250 ml Schlenk flask, equipped with a magnetic stir bar, was connected to a Schlenk manifold by a rubber hose and purged of O_2 and moisture by successively applying vacuum and N_2. The flask was placed under an N_2 atmosphere and charged with 75 ml of dry Et_2O. Ethyl 4-pentenoate (11 ml, 10 g, 78 mmol, 1.0 equiv) was added to the flask by syringe. The flask was placed in an ice bath, and a solution of phenylmagnesium bromide in Et_2O (3 M, 57 ml, 170 mmol, 2.2 equiv) was added slowly to the reaction flask by syringe. The reaction was stirred overnight. The ice bath was allowed to warm to room temperature. A saturated solution of sodium sulfate (35 ml) was added to the flask. The aqueous and organic phases were separated, and the organic phase was washed with brine (3 x 75 ml). The organic phase was dried over anhydrous $MgSO_4$. The diethyl ether solvent was removed under a gentle vacuum (300 Torr). The colorless oil that was obtained was slowly solidified to a white solid (18 g, 76 mmol, 97 % yield). The product used without further purification. The ^1H NMR spectrum of the product showed no signs of byproducts. ^1H NMR (500.16 MHz, toluene-d_8): δ 7.31 (d, J^{H-H} = 7 Hz, 4H), 7.09 (t, J^{H-H} = 7 Hz, 4H, overlaps with solvent), 7.00 (t, J^{H-H} = 7 Hz, 4H, overlaps with solvent), 5.74 (ddt, J^{H-H} = 17 Hz, J^{H-H} = 10 Hz, J^{H-H} = 7 Hz, 1H), 4.95 (m, 2H), 4.92 (m, 2H), 2.11 (m, 2H, overlaps with solvent), 1.99 (dd, J^{H-H}

=15 Hz, $J^{H\text{-}H}$ = 7 Hz, 2H) 1.42 (s, 1H) ppm. $^{13}C\{^{1}H\}$ NMR (150.9 MHz, toluene-d_8) : δ 147.3, 139.0, 128.1, 126.7, 126.3, 114.5, 77.4, 40.8, 28.4 ppm.

Synthesis of alkoxo alkene complexes **2a-2m**

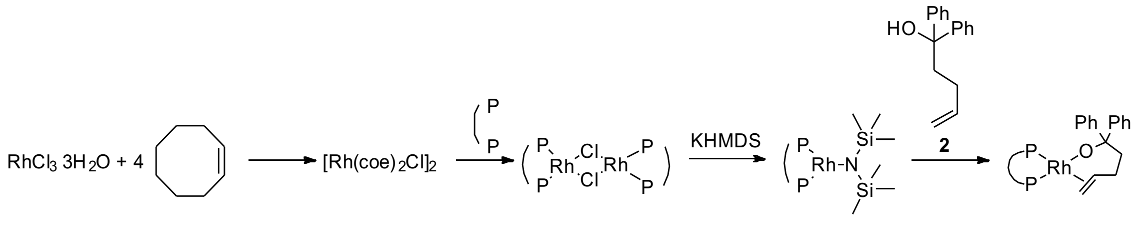

Preparation of $[Rh(coe)_2Cl]_2$.[7]

To a 3-neck round-bottomed flask containing $RhCl_3.3H_2O$ (175 mg, 0.670 mmol, 1.00 equiv) were sequentially added degassed water (1 mL), degassed iPrOH (4 mL) and cyclooctene (0.56 mL, 4.3 mmol, 6.4 equiv). The resulting dark red solution was heated under reflux for 2 h. Afterwards, the resulting orange suspension was cooled to room temperature. The precipitate was collected by filtration, washed with cold ethanol, washed with petroleum ether and dried in vacuo to give $[Rh(coe)_2Cl]_2$ (170 mg, 35 %) as yellow solid.

General method for the preparation of rhodium diphosphine chloro complexes.

$[Rh(COE)_2(\mu\text{-}Cl)]_2$ (200. mg, 0.279 mmol, 1.00 equiv) was weighed inside a glovebox in a 20 ml vial. The corresponding diphosphine **a**-**m** (0.279 mmol, 2.00 equiv) was weighed inside the same glovebox in a 20 ml vial. The weighed diphosphine was dissolved in 6 ml of dry THF and transferred to the vial containing the weighed $[Rh(COE)_2(\mu\text{-}Cl)]_2$. A magnetic stir bar was added to the vial. The vial was capped, and the reaction was stirred overnight at room temperature. The solution was concentrated under vacuum to a volume of approximately 1 ml and then diluted with Et_2O (~15 ml). The precipitated solid was collected on a glass frit and transferred to a tared 20 ml vial. The solid was dried under vacuum and used as obtained (yields 36-92%).

General method for the preparation of rhodium diphosphine silyl-amido complexes (**1a-m**).

Rhodium diphosphine chloride complexes, were weighed inside a glovebox in 20 ml vial (0.128 mmol, 1.0 equiv). THF (2 ml) and a magnetic stir bar were added to the vial. A solution of KHMDS (51.1 mg, 0.256 mmol, 2.0 equiv) in THF (5 ml) was prepared in a 20 ml separate vial and added to the rhodium diphosphine complex by pipet. The vial containing the two reagents was capped. The resulting solution was stirred overnight inside the glovebox at room temperature. The THF solvent was removed under vacuum. The residue was dissolved in pentane and filtered through Celite. The filtrate was collected, and the solvent removed under vacuum to yield a solid.

General method for the preparation of Rh alkoxo-alkene complexes (**3a-m**).

Rhodium diphosphine silylamido complex, **1a-m**, (0.018 mmol, 1 equiv) was weighed inside a glovebox in a 3 ml vial. The weighed complex was dissolved in toluene-d_8 (0.5 ml)/THF-d_8 and transferred to a screw cap NMR tube. 1,3,5-trimethoxybenzene (TMB) (1.2 mg) was added to the NMR tube as an internal standard. The screw capped NMR tube was sealed with a cap containing a septum. 1,1-Diphenyl-4-penten-1-ol was weighed inside the glovebox in a 3 ml vial and dissolved in 0.1 ml of toluene-d_8. The 1,1-diphenyl-4-penten-1-ol (4.3 mg, 0.018 mmol, 1 equiv) solution was loaded into a 250 µL syringe. The needle of the syringe was inserted into a rubber stopper, and both the syringe and the screw capped NMR tube were removed from the glovebox. The NMR tube was placed in a dry ice / acetone bath. An NMR probe was cooled to -50 °C. Initial ^{1}H and ^{31}P NMR spectra of the silylamido complexes, were collected at -50 °C. The NMR tube was ejected and placed in the dry ice / acetone bath. The 1,1-diphenyl-4-penten-1-ol solution was syringed into the NMR tube. The NMR tube was kept in the dry ice / acetone bath for 5 minutes and then shaken vigorously for 10 seconds before returning the sample to the dry ice / acetone bath. The tube has shaken two more times by this procedure. The solutions became dark red. NMR spectra of the red solution were collected at -50 °C.

General method for the preparation of Rh alkoxo-alkene complex, **5f**

Rhodium diphosphine silylamido complex, **1f**, (0.018 mmol, 1 equiv) was weighed inside a glovebox in a 3 ml vial. The weighed complex was dissolved in toluene-d_8 (0.5 ml). A mixture of Z/E 1,1-Diphenyl-3-penten-1-ol was weighed inside the glovebox in a 3 ml vial and dissolved in 0.1 ml of toluene-d_8. Both solutions were mixed and left stirring for 5 minutes. The dark silyl amido solution turned red. The solution was transferred into a screw cap NMR tube and measured at room temperature.

Characterization of Rhodium Silyl Amido Complexes, **1a-m** and Alkoxo Alkene Complexes, **3a-m**.

Rhodium Diphosphine Silylamido Complex, **1a**.

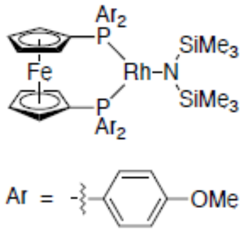

Dark blue solid (144 mg, 0.154 mmol, 55.2 % yield). ^{1}H NMR (499.4 MHz, toluene-d_8, 25 °C): δ 7.97 (s, broad, 8H), 6.71 (d, $J^{H\text{-}H}$ = 8 Hz, 8H), 4.59 (s, 4H), 3.95 (s, 4H), 3.15 (s, 12H), 0.65 (s, 18H) ppm. ^{13}C{^{1}H} NMR (125.7 MHz, THF-d_8, 25 °C): δ 161.6 (t, $J^{C\text{-}P}$ = 25.1 Hz), 136.0, 130.11, 111.4, 83.5 (t, $J^{C\text{-}P}$ = 28.9Hz), 75.9, 72.2, 55.4, 7.1 ppm. ^{31}P{^{1}H} NMR (202.2 MHz, toluene-d_8, 25 °C): δ 62.5 (d, $J^{P\text{-}Rh}$ = 182 Hz) ppm. Analysis Calculated For $C_{44}H_{54}FeNO_4P_2RhSi_2$: C, 56.35; H, 5.80; N, 1.49. Found: C, 56.57; H, 5.60; N, 1.12.

Rhodium Diphosphine Silylamido Complex, **1b**.

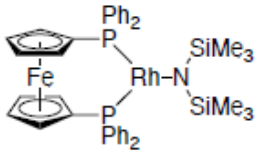

Dark green solid (221 mg, 0.270 mmol, 96.8 % yield). ^{1}H NMR (499.4 MHz, C_6D_6, 25 °C): δ 7.99 (m, 8 H), 7.05 (t, J = 7.5 Hz, 8 H), 7.01 (q, J = 7.5 Hz, 4 H), 4.45 (s, broad, 4H), 3.84 (s, broad, 4 H), 0.51 (s, 18 H) ppm. ^{13}C{^{1}H} NMR (125.7 MHz, C_6D_6, 25 °C) δ 138.7 (t, $J^{C\text{-}P}$ = 23 Hz), 134.6 (t, $J^{C\text{-}P}$ = 6 Hz), 130.0, 128.7, 81.9, (t, $J^{C\text{-}P}$ = 30 Hz), 76.0 (t, unresolved), 72.4 (t, unresolved), 7.3 ppm. ^{31}P{^{1}H} NMR (202.2 MHz, C_6D_6): δ 64.1 (d, $J^{P\text{-}Rh}$ = 183 Hz) ppm. Analysis Calculated For $C_{40}H_{46}FeNP_2RhSi_2$: C, 58.76; H, 5.67; N, 1.71. Found: C, 58.80; H, 5.62; N, 1.38.

Rhodium Diphosphine Silylamido Complex, **1c**.

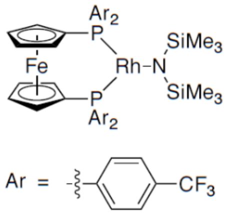

Dark green solid (100. mg, 0.092 mmol, 33 %). ^{1}H NMR (499.4 MHz, THF-d_8, 25 °C) δ 7.99 (m, 8 H), 7.63 (m, 8 H), 4.85(s, broad, 4H), 4.61 (s, broad, 4 H), 0.26 (s, 18 H) ppm. $^{31}P\{^{1}H\}$ NMR (202.2 MHz, C_6D_6, 25 °C) δ 66.3 (d, $J^{P\text{-}Rh}$ = 183 Hz) ppm.

Rhodium Diphosphine Silylamido Complex, **1d**.

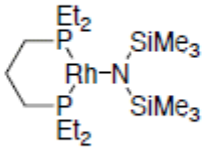

Purple solid (127 mg, 0.263 mmol, 94.1% yield). ^{1}H NMR (500.2 MHz, toluene-d_8, 25 °C) δ 1.45-1.25 (overlapping multiplets, 6H), 1.18 (s, broad, 4H) 1.03 (m, 12H), 0.97 (s, broad, 4H), 0.48 (s, 18H) ppm. $^{13}C\{^{1}H\}$ NMR (125.7 MHz, toluene-d_8, 25 °C) δ 24.0 (t, J = 20 Hz), 22.7 (t, $J^{C\text{-}P}$ = 13 Hz), 20.1, 9.1, 7.7 ppm. $^{31}P\{^{1}H\}$ NMR (202.2 MHz, toluene- d_8, 25 °C) δ 39.1 (d, $J^{P\text{-}Rh}$ = 157 Hz) ppm.

Rhodium Diphosphine Silylamido Complex, **1e**.

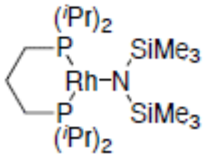

Purple solid (110 mg, 0.204 mmol, 73.1 % yield). ^{1}H NMR (499.4 MHz, toluene-d_8, 25 °C) δ 1.87 (s, broad, 2H), 1.67 (s, broad, 4H), 1.51 (s, broad, 4H), 1.18 (s, broad, 12H), 1.05 (s, broad, 12H), 0.51 (s, broad, 18H) ppm. $^{13}C\{^{1}H\}$ NMR (125.7 MHz, toluene-d_8, 25 °C) δ 27.58 (t, J = 12.8 Hz), 22.45, 19.55, 19.20, 19.17 (t, J = 17 Hz), 7.86 ppm. $^{31}P\{^{1}H\}$ NMR (202.2 MHz, toluene-d_8, 25 °C) δ 63.3 (d, $J^{P\text{-}Rh}$ = 164 Hz) ppm. Analysis Calculated For $C_{21}H_{52}NP_2RhSi_2$: C, 46.74; H, 9.71; N, 2.60. Found: C, 46.94; H, 9.58; N, 2.28.

Rhodium Diphosphine Silylamido Complex, **1f**.

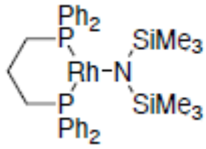

Dark blue solid (111 mg, 0.163 mmol, 58.9 % yield). ^{1}H NMR (499.92 MHz, THF-d_8, 25 °C): δ7.70 (m, broad, 8H), 7.19 (m, broad, 12H), 2.39 (s, broad, 2H), 2.03 (m, broad, 4H), 0.12 (s, broad, 18H) ppm. $^{13}C\{^{1}H\}$ NMR (125.7 MHz, THF-d_8, 25 °C): δ 138.1 (t, J^{C-P} = 23 Hz), 133.7 (t, J = 5.0 Hz), 130.2, 128.7 (t, J = 5.0 Hz), 26.6 (t, J^{C-P} = 21.4 Hz), 21.3, 7.4 ppm. $^{31}P\{^{1}H\}$ NMR (202.2 MHz, C_6D_6, 25 °C): δ 40.5 (d, J^{P-Rh} = 169 Hz) ppm. Analysis Calculated For $C_{33}H_{44}NP_2RhSi_2$: C, 58.66; H, 6.56; N, 2.07. Found: C, 58.92; H, 6.19; N, 1.87.

Rhodium Diphosphine Silylamido Complex, **1g**.

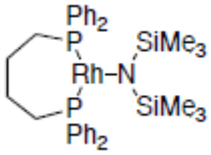

Dark blue solid (114 mg, 0.165 mmol, 59.2 % yield). ^{1}H NMR (399.7 MHz, THF-d_8, 25 °C): δ 7.70 (m, 8H), 7.34(q, J^{H-H} = 7 Hz, 4H), 7.30 (q, J^{H-H} = 7 Hz, 8H), 2.41 (s, broad, 4H), 1.87 (m, broad, 4H), 0.02 (s, 18H) ppm. $^{13}C\{^{1}H\}$ NMR (125.7 MHz, THF- d_8, 25 °C): δ 138.8 (t, J^{C-P} = 21.4 Hz), 133.5, 129.9, 128.65, 29.5 (t, J^{C-P} = 17.6 Hz), 24.8, 6.8 ppm. $^{31}P\{^{1}H\}$ NMR (202.3 MHz, THF-d_8, 25 °C): δ 59.7 (d, J^{P-Rh} = 171 Hz) ppm. Analysis Calculated For $C_{34}H_{46}NP_2RhSi_2$: C, 59.20; H, 6.72; N, 2.03. Found: C, 59.24; H, 6.69; N, 2.30.

Rhodium Diphosphine Silylamido Complex, **1h**.

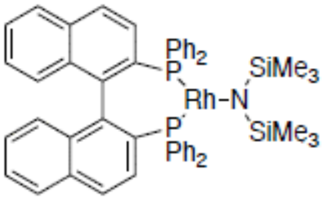

Dark green solid (222 mg, 0.251 mmol, 89.8 % yield). ^{1}H NMR (125.7 MHz, THF-d_8, 25 °C): δ 8.30 (s, broad, 4H), 7.87 (s, broad, 4H), 7.55 (d, broad, J^{H-H} = 10 Hz, 2H), 7.50 (s, broad, 4H), 7.39 (s, broad, 4H), 7.30 (m, broad, 6H), 7.07 (m, broad, 2H), 6.95 (d, broad, J^{H-H} = 10 Hz, 2H), 6.57 (m, broad, 2H), 6.48 (m, broad, 2H), 0.36 (s, 18H) ppm. $^{13}C\{^{1}H\}$ NMR (125.7 MHz, THF- d_8, 25 °C): δ 138.8 (t, J^{C-P} = 6 Hz), 136.0 (t, J^{C-P} = 5 Hz), 135.5 (t, J^{C-P} = 26 Hz), 135.3 (t, J^{C-P} = 24 Hz), 134.6, 134.2 (m, broad, overlapping), 134.1 (m, broad, overlapping), 133.5 (t, J^{C-P} = 26 Hz), 130.7, 129.1, 128.9, 128.8, 128.7-128.4 (overlapping multiplets, 2C), 128.2, 127.1, 126.9 (t, J^{C-P} = 5 Hz), 126.7, 7.8 ppm. $^{31}P\{^{1}H\}$ NMR (202.2 MHz, THF-d_8, 25 °C): δ 57.8 (d, J^{P-Rh} = 174 Hz)

ppm. Analysis Calculated For $C_{50}H_{50}NP_2RhSi_2$: C, 67.78; H, 5.69; N, 1.58. Found: C, 67.89; H, 5.56; N, 1.63.

Rhodium Diphosphine Silylamido Complex, **1i**.

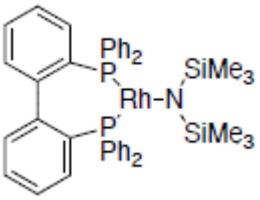

Dark green solid (90. mg, 0.11 mmol, 41 % yield). ^{1}H NMR (500.1 MHz, toluene- d_8, 25 °C): δ 8.18 (quartet, $J^{H\text{-}H}$ = 5 Hz, 4H), 7.98 (s, broad, 4H), 7.12 (t, $J^{H\text{-}H}$ = 5 Hz, 4H), 7.08 (quartet, $J^{H\text{-}H}$ = 5 Hz, 2H), 7.01 (t, $J^{H\text{-}H}$ = 5 Hz, 2H), 6.84 (overlapping signals, 6H), 6.73 (t, $J^{H\text{-}H}$ = 5 Hz, 2H), 6.54 (t, $J^{H\text{-}H}$ = 5 Hz, 2H), 6.45 (d, $J^{H\text{-}H}$ = 5 Hz, 2H), 0.65 (s, 18H) ppm. ^{13}C{^{1}H} NMR (125.7 MHz, THF-d_8, 25 °C): δ 144.6, 135.8, 135.4, 135.0, 133.6, 131.4, 130.6, 130.53, 130.0, 129.7, 128.9, 128.6, 127.8, 127.4, 126.5, 7.6 ppm. ^{31}P{^{1}H} NMR (202.2 MHz, THF- d_8, 25 °C): δ 57.8 (d, $J^{P\text{-}Rh}$ = 174 Hz) ppm. Analysis Calculated For $C_{42}H_{46}NP_2RhSi_2$: C, 64.19; H, 5.90; N, 1.78. Found: C, 64.12; H, 6.17; N, 1.55.

Rhodium Diphosphine Silylamido Complex, **1j**.

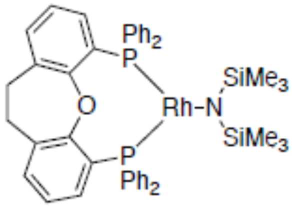

Dark green solid (232 mg, 0.280 mmol, 98 % yield). ^{1}H NMR (500.2 MHz, THF-d_8, 25 °C): δ 7.40 (s, broad, 8H), 7.25 (d, $J^{H\text{-}H}$ = 5 Hz, 2H), 7.21 (t, $J^{H\text{-}H}$ = 5 Hz, 4H), 7.08 (t, $J^{H\text{-}H}$ = 5 Hz, 8H), 6.83 (t, $J^{H\text{-}H}$ = 5 Hz, 2H), 6.26 (s, broad, 2H), 3.25 (s, 4H), 0.15 (s, 18H) ppm. ^{13}C{^{1}H} NMR (125.7 MHz, THF-d_8, 25 °C): δ 158.6, 135.1 (overlapping signals, 2C, one carbon signal is a triplet, $J^{C\text{-}P}$ = 25 Hz), 133.4, 133.3, 131.5, 129.9, 128.4 (t, $J^{Rh\text{-}P}$ = 5 Hz), 127.9 (t, $J^{Rh\text{-}P}$ = 23 Hz), 125.1, 31.6, 6.7 ppm. ^{31}P{^{1}H} NMR (202.2 MHz, THF-d_8, 25 °C): δ 57.4 (d, $J^{P\text{-}Rh}$ = 180 Hz) ppm. Analysis Calculated For $C_{44}H_{48}NOP_2RhSi_2$: C, 63.83; H, 5.84; N, 1.69. Found: C, 64.23; H, 6.13; N, 1.38.

Rhodium Diphosphine Silylamido Complex, **1k**.

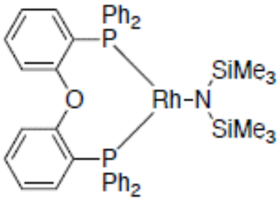

Dark green solid (154 mg, 0.192 mmol, 68.8 % yield). ^{1}H NMR (499.4 MHz, THF-d_8, 25 °C): δ 7.52 (s, broad, 8H), 7.32 (s, broad, 8H), 7.23 (s, broad, 8H), 6.71 (s, broad, 2H), 6.34 (s, broad, 2H), 0.02 (s, 18H) ppm. ^{13}C{^{1}H} NMR (125.8 MHz, THF-d_8, 25 °C): δ 160.5, 136.7 (t, J^{C-P} = 25 Hz), 134.0, 133.6, 131.4, 129.4, 129.1, 127.8, 127.3 (t, J^{C-P} = 24 Hz), 124.1, 122.6, 5.9 ppm. ^{31}P{^{1}H} NMR (202.2 MHz, THF-d_8, 25 °C): δ 56.3 (d, J^{P-Rh} = 181 Hz) ppm.

Rhodium Diphosphine Silylamido Complex, **1l**.

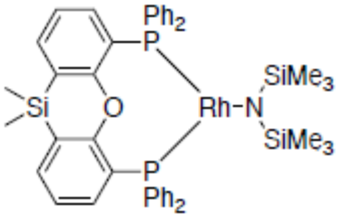

Dark green solid (168 mg, 0.191 mmol, 68.5 % yield). ^{1}H NMR (499.4 MHz, toluene-d_8, 25 °C): δ 8.02-7.03 (overlapping signals, broad, 26H), 0.77 (s, broad, 9H), 0.48 (s, broad, 9H) ppm. 0.35 (s, broad, 3H), 0.18(s, broad, 3H) ^{13}C{^{1}H} NMR (126 MHz, THF-d_8, 25 °C): δ 164.13, 136.58 (broad), 135.95, 133. 74, 133.59 (broad), 130.41 (broad), 129.59 (broad), 126.35, 125.82 (t, J = 23 Hz), 125.06 ppm. ^{31}P{^{1}H} NMR (202.2 MHz, toluene-d_8, 25 °C): δ 52.98 (d, J^{P-Rh} = 183 Hz) ppm. Analysis Calculated For $C_{44}H_{50}NOP_2RhSi_3$: C, 61.59; H, 5.87; N, 1.63. Found: C, 61.61; H, 6.12; N, 1.44.

Rhodium Diphosphine Silylamido Complex, **1m**.

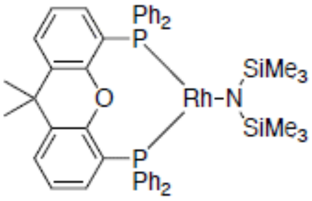

Dark green solid (152 mg, 0.181 mmol, 64.7 % yield). ^{1}H NMR (499.4 MHz, C_6D_6, 25 °C): δ 7.33 (s, broad, 4H), 7.08 (m, broad, 4H), 7.00 (d, broad, J^{H-H} = 7Hz, 4H), 6.80 (t, J^{H-H} = 7Hz, 2H), 6.69-6.57 (overlapping signals, m, 10H), 6.40 (s, broad, 2H), 1.50 (s, broad, 3H), 1.41 (s, broad, 3H),0.67 (s, broad, 9H), 0.23 (s, broad, 9H) ppm. ^{13}C{^{1}H} NMR (125.7 MHz, THF-d_8, 25 °C): δ

156.9, 137.4, 136.6 (broad), 133.5 (broad), 130.5 (broad), 129.6, 128.6 (broad), 126.4, 125.6 (t, J^{C-P} = 21 Hz), 125.1 (m), 38.0, 30.3, 22.8, 6.42, 2.8 ppm. $^{31}P\{^{1}H\}$ NMR (202.2 MHz, C_6D_6, 25 °C): δ 49.6 (d, J^{P-Rh} = 187 Hz) ppm. Analysis Calculated For $C_{45}H_{50}NOP_2RhSi_2$: C, 64.20; H, 5.99; N, 1.66. Found: C, 63.91; H, 5.89; N, 1.84.

Rhodium Alkoxo Alkene Complex, **3a**.

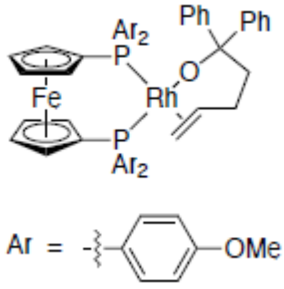

^{1}H NMR (499.4 MHz, toludene-d_8, -40 °C): δ 8.28 (s, broad, 2H), 8.00 (t, J =9 Hz, 2H), 7.91 (s, broad, 1H), 7.53 (d, J = 7 Hz, 2H), 7.34 (d, overlapping signals, J = 8 Hz, 2H), 7.32 (m, overlapping signals, 1H), 7.12-7.05 (overlapping signals, 4H), 7.02 (m, overlapping signals, 1H), 6.97 (m 1H), 6.88 (d, J = 8 Hz, 2H), 6.62 (m, overlapping signals, 4H), 6.54 (t, overlapping signals, J = 8 Hz, 4H), 5.14 (s, 1H), 4.63 (d, J = 13 Hz, 1H), 4.43 (s, 1H), 4.27 (s, 1H), 4.08 (s, 1H), 4.04 (m, broad, 1H), 3.89 (s, 1H), 3.84 (s, 1H), 3.77 (s, 1H), 3.72 (s, 1H), 3.40 (m, broad, 1H), 3.33 (s, 3H), 3.25 (s, 3H), 3.11 (s, 3H), 3.09 (s, 3H), 2.76 (m, overlapping signals, 2H), 2.38 (m, broad, 1H), 1.36 (m, broad,1H) ppm. $^{13}C\{^{1}H\}$ NMR (alkenyl carbons, 126 MHz, THF-d_8, -40 °C): δ 78.47 (m), 63.55 (m) ppm. $^{31}P\{^{1}H\}$ NMR (202.2 MHz, toluene-d_8, -40 °C): δ 48.6 (dd, J^{P-Rh} = 153 Hz, J^{P-P} = 49 Hz), 10.4 (dd, J^{P-Rh} = 165 Hz, J^{P-P} = 49 Hz) ppm.

Rhodium Alkoxo Alkene Complex, **3b**.

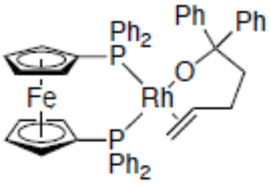

^{1}H NMR (499.7 MHz, toluene-d_8, -40 °C): δ 8.23 (s, broad, 2H), 8.05-7.85 (overlapping signals, broad, 4H), 7.47-6.78 (overlapping signals, broad, 24H), 5.16 (s, broad, 1H), 4.55 (d, broad, J = 13 Hz, 1H), 4.24 (s, broad, 1H), 4.21 (s, broad, 1H), 3.96 (s, broad, overlapping signals, 2H), 3.74 (s, broad, 1H), 3.71 (s, broad, 1H), 3.60 (s, broad, 1H), 3.49 (s, broad, 1H), 3.31 (s, broad, 1H), 2.69 (m, broad, 2H), 2.25 (s, broad, 1H), 1.68 (s, broad, 1H) ppm. $^{13}C\{^{1}H\}$ NMR (alkenyl carbons, 126 MHz, toluene-d_8, -40 °C): δ 72.67 (m), 64.26 (m) ppm. $^{31}P\{^{1}H\}$ NMR (202.2 MHz, toluene-d_8, -40 °C): δ 51.43 (dd, J^{P-Rh} = 151 Hz, J^{P-P} = 47 Hz), 14.33 (dd, J^{P-Rh} = 166 Hz, J^{P-P} = 47 Hz) ppm.

Rhodium Alkoxo Alkene Complex, **3c**.

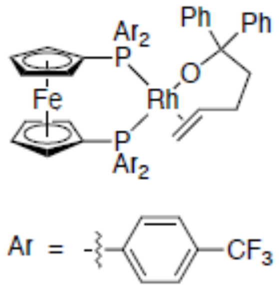

^{1}H NMR (499.7 MHz, THF-d_8, -40 °C): δ 8.25 (m, broad, 1H), 8.13 (s, broad, 1H), 7.93 (s, broad, 1H), 7.83 (m, 2H), 7.77 (d, J= 8 Hz, 2H), 7.63 (t, overlapping signals, J = 7.3 Hz, 3H), 7.47 (d, J = 7 Hz, 4H) 7.24 (dd, overlapping signals, J = 15 Hz, J = 8 Hz, 4H), 7.05 (d, J = 6 Hz, 2H), 6.92 (d, J = 7Hz, 2H), 6.83 (s, broad, 2H), 6.75 (s, broad, 2H), 5.23 (s, 1H), 4.76 (s, 1H), 4.54 (s, 1H), 4.51 (s, 1H), 4.33 (d, J = 13 Hz, 1H), 4.28 (s, 1H), 4.24 (s, 1H), 3.79 (s, broad, overlapping signal, 1H), 3.76 (s, 1H), 3.48 (s, 1H), 3.15 (s, broad, 1H), 2.48 (m, broad, 1H), 2.33 (m, 1H), 2.00 (s, broad, 1H), 1.43 (s, broad, 1H) ppm. $^{31}P\{^1H\}$ NMR (202.3 MHz, THF-d_8, -40 °C): δ 51.8 (dd, $J^{P\text{-}Rh}$ = 154 Hz, $J^{P\text{-}P}$ = 48 Hz), 14.5 (dd, $J^{P\text{-}Rh}$ = 168 Hz, $J^{P\text{-}P}$ = 48 Hz) ppm.

Rhodium Alkoxo Alkene Complex, **3d**.

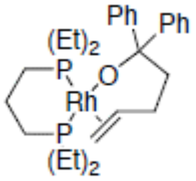

^{1}H NMR (500.2 MHz, toluene-d_8, -40 °C): δ 7.90 (m, broad, 2H), 7.75 (d, broad, J = 7 Hz, 2H), 7.44-7.30 (overlapping signals, 3H), 7.24 (m, 2H), 7.17 (m, broad, 1H), 4.68 (d, broad, J = 13 Hz, 1H), 4.14 (s, broad, 1H), 4.02 (s, broad, 1H), 2.80 (t, J = 8Hz, 1H), 2.70 (t, J=11 Hz, 1H), 2.33 (m, 2H), 2.12 (s, broad, 1H), 1.64-0.56 (overlapping signals, 25H) ppm. $^{13}C\{^1H\}$ NMR (alkenyl carbons, 126 MHz, toluene-d_8, -40 °C): δ 81.02 (m), 68.84 (m) ppm. $^{31}P\{^1H\}$ NMR (202.2 MHz, toluene-d_8, -40 °C): δ 27.13 (dd, $J^{P\text{-}Rh}$ = 145 Hz, $J^{P\text{-}P}$ = 58 Hz), 7.70 (dd, $J^{P\text{-}Rh}$ = 161 Hz, $J^{P\text{-}P}$ = 59 Hz) ppm.

Rhodium Alkoxo Alkene Complex, **3e**.

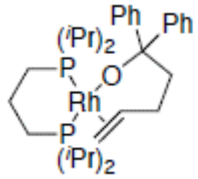

^{1}H NMR (499.4 MHz, toluene-d_8, -40 °C): δ 7.51 (s, broad, 2H), 7.44 (s, broad, 2H), 7.11 (s, broad, 4H), 6.98 (s, broad, 2H), 4.21 (s, broad, 1H), 4.09, (s, broad, overlapping signals, 2H), 3.61 (s, broad, 1H), 2.63 (s, broad, 1H), 2.41(s, broad, 1H), 2.22 (s, broad, 1H), 2.12 (s, broad, 1H) 2.06-

0.80 (overlapping signals, broad, 33H) ppm. $^{13}C\{^1H\}$ NMR (alkenyl carbons, 126 MHz, toluene-d_8, -40 °C): δ 71.83 (m), 66.04 (m) ppm. $^{31}P\{^1H\}$ NMR (202.2 MHz, toluene-d_8, -40 °C): δ 27.50 (dd, J^{P-Rh} = 144 Hz, J^{P-P} = 57 Hz), 7.76 (dd, J^{P-Rh} = 162 Hz, J^{P-P} = 56 Hz) ppm.

Rhodium Alkoxo Alkene Complex, **3f**.

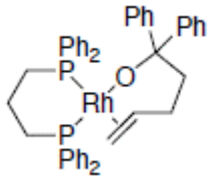

^{1}H NMR (500.2 MHz, toluene-d_8, -40 °C): δ 7.87 (s, broad, 2H), 7.65 (s, broad, 2H), 7.34-6.72 (overlapping signals, broad, 26H), 4.81 (s, broad, 1H), 4.10 (s, broad, 1H), 3.54 (s, broad, 1H), 2.75 (s, broad, 2H), 2.53 (s, broad, 1H), 1.98 (s, broad, 2H), 1.85 (s, broad, 1H), 1.77 (s, broad, 1H), 1.54 (s, broad, 1H), 1.23 (s, broad, 2H) ppm. $^{13}C\{^1H\}$ NMR (alkenyl carbons,126 MHz, THF-d_8, -40 °C): δ 83.06 (m), 70.06 (m) ppm. $^{31}P\{^1H\}$ NMR (202.2 MHz, toluene-d_8, -40 °C): δ 34.94 (dd, J^{P-Rh} = 140 Hz, J^{P-P} = 61 Hz), 0.18 (dd, J^{P-Rh} = 161 Hz, J^{P-P} = 61 Hz) ppm.

Rhodium Alkoxo Alkene Complex, **3g**.

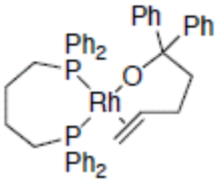

^{1}H NMR (500.2 MHz, toluene-d_8, -40 °C): δ 7.98 (s, broad, 2H), 7.60 (s, broad, 2H), 7.42 (s, broad, overlapping signals, 6H), 7.26-6.76 overlapping signals, broad, 20H), 4.64 (s, broad, 1H), 3.65 (s, broad, 1H), 3.04 (s, broad, 1H), 2.60 (m, broad, 2H), 2.43 (s, broad, 1H), 2.10 (overlapping signals, broad, 1H), 1.97 (s, broad, 2H), 1.90-1.62 (overlapping signals, broad, 5H), 1.33 (s, broad, 1H) ppm. $^{13}C\{^1H\}$ NMR (alkenyl carbons, 126 MHz, THF-d_8, -40 °C): δ 73.79 (m), 63.54 (m) ppm. $^{31}P\{^1H\}$ NMR (202.2 MHz, toluene-d_8, -40 °C): δ 52.28 (s, broad), 1.98 (s, broad) ppm.

Rhodium Alkoxo Alkene Complex, **3h**.

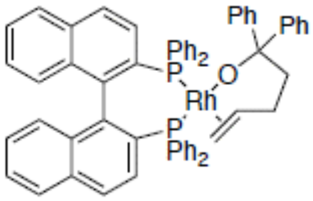

^{1}H NMR (499.4 MHz, THF-d_8, -40 °C): δ 8.00 (s, broad, 1H), 7.79 (s, broad, 2H), 7.73 (s, broad, 1H), 7.62-6.78 (overlapping signals, broad, 30H), 6.46 (s, broad, overlapping signals, 4H), 6.37 (s, broad, overlapping signals, 4H), 4.37 (d, *J* =12 Hz, 1H), 4.14 (s, broad, 1H), 3.08 (s, broad, 1H), 2.94 (s, broad, 1H), 2.74 (s, broad, 1H), 1.91 (s, broad, 1H), 1.42 (s, broad, 1H) ppm. ^{13}C{^{1}H} NMR (alkenyl carbons, 126 MHz, THF-d_8, -40 °C): δ 82.34 (m), 2nd signal overlaps with THF-d_8 ppm. ^{31}P{^{1}H} NMR (202.2 MHz, THF-d_8, -40 °C): δ 54.73 (dd, $J^{P\text{-}Rh}$ = 138 Hz, $J^{P\text{-}P}$ = 60 Hz), 22.12 (dd, $J^{P\text{-}Rh}$ = 165 Hz, $J^{P\text{-}P}$ = 60 Hz) ppm.

Rhodium Alkoxo Alkene Complex, **3i**.

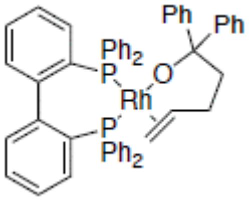

^{1}H NMR (499.4 MHz, THF-d_8, -40 °C) δ 7.83-6.63 (overlapping signals, 38H), 4.14 (d, *J* = 13 Hz, 1H), 3.97 (s, 1H), 2.76 (m, 1H), 2.52 (s, 1H), 2.38 (t, *J* = 11 Hz, 1H), 1.59 (s, 1H), 1.22 (s, 1H) ppm. ^{13}C{^{1}H} NMR (alkenyl carbons, 126 MHz, THF-d_8, -40 °C): δ 80.71 (m), 65.28 (m) ppm. ^{31}P{^{1}H} NMR (202.2 MHz, THF-d_8, -40 °C): δ 50.4 (dd, $J^{P\text{-}Rh}$ = 138 Hz, $J^{P\text{-}P}$ = 61 Hz), 16.46 (dd, $J^{P\text{-}Rh}$ = 162 Hz, $J^{P\text{-}P}$ = 61 Hz) ppm.

Rhodium Alkoxo Alkene Complex, 3**j**.

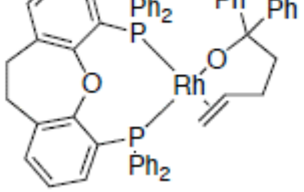

^{1}H NMR (499.4 MHz, toluene-d_8, -40 °C): δ 8.28 (s, broad, 2H), 8.17 (d, *J* = 7 Hz, 2H), 7.69 (d, *J* = 8 Hz, 2H), 7.45 (m, 2H), 7.37 (d, *J* = 8 Hz, 2H), 7.25-6.75 (overlapping signals, m, 22H), 6.68 (m, 1H), 6.49 (d, *J* = 8 Hz, 2H), 6.30 (t, *J* = 8 Hz, 1H),5.16 (d, *J* = 13 Hz, 1H), 3.68 (s, 1H), 3.19 (t, *J* = 12 Hz, 1H), 3.01 (s, broad, 1H), 2.79 (s, broad, overlapping signal, 2H), 2.75 (s, broad,

overlapping signal, 1H), 2.64-2.40 (s, broad, overlapping signal, 1H), 2.47 (m, overlapping signal, 1H), 2.40-2.21 (overlapping signals, m, 2H) ppm. $^{13}C\{^1H\}$ NMR (alkenyl carbons, 126 MHz, THF-d_8, -40 °C): δ 60.67 (m), 59.98 (m) ppm. $^{31}P\{^1H\}$ NMR (202.2 MHz, toluene-d_8, -40 °C): δ 37.0 (dd, $J^{P\text{-}Rh}$ = 155 Hz, $J^{P\text{-}P}$ = 31 Hz), -7.59 (d, broad, $J^{P\text{-}Rh}$ = 155 Hz) ppm.

Rhodium Alkoxo Alkene Complex, **3k**.

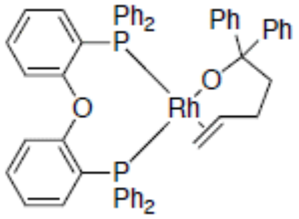

^1H NMR (499.4 MHz, toluene-d_8, -40 °C) δ 8.03 (s, broad, 2H), 7.86 (s, broad, 2H), 7.59 (s, broad, 2H), 7.52 (s, broad, 2H), 7.32 (s, broad, 2H), 7.28-6.83 (overlapping signals, broad, 20H), 6.70 (s, broad, 2H), 6.57 (s, broad, 4H), 6.32 (s broad, 1H), 6.17 (s, broad, 1H), 5.12 (s, broad, 1H), 3.66 (s, broad, 1H), 3.03 (s, broad, 1H), 2.68 (overlapping signals, broad, 3H), 2.24 (s, broad, 1H) ppm. $^{31}P\{^1H\}$ NMR (202.2 MHz, toluene-d_8, -40 °C): δ 35.87 (dd, $J^{P\text{-}Rh}$ = 148 Hz, $J^{P\text{-}P}$ = 32 Hz), -2.76 (dd, $J^{P\text{-}Rh}$ = 162 Hz, $J^{P\text{-}P}$ = 32 Hz) ppm. The complex gradually precipitated from solution at -40 °C. Consequently, a ^{13}C NMR spectrum was not obtained.

Rhodium Alkoxo Alkene Complex, **3l**.

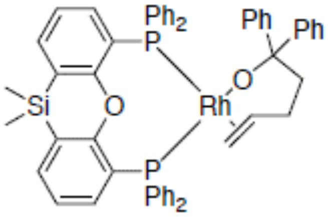

^1H NMR (499.4 MHz, toluene-d_8, -40 °C) δ 8.33 (s, broad, 2H), 7.50-6.27 (broad, overlapping signals, 34H), 4.16 (d, J = 12 Hz, 1H), 2.89 (d, broad, overlapping signal, J = 13 Hz, 2H), 2.84 (s, broad, overlapping signal, 1H), 2.57 (m, broad, overlapping signal, 1H), 2.51 (s, broad, overlapping signal, 1H), 2.21 (t, J = 17 Hz, 1H), 0.38 (s, broad, 3H), 0.31 (s, broad, 3H) ppm. $^{31}P\{^1H\}$ NMR (202.2 MHz, toluene-d_8, -40 °C): δ 39.2 (s, broad), 8.21 (s, broad) ppm. The complex gradually precipitated from solution at -40 °C. Consequently, a ^{13}C NMR spectrum was not obtained.

Rhodium Alkoxo Alkene Complex, **3m**.

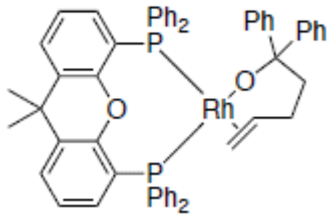

^{1}H NMR (499.4 MHz, toluene-d_8, -40 °C) δ 8.46 (s, broad, 2H), 8.19 (s, broad, 2H), 7.48 (s, broad, 4H), 7.44 (s, broad, 4H), 7.31-6.42 (overlapping signals, 24H), 3.83 (d, broad, J =10 Hz, 1H), 2.80 (overlapping signals, broad, 2H), 1.06 (s, broad, 1H), 2.37 (s, broad, 1H), 2.25-2.00 (overlapping signals, broad, 2H), 1.46 (s, broad, 3H), 1.42 (s, broad, 3H) ppm. ^{31}P{^{1}H} NMR (202.3 MHz, toluene-d_8, -40 °C): δ 55.4 (dd, $J^{P\text{-}Rh}$ = 135 Hz, $J^{P\text{-}P}$ = 55 Hz), 22.9 (dd, broad, $J^{P\text{-}Rh}$ = 158 Hz, $J^{P\text{-}P}$ = 57 Hz) ppm. The complex gradually precipitated from solution at -40 °C. Consequently, a ^{13}C NMR spectrum was not obtained.

Rhodium Alkoxo Alkene Complex, **5f**. (Z/E = 37:63)

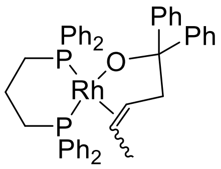

^{1}H NMR (499.4 MHz, toluene-d8) δ 8.05 (s, broad, 2H), 7.73 (s, broad, 4H), 7.48 (s, broad, 4H), 7.64 (s, broad, 4H), 7.55 (s, broad, 2H), 7.42 (s, broad, 2H), 7.28-6.78 (overlapping signals, 42H), 5.46 (m, broad, 1H), 4.53 (m, broad, 1H), 3.93 (m, broad, 1H), 3.73 (d, broad, J =16 Hz, 1H), 3.39 (m, 2H), 3.21 (m, broad, 1H), 2.29 (m, broad, 1H), 2.05-1.83 (m, 12H), 1.79 (d, broad, J =7 Hz, 3H), 0.99 (d, broad, J =6 Hz, 3H) ^{31}P{^{1}H} NMR (202.3 MHz, toluene-d8): δ 32.4 (dd, JP-Rh = 138 Hz, JP-P = 69 Hz), 6.2 (dd, broad, JP-Rh = 166 Hz, JP-P = 69 Hz) ppm.

Independent Synthesis of 2.2-Diphenyl-5-Methylene-Tetrahydrofuran (**4a**).

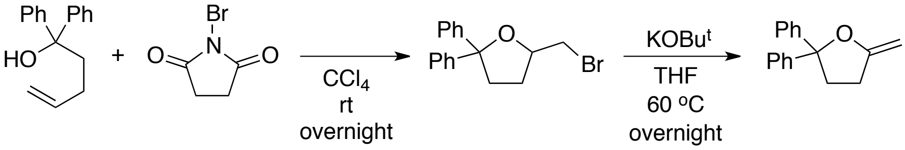

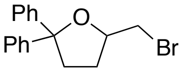

2,2-Diphenyl-5-methylene-tetrahydrofuan was synthesized according to the procedures reported by Hartwig and co-workers.[8] A 20 ml vial, equipped with a magnetic stir bar, was charged with 1,1-diphenyl-4-penten-1-ol (1 g, 4.2 mmol, 1.0 equiv), CCl_4 (10 ml), and *N*-bromosuccinimide (820 mg, 4.6 mmol, 1.1 equiv). The vial was capped and the resulting solution was stirred overnight at room temperature. Pentane was added to the product solution and the solution was filtered through Celite. The filtrate was collected. NaOAc was added to the filtrate and the solvent was removed under vacuum. The resulting 2,2-diphenyl-5-bromomethyltetrahydrofuan was used without further purification. Yield: 1.1 g, 3.4 mmol, 81 %.

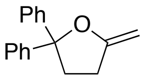

The crude 2,2-diphenyl-5-bromomethyltetrahydrofuan (1.1 g, 3.4 mmol, 1.0 equiv) was transferred to a 20 ml vial inside a glovebox. The vial was equipped with a magnetic stir bar and then charged with dry THF (10 ml) and KO^{t}Bu (0.38 g, 3.4 mmol, 1 equiv). The vial was capped and removed from the glovebox. The reaction was heated to 60 °C overnight. The product solution was cooled to room temperature and concentrated under vacuum. The residual solid was extracted with pentane and filtered through Celite. The filtrate was collected and concentrated under vacuum. The crude product was purified by column chromatography. A white solid was obtained. Yield: 0.44 g, 1.9 mmol, 55 %. The product slowly decomposed at room temperature over the course of a year. ^{1}H NMR (499.7 MHz, C_6D_6): δ 7.40 (d, $J^{H\text{-}H}$ = 7 Hz, 4H), 7.08 (t, $J^{H\text{-}H}$ = 7 Hz, 4H), 6.99 (d, $J^{H\text{-}H}$ = 7 Hz, 2H), 4.72 (s, 1H), 3.98 (s, 1H), 2.23 (s, 4H) ppm. ^{13}C{^{1}H} NMR (125.6 MHz, $CDCl_3$): δ 162.13, 144.89, 128.44, 127.35, 125.97, 90.83, 80.25, 38.00, 29.10 ppm.

Independent Synthesis of 5-Methyl-2,2-Diphenyl-2,3-Dihydrofuran (**4b**).

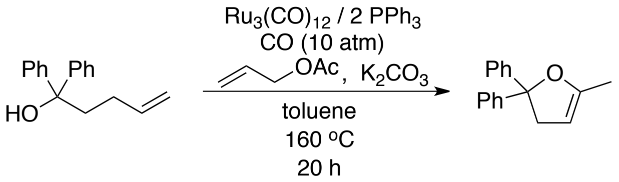

5-Methyl-2,2-diphenyl-2,3-dihydrofuran was synthesized according to the procedures reported by Mitsudo and co-workers.[9] A steel Parr bomb, equipped with a magnetic stir bar, was charged with $Ru_3(CO)_{12}$ (45 mg, 0.070 mmol, 0.017 equiv), PPh_3 (110 mg, 0.42 mmol, 0.10 equiv), allyl acetate (3.4 ml, 3.2 g, 31 mmol, 7.5 equiv), K_2CO_3 (1.4 g, 10. mmol, 2.5 equiv), 1,1-diphenyl-4-penten-1-ol (1.0 g, 4.2 mmol, 1.0 equiv), and toluene (20 ml) inside a nitrogen-filled glovebox. The bomb was sealed and removed from the box. The bomb was then charged with CO (10 atm) and heated to 160 °C for 20 h. The bomb was opened, and the product solution washed with brine and purified by column chromatography. Yield: 640 mg, 2.7 mmol, 65 %. ^{1}H NMR (499.7 MHz, $CDCl_3$): δ 7.52 (d, $J^{H\text{-}H}$ = 8 Hz, 4H), 7.39 (t, $J^{H\text{-}H}$ = 8 Hz, 4H), 7.31 (d, $J^{H\text{-}H}$ = 8 Hz, 2H), 4.66 (s, 1H), 3.41 (s, 2H), 2.02 (s, 3H) ppm. ^{13}C{^{1}H} NMR (125.6 MHz, $CDCl_3$): δ 153.37, 146.54,128.08, 126.95, 125.75, 93.92, 90.66, 45.00, 13.76 ppm.

Independent Synthesis of 5-Methyl-2,2-Diphenyl-2,5-Dihydrofuran (**4c**).

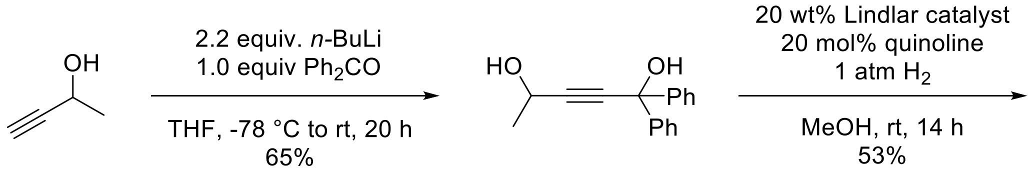

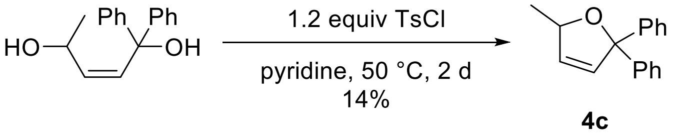

4c

To a solution of but-3-yn-2-ol (550 µL, 7.0 mmol, 1.00 equiv) in THF (17 mL) in a 100-mL round-bottom flask *n*-butyllithium (2.5 M in hexane, 6.2 mL, 15 mmol, 2.2 equiv) was added dropwise at -78 °C. The reaction mixture was stirred for 1 h at -78 °C. Then, a solution of benzophenone (1.3 g, 7.0 mmol, 1.0 equiv) in THF (17 mL) was added, and the reaction mixture was allowed to warm to ambient temperature over a course of 20 h. A saturated aqueous solution of ammonium chloride was added, and the aqueous layer was extracted three times with ethyl acetate. The combined organic layers were dried with magnesium sulfate, and the volatile materials were evaporated under reduced pressure. The crude product was purified by column chromatography (30% ethyl acetate in hexanes) to yield 1,1-diphenylpent-2-yne-1,4-diol as a white solid (1.2 g, 65%).

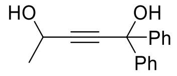

^{1}H NMR (500 MHz, $CDCl_3$): δ 7.58 (d, $J_{H\text{-}H}$ = 7.1 Hz, 4H), 7.32 (t, $J_{H\text{-}H}$ = 7.5 Hz, 4H), 7.26 (t, $J_{H\text{-}H}$ = 7.3 Hz, 2H), 4.66 (dq, $J_{H\text{-}H}$ = 5.2 Hz, $J_{H\text{-}H}$ = 6.6 Hz, 1H), 2.95 (s, 1H), 2.07 (d, $J_{H\text{-}H}$ = 5.3 Hz, 1H), 1.51 (d, $J_{H\text{-}H}$ = 6.6 Hz, 3H) ppm. ^{13}C{^{1}H} NMR (126 MHz, $CDCl_3$): δ 144.9, 128.4, 127.9, 126.1, 89.2, 86.8, 74.4, 58.7, 24.3 ppm.

These data are consistent with previously reported values.[10]

A 50-mL round-bottom flask was charged with 1,1-diphenylpent-2-yne-1,4-diol (397 mg, 1.57 mmol, 1.00 equiv), Lindlar catalyst (79 mg, 20 wt%), quinoline (37 μL, 0.31 mmol, 0.20 equiv), and methanol (5 mL). The reaction mixture was stirred under hydrogen (1 atm) at ambient temperature for 14 h. It was then filtered through a pad of Celite, and the filtrate was collected. The volatile materials were evaporated under reduced pressure, and the crude product was purified by column chromatography (20% to 30% ethyl acetate in hexanes) to yield (*Z*)-1,1-diphenylpent-2-ene-1,4-diol as a colorless oil (211 mg, 53%).

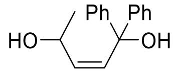

^{1}H NMR (500 MHz, $CDCl_3$): δ 7.47 – 7.41 (m, 4H), 7.35 – 7.30 (m, 4H), 7.29 – 7.24 (m, 2H), 6.20 (dd, $J_{H\text{-}H}$ = 12.1 Hz, $J_{H\text{-}H}$ = 1.5 Hz, 1H), 5.66 (dd, $J_{H\text{-}H}$ = 12.1 Hz, $J_{H\text{-}H}$ = 6.6 Hz, 1H), 4.68 (dp, $J_{H\text{-}H}$ = 1.5 Hz, $J_{H\text{-}H}$ = 6.4 Hz, 1H), 4.56 (br, 1H), 2.78 (br, 1H), 1.23 (d, $J_{H\text{-}H}$ = 6.4 Hz, 3H) ppm. ^{13}C{^{1}H} NMR (126 MHz, $CDCl_3$): δ 148.0 147.1, 137.9, 134.9, 128.3, 128.3, 127.2, 126.6, 126.5, 79.3, 64.5, 23.4 ppm. HRMS (EI), calculated for $C_{17}H_{16}O$ (M-H_2O): 236.1201, found: 236.1201.

A 50-mL round-bottom flask was charged with (*Z*)-1,1-diphenylpent-2-ene-1,4-diol (95 mg, 0.37 mmol, 1.0 equiv), tosyl chloride (85 mg, 0.45 mmol, 1.2 equiv), and pyridine (4 mL). The reaction mixture was heated at 50 °C for 2 days. Water was added, and the aqueous layer was extracted three times with ethyl acetate. The combined organic layers were dried with magnesium sulfate, and the volatile materials were evaporated under reduced pressure. The crude product was purified by column chromatography (4% ethyl acetate in hexanes) to yield 5-methyl-2,2-diphenyl-2,5-dihydrofuran (**4c**) as a white solid (12 mg, 14%).

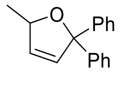

^{1}H NMR (500 MHz, $CDCl_3$): δ 7.39 – 7.28 (m, 8H), 7.29 – 7.21 (m, 2H), 6.27 (dd, $J_{H\text{-}H}$ = 5.9 Hz, $J_{H\text{-}H}$ = 2.3 Hz, 1H), 5.95 (dd, $J_{H\text{-}H}$ = 5.9 Hz, $J_{H\text{-}H}$ = 1.4 Hz, 1H), 5.43 – 4.88 (m, 1H), 1.39 (d, $J_{H\text{-}H}$ =

6.5 Hz, 3H) ppm. $^{13}C\{^1H\}$ NMR (126 MHz, $CDCl_3$): δ 146.1, 145.5, 132.6, 131.3, 128.3, 128.2, 127.2, 127.1, 126.7, 126.5, 94.6, 82.1, 21.9 ppm. HRMS (EI), calculated for $C_{17}H_{16}O$ (M): 236.1201, found: 236.1201.

Synthesis of ^{13}C-labelled alcohol **2** (**2-^{13}C**).

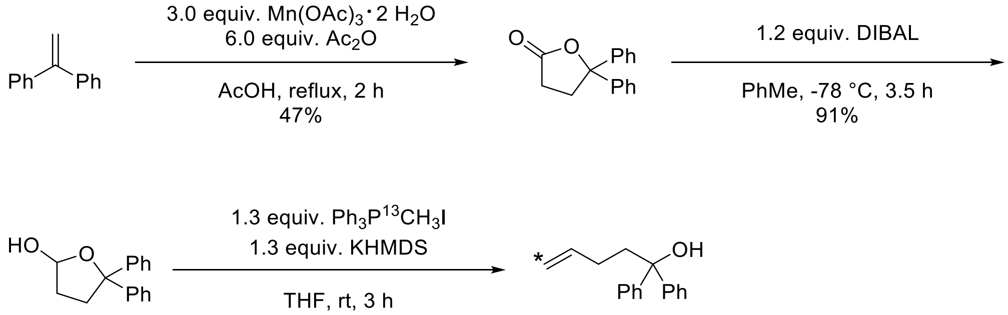

2-^{13}C

A 50-mL round-bottom flask was charged with 1,1-diphenylethene (368 mg, 2.04 mmol, 1.00 equiv), manganese(III) acetate dihydrate (1.6 g, 6.0 mmol, 3.0 equiv), acetic anhydride (1.1 mL, 12.0 mmol, 6.0 equiv), and acetic acid (30 mL). The reaction mixture was heated under reflux for 2 h. After cooling to room temperature, the volatile materials were evaporated under reduced pressure, and saturated sodium thiosulfate solution was added to the residue. The aqueous layer was extracted three times with ethyl acetate, and the combined organic layers were dried with magnesium sulfate. The solvent was removed under reduced pressure, and the crude product was purified by column chromatography (0% to 20% ethyl acetate in hexanes) to give 5,5-diphenyldihydrofuran-2(3*H*)-one as a yellow solid (225 mg, 47%).

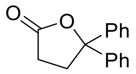

^1H NMR (600 MHz, $CDCl_3$): δ 7.43 (d, J_{HH} = 7.3 Hz, 4H), 7.35 (t, J_{HH} = 7.7 Hz, 4H), 7.28 (t, J_{HH} = 7.3 Hz, 2H), 2.91 (t, J_{HH} = 7.8 Hz, 2H), 2.57 (t, J_{HH} = 7.8 Hz, 2H) ppm. $^{13}C\{^1H\}$ NMR (151 MHz, $CDCl_3$): δ 176.2, 143.1, 128.7, 128.0, 125.4, 89.8, 35.7, 29.1 ppm.

These data are consistent with previously reported values.[11]

To a solution of diphenyldihydrofuran-2(3*H*)-one (140 mg, 0.59 mmol, 1.0 equiv) in toluene (2 mL) in a 50-mL round-bottom flask was added dropwise DIBAL (25 wt% in toluene, 0.69 mL, 0.70 mmol, 1.2 equiv) at -78 °C. The reaction mixture was stirred at -78 °C for 3.5 h. Then, methanol was added dropwise, and the reaction was allowed to warm to ambient temperature slowly. Saturated potassium sodium tartrate solution was added. The aqueous layer was extracted three times with ethyl acetate, and the combined organic layers were dried with magnesium sulfate. The volatile materials were evaporated under reduced pressure, and the crude product was

purified by column chromatography (17% ethyl acetate in hexanes) to yield 5,5-diphenyltetrahydrofuran-2-ol as a white solid (128 mg, 91%).

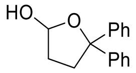

^{1}H NMR (500 MHz, $CDCl_3$): δ 7.47 (d, J_{HH} = 7.7 Hz, 2H), 7.41 (d, J_{HH} = 7.2 Hz, 2H), 7.31 (t, J_{HH} = 7.6 Hz, 4H), 7.22 (t, J_{HH} = 7.3 Hz, 2H), 5.74 (d, J_{HH} = 3.0 Hz, 1H), 3.09 (s, 1H), 2.94 – 2.47 (m, 2H), 2.22 – 1.81 (m, 2H). ppm. ^{13}C{^{1}H} NMR (126 MHz, $CDCl_3$): δ 146.8, 146.1, 128.2, 128.2, 127.0, 126.9, 126.3, 125.8, 99.0, 89.8, 36.8, 33.6 ppm.

These data are consistent with previously reported values.[12]

To a solution of triphenylphosphine (334 mg, 1.28 mmol, 1.0 equiv) in toluene (3.2 mL) in a 25-mL round-bottom flask was added ^{13}C-methyl iodide (87 µL, 1.4 mmol, 1.1 equiv). The reaction mixture was stirred for 18 h at ambient temperature. The precipitate was separated by filtration, washed with hexanes, and dried to yield ^{13}C-methyltriphenylphosphonium iodide as a white solid (433 mg, 84%). The product was used without characterization.

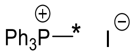

To a suspension of ^{13}C-methyltriphenylphosphonium iodide (210 mg, 0.51 mmol, 1.3 equiv) in THF (0.6 mL) in a 4-mL vial was added a solution of KHMDS (103 mg, 0.51 mmol, 1.32 equiv) in THF (0.4 mL), and the reaction mixture was stirred at ambient temperature for 1 h. Then, a solution of 5,5-diphenyltetrahydrofuran-2-ol (94 mg, 0.39 mmol, 1.0 equiv) in THF (0.5 mL) was added, and the reaction mixture was stirred for an additional 3 h. Subsequently, water was added, and the aqueous layer was extracted three times with ethyl acetate. The combined organic layers were dried with magnesium sulfate, and the solvent was removed under reduced pressure. The crude product was purified by column chromatography to yield 5-^{13}C-1,1-diphenylpent-4-en-1-ol (**^{13}C-2**) as a colorless oil (77 mg, 82%).

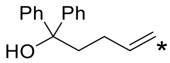

^{1}H NMR (400 MHz, $CDCl_3$): δ 7.42 (d, J_{HH} = 7.1 Hz, 4H), 7.32 (t, J_{HH} = 7.6 Hz, 4H), 7.23 (t, J_{HH} = 7.3 Hz, 2H), 5.86 (ddt, J_{HH} = 16.8 Hz, J_{HH} = 10.1 Hz, J_{HH} = 6.5 Hz, 1H), 5.28 – 5.09 (m, 1H), 4.91 – 4.70 (m, 1H), 2.45 – 2.36 (m, 2H) 2.17 (br, 1H), 2.13 – 2.00 (m, 2H) ppm. ^{13}C{^{1}H} NMR (100 MHz, $CDCl_3$, only labelled carbon shown): δ 114.9 ppm. HRMS (EI), calculated for [$C_{16}{}^{13}CH_{16}$ (M-H_2O)]: 221.1286, found: 221.1286.

Synthesis of 1,1-bis(4-fluorophenyl)pent-4-en-1-ol (**S1**).

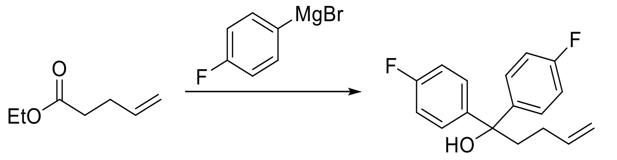

To a solution of ethyl pent-4-enoate (112 g, 0.87 mmol, 1.0 equiv) in THF (2.5 mL) in a 50-mL round-bottom flask was added 4-flourophenylmagnesium bromide (1.0 M in THF, 3.5 mL, 3.5 mmol, 4.0 equiv) at 0 °C. The reaction mixture was stirred at ambient temperature for 17 h. Then, a saturated solution of ammonium chloride was added, and the aqueous layer was extracted three times with ethyl acetate. The combined organic layers were dried with magnesium sulfate, and the solvent was removed under reduced pressure. The resulting crude product was purified by column chromatography (5% ethyl acetate in hexanes) to yield 1,1-bis(4-fluorophenyl)pent-4-en-1-ol (**S1**) as a colorless oil (174 mg, 73%).

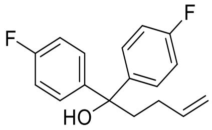

^{1}H NMR (500 MHz, $CDCl_3$): δ 7.39 – 7.34 (m, 4H), 7.02 – 6.97 (m, 4H), 5.85 (ddt, J_{HH} = 16.9 Hz, J_{HH} = 10.2 Hz, J_{HH} = 6.6 Hz, 1H), , 5.03 – 4.96 (m, 2H), 2.36 – 2.33 (m, 2H), 2.15 (s, 1H), 2.07 – 2.02 (m, 2H) ppm. ^{13}C{^{1}H} NMR (126 MHz, $CDCl_3$): δ 161.9 (J_{CF} = 246.0 Hz), 142.7 (J_{CF} = 3.2 Hz), 138.5, 127.9 (J_{CF} = 8.0 Hz), 115.2 (J_{CF} = 21.4 Hz) 115.2, 77.8, 41.3, 28.4 ppm. ^{19}F{^{1}H} NMR (470 MHz, $CDCl_3$): δ -115.87 ppm. HRMS (EI), calculated for [$C_{17}H_{14}F_2$ (M-H_2O)]: 256.1064, found: 256.1061.

Synthesis of 1,1-bis(phenyl-*d*$_5$)pent-4-en-1-ol (**2-*d*$_{10}$**).

To a suspension of magnesium (91 mg, 3.8 mmol, 4.8 equiv) in THF (2 mL) in a 100-mL round-bottom flask was added 1,2-dibromoethane (0.1 mL). The mixture was stirred for 10 minutes. Then, a solution of bromobenzene-*d*$_5$ (506 mg, 3.12 mmol, 4.0 equiv) in THF (1 mL) was added dropwise over 5 minutes. The reaction mixture was heated to reflux for 10 minutes before magnesium (91 mg, 3.75 mmol, 4.8 equiv) was added. The reaction mixture was heated at reflux for another 2 h. After cooling, a solution of ethyl pent-4-enoate (100 mg, 0.78 mmol, 1.0 equiv) in THF (1 mL) was added dropwise at 0 °C. The reaction mixture was stirred at ambient temperature for 16 h. A saturated solution of ammonium chloride was added, and the aqueous layer was extracted three times with ethyl acetate. The combined organic layers were dried with magnesium sulfate, and the volatile materials were evaporated under reduced pressure. The crude product was purified by column chromatography (6% ethyl acetate in hexanes) to yield 1,1-bis(phenyl-*d*$_5$)pent-4-en-1-ol (**2-*d*$_{10}$**) as a colorless oil that crystallized within a week (178 mg, 92%).

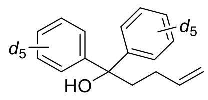

^{1}H NMR (500 MHz, $CDCl_3$): δ 5.87 (ddt, J_{HH} = 16.9 Hz, J_{HH} = 10.2 Hz, J_{HH} = 6.6 Hz, 1H), 5.02 (dq, J_{HH} = 17.1 Hz, J_{HH} = 1.7 Hz, 1H), 4.96 (dq, J_{HH} = 10.2 Hz, J_{HH} = 1.5 Hz, 1H), 2.42 – 2.37 (m, 2H), 2.18 (s, 1H), 2.13 – 2.04 ppm. ^{2}H NMR (77 MHz, toluene): δ 7.44, 7.23, 7.14 ppm. ^{13}C{^{1}H} NMR (151 MHz, $CDCl_3$): δ 146.9, 138.8, 127.8 (t, J_{CD} = 24.3 Hz), 126.5 (t, J_{CD} = 24.2 Hz), 125.7 (t, J_{CD} = 24.1 Hz), 114.9, 78.4, 41.2, 28.5 ppm. HRMS (EI), calculated for [$C_{17}H_6D_{10}$ (M-H_2O)]: 230.1880, found: 230.1877.

Kinetic Data of Alkoxo Alkene Complexes

Figure S1. Plot of the decay of complex **3f** with 1 equiv of PPh_3 at 35ºC.

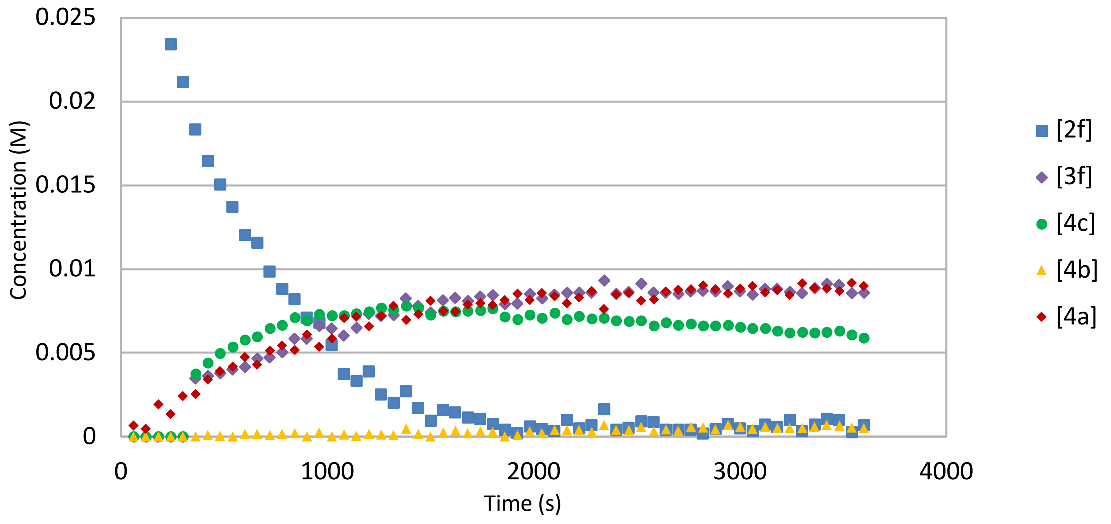

Table S1. Initial rates of reaction of **3f** with varied phosphine concentrations at 25 ºC.

**Entry.**	**Equiv PPh_3**	**Initial rate (mol/s)**
**1**	0.2	$3.0x10^{-4}$
**2**	0.4	$3.6x10^{-4}$
**3**	0.5	$2.9x10^{-4}$
**4**	0.6	$3.0x10^{-4}$
**4**	0.8	$3.0x10^{-4}$
**5**	1	$3.6x10^{-4}$
**6**	10	$3.4x10^{-4}$
**7**	20	$3.2x10^{-4}$

Figure S2. Plot of the decay of alkoxo alkene complex 3**f** with less than 1 equiv of added PPh_3.

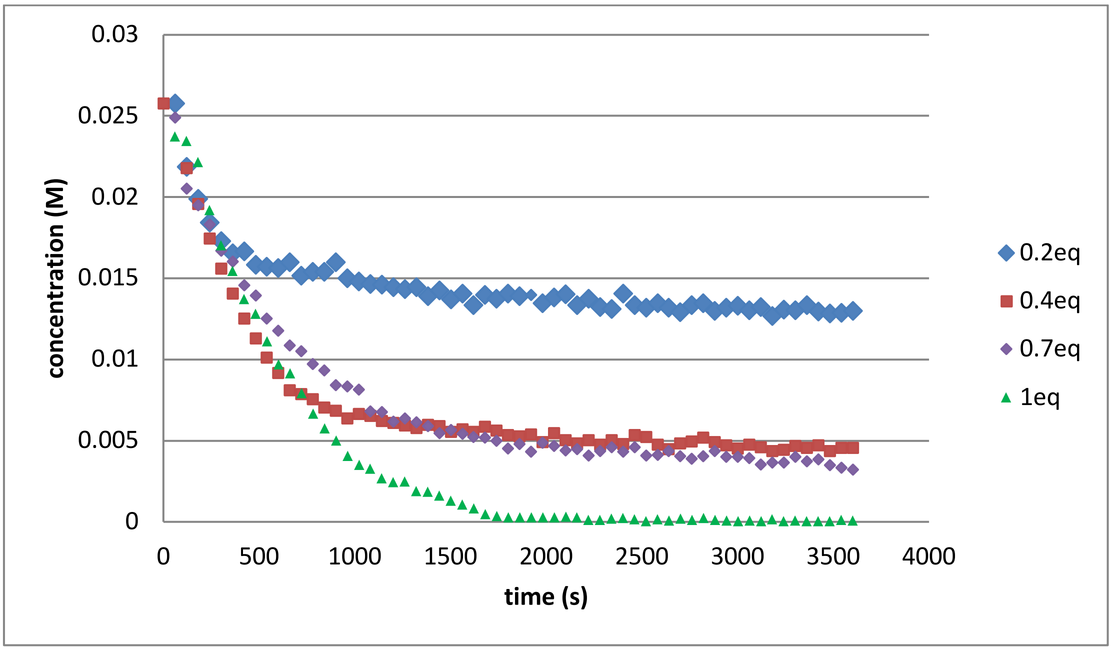

Table S2. Product Selectivity for reactions of **3f** with varying amounts of added PPh_3.

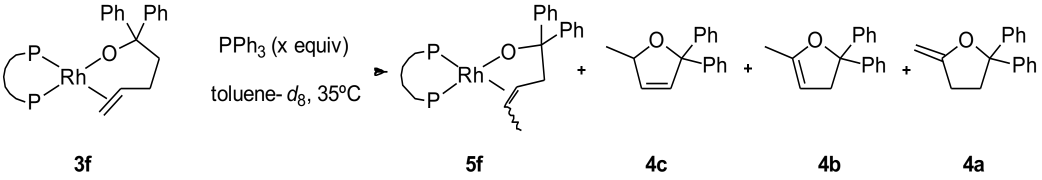

entry	equiv of PPh_3	k_{obs} (s^{-1})	5f (%)[a]	4c (%)[a]	4b(%)[a]	4a (%)[a]	Conv (%)
**1**[b]	0.0	-	42	0	42	13	99
**2**	0.1	-	1	14	3	11	39
**3**	0.2	-	1	14	5	23	50
**4**	0.4	-	24	9	4	41	87
**5**	0.5	-	12	22	6	42	82
**6**	0.7	-	18	15	8	44	90
**7**	1	1.00×10^{-3}	33	23	2	35	97
**8**	5	0.86×10^{-3}	24	14	0	56	99
**9**	10	1.50×10^{-3}	17	16	0	67	99
**10**	20	0.80×10^{-4}	11	18	0	71	99

Results in 60 min reaction. a) NMR yields calculated using TMB as an internal standard. b) Reaction conducted at 70 °C.

Decreasing the amount of added PPh_3 led to lower yields of the main product **4a** and higher yields of isomerized products (**4b-c**, **5f**).

Table S3. Product Selectivity for reactions of **3f** in the presence of a series of added ligands.

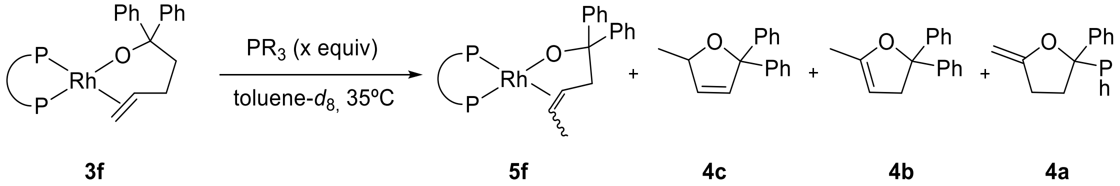

entry	R	equiv of PR_3	5f (%)[a]	4c (%)[a]	4b(%)[a]	4a (%)[a]	Conv (%)
**1**	*p*-OMe-C_6H_4	1	0	46	1	37	87
**2**	*p*-OMe-C_6H_4	5	1	33	2	62	98
**3**	Ph	1	33	23	2	35	97
**4**	Ph	5	23	14	0	57	>99
**5**	*p*-CF_3-C_6H_4	1	41	18	0	35	>99
**6**	*p*-CF_3-C_6H_4	5	22	10	0	58	>99
**7**	Et	1	0	36	7	50	>99
**8**	Et	5	0	12	9	76	>99
**9**	Cy	2	35	0	0	31	95
**10**	Cy	5	0	0	0	54	95
**11**	OPh	1	0	12	0	63	>99
**12**	OPh	2	7	3	0	70	>99

Results are for reactions after 60 min. a) NMR yields calculated using TMB as an internal standard.

Alkene dissociation occurred in the presence of added PCy_3 (2 equiv: 21% dissociation; 5 equiv: 42%) and $P(OPh)_3$ (1 equiv: 0%; 2 equiv: 7%). While the reactions with added PEt_3 formed **4a** in higher yield than that with PPh_3, kinetic studies were conducted with PPh_3 as added ligand because PEt_3 led to displacement of the ancillary ligand in complexes containing less strongly binding ancillary ligands. Such a behavior was not observed with added PPh_3.

Table S4. Observed rate constants, k_{obs}, for the reactions of alkoxo alkene complexes in the presence of varying equiv of added PPh_3.

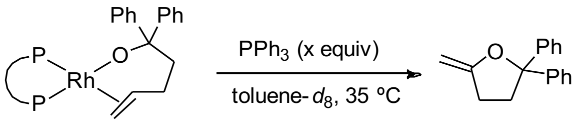

3b, 3e-g

entry	equiv of PPh_3	3b	3e	3f	3g
**1**	0	3.8×10^{-4} s^{-1}	[a]8.8×10^{-4} s^{-1}	[a]11.0×10^{-4} s^{-1}	[a]53.0×10^{-4} s^{-1}
**2**	1	3.9×10^{-4} s^{-1}	0.6×10^{-4} s^{-1}	10.0×10^{-4} s^{-1}	14.0×10^{-4} s^{-1}
**3**	2	-	0.7×10^{-4} s^{-1}	8.6×10^{-4} s^{-1}	12.0×10^{-4} s^{-1}
**5**	10	4.2×10^{-4} s^{-1}	1.4×10^{-4} s^{-1}	7.7×10^{-4} s^{-1}	14.0×10^{-4} s^{-1}
**6**	20	4.2×10^{-4} s^{-1}	2.0×10^{-4} s^{-1}	8.4×10^{-4} s^{-1}	13.0×10^{-4} s^{-1}

Toluene-*d*$_8$ was used as solvent. a) Reactions performed at 70 ºC. At 35 ºC the reactions were too slow to measure.

Figure S3. Plot of the decay of **3f** in the absence of PPh_3 at 35 ºC.

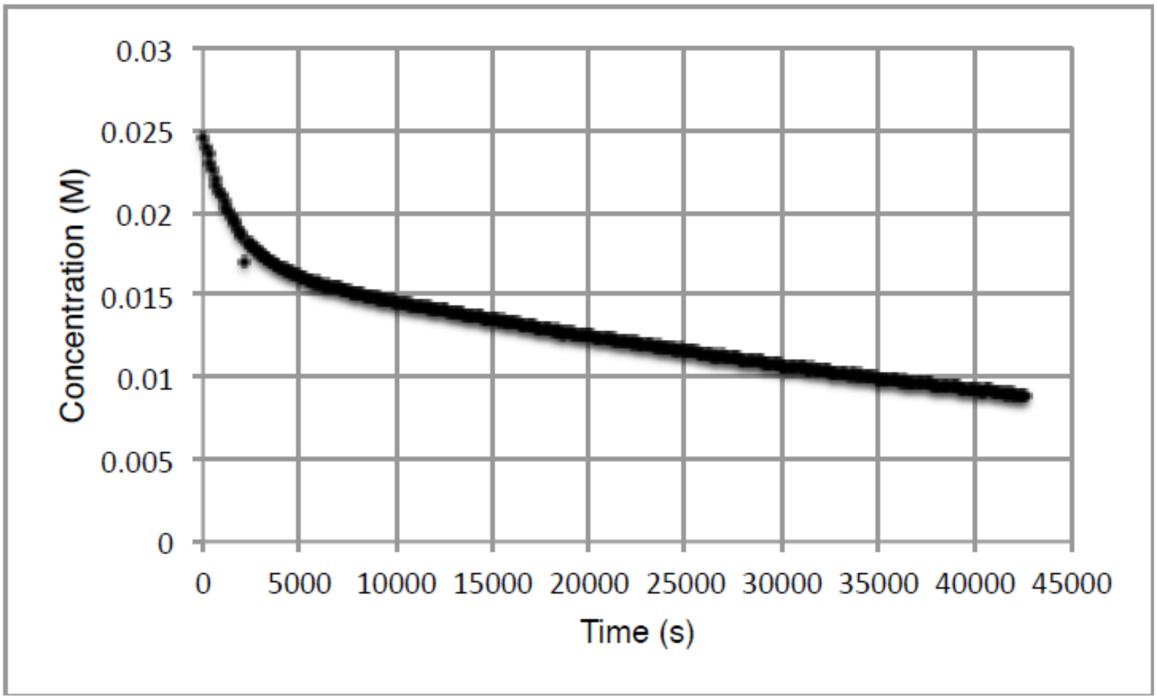

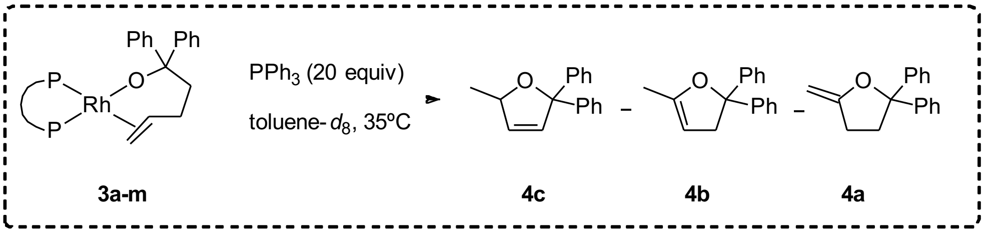

Figure S4. Plot of the decay of **3a** and formation of furans **4a+4b+4c** in the presence of 20 equiv of added PPh_3 at 35 ºC.

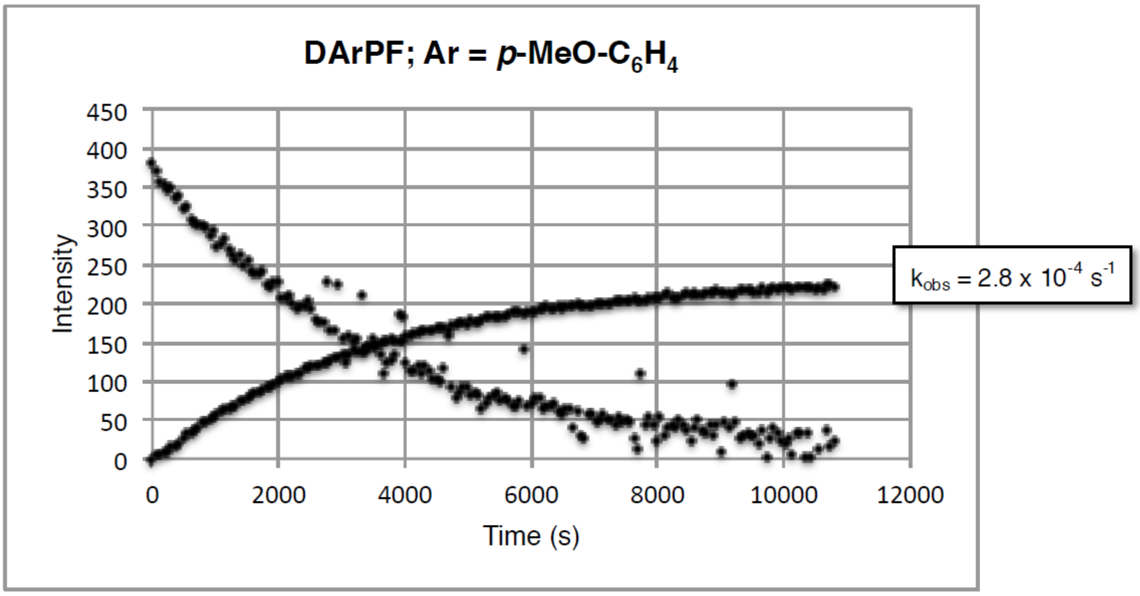

Figure S5. Plot of the decay of **3b** and formation of furans **4a+4b+4c** in the presence of 20 equiv of added PPh_3 at 35 ºC.

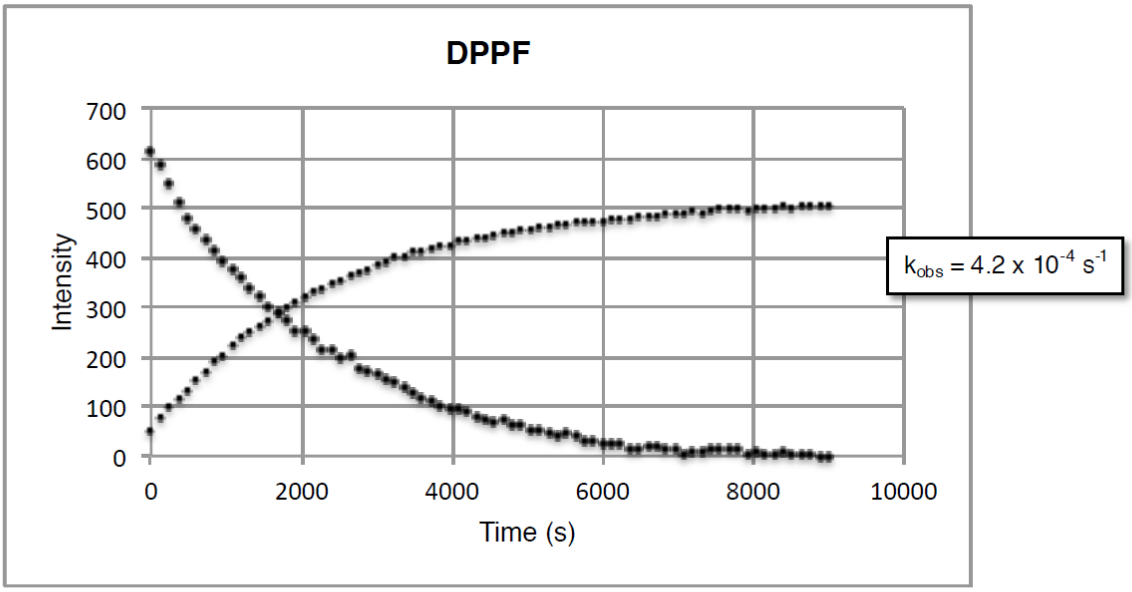

Figure S6. Plot of the decay of **3d** and formation of furans **4a+4b+4c** in the presence of 20 equiv of added PPh_3 at 35 °C.

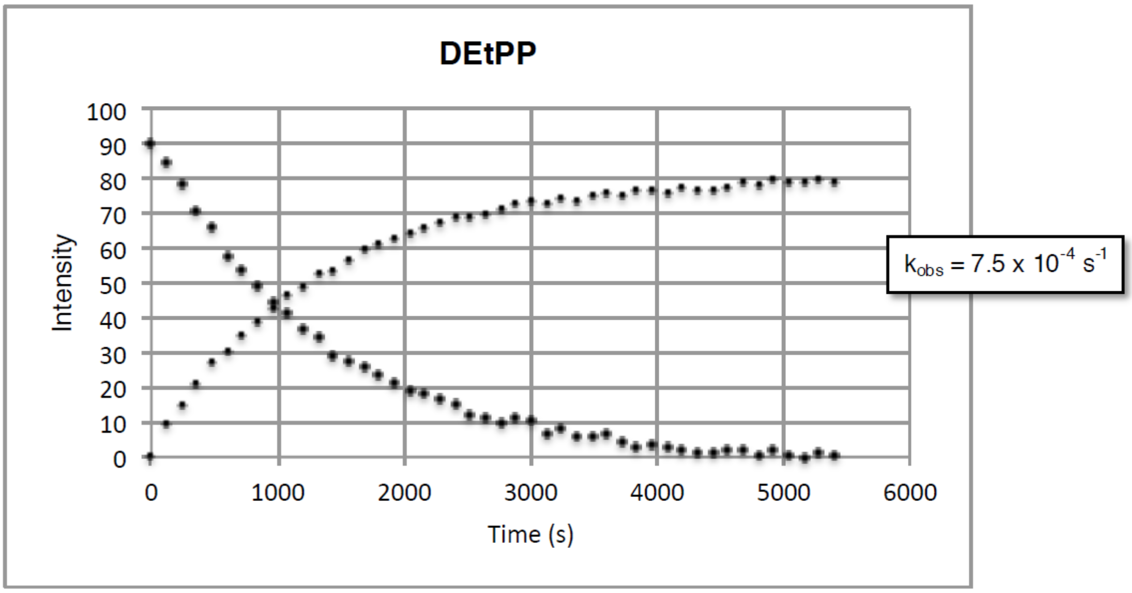

Figure S7. Plot of the decay of **3e** and formation of furans **4a+4b+4c** in the presence of 20 equiv of added PPh_3 at 35 °C.

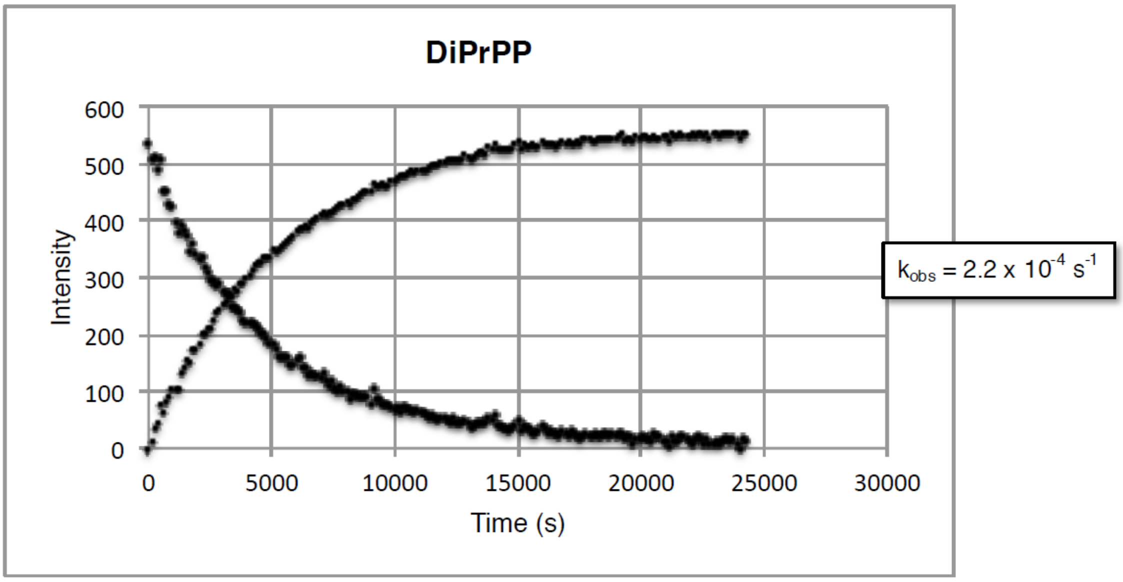

Figure S8. Plot of the decay of **3f** and formation of furans **4a+4b+4c** in the presence of 20 equiv of added PPh_3 at 35 ºC.

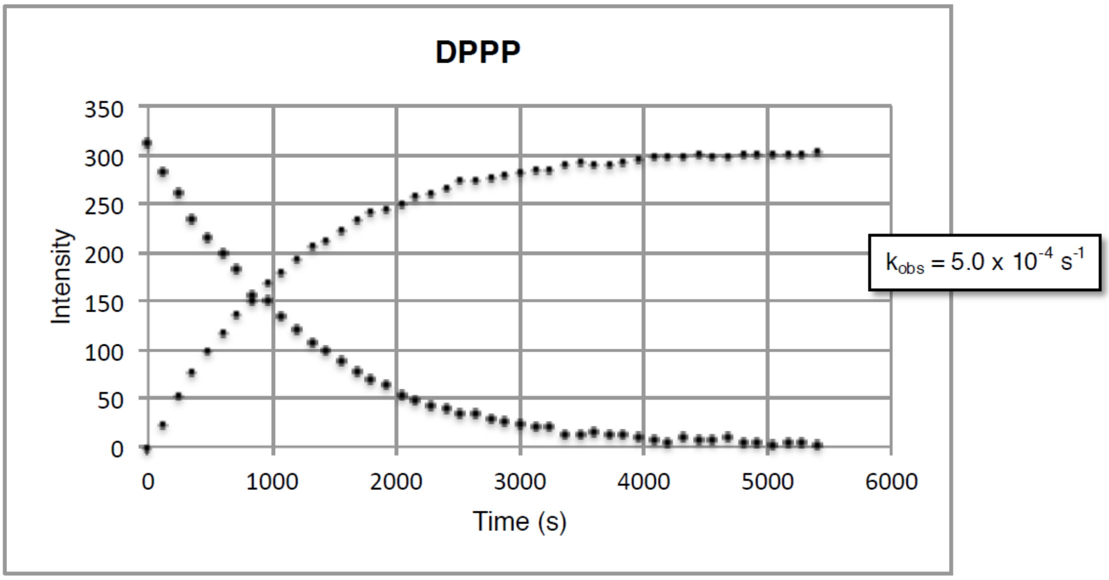

Figure S9. Plot of the decay of **3g** and formation of furans **4a+4b+4c** in the presence of 20 equiv PPh_3 at 35 ºC.

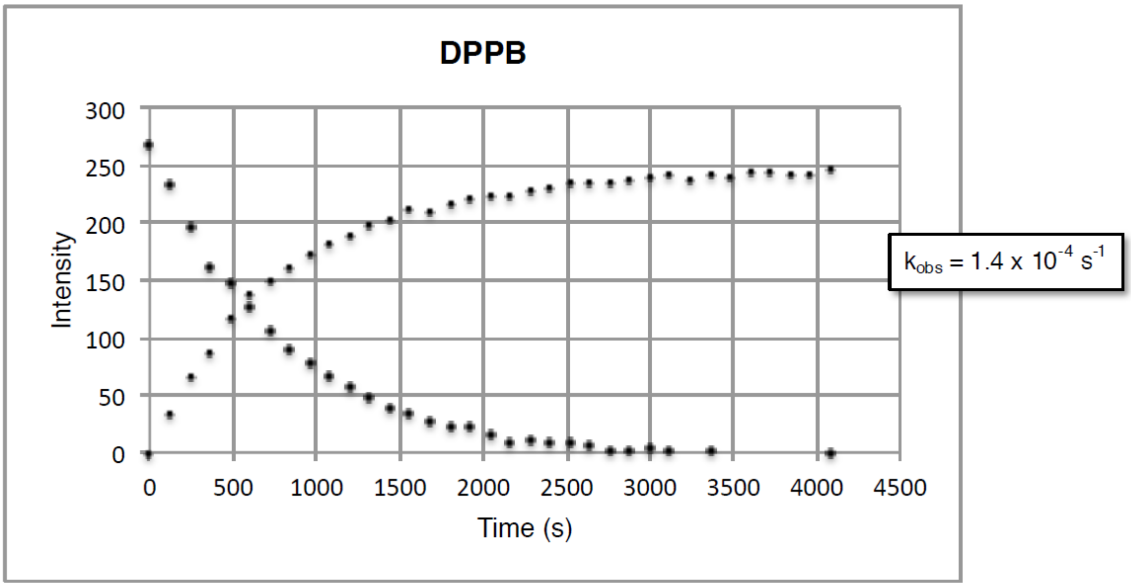

Figure S10. Plot of the decay of **3h** and formation of furans **4a+4b+4c** in the presence of 20 equiv PPh_3 at 35 ºC.

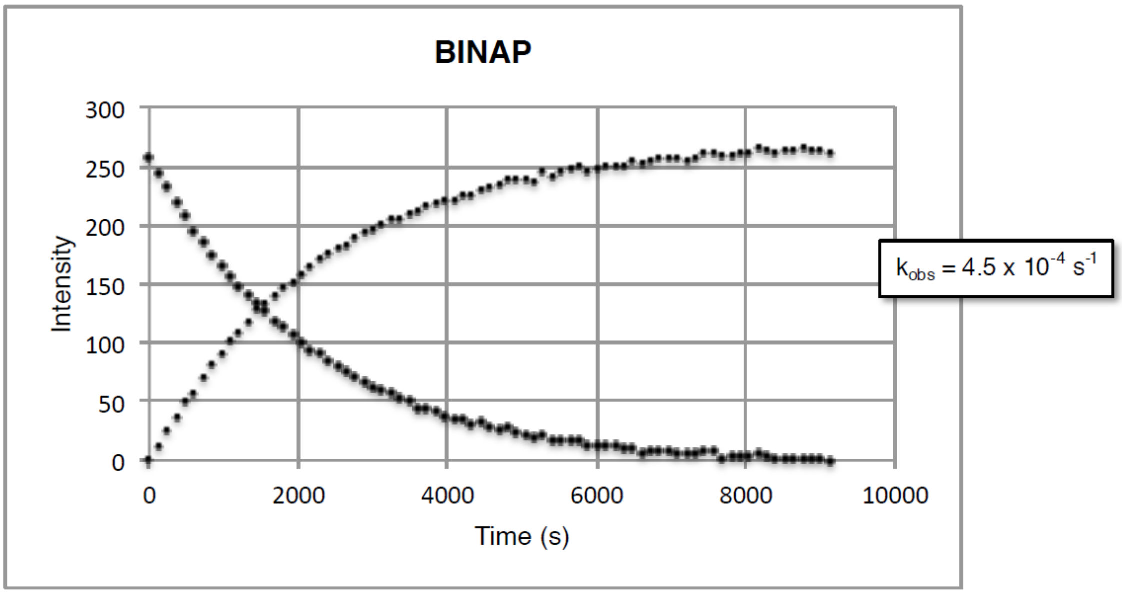

Figure S11. Plot of the decay of **3i** and formation of furans **4a+4b+4c** in the presence of 20 equiv PPh_3 at 35 ºC.

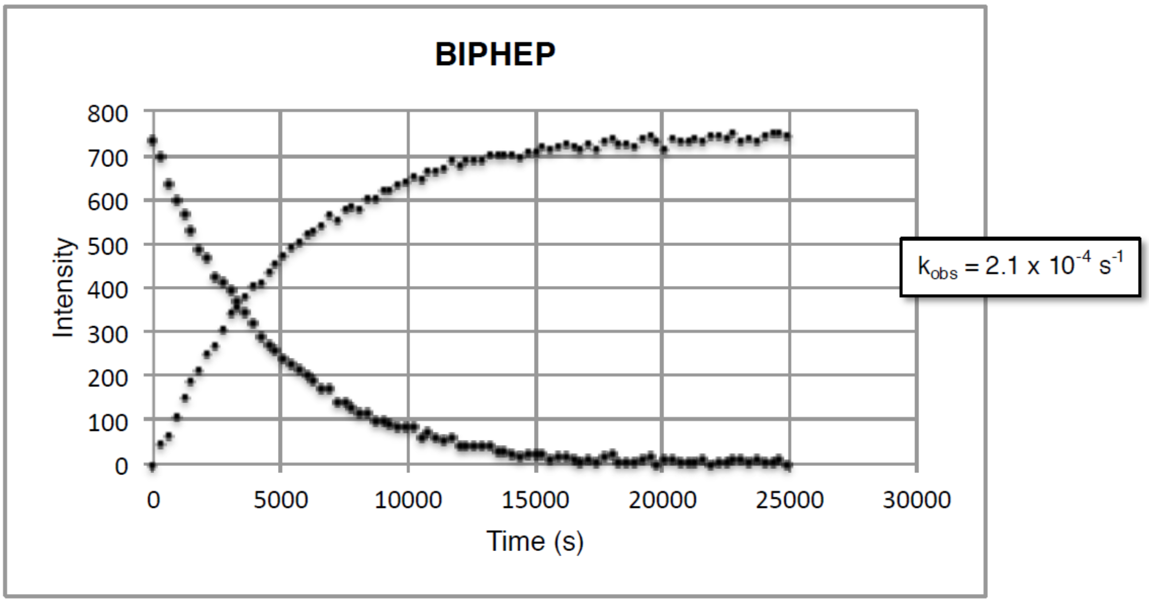

Figure S12. Plot of the decay of **3j** and formation of furans **4a+4b+4c** in the presence of 20 equiv PPh_3 at 35 ºC.

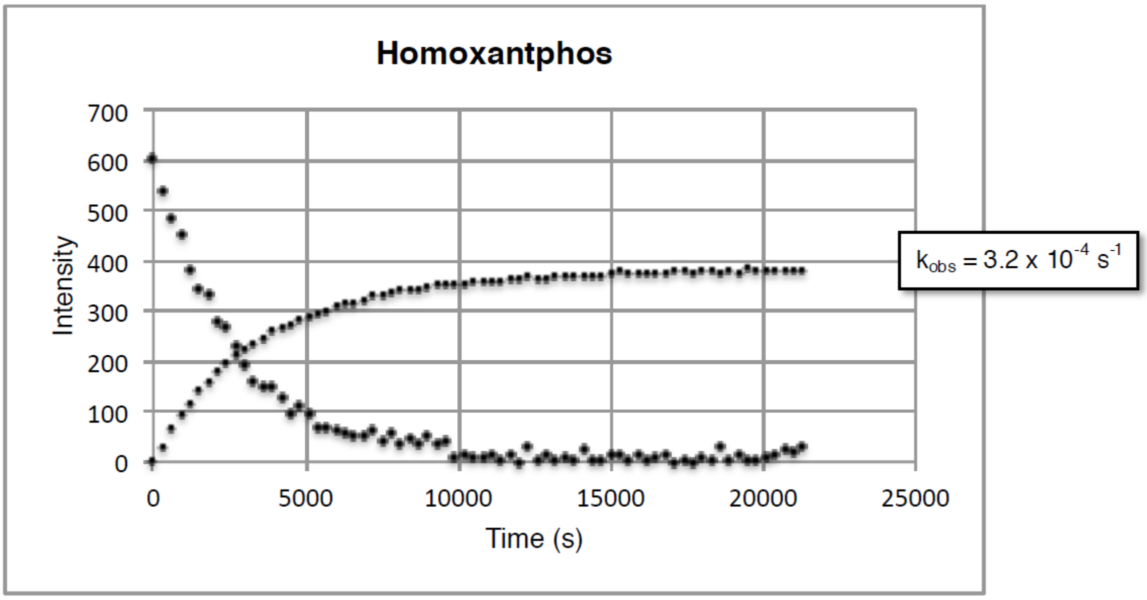

Figure S13. Plot of the decay of **3k** and formation of furans **4a+4b+4c** in the presence of 20 equiv PPh_3 at 35 ºC

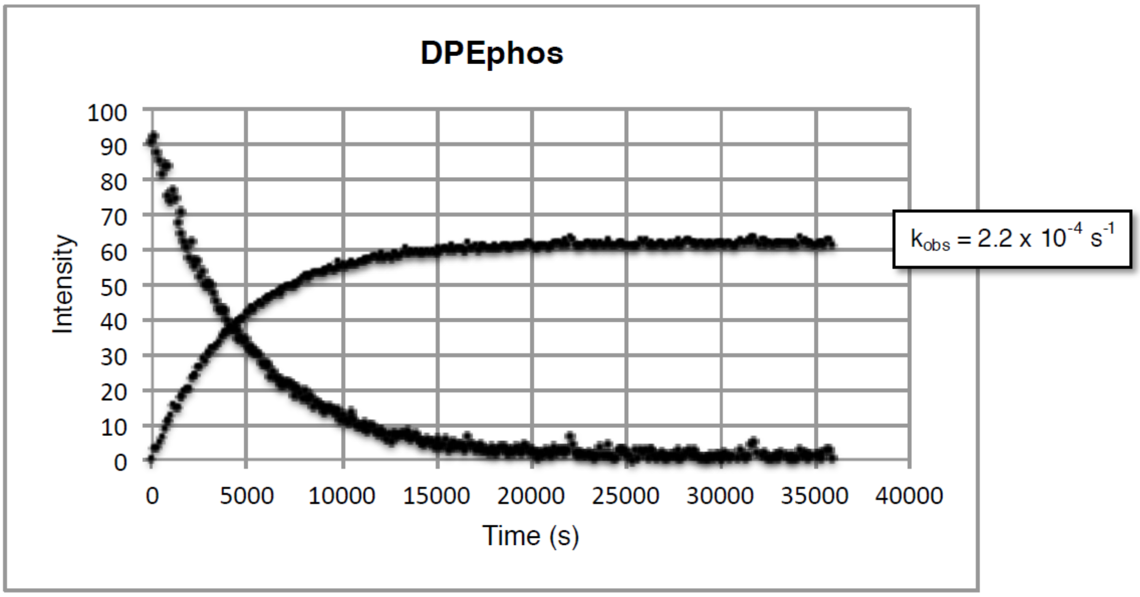

Figure S14. Plot of the decay of **3l** and formation of furans **4a+4b+4c** in the presence of 20 equiv PPh_3 at 35 ºC.

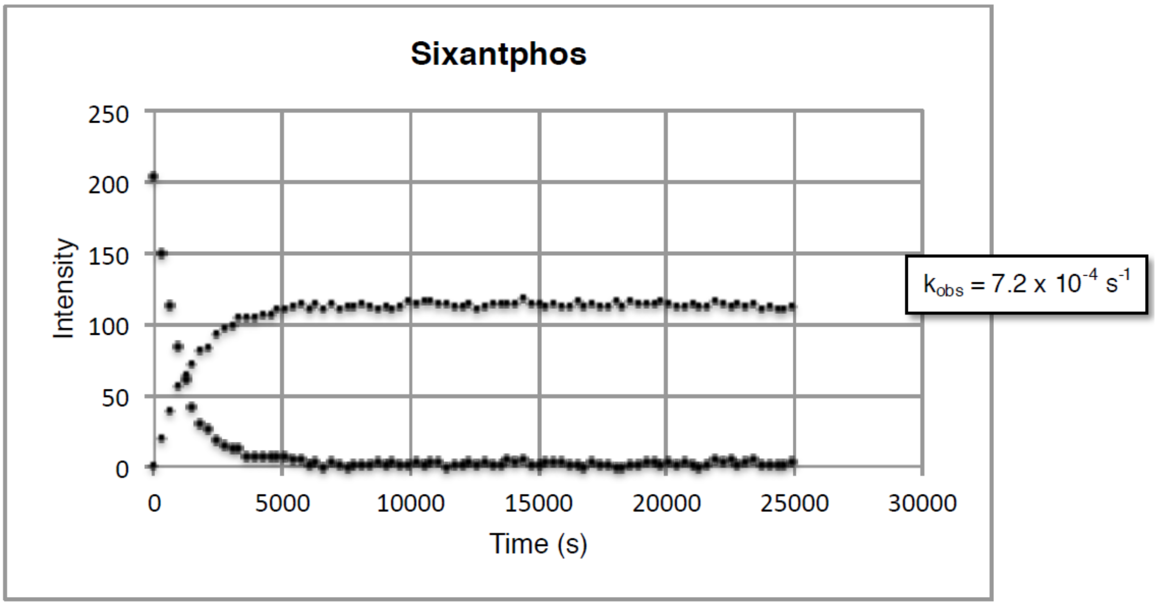

Figure S15. Plot of the decay of **3m** and formation of furans **4a+4b+4c** in the presence of 20 equiv PPh_3 at 35 ºC.

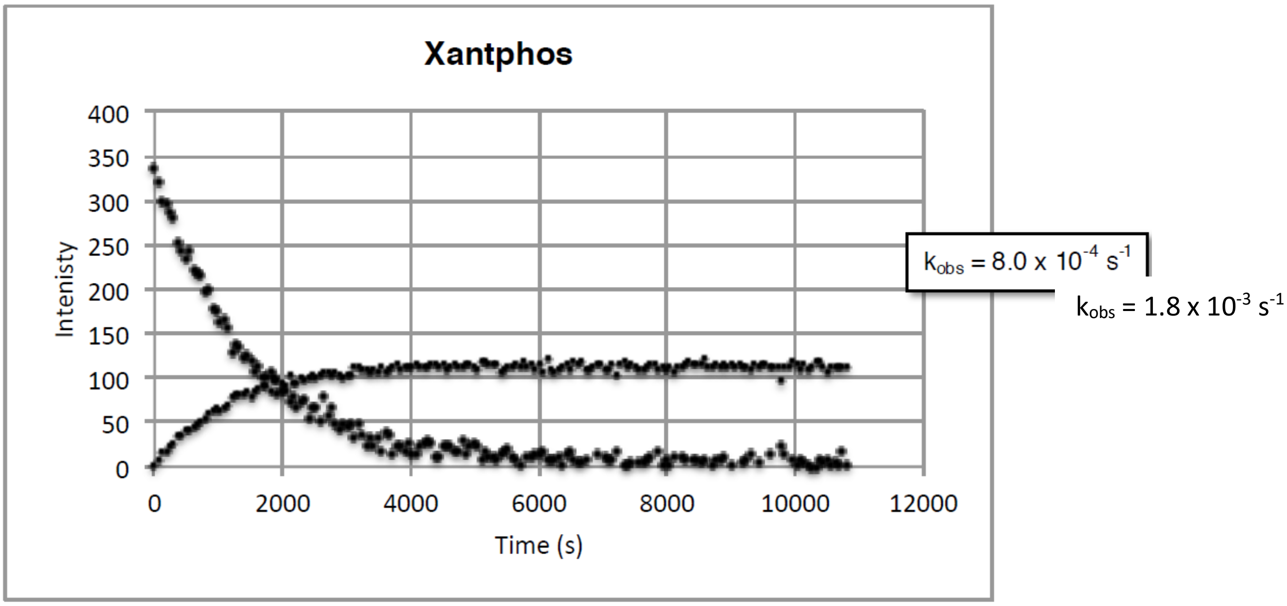

Spectroscopic Data

Figure S16. $^{31}P\{^1H\}$ NMR spectrum (202.2 MHz, toluene-d_8) of **1a** at 25 °C.

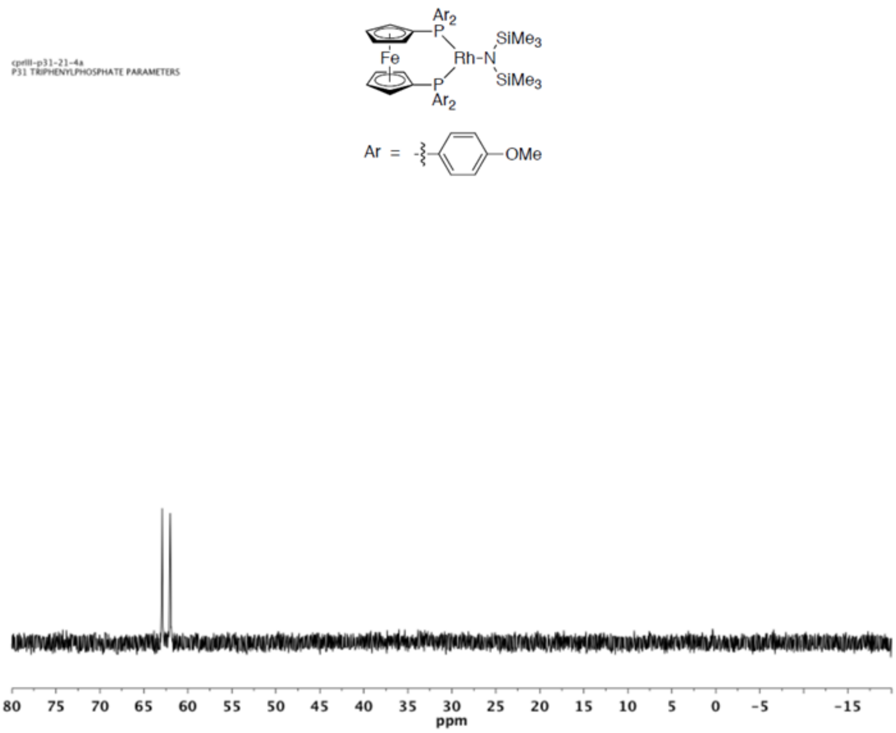

Figure S17. ^1H NMR spectrum (499.4 MHz, toluene-d_8) of **1a** with 1,3,5-trimethoxybenzene (TMB) as internal standard at 25 °C.

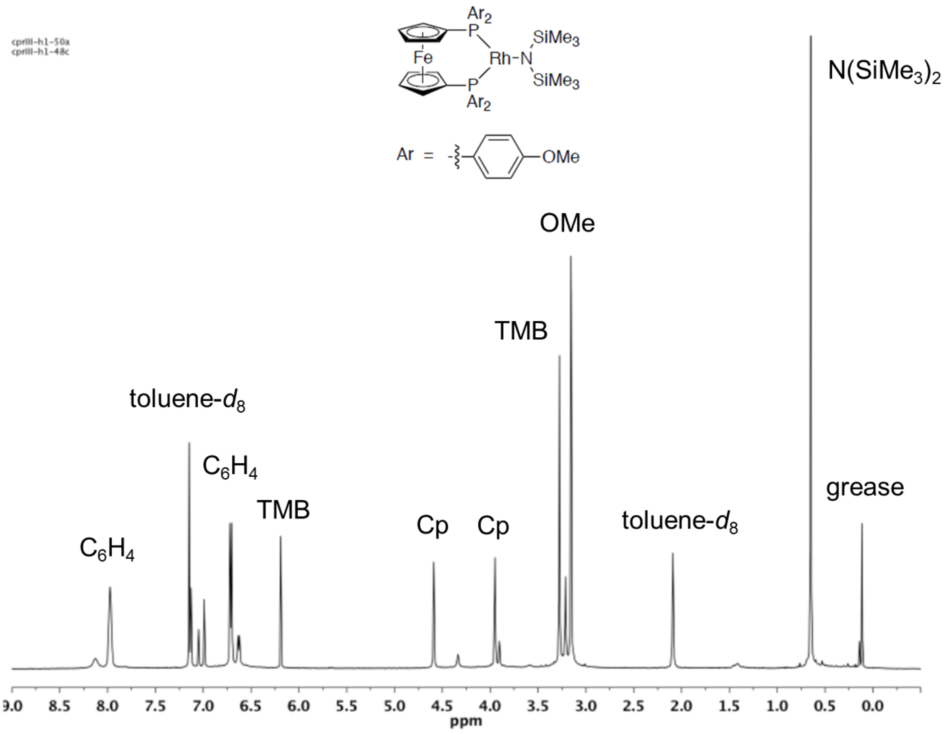

Figure S18. $^{31}P\{^1H\}$ NMR spectrum (202.2 MHz, toluene-d_8) of **1b** at 25 °C.

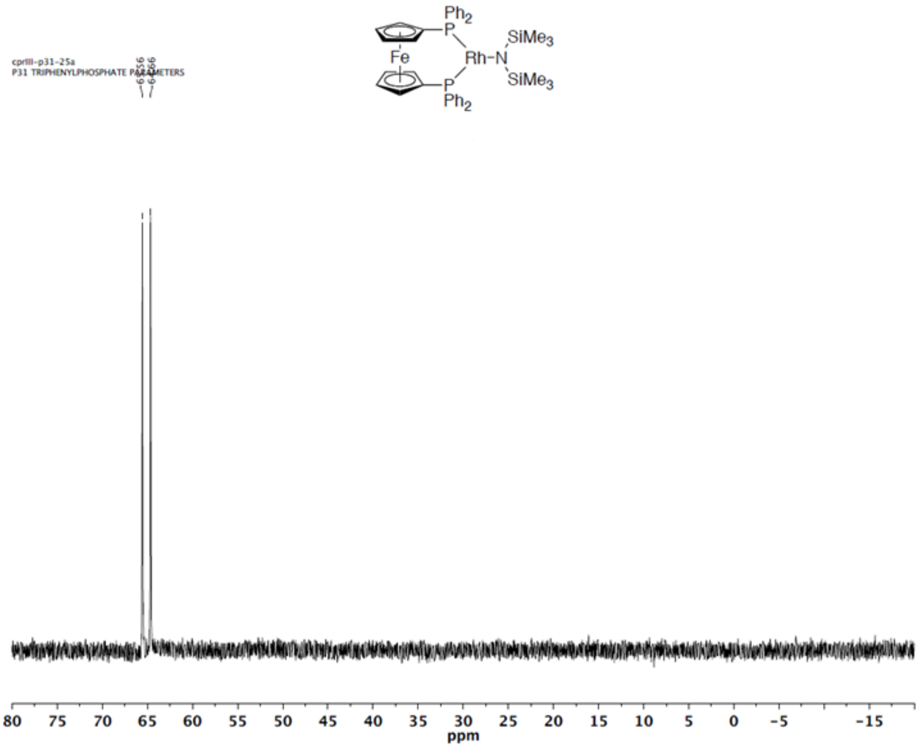

Figure S19. ^1H NMR spectrum (499.4 MHz, toluene-d_8) of **1b** with 1,3,5-trimethoxybenzene (TMB) as internal standard at 25 °C.

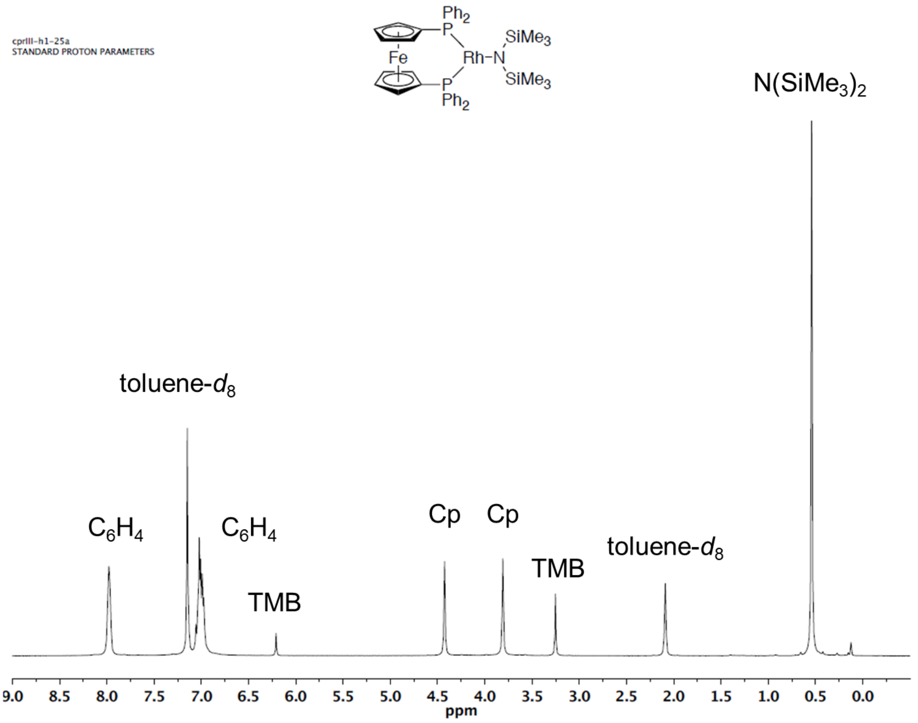

.

Figure S20. $^{31}P\{^1H\}$ NMR spectrum (202.2 MHz, THF-d_8) of **1c** at 25 °C.

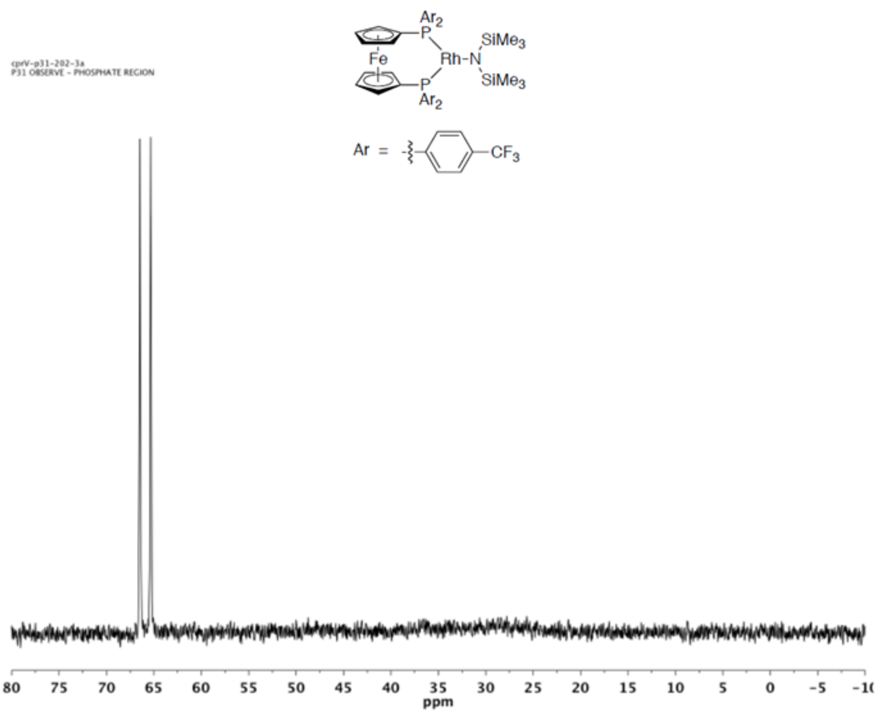

Figure S21. ^1H NMR spectrum (499.4 MHz, THF-d_8) of **1c** with 1,3,5-trimethoxybenzene (TMB) as internal standard at 25 °C.

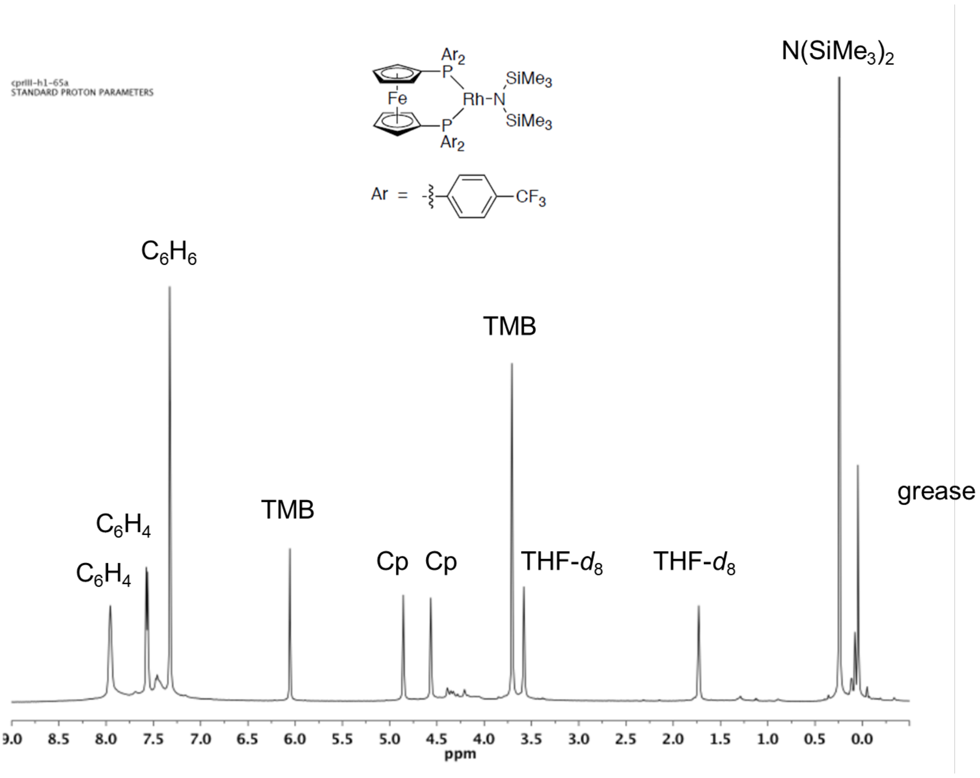

Figure S22. $^{31}P\{^1H\}$ NMR spectrum (202.2 MHz, toluene-d_8) of **1d** at 25 °C.

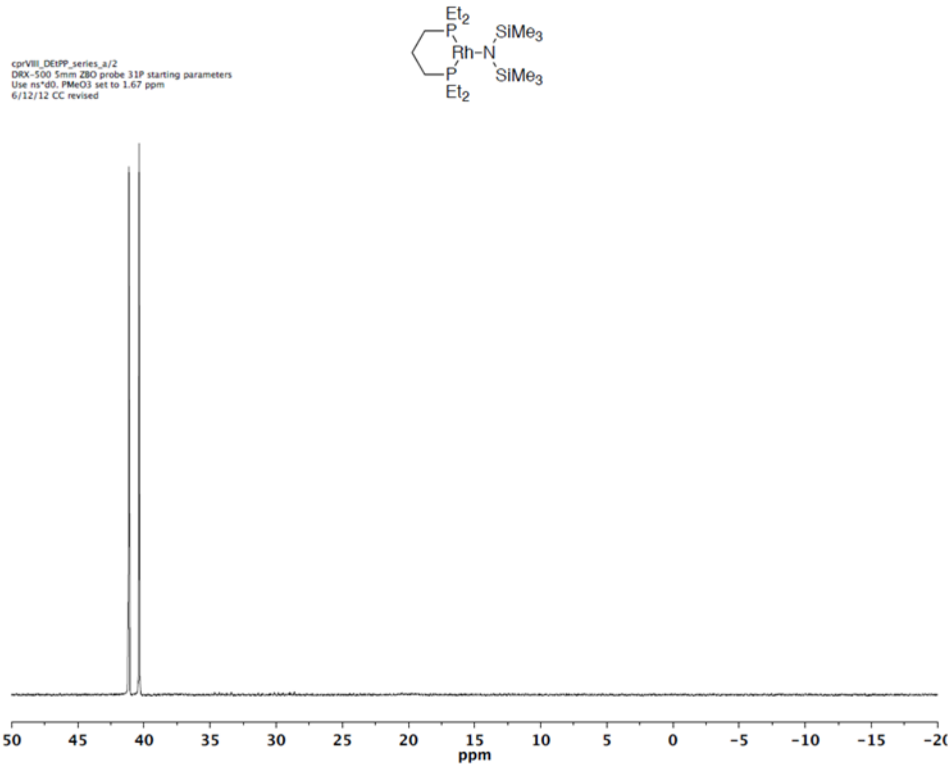

Figure S23. ^1H NMR spectrum (499.4 MHz, toluene-d_8) of **1d** with 1,3,5-trimethoxybenzene (TMB) as internal standard at 25 °C.

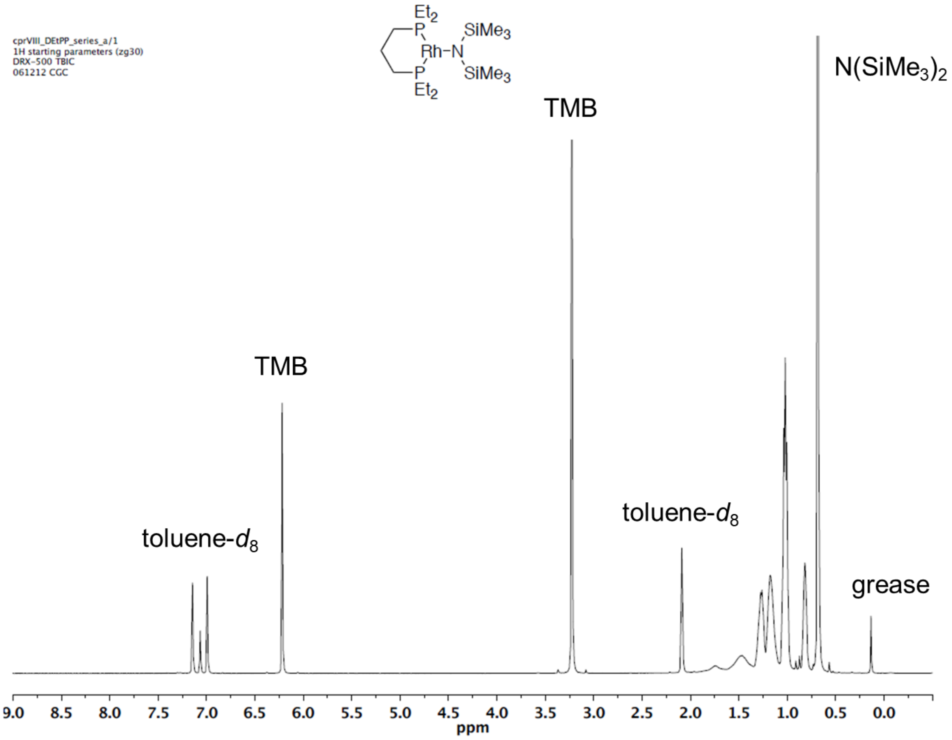

Figure S24. $^{31}P\{^{1}H\}$ NMR spectrum (202.2 MHz, THF-d_8) of **1e** at 25 °C.

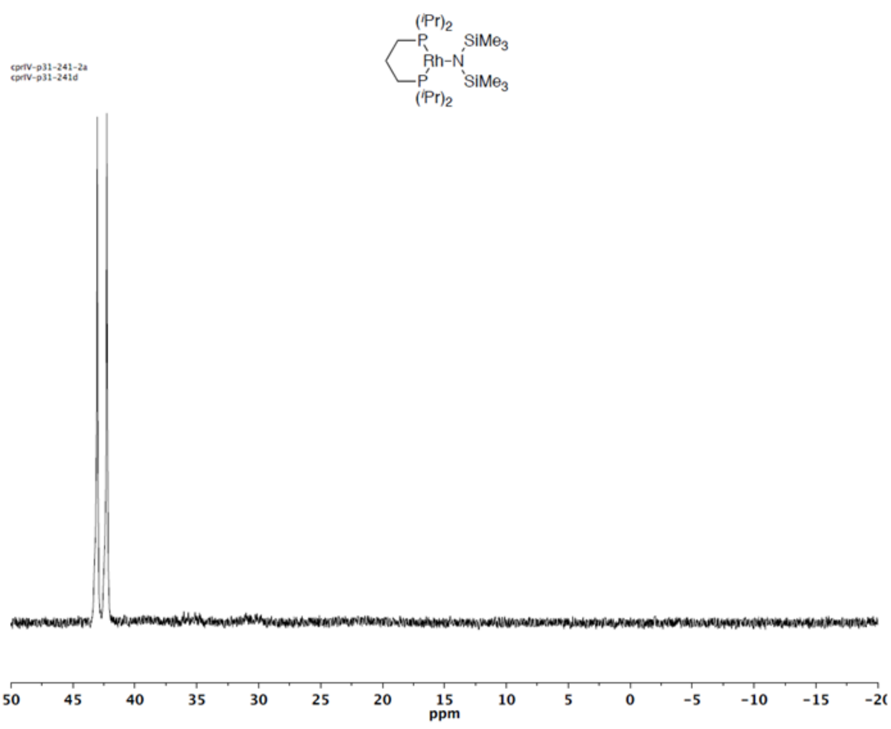

Figure S25. ^{1}H NMR spectrum (499.4 MHz, THF-d_8) of **1e** with 1,3,5-trimethoxybenzene (TMB) as internal standard at 25 °C.

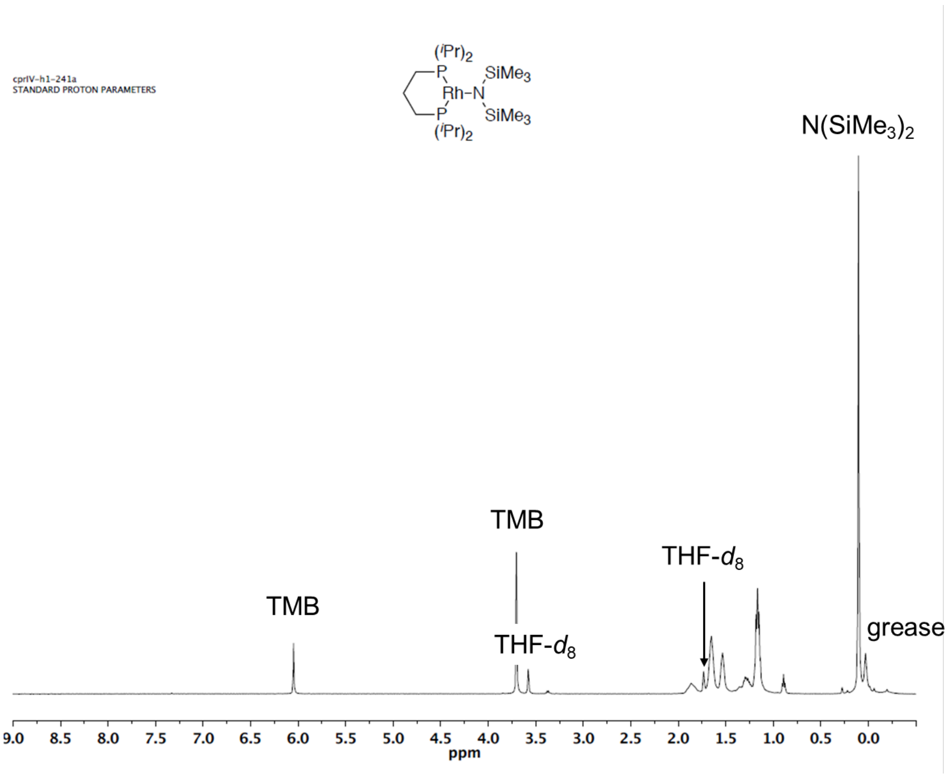

Figure S26. $^{31}P\{^1H\}$ NMR spectrum (202.2 MHz, toluene-d_8) of **1f** at 25 °C.

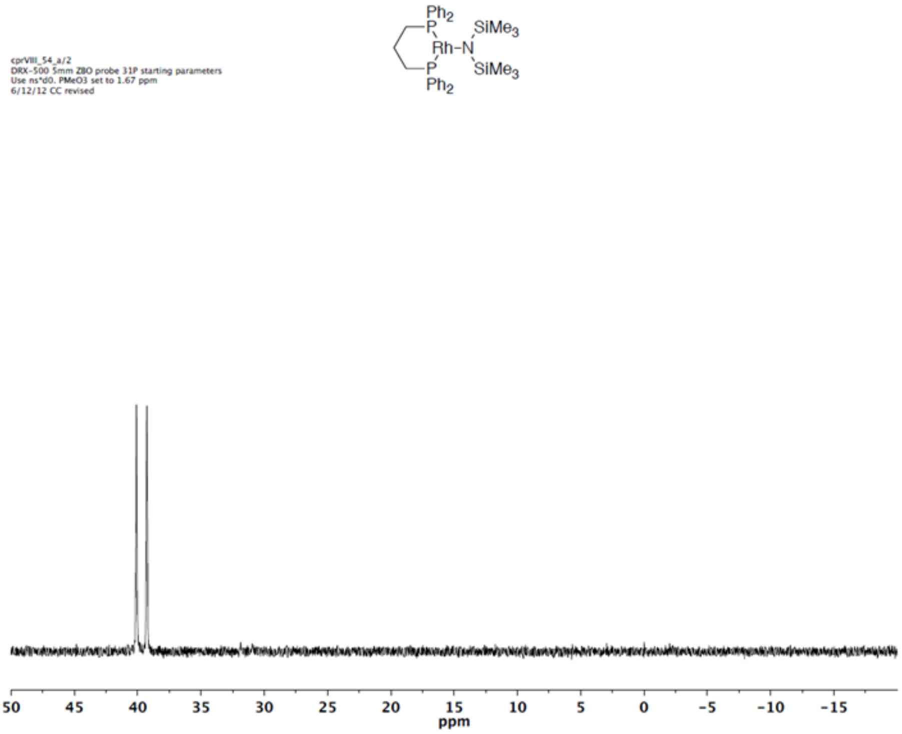

Figure S27. ^1H NMR spectrum (499.4 MHz, toluene-d_8) of **1f** at 25 °C.

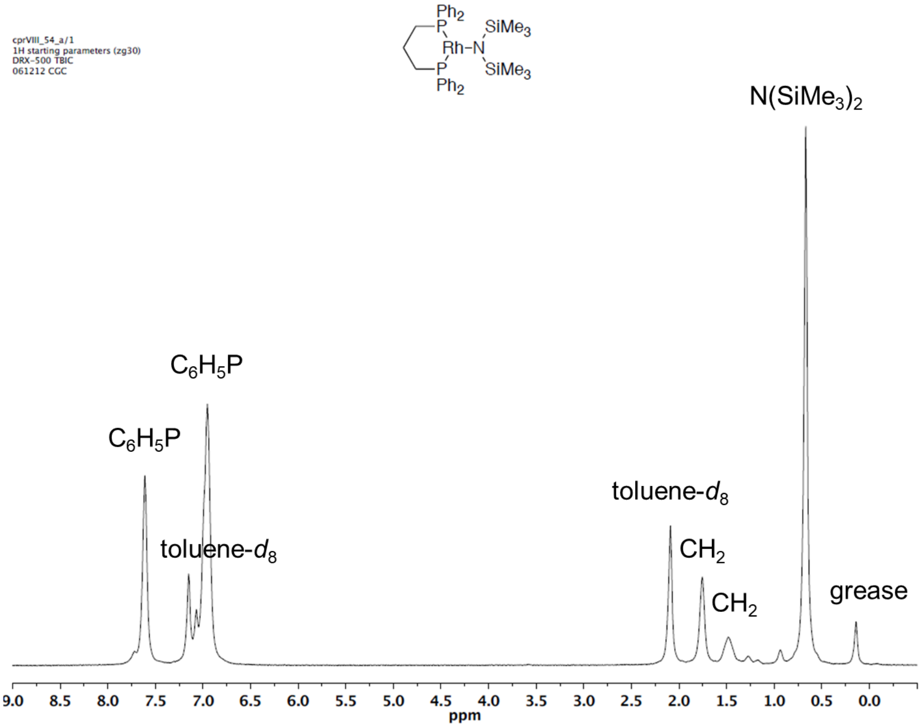

Figure S28. $^{31}P\{^1H\}$ NMR spectrum (202.2 MHz, toluene-d_8) of **1g** at 25 °C.

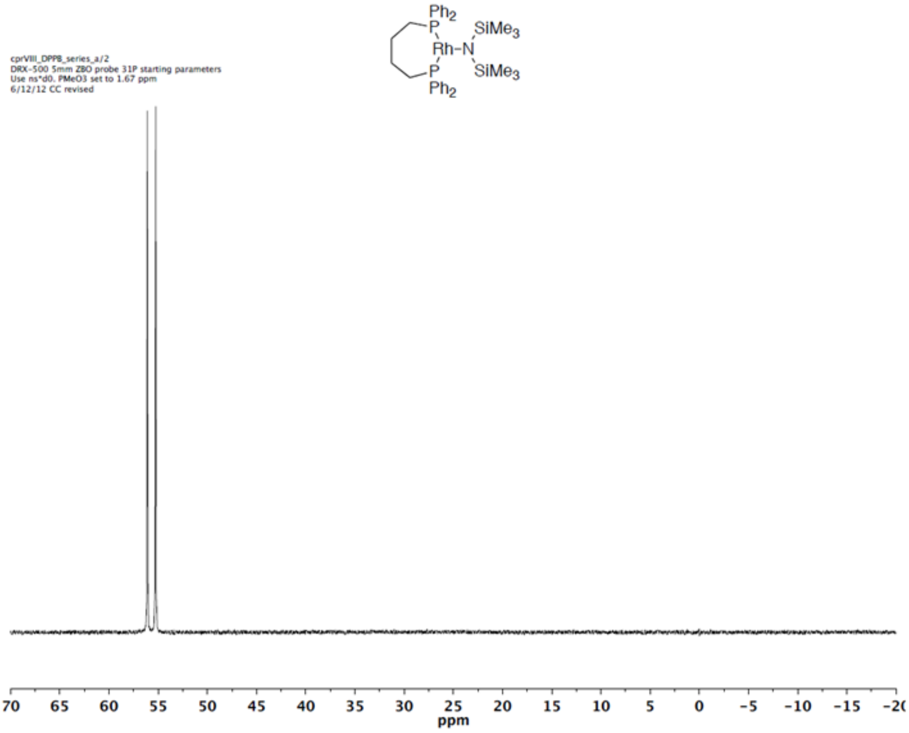

Figure S29. ^1H NMR spectrum (499.4 MHz, toluene-d_8) of **1g** with 1,3,5-trimethoxybenzene (TMB) as internal standard at 25 °C.

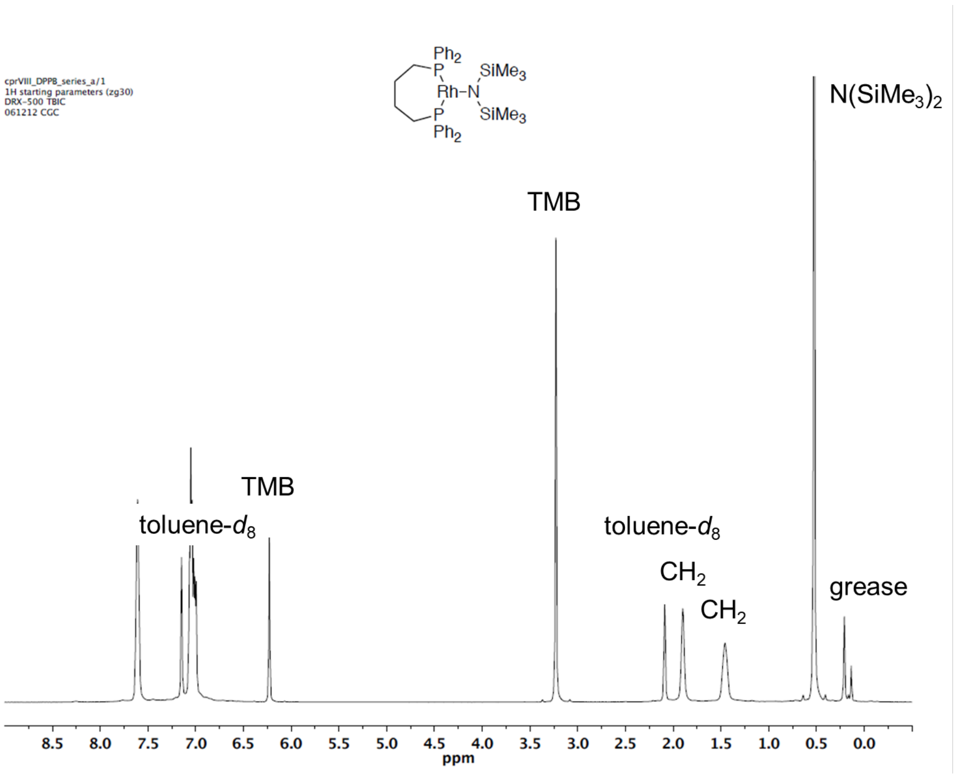

Figure S30. $^{31}P\{^{1}H\}$ NMR spectrum (202.2 MHz, toluene-d_8) of **1h** at 25 °C.

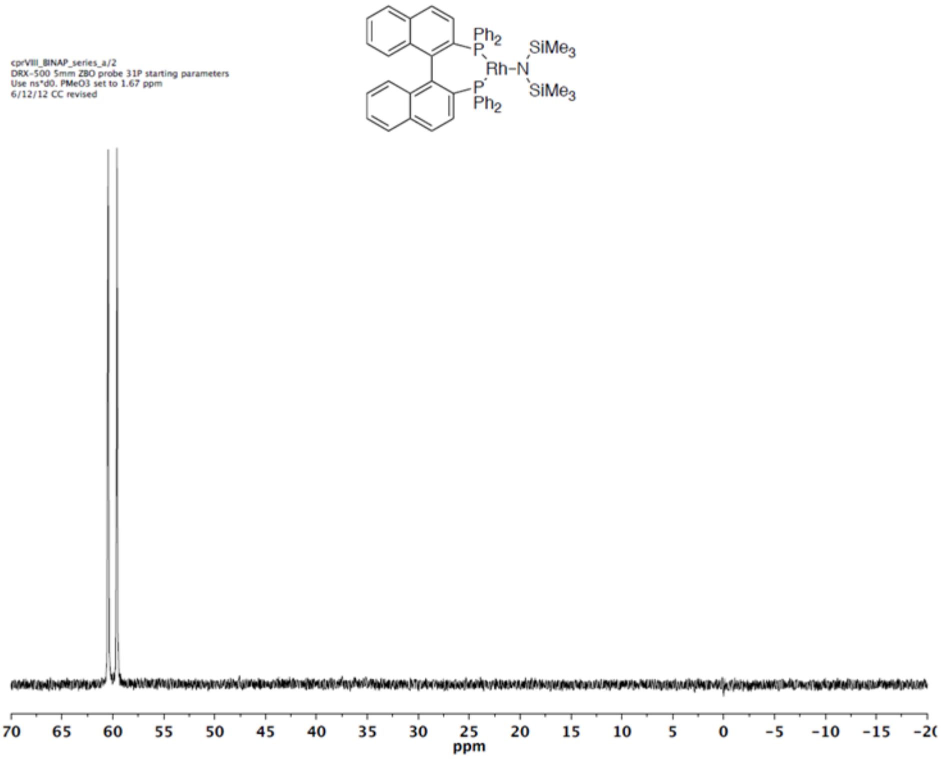

Figure S31. ^{1}H NMR spectrum (499.4 MHz, toluene-d_8) of **1h** with 1,3,5-trimethoxybenzene (TMB) as internal standard at 25 °C.

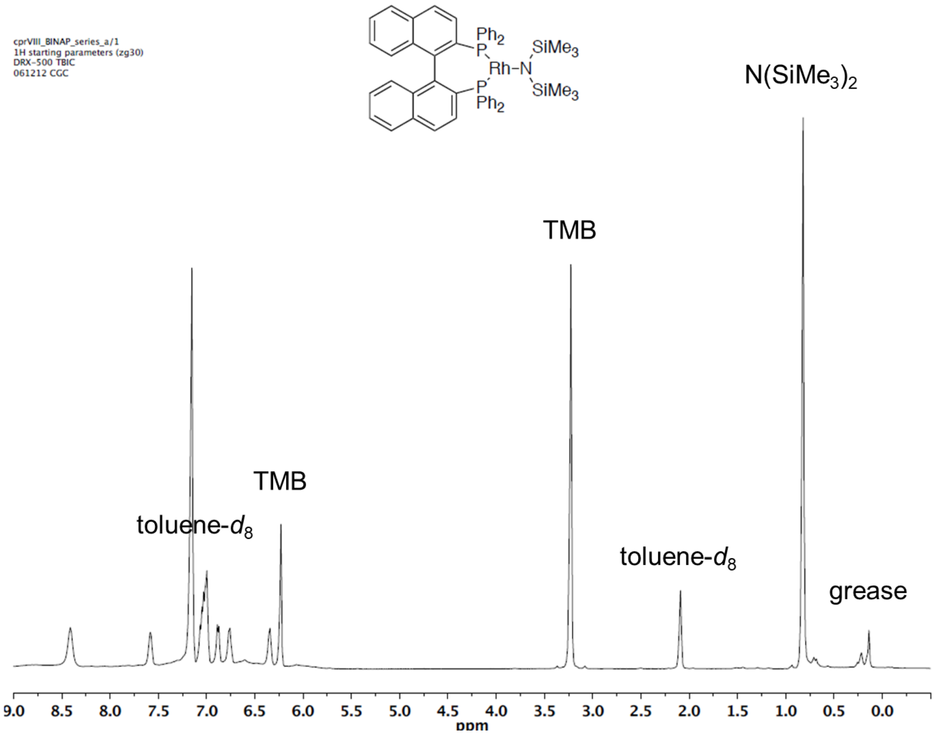

Figure S32. $^{31}P\{^{1}H\}$ NMR spectrum (202.2 MHz, THF-d_8) of **1i**.

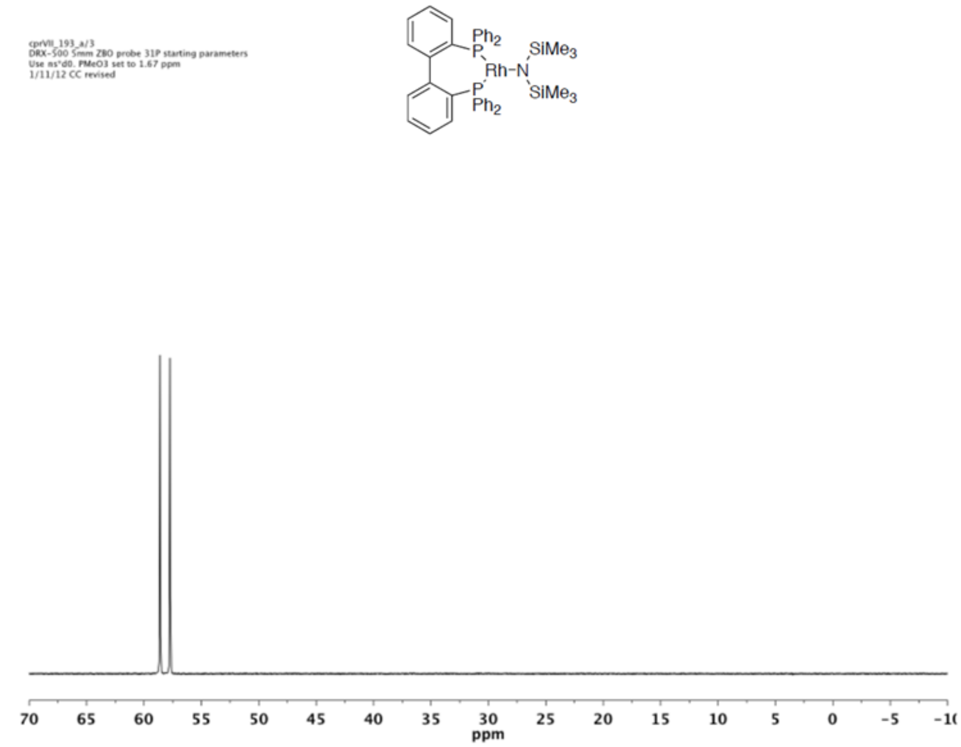

Figure S33. ^{1}H NMR spectrum (499.4 MHz, THF-d_8) of **1i** with 1,3,5-trimethoxybenzene (TMB) as internal standard at 25 °C.

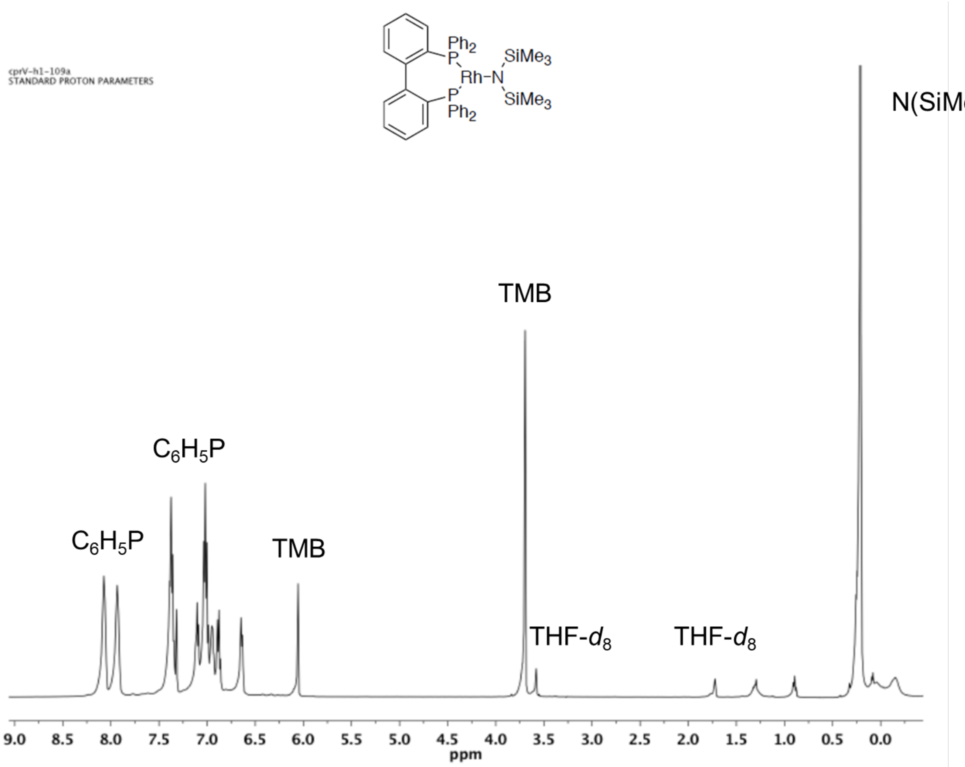

Figure S34. $^{31}P\{^1H\}$ NMR spectrum (202.2 MHz, toluene-d_8) of **1j** at 25 °C.

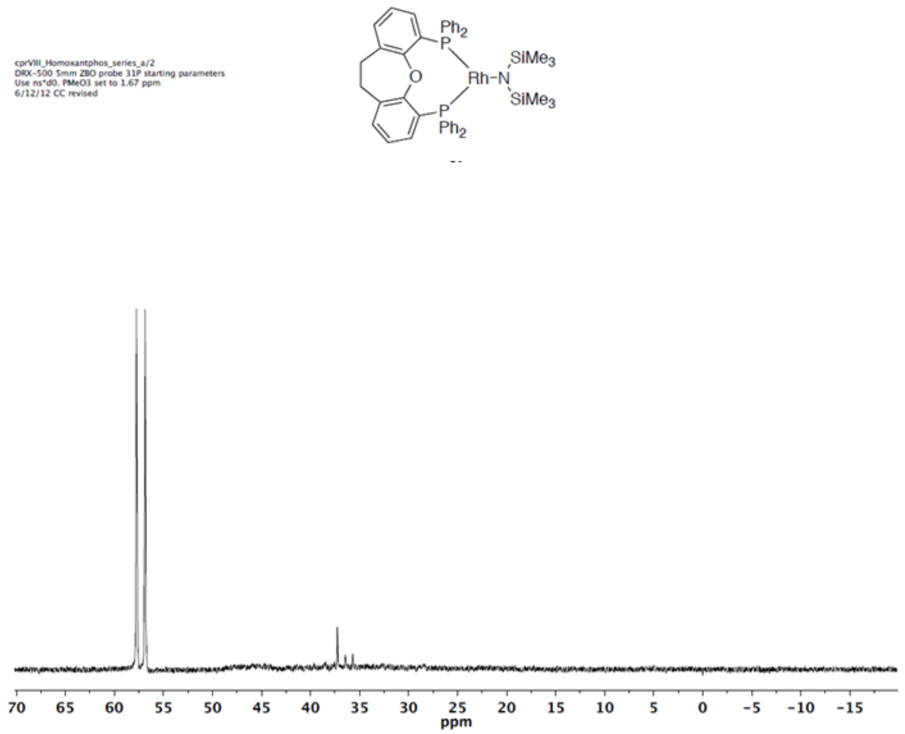

Figure S35. ^1H NMR spectrum (499.4 MHz, toluene-d_8) of **1j** with 1,3,5-trimethoxybenzene (TMB) as internal standard at 25 °C.

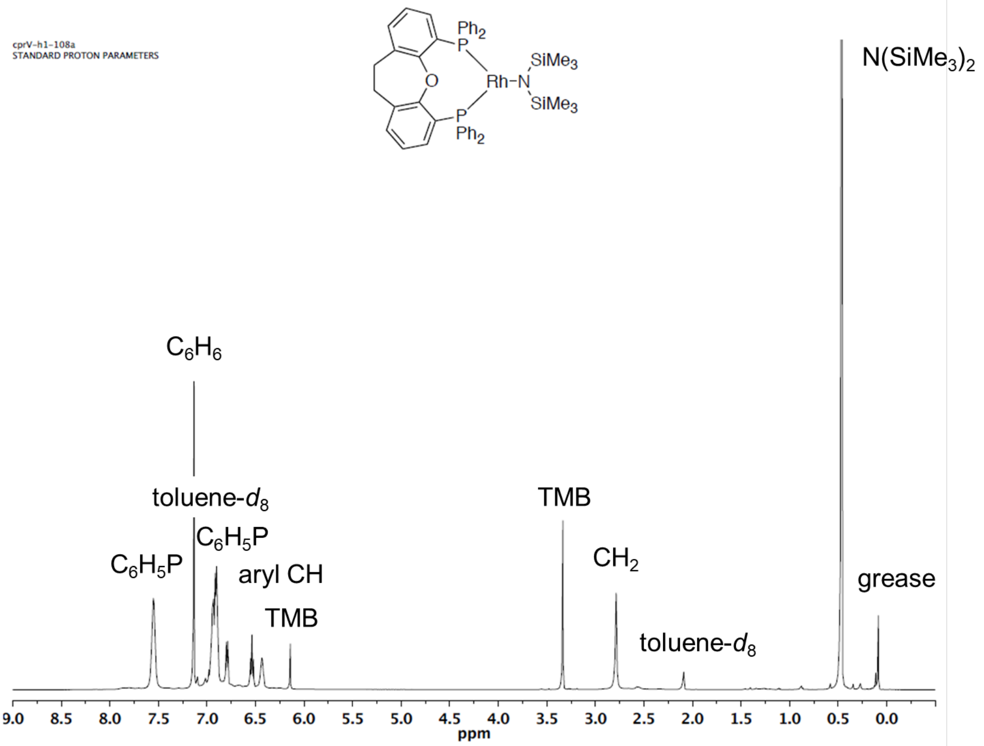

Figure S36. $^{31}P\{^{1}H\}$ NMR spectrum (202.2 MHz, toluene-d_8) of **1k** at 25 °C.

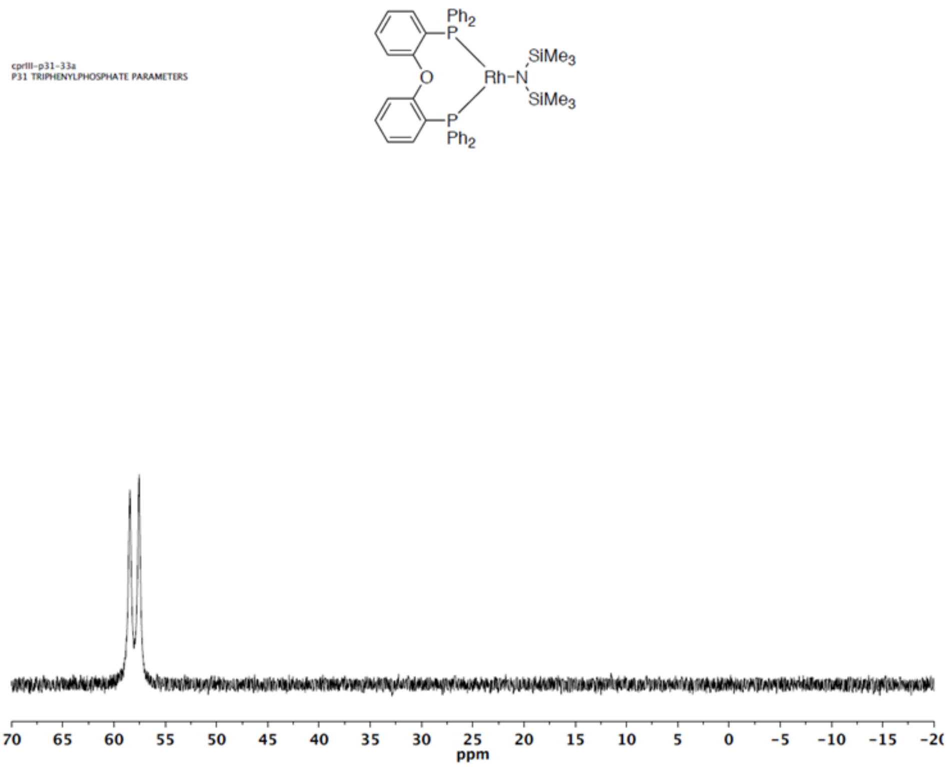

Figure S37. ^{1}H NMR spectrum (499.4 MHz, toluene-d_8) of **1k** with 1,3,5-trimethoxybenzene (TMB) as internal standard at 25 °C.

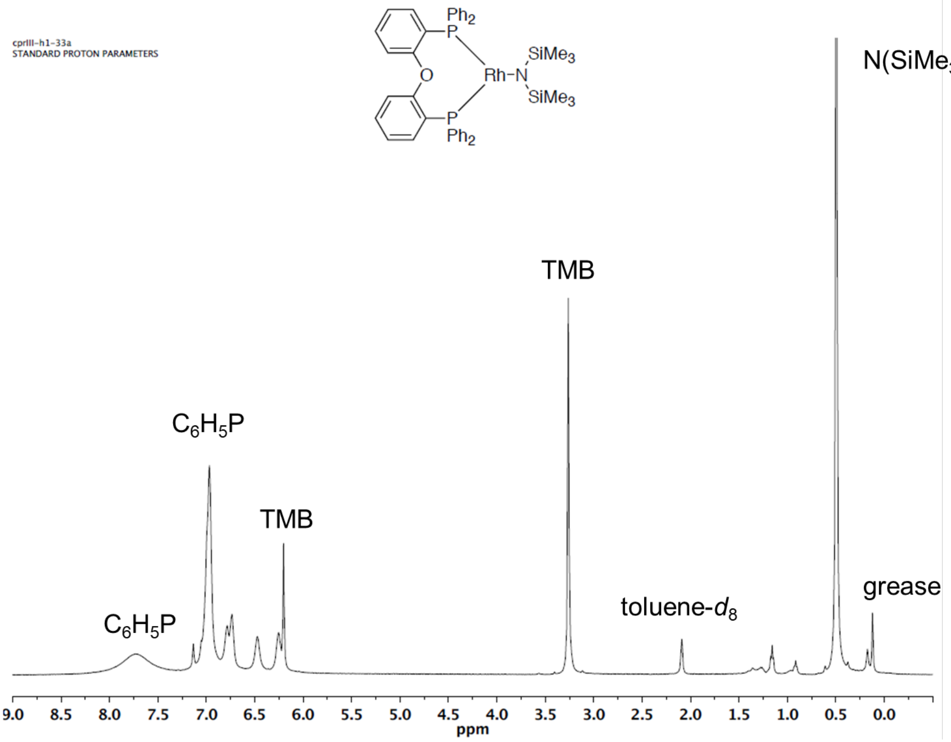

Figure S38. $^{31}P\{^{1}H\}$ NMR spectrum (202.2 MHz, toluene-d_8) of **1l** at 25 °C.

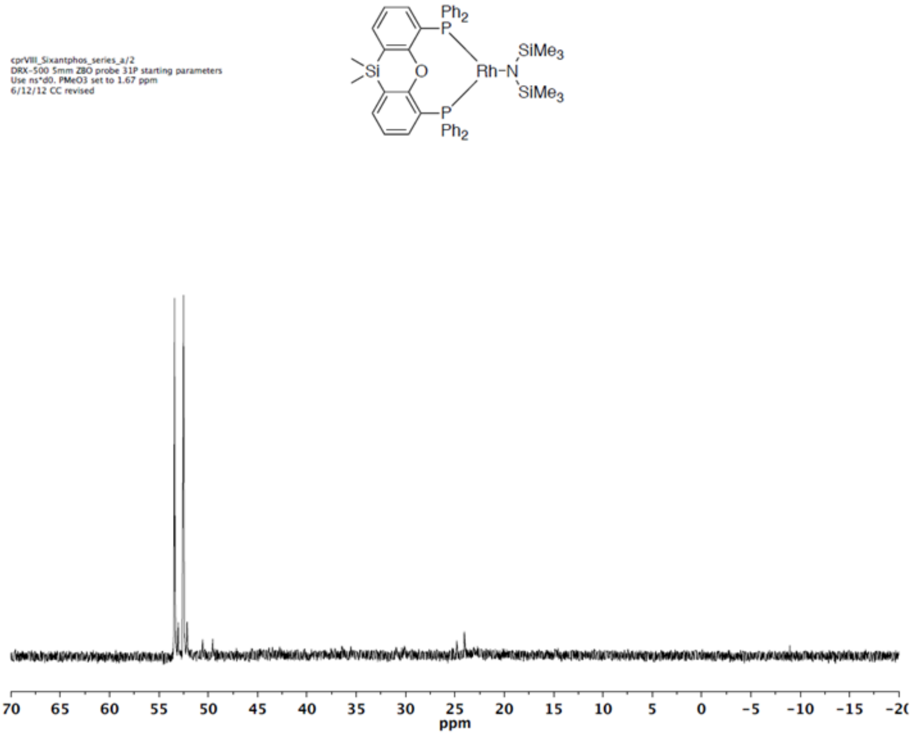

Figure S39. ^{1}H NMR spectrum (499.4 MHz, toluene-d_8) of **1l** with 1,3,5-trimethoxybenzene (TMB) as internal standard at 25 °C.

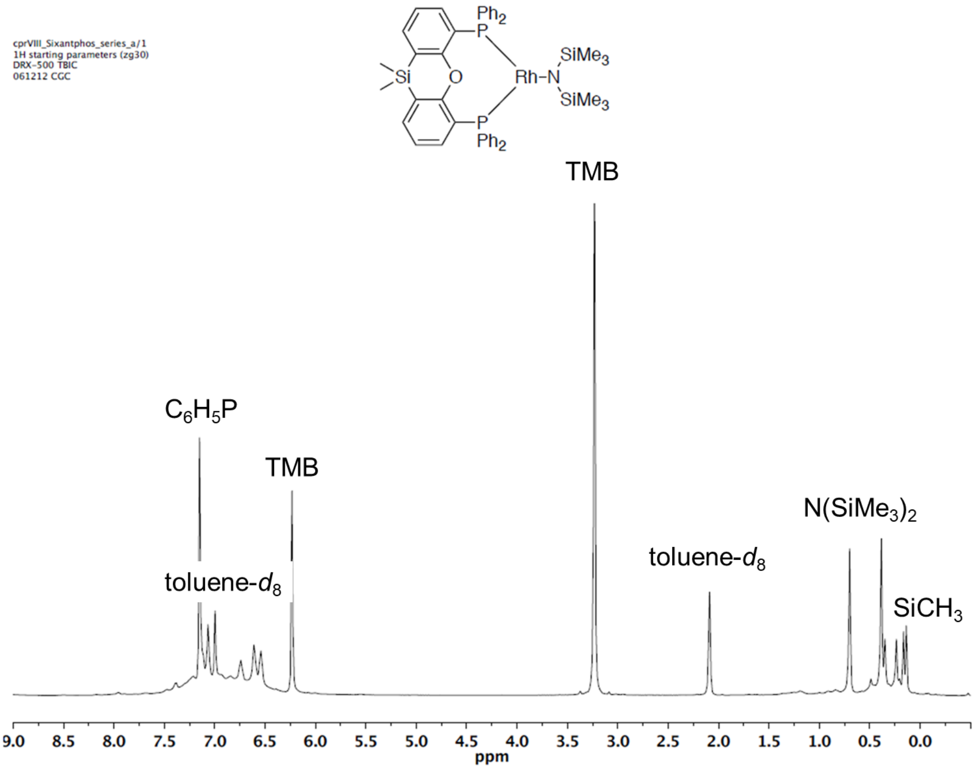

Figure S40. $^{31}P\{^{1}H\}$ NMR spectrum (202.2 MHz, toluene-d_8) of **1m** at 25 °C.

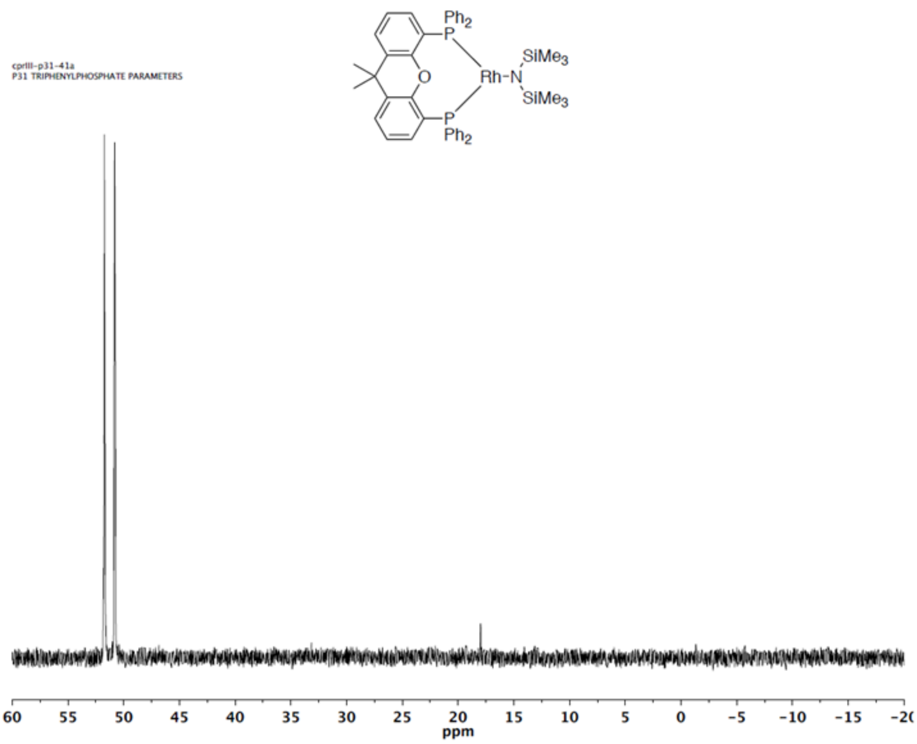

Figure S41. ^{1}H NMR spectrum (499.4 MHz, toluene-d_8) of **1m** with 1,3,5-trimethoxybenzene (TMB) as internal standard at 25 °C.

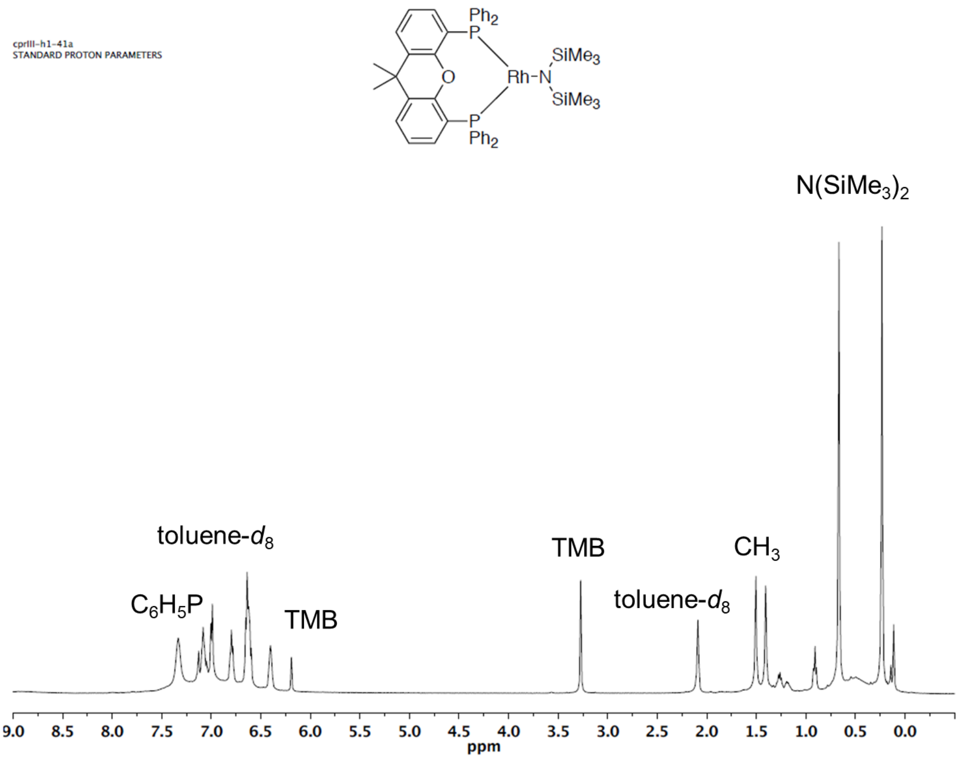

Figure S42. $^{31}P\{^{1}H\}$ NMR spectrum (202.2 MHz, toluene-d_8) of **3a** at -40 °C.

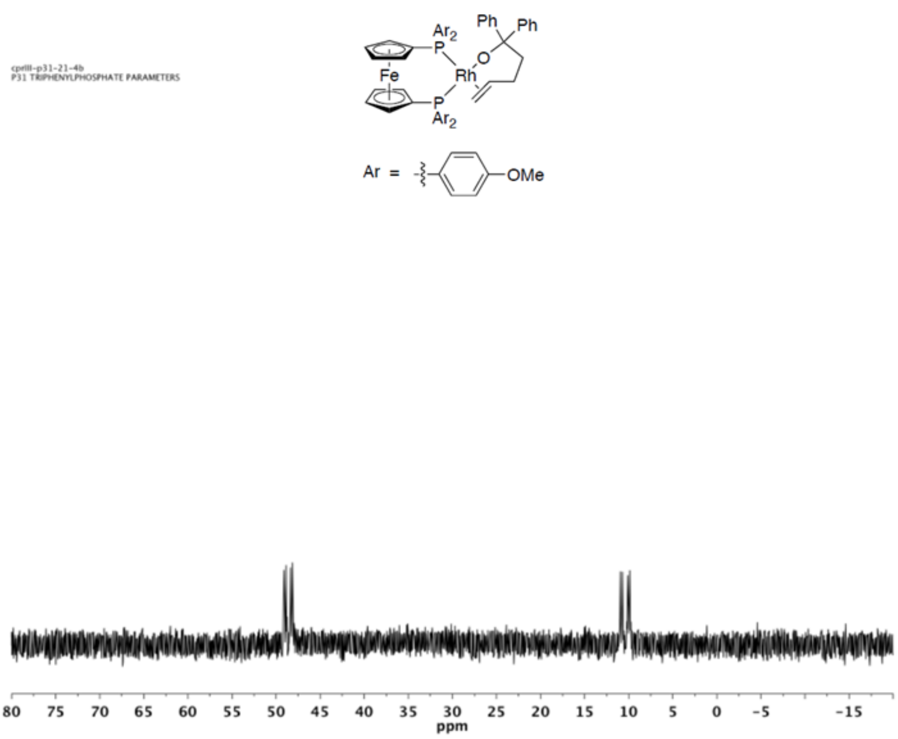

Figure S43. ^{1}H NMR spectrum (499.4 MHz, toluene-d_8) of **3a** with 1,3,5-trimethoxybenzene (TMB) as internal standard at -40 °C.

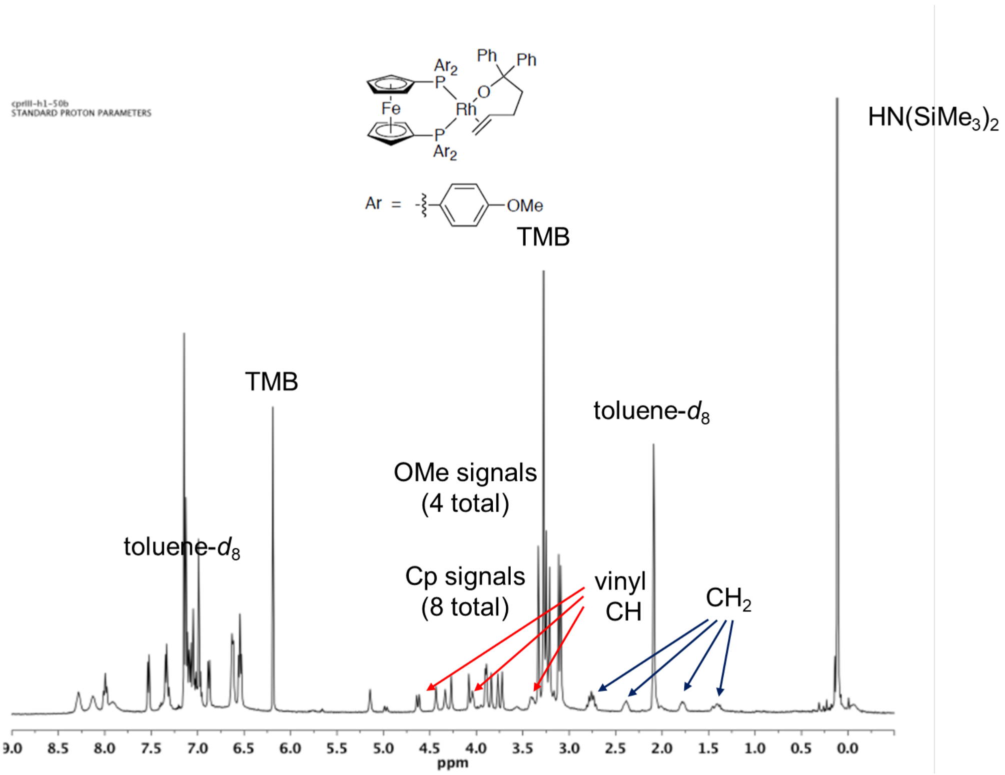

Figure S44. $^{13}C\{^{1}H\}$ NMR spectrum (125.7 MHz, toluene-d_8) of **3a** with 1,3,5-trimethoxybenzene (TMB) as internal standard at -40 °C.

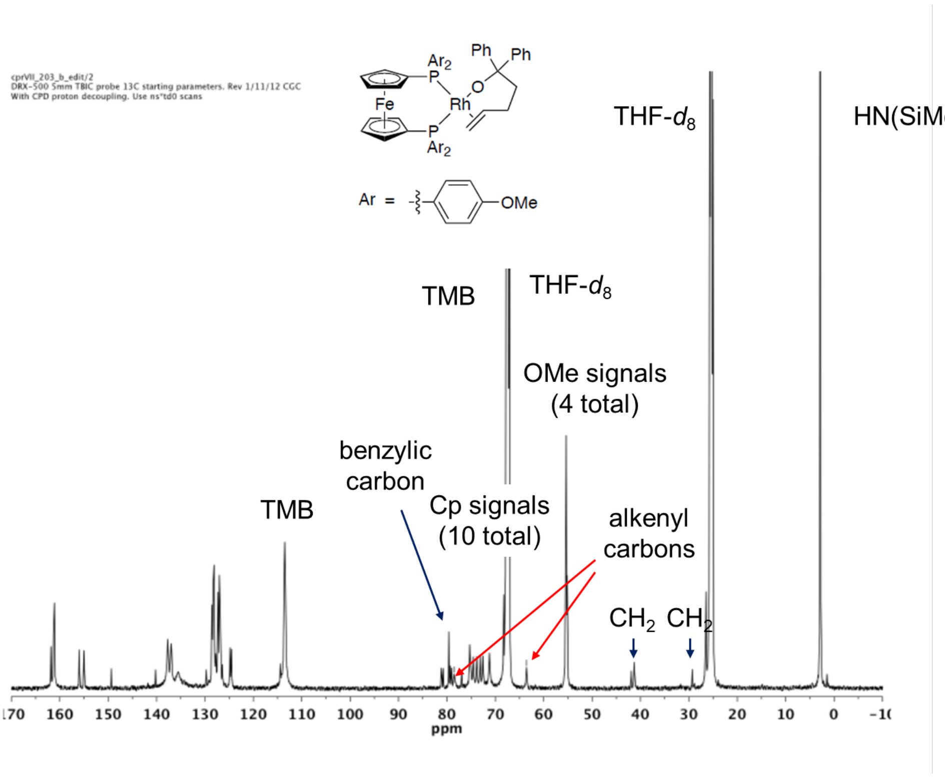

Figure S45. $^{31}P\{^{1}H\}$ NMR spectrum (202.2 MHz, toluene-d_8) of **3b** at -40 °C.

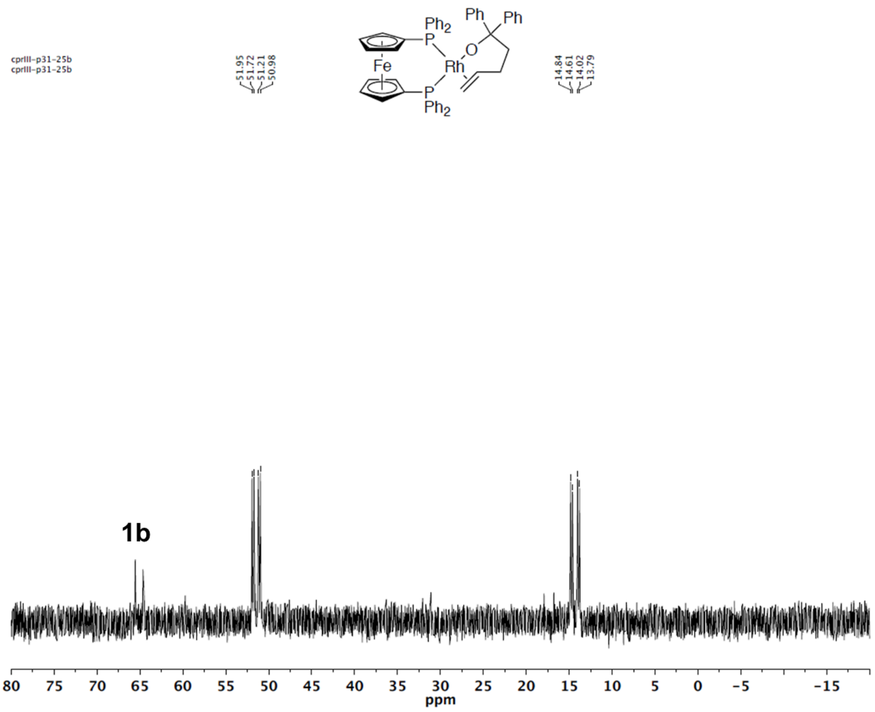

Figure S46. ^{1}H NMR spectrum (499.4 MHz, toluene-d_8) of **3b** with 1,3,5-trimethoxybenzene (TMB) as internal standard at -40 °C.

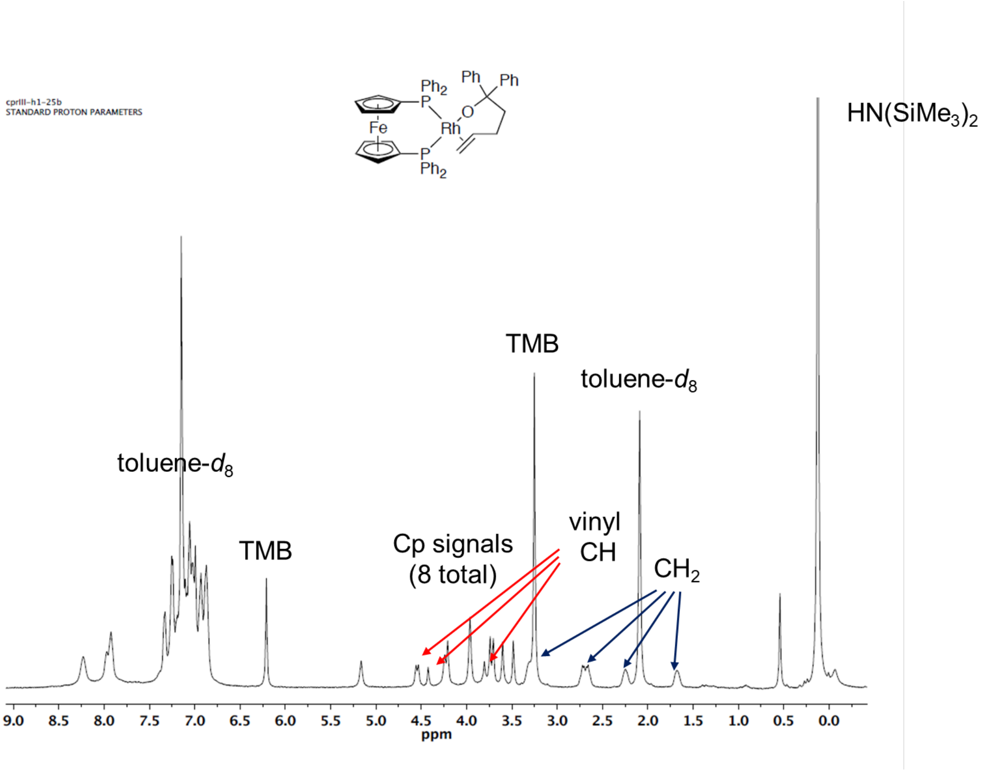

Figure S47. $^{13}C\{^{1}H\}$ NMR spectrum (125.7 MHz, toluene-d_8) of **3b** with 1,3,5-trimethoxybenzene (TMB) as internal standard at -40 °C.

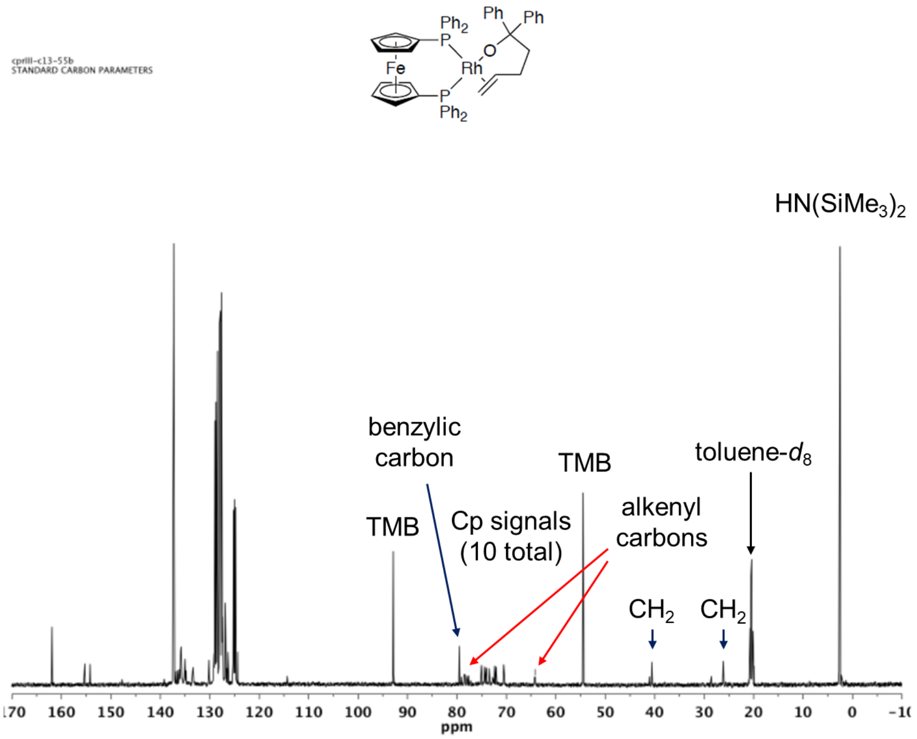

Figure S48. $^{31}P\{^1H\}$ NMR spectrum (202.2 MHz, THF-d_8) of **3c** at -40 °C.

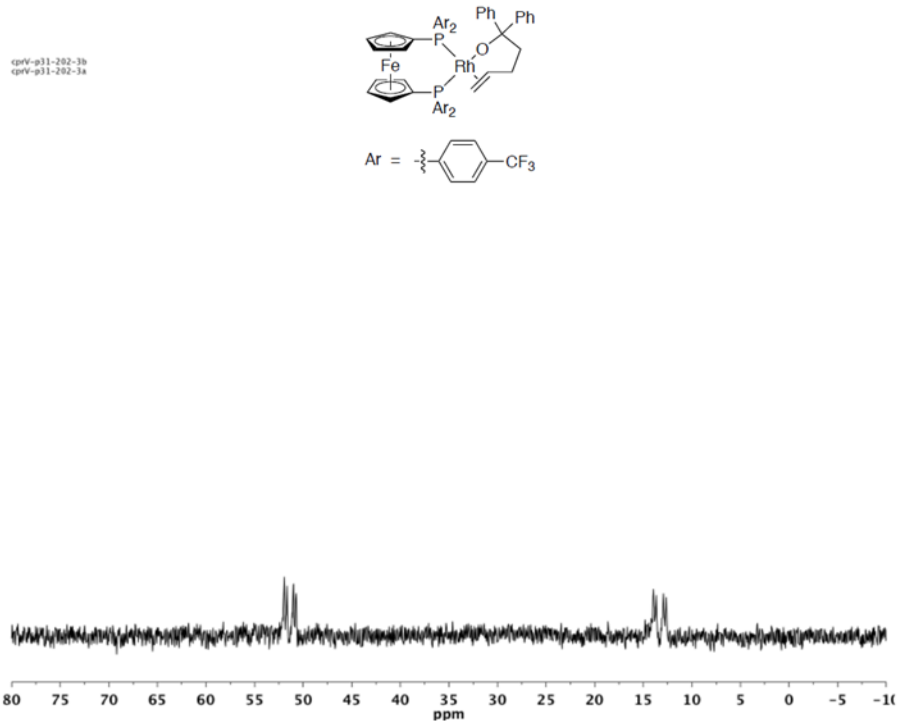

Figure S49. ^1H NMR spectrum (499.4 MHz, THF-d_8) of 3**c** with 1,3,5-trimethoxybenzene (TMB) as internal standard at -40 °C.

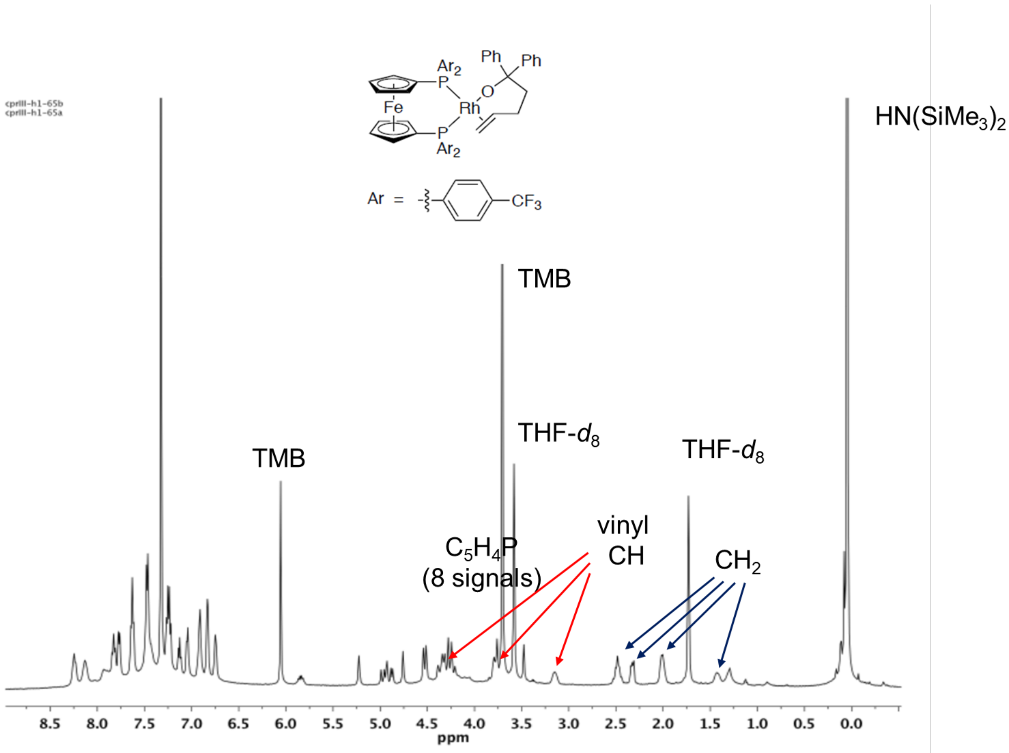

Figure S50. $^{31}P\{^{1}H\}$ NMR spectrum (202.2 MHz, toluene-d_8) of **3d** at -40 °C.

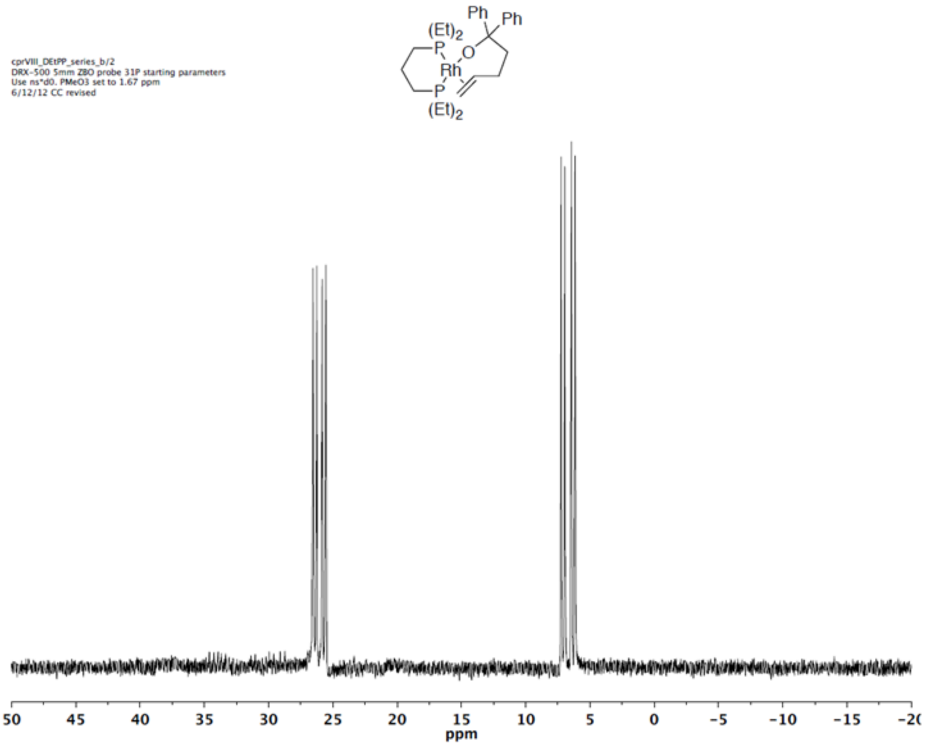

Figure S51. ^{1}H NMR spectrum (499.4 MHz, toluene-d_8) of **3d** with 1,3,5-trimethoxybenzene (TMB) as internal standard at -40 °C.

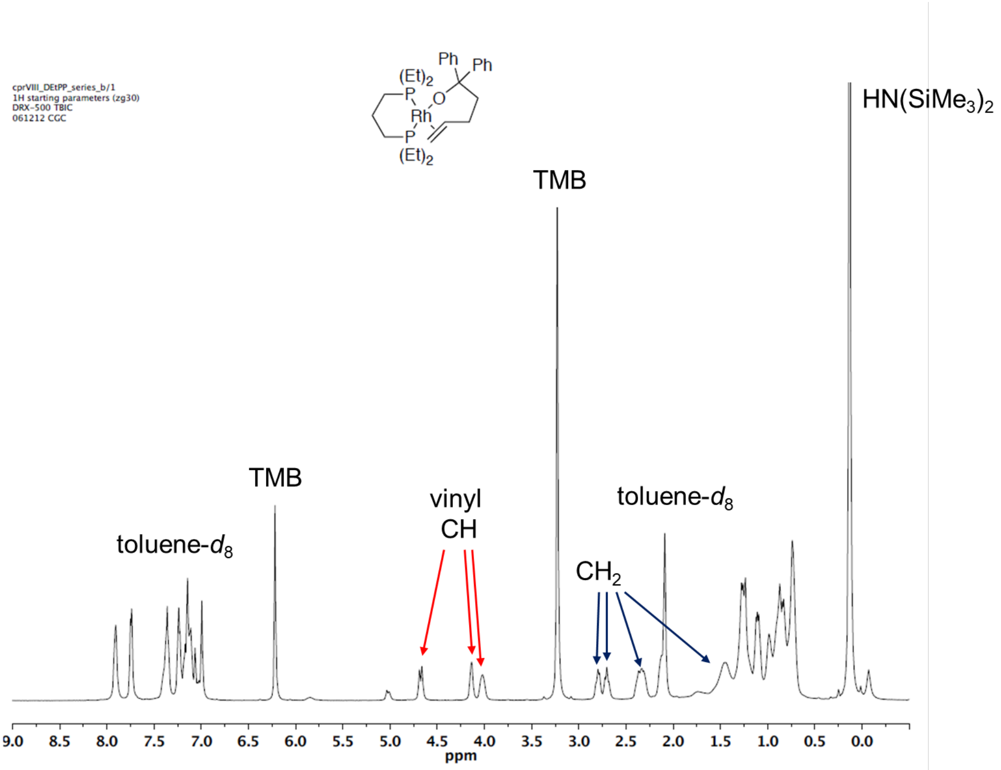

Figure S52. $^{13}C\{^1H\}$ NMR spectrum (125.7 MHz, toluene-d_8) of **3d** at -40 °C.

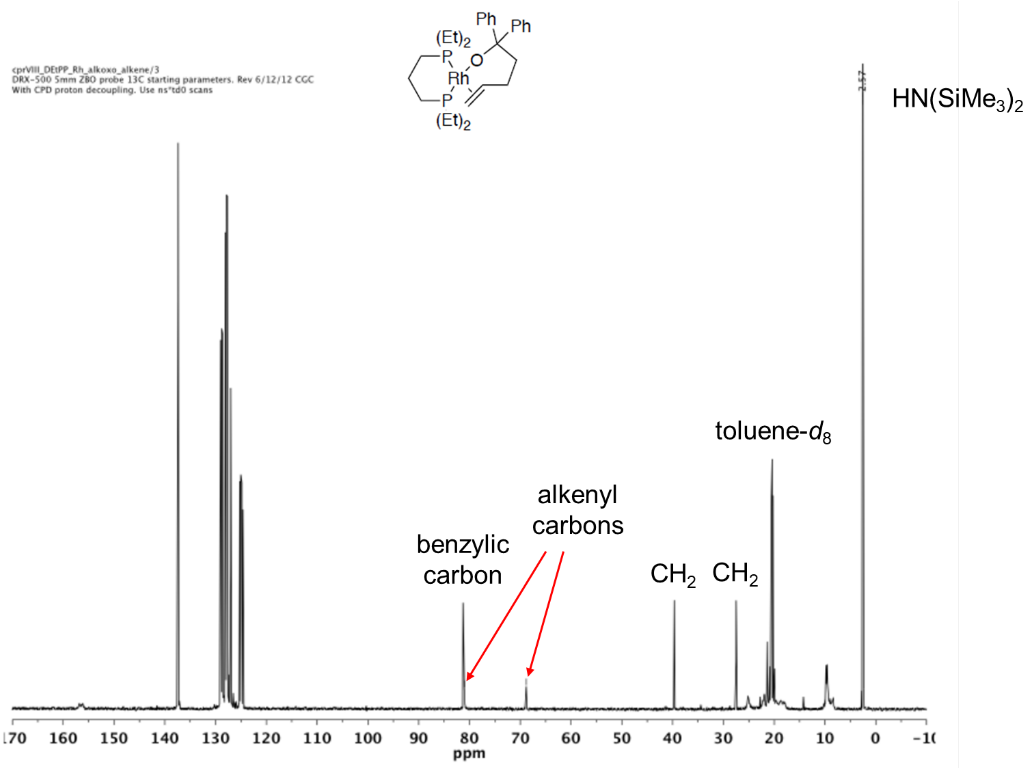

Figure S53. $^{31}P\{^1H\}$ NMR spectrum (202.2 MHz, THF-d_8) of **3e** at -40 °C.

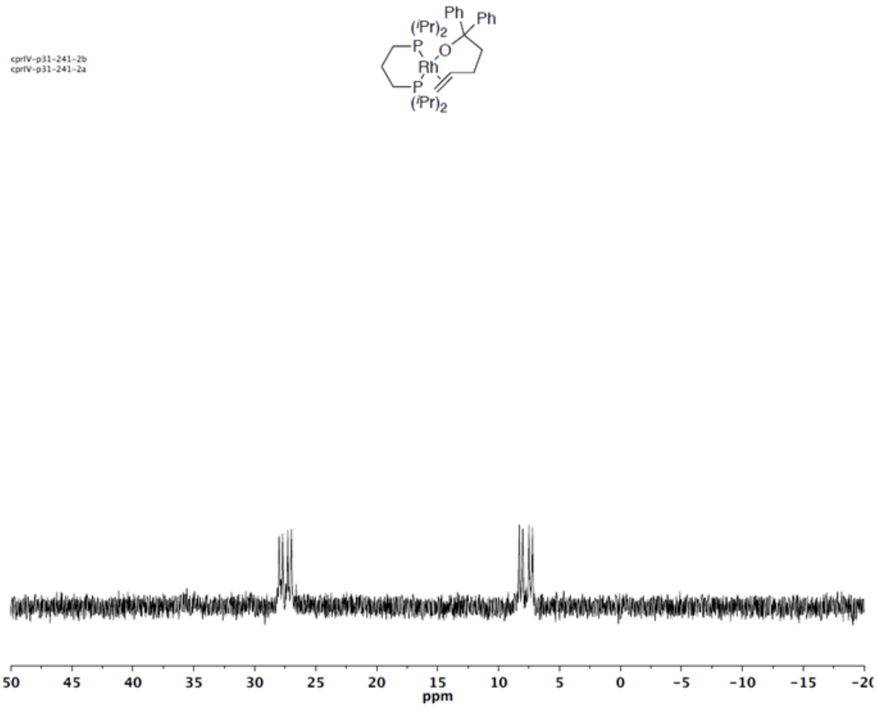

Figure S54. ^{1}H NMR spectrum (499.4 MHz, THF-d_8) of **3e** with 1,3,5-trimethoxybenzene (TMB) as internal standard at -40 °C.

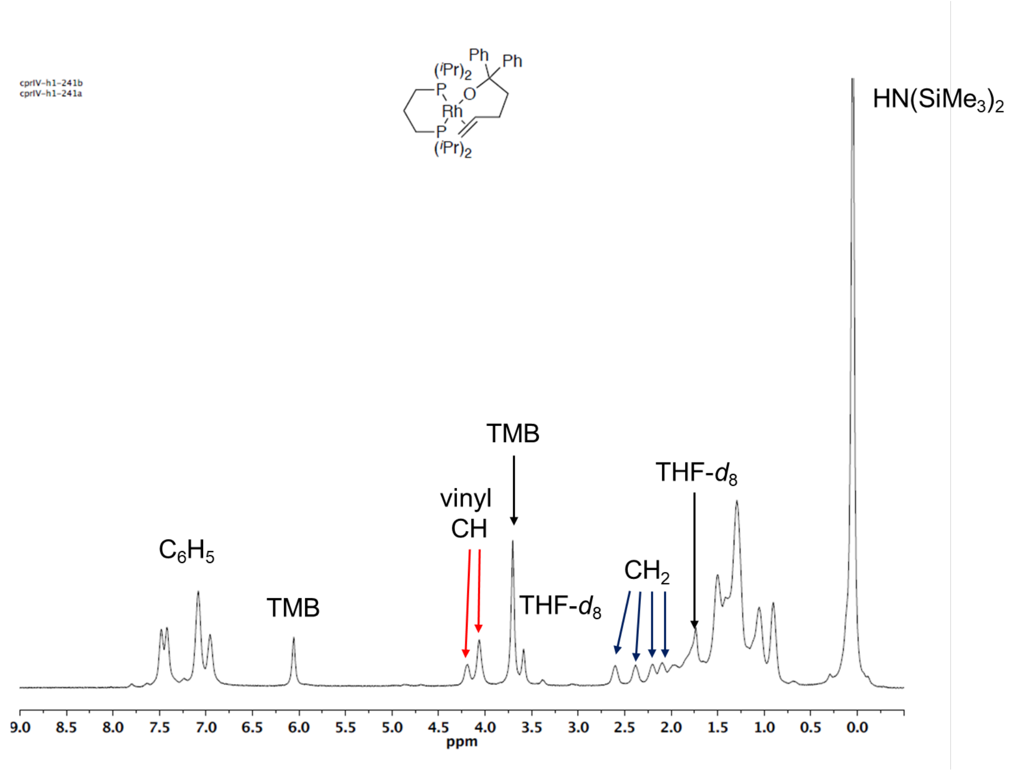

Figure S55. $^{13}C\{^{1}H\}$ NMR spectrum (125.7 MHz, toluene-d_8) of **3e** at -40 °C.

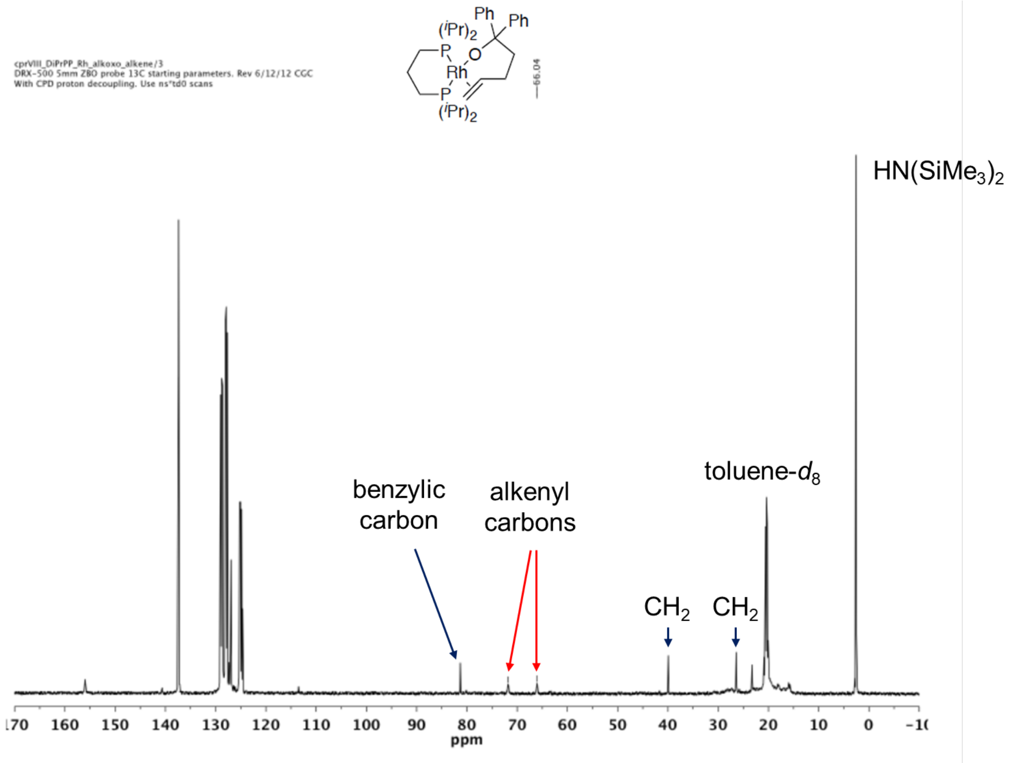

Figure S56. $^{31}P\{^{1}H\}$ NMR spectrum (202.2 MHz, toluene-d_8) of **3f** at -40 °C.

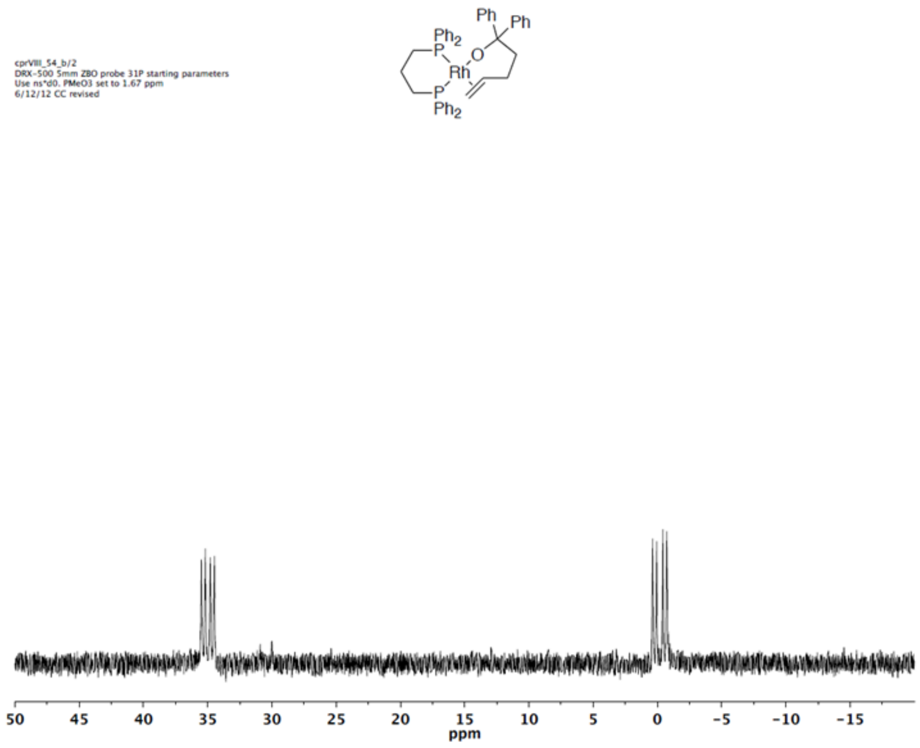

Figure S57. ^{1}H NMR spectrum (499.4 MHz, toluene-d_8) of **3f** at -40 °C.

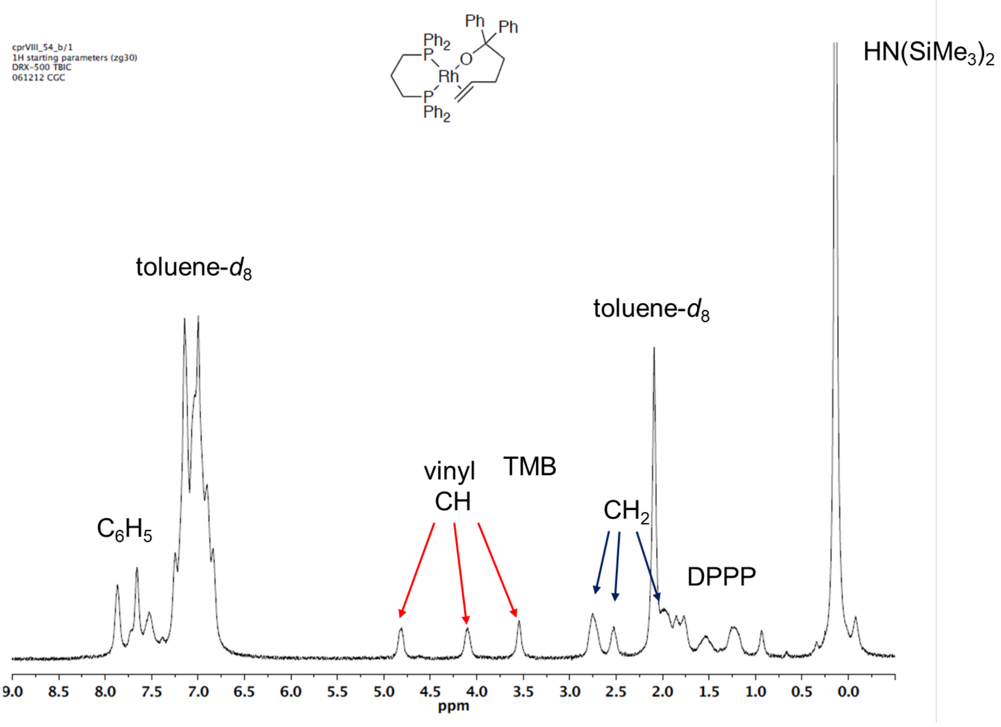

Figure S58. $^{13}C\{^1H\}$ NMR spectrum (125.7 MHz, THF-d_8) of **3f** at -40 °C.

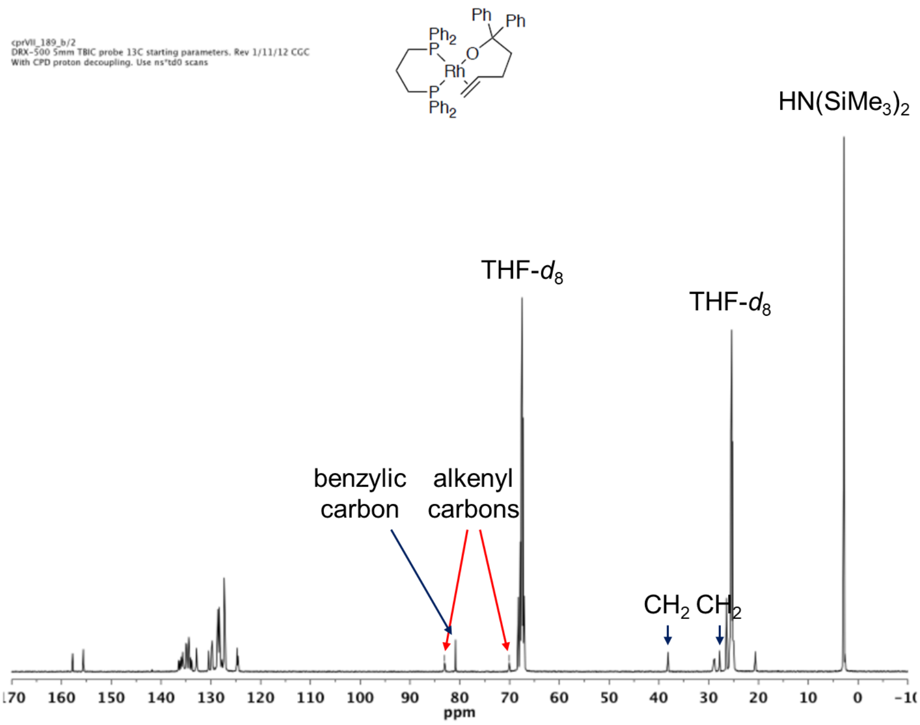

Figure S59. $^{31}P\{^1H\}$ NMR spectrum (202.2 MHz, toluene-d_8) of **3g** at -40 °C.

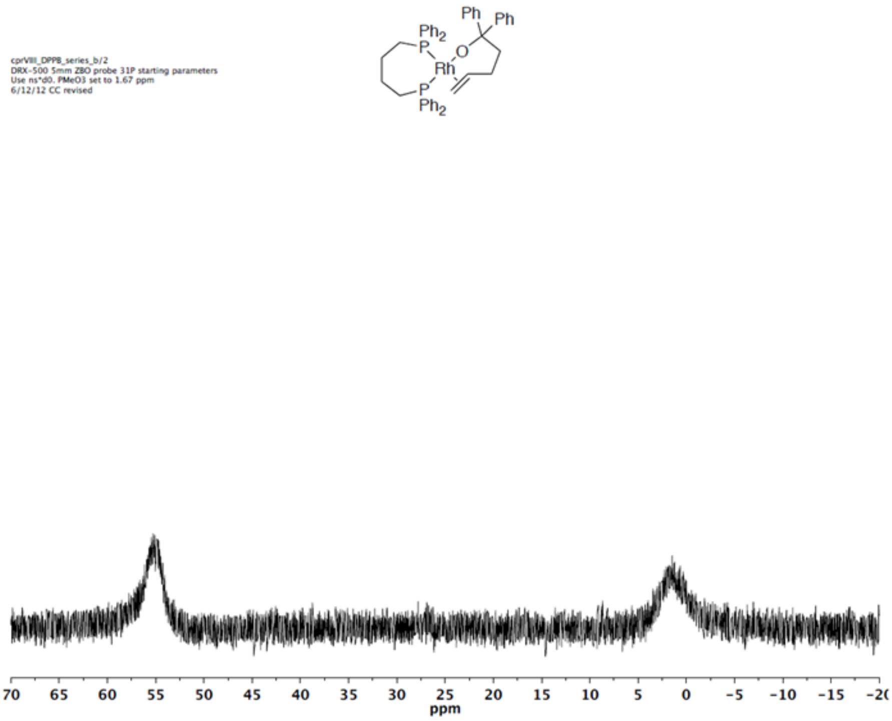

Figure S60. ^{1}H NMR spectrum (499.4 MHz, toluene-d_8) of **3g** with 1,3,5-trimethoxybenzene (TMB) as internal standard at -40 °C.

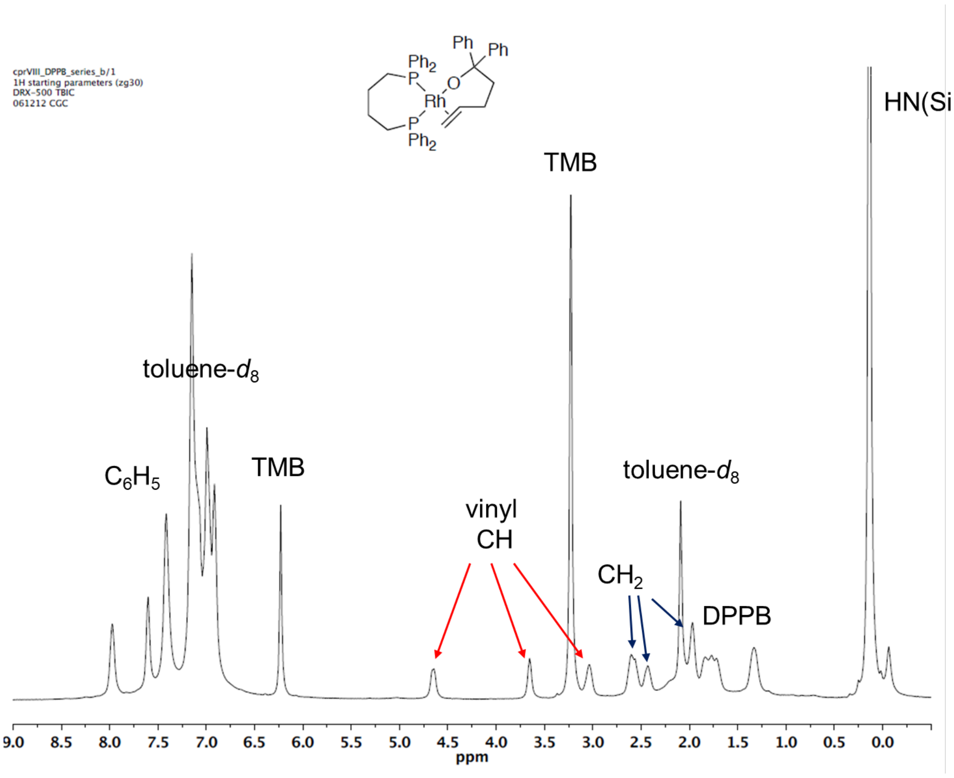

Figure S61. ^{13}C{^{1}H} NMR spectrum (125.7 MHz, THF-d_8) of **3g** at -40 °C.

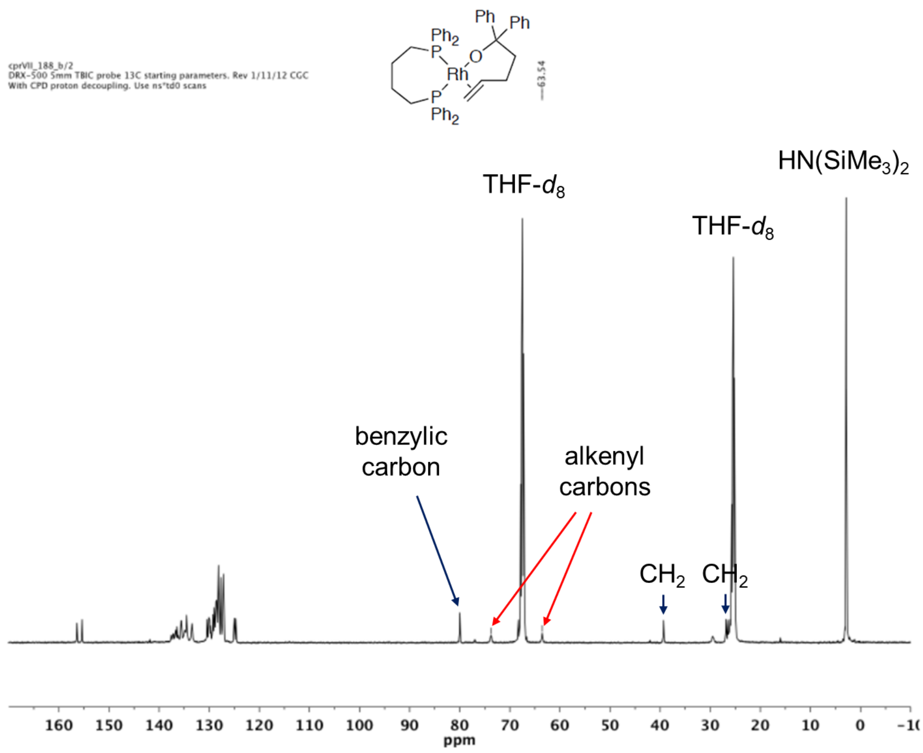

Figure S62. $^{31}P\{^{1}H\}$ NMR spectrum (202.2 MHz, toluene-d_8) of **3h** at -40 °C.

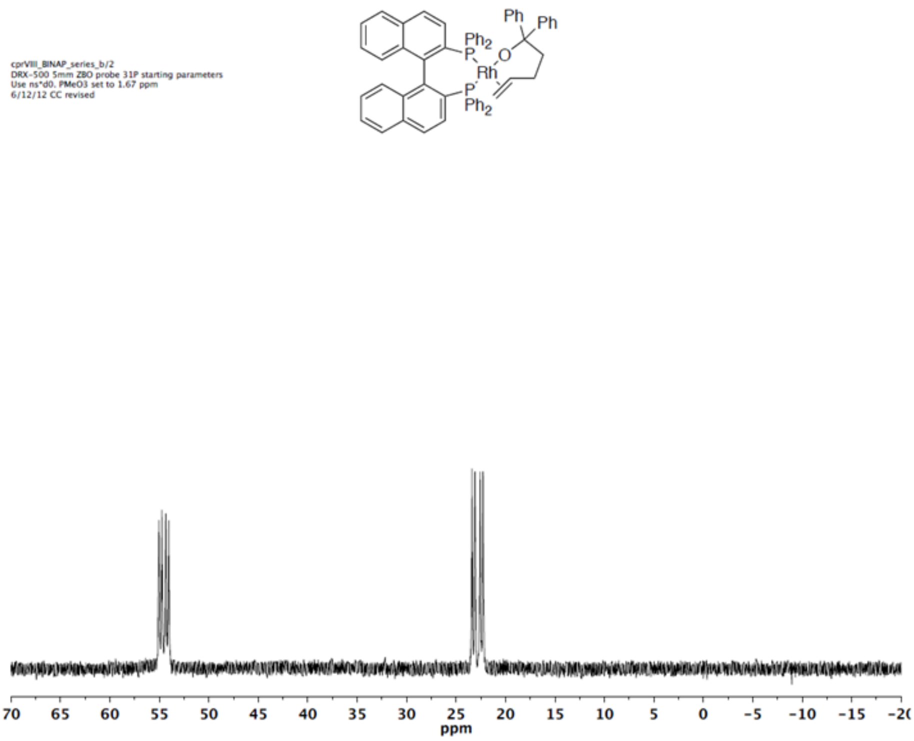

Figure S63. ^{1}H NMR spectrum (499.4 MHz, toluene-d_8) of **3h** with 1,3,5-trimethoxybenzene (TMB) as internal standard at -40 °C.

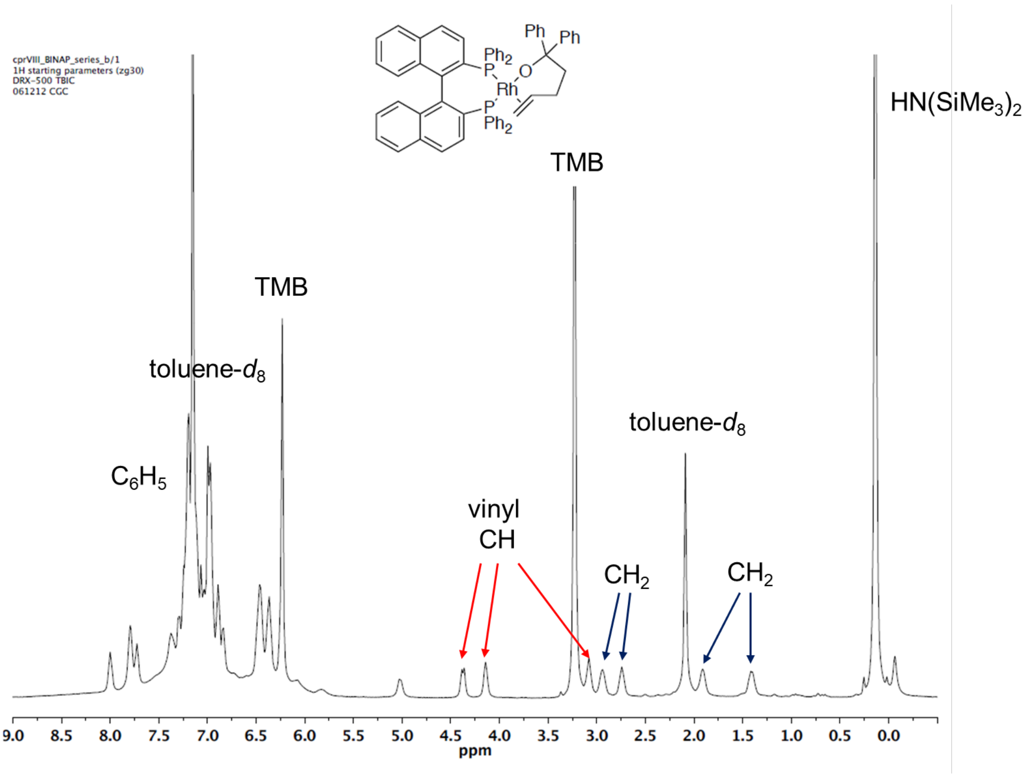

Figure S64. $^{13}C\{^1H\}$ NMR spectrum (125.7 MHz, THF-d_8) of **3h** at -40 °C.

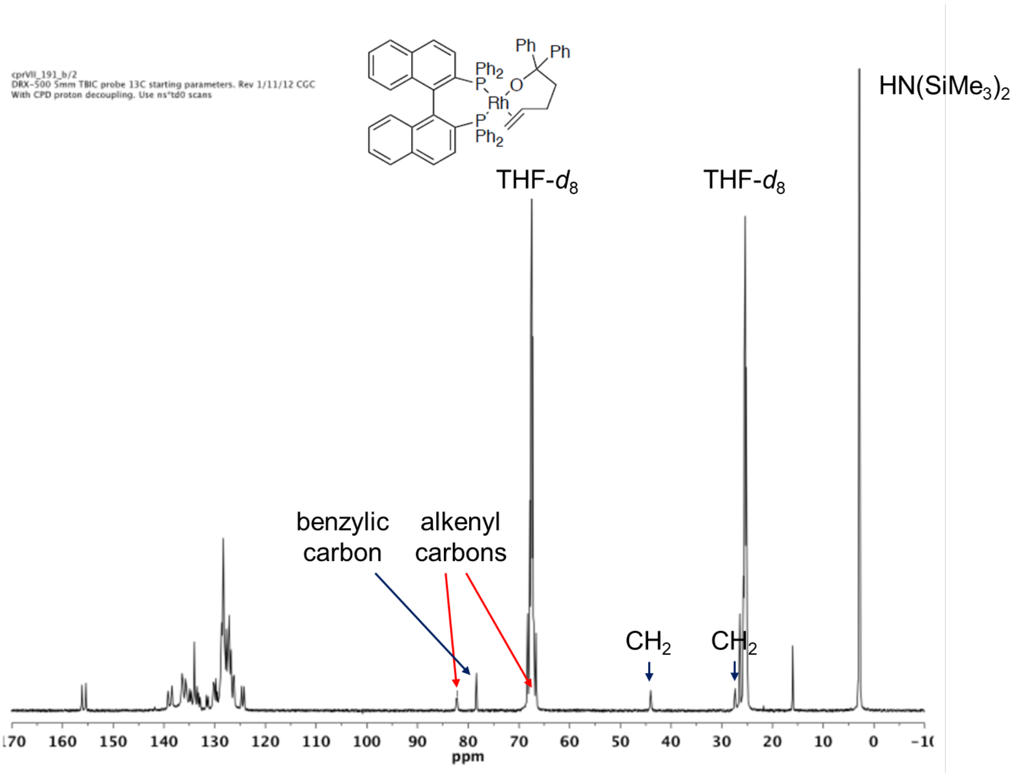

Figure S65. $^{31}P\{^1H\}$ NMR spectrum (202.2 MHz, THF-d_8) of **3i** at -40 °C.

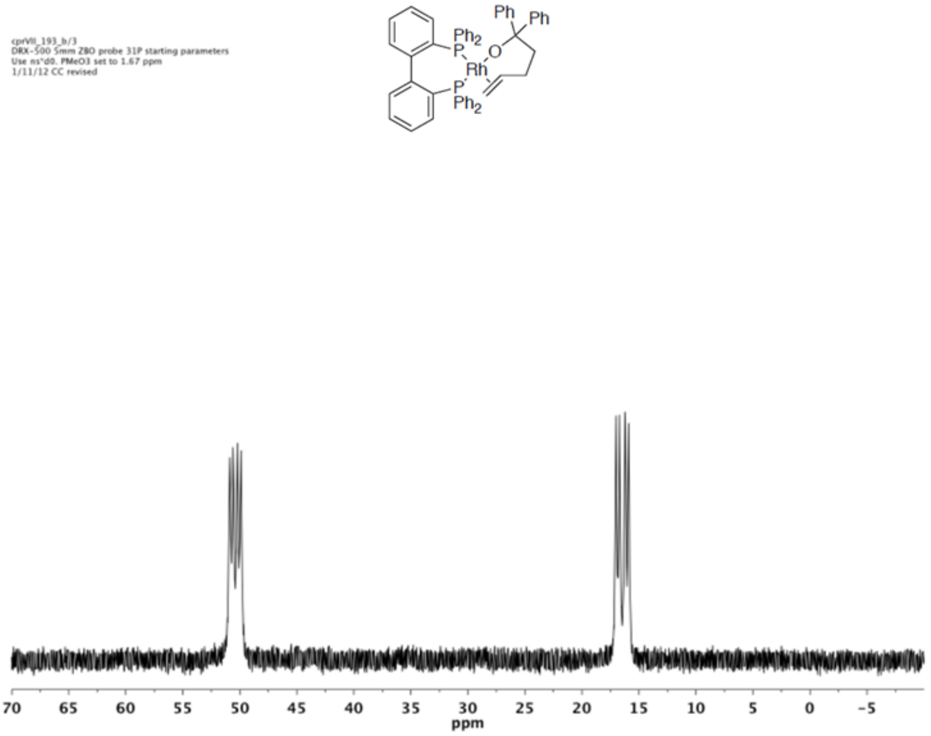

Figure S66. ^{1}H NMR spectrum (499.4 MHz, THF-d_8) of **3i** with 1,3,5-trimethoxybenzene (TMB) as internal standard at -40 °C.

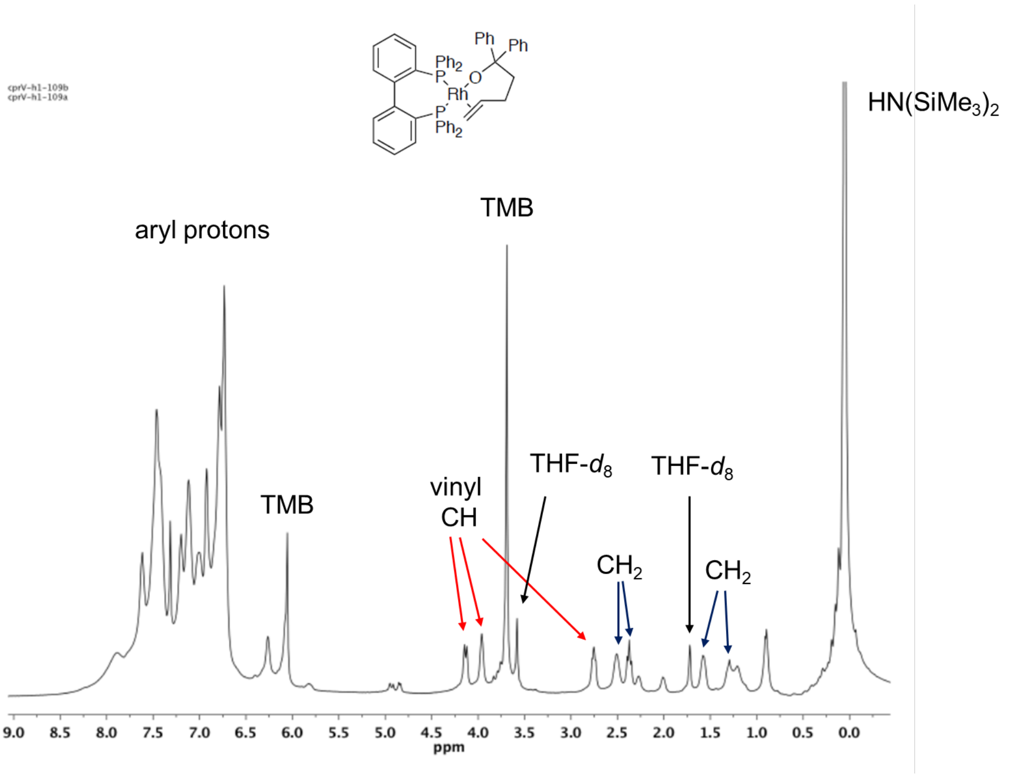

Figure S67. ^{13}C{^{1}H} NMR spectrum (125.7 MHz, THF-d_8) of **3i** at -40 °C.

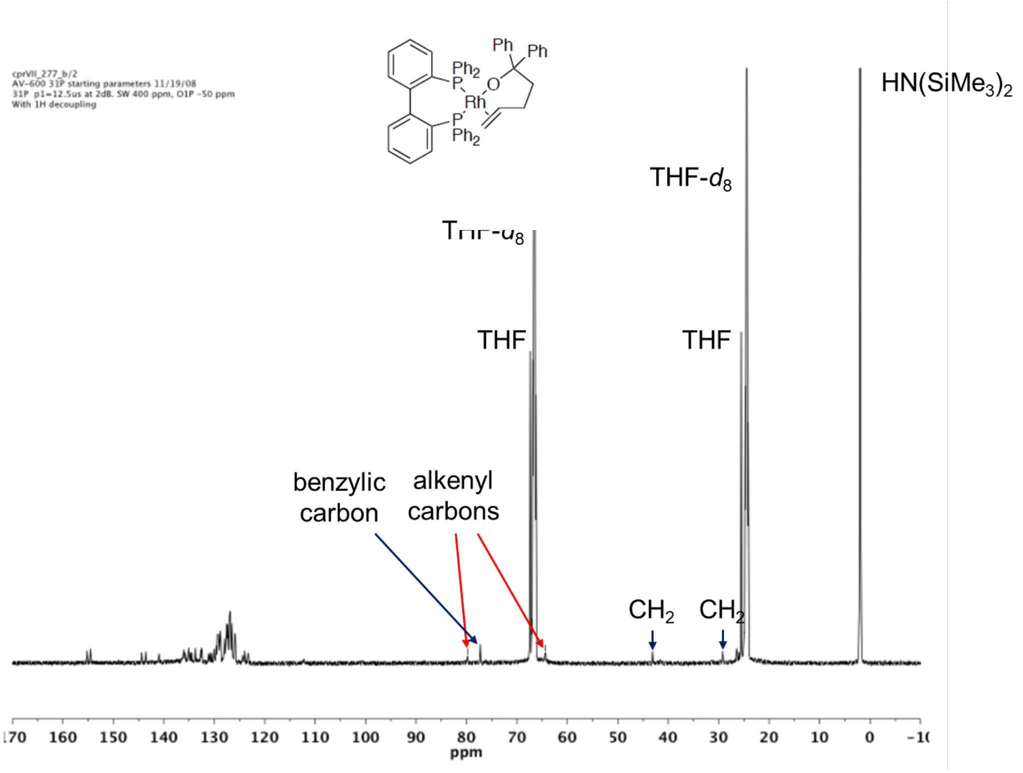

Figure S68. $^{31}P\{^{1}H\}$ NMR spectrum (202.2 MHz, THF-d_8) of **3j** at -40 °C.

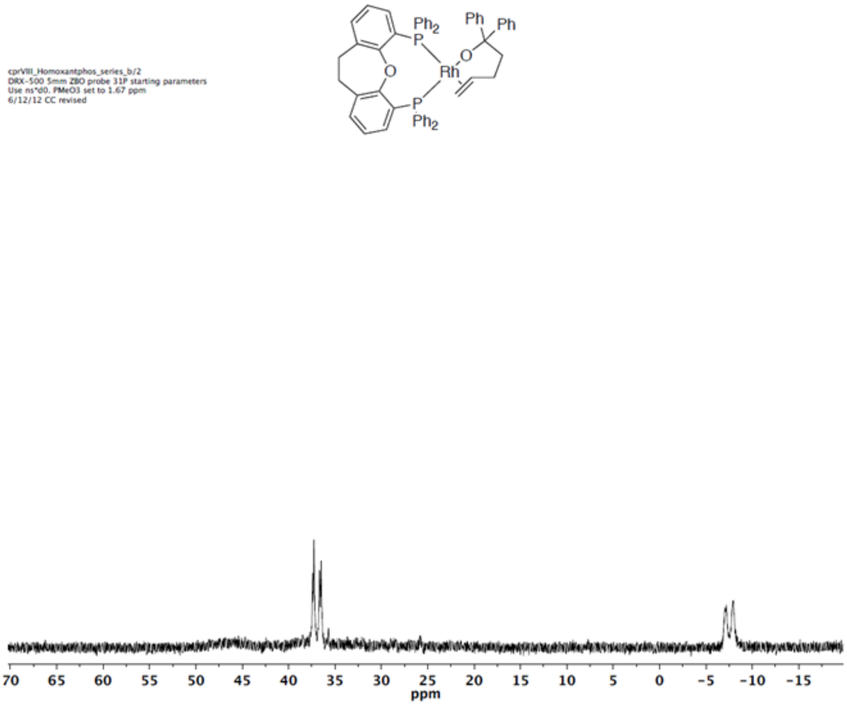

Figure S69. ^{1}H NMR spectrum (499.4 MHz, THF-d_8) of **3j** with 1,3,5-trimethoxybenzene (TMB) as internal standard at -40 °C.

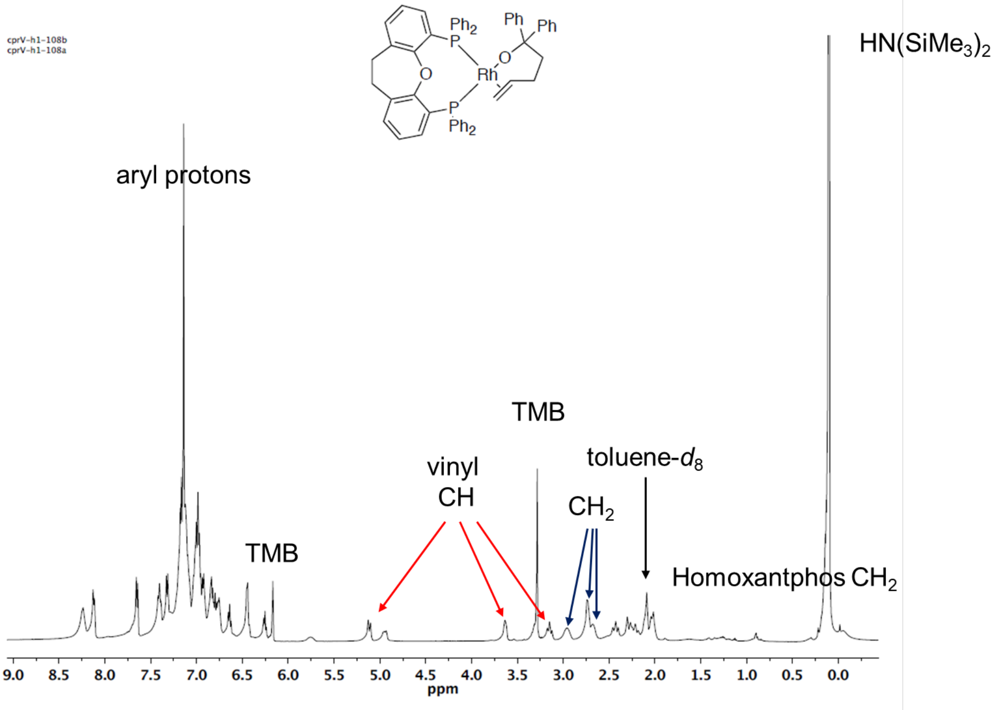

Figure S70. $^{13}C\{^1H\}$ NMR spectrum (125.7 MHz, TFH-d_8) of **3j** at -40 °C.

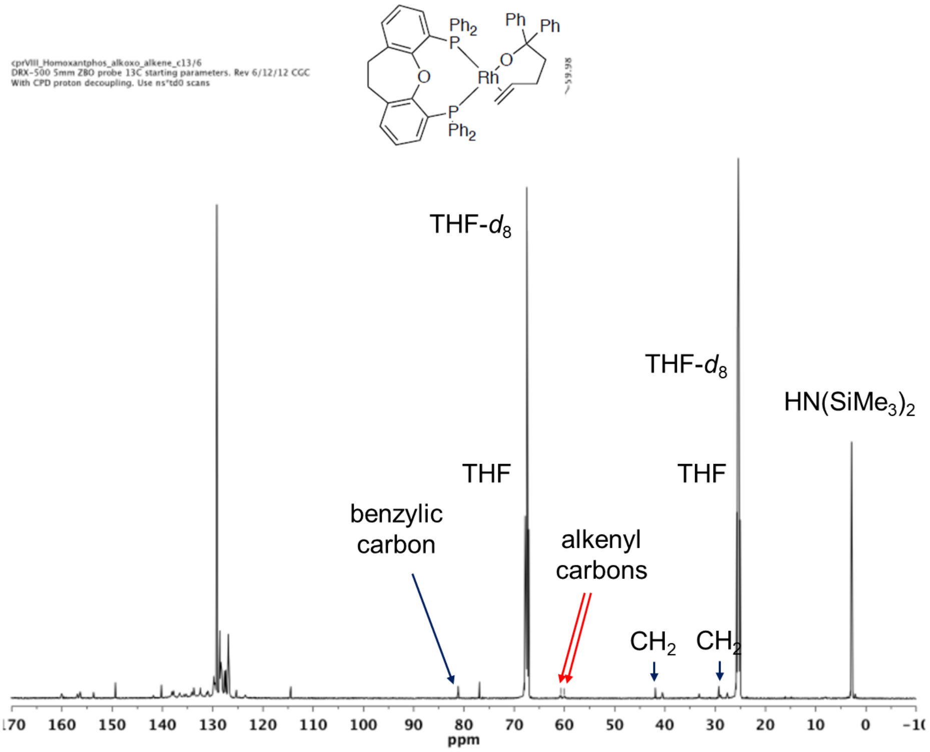

Figure S71. $^{31}P\{^1H\}$ NMR spectrum (202.2 MHz, toluene-d_8) of **3k** at -40 °C.

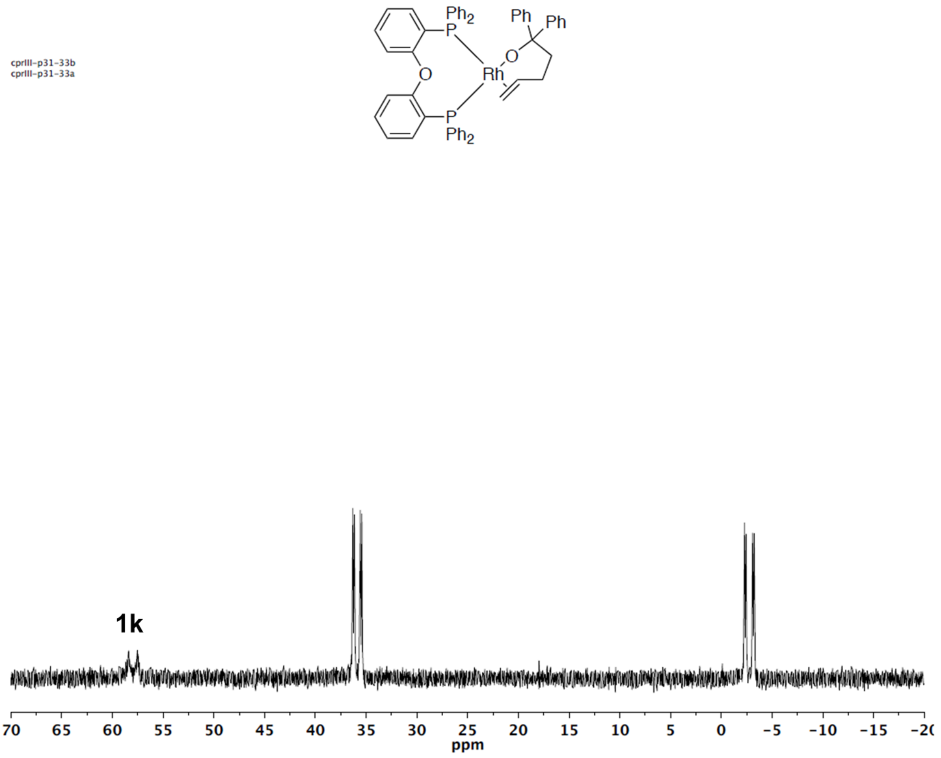

Figure S72. ^{1}H NMR spectrum (499.4 MHz, toluene-d_8) of **3k** with 1,3,5-trimethoxybenzene (TMB) as internal standard at -40 °C.

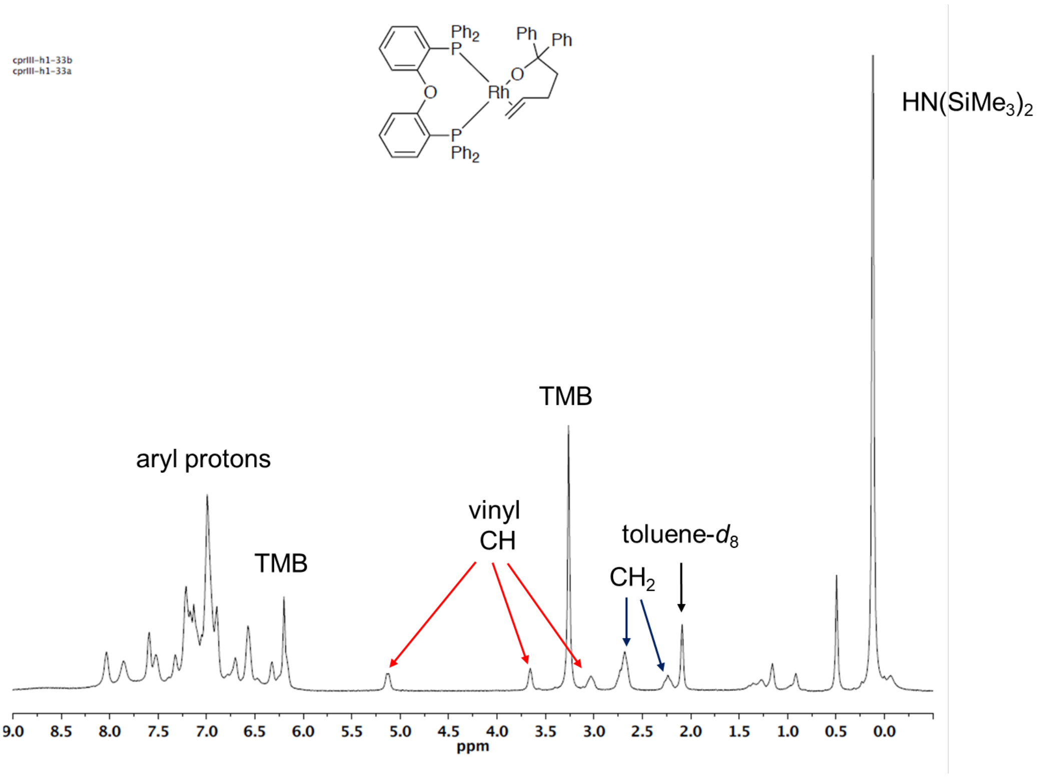

Figure S73. $^{31}P\{^{1}H\}$ NMR spectrum (202.2 MHz, toluene-d_8) of **3l** at -40 °C.

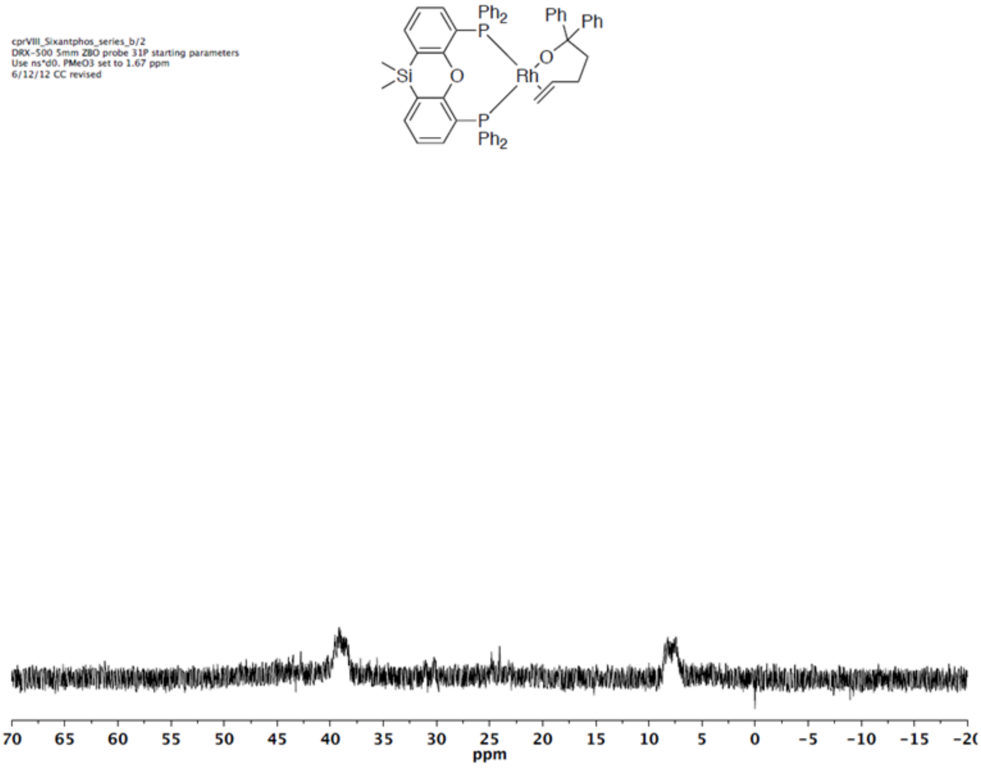

Figure S74. ^{1}H NMR spectrum (499.4 MHz, toluene-d_8) of **3l** with 1,3,5-trimethoxybenzene (TMB) as internal standard at -40 °C.

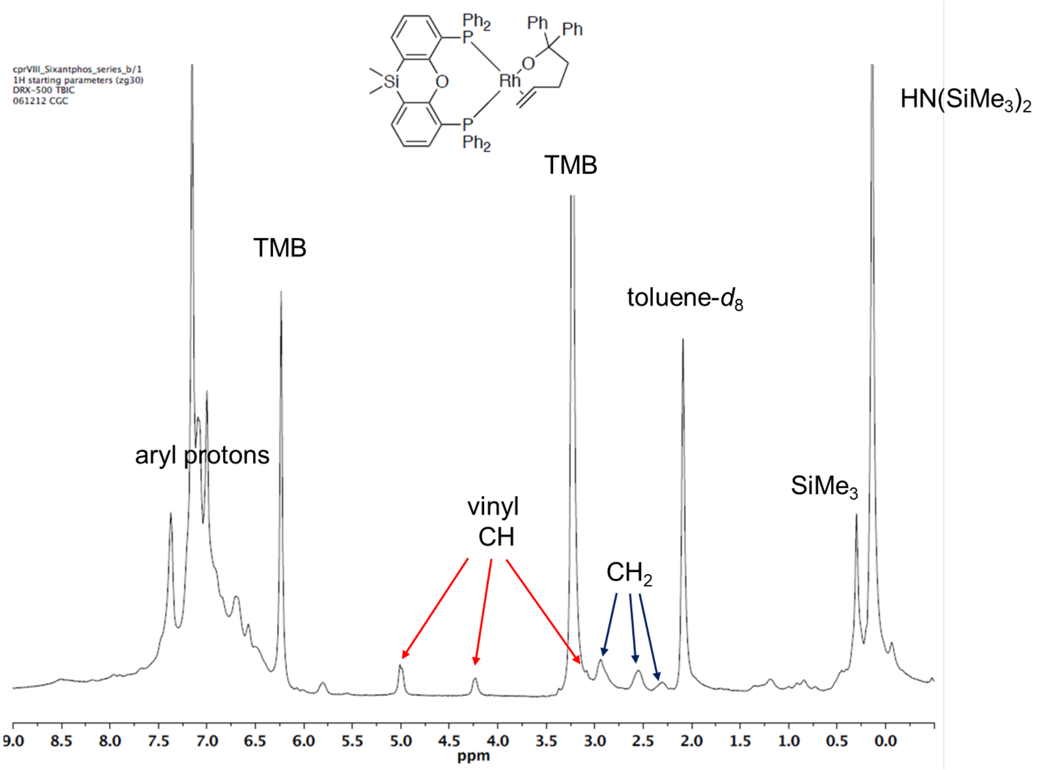

Figure S75. $^{31}P\{^{1}H\}$ NMR spectrum (202.2 MHz, toluene-d_8) of **3m** at -40 °C.

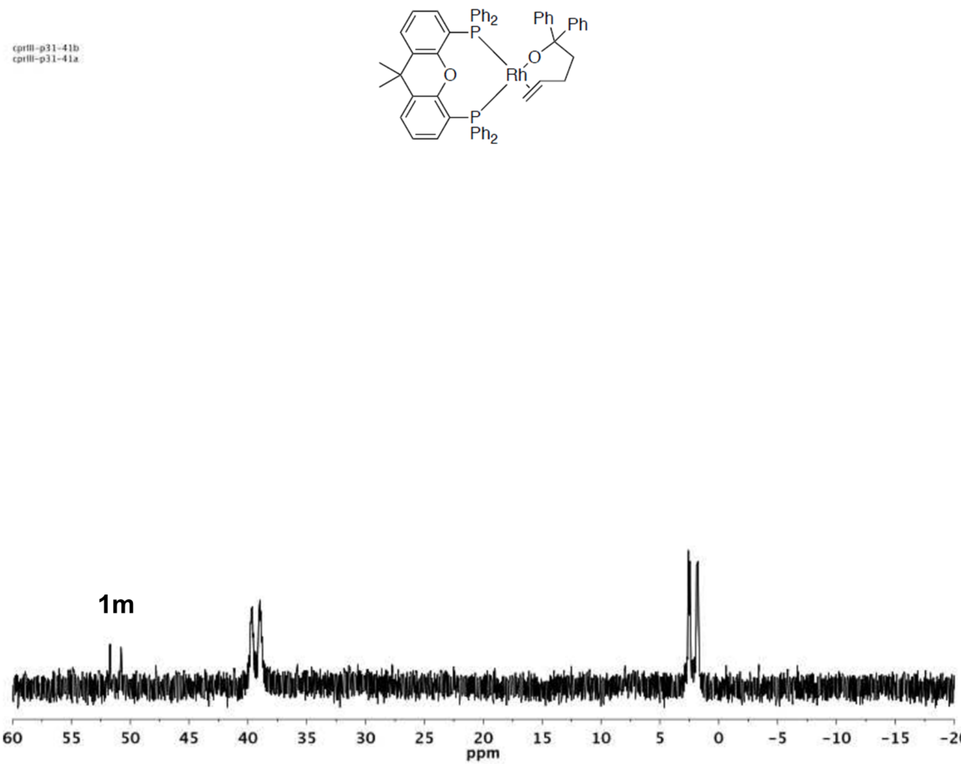

Figure S76. ^{1}H NMR spectrum (499.4 MHz, toluene-d_8) of **3m** with 1,3,5-trimethoxybenzene (TMB) as internal standard at -40 °C.

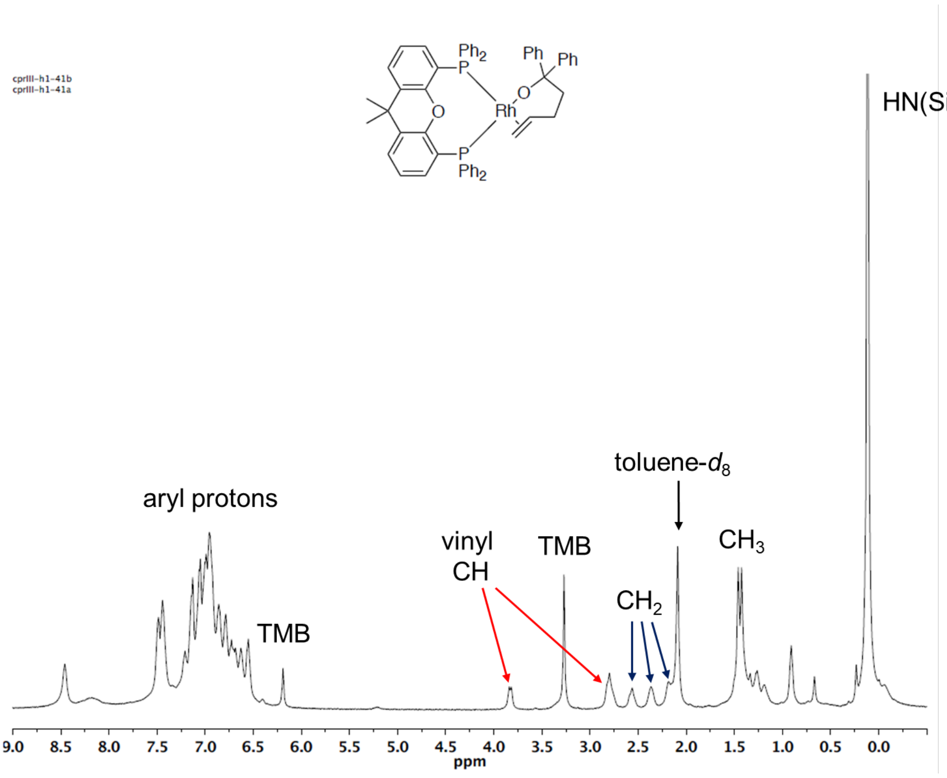

Figure S77. ^{1}H NMR spectrum (499.4 MHz, toluene-d_8) of **5f** at 25 °C.

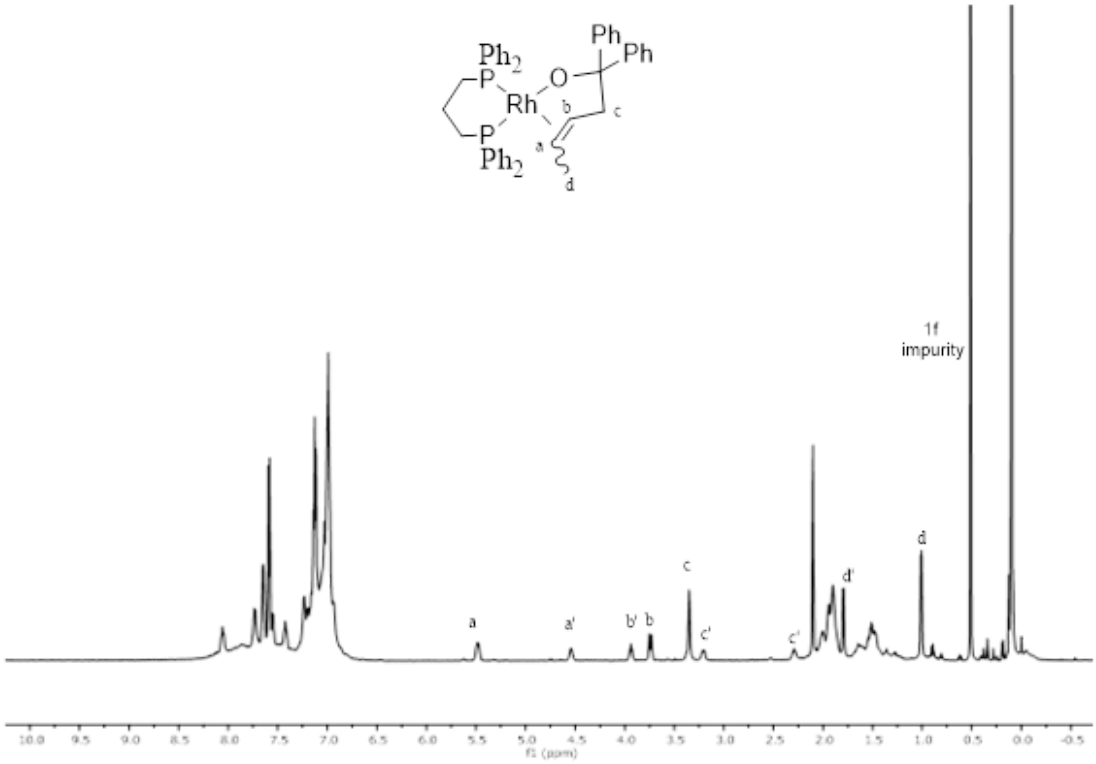

Figure S78. $^{31}P\{^1H\}$ NMR spectrum (202.2 MHz, toluene-d_8) of **5f** at 25 °C.

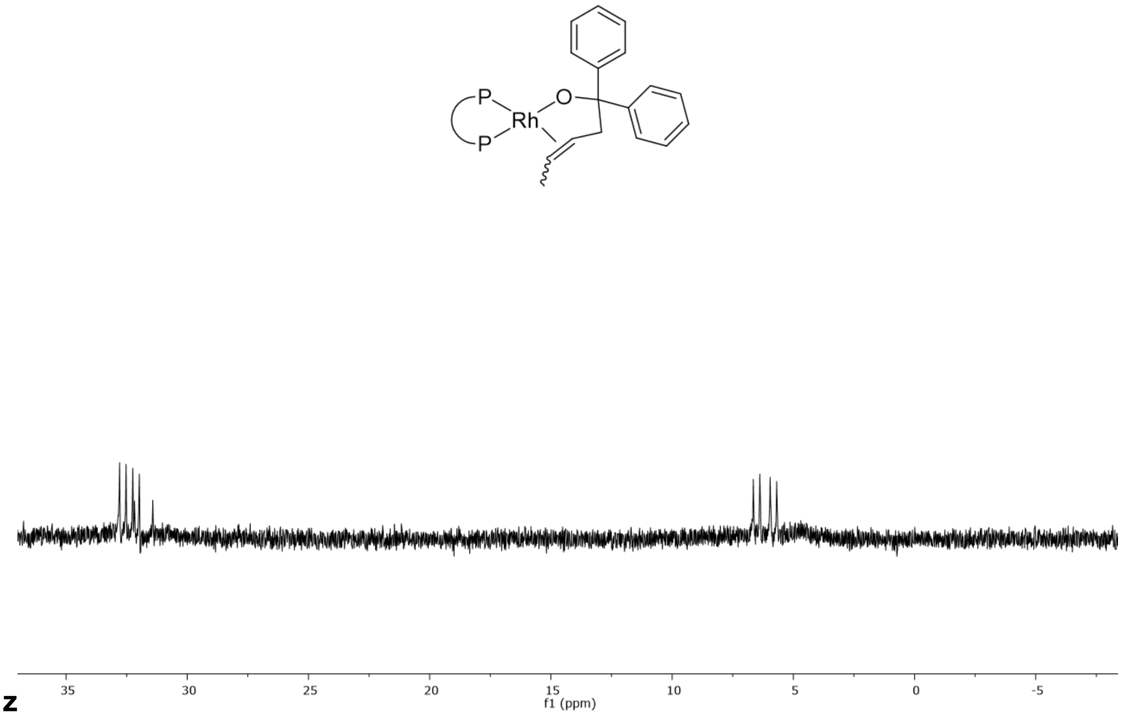

Figure S79. ^1H NMR spectrum (400 MHz, $CDCl_3$) of methylene furan product, **4a**, at 25 °C.

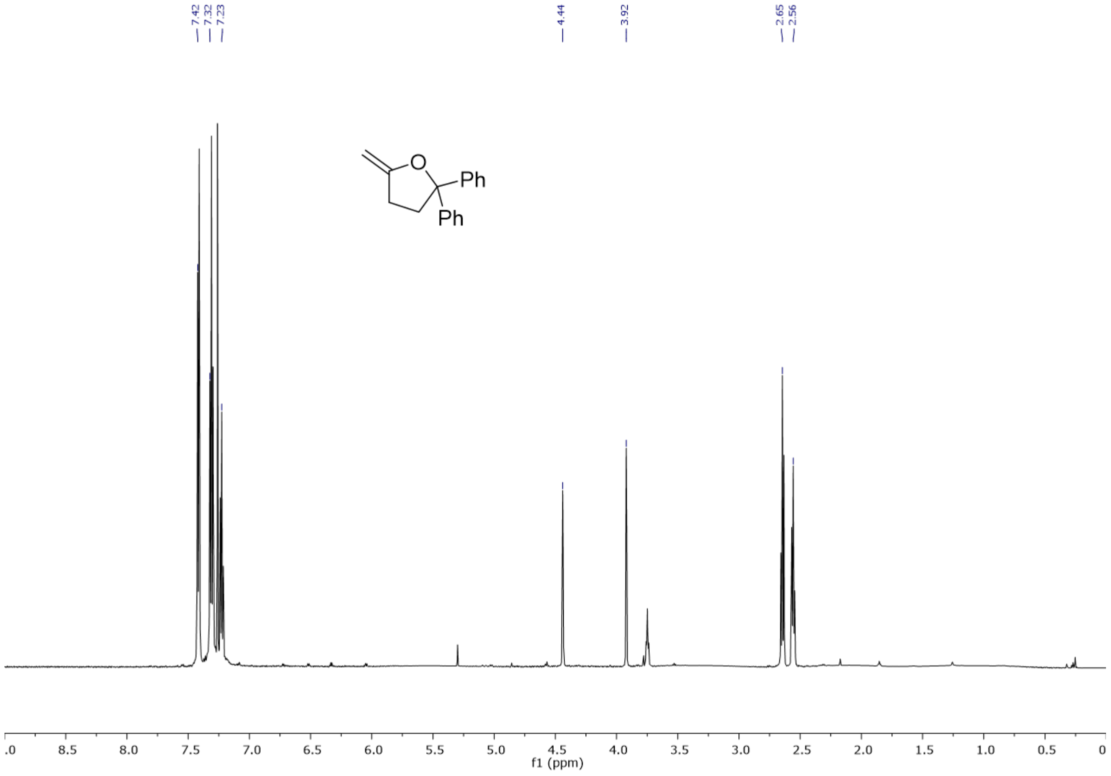

Figure S80. $^{13}C\{^1H\}$ NMR spectrum (101 MHz, $CDCl_3$) of methylene furan product, **4a**.

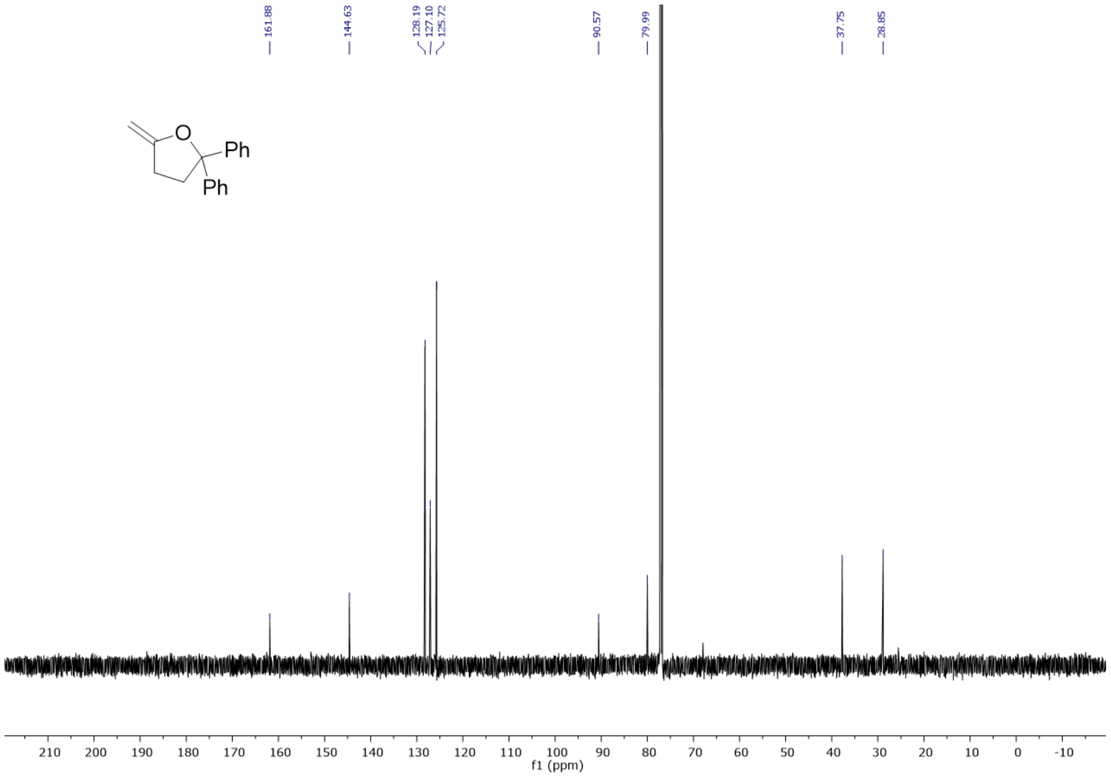

Figure S81. ^{1}H NMR spectrum (400 MHz, $CDCl_3$) of ^{13}C-labelled alcohol **2-^{13}C**.

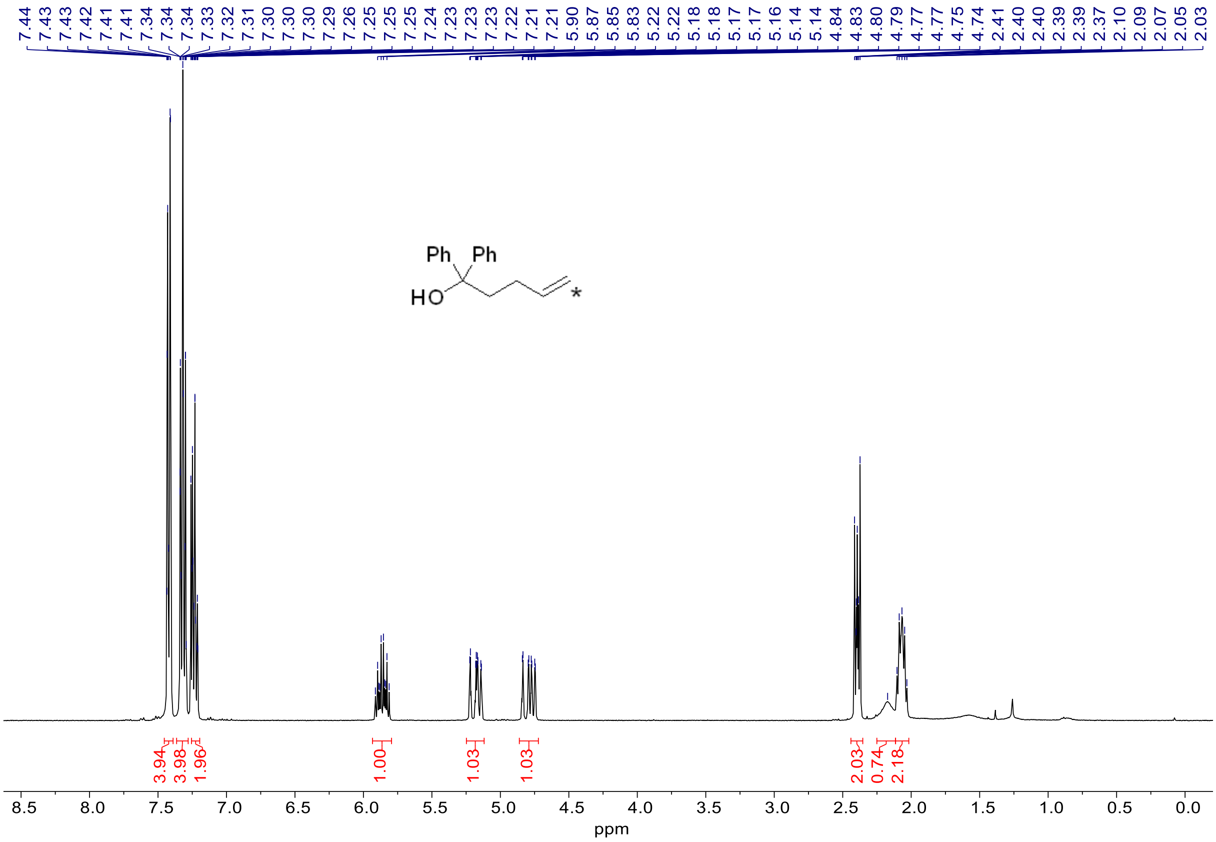

Figure S82. $^{13}C\{^{1}H\}$ NMR spectrum (101 MHz, $CDCl_3$) of ^{13}C-labelled alcohol **2-^{13}C**.

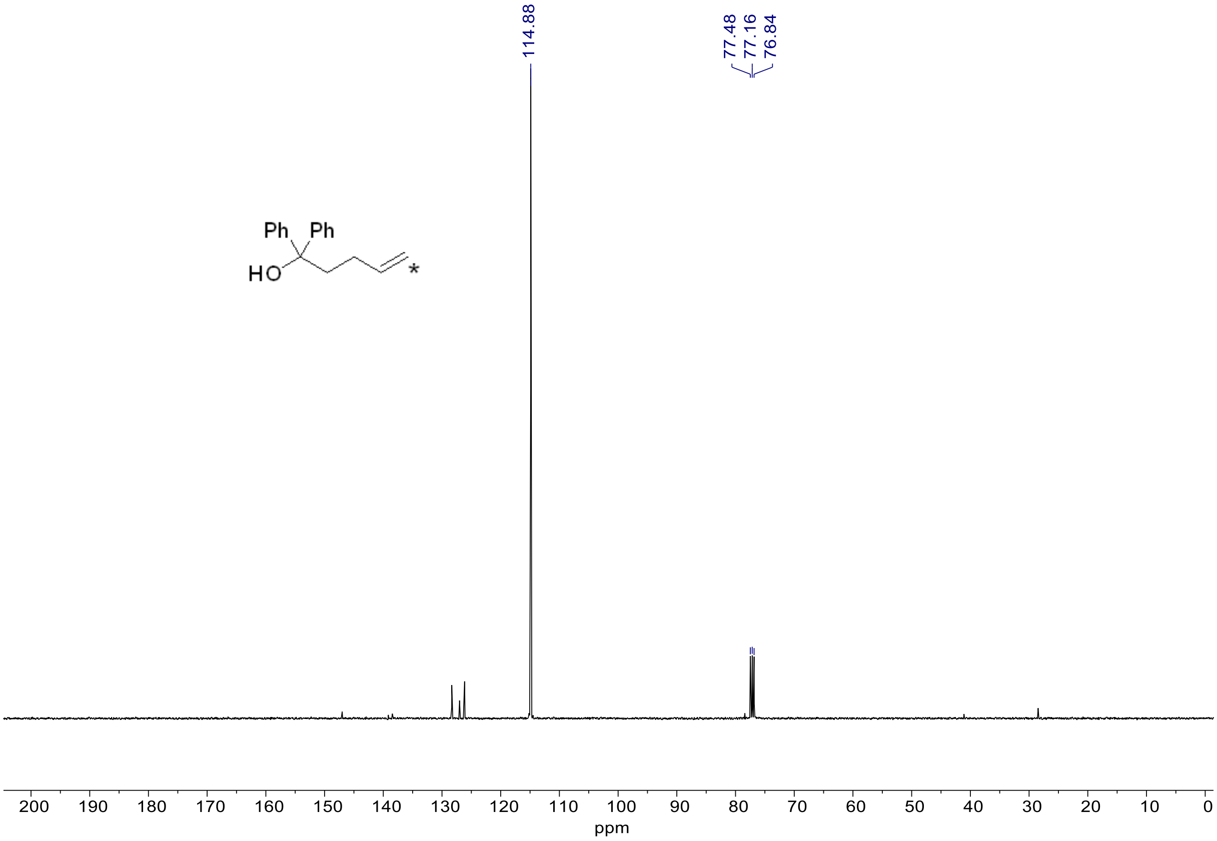

Figure S83. ^{1}H NMR spectrum (500 MHz, $CDCl_3$) of fluorinated alcohol **S1**.

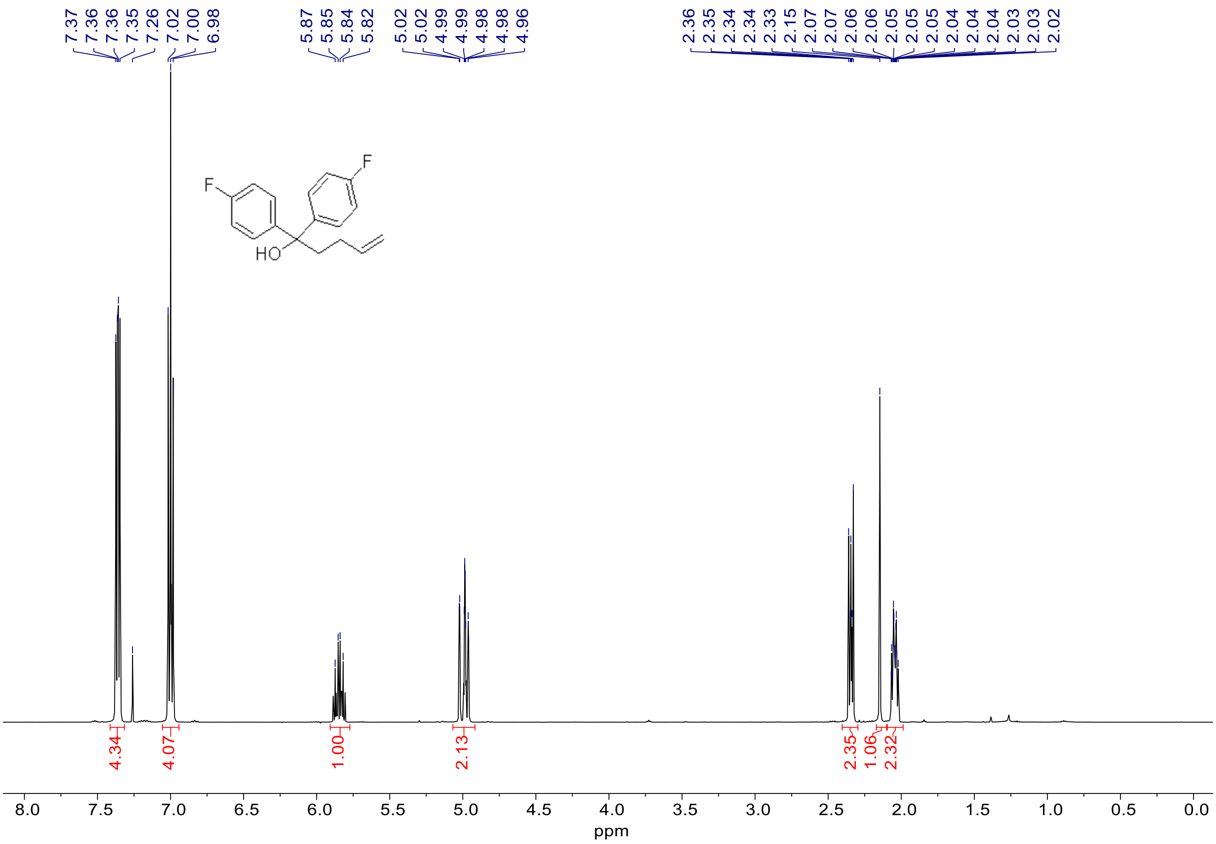

Figure S84. $^{13}C\{^{1}H\}$ NMR spectrum (126 MHz, $CDCl_3$) of fluorinated alcohol **S1**.

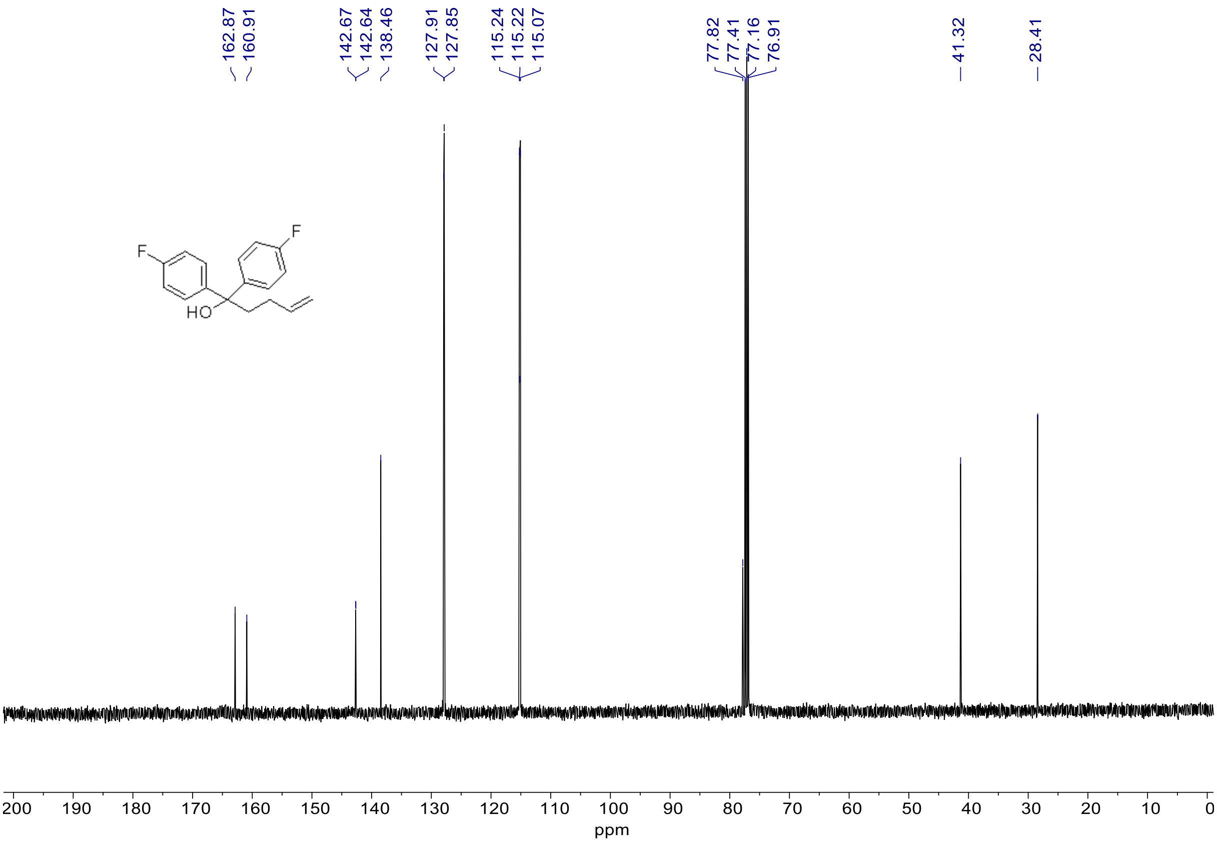

Figure S85. $^{19}F\{^{1}H\}$ NMR spectrum (470 MHz, $CDCl_3$) of fluorinated alcohol **S1**.

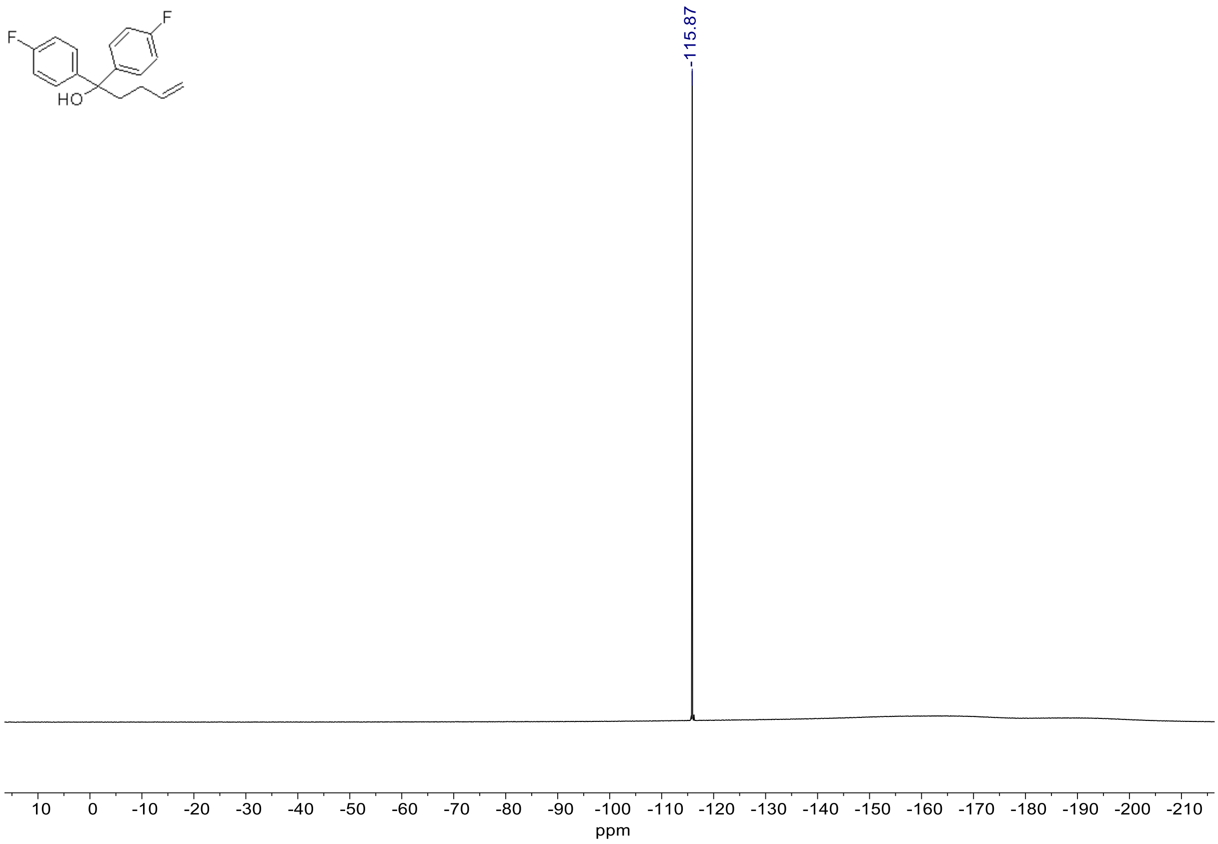

Figure S86. ^{1}H NMR spectrum (500 MHz, $CDCl_3$) of deuterated alcohol **2-*d*₁₀**.

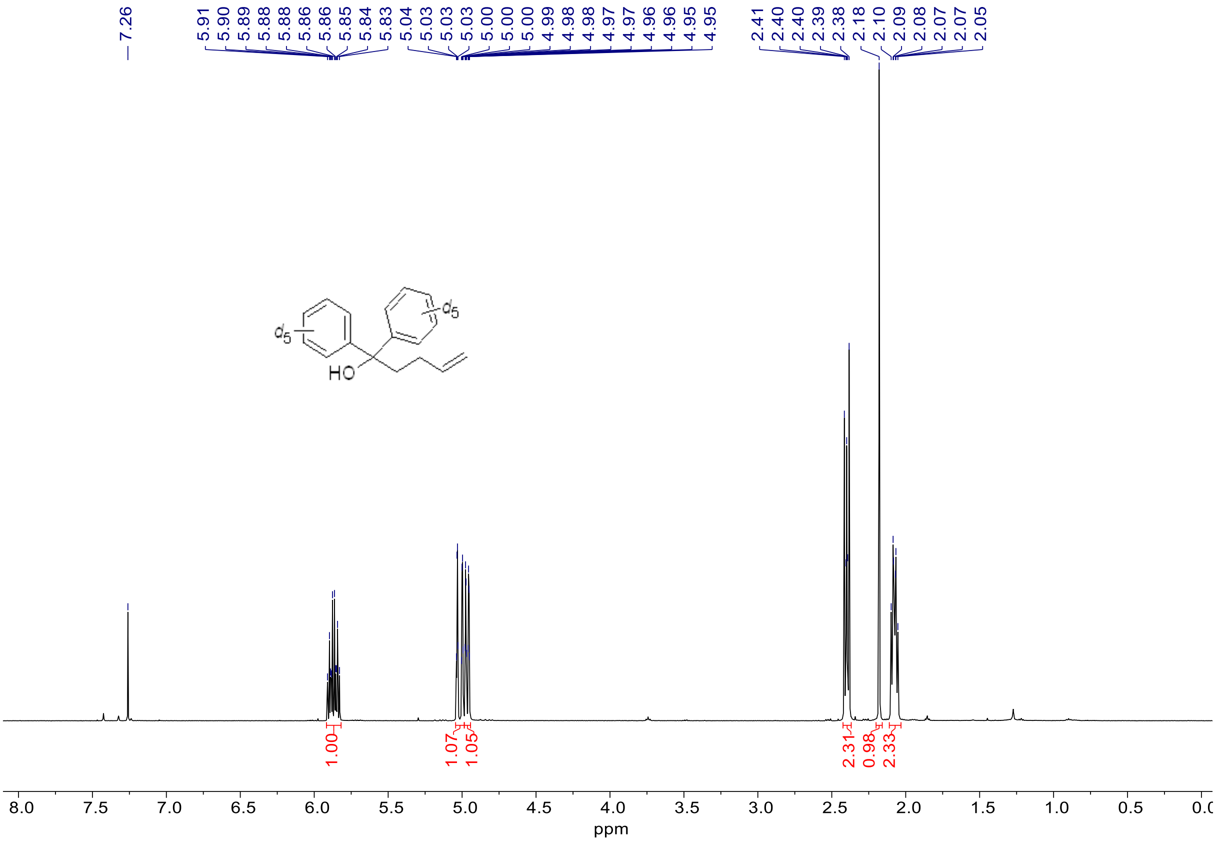

Figure S87. ^{2}H NMR spectrum (77 MHz, toluene) of deuterated alcohol **2-*d*₁₀**.

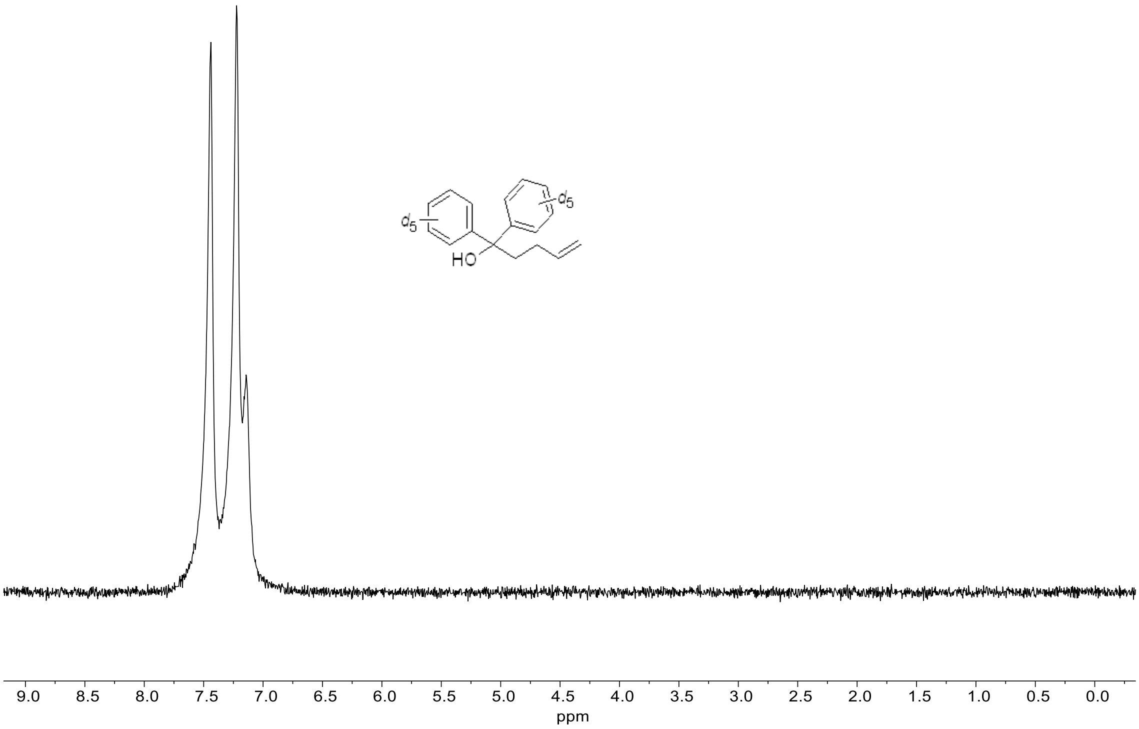

Figure S88. $^{13}C\{^1H\}$ NMR spectrum (151 MHz, $CDCl_3$) of deuterated alcohol **2-*d*$_{10}$**.

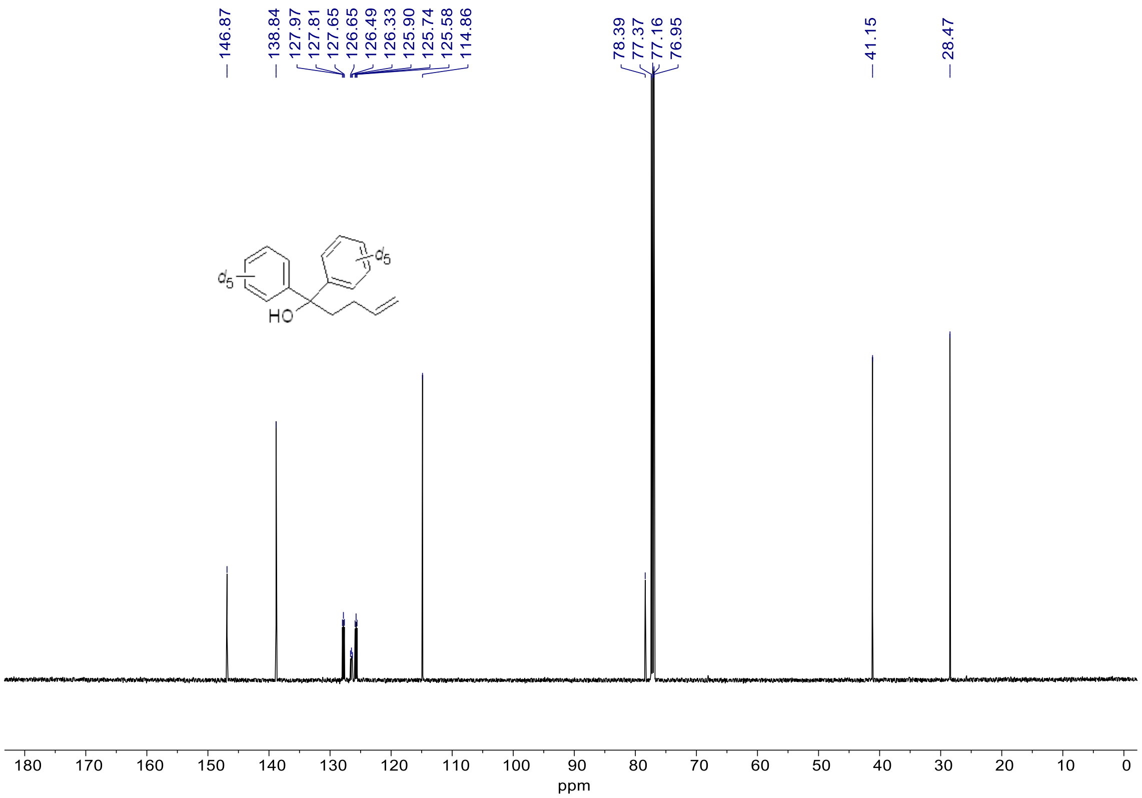

Figure S89. ^{1}H NMR spectrum (500 MHz, $CDCl_3$) of (*Z*)-1,1-diphenylpent-2-ene-1,4-diol.

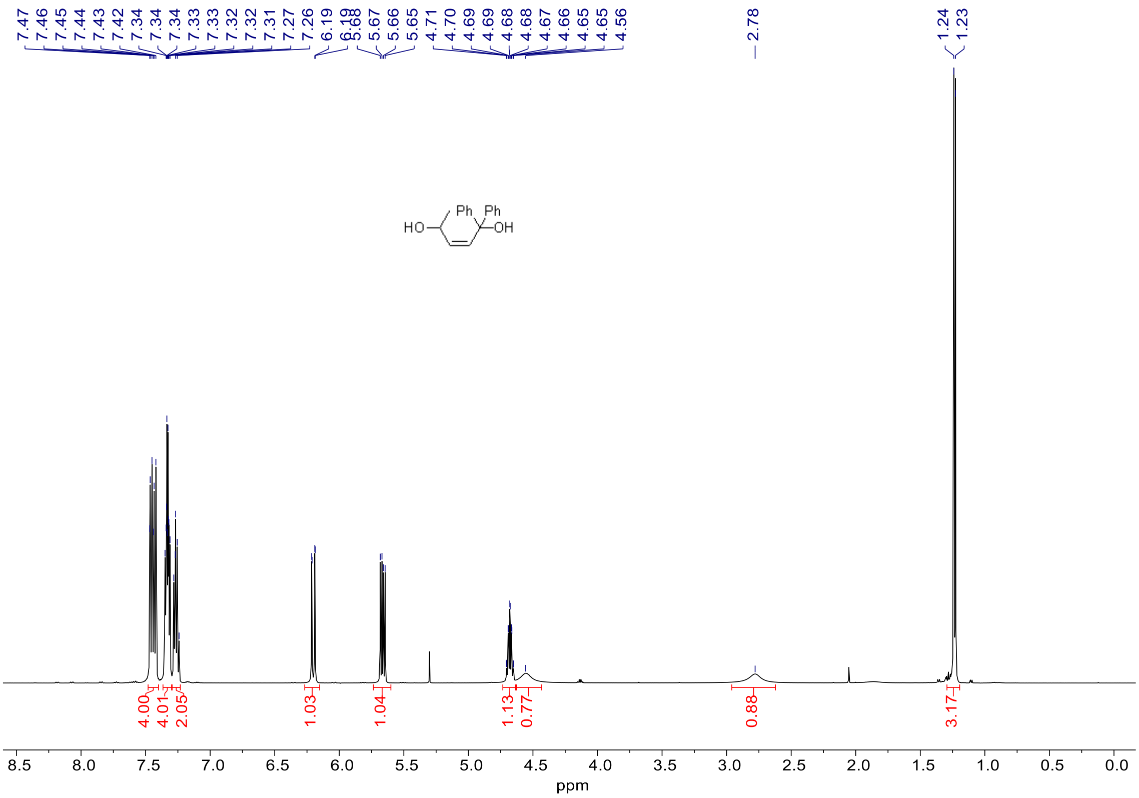

Figure S90. $^{13}C\{^{1}H\}$ NMR spectrum (126 MHz, $CDCl_3$) of (*Z*)-1,1-diphenylpent-2-ene-1,4-diol.

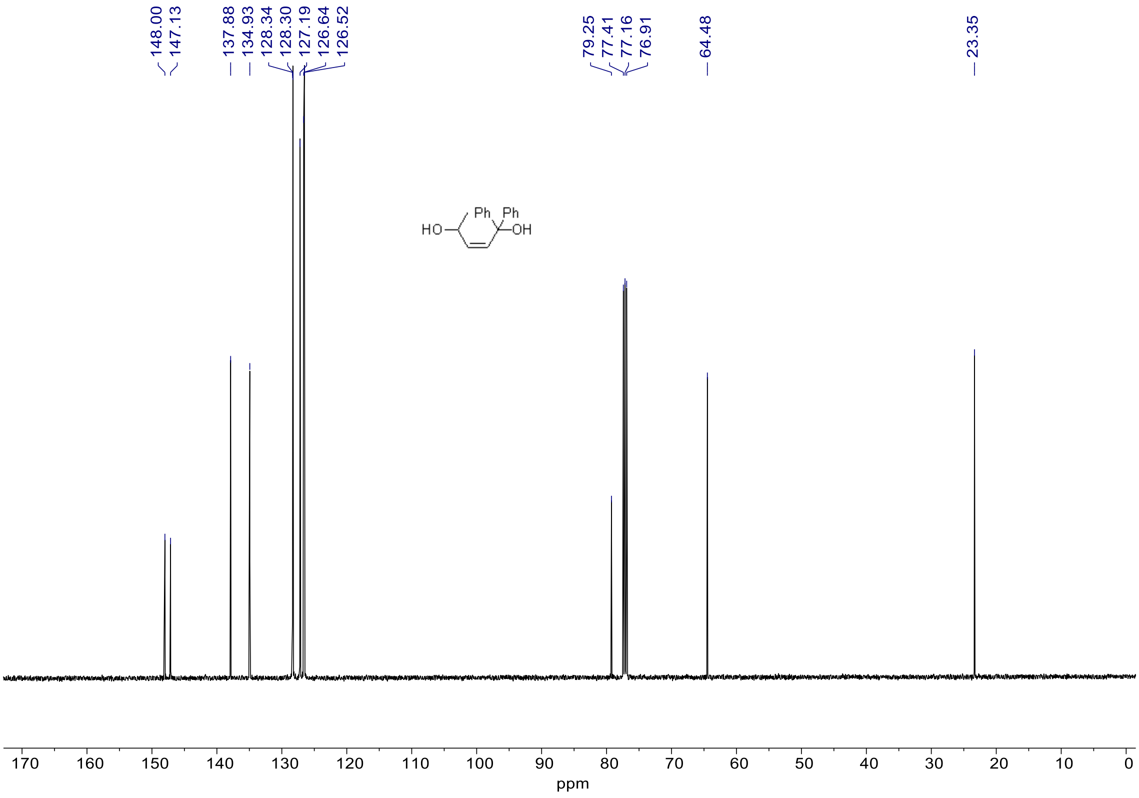

Figure S91. ^{1}H NMR spectrum (500 MHz, $CDCl_3$) of dihydrofuran **4c**.

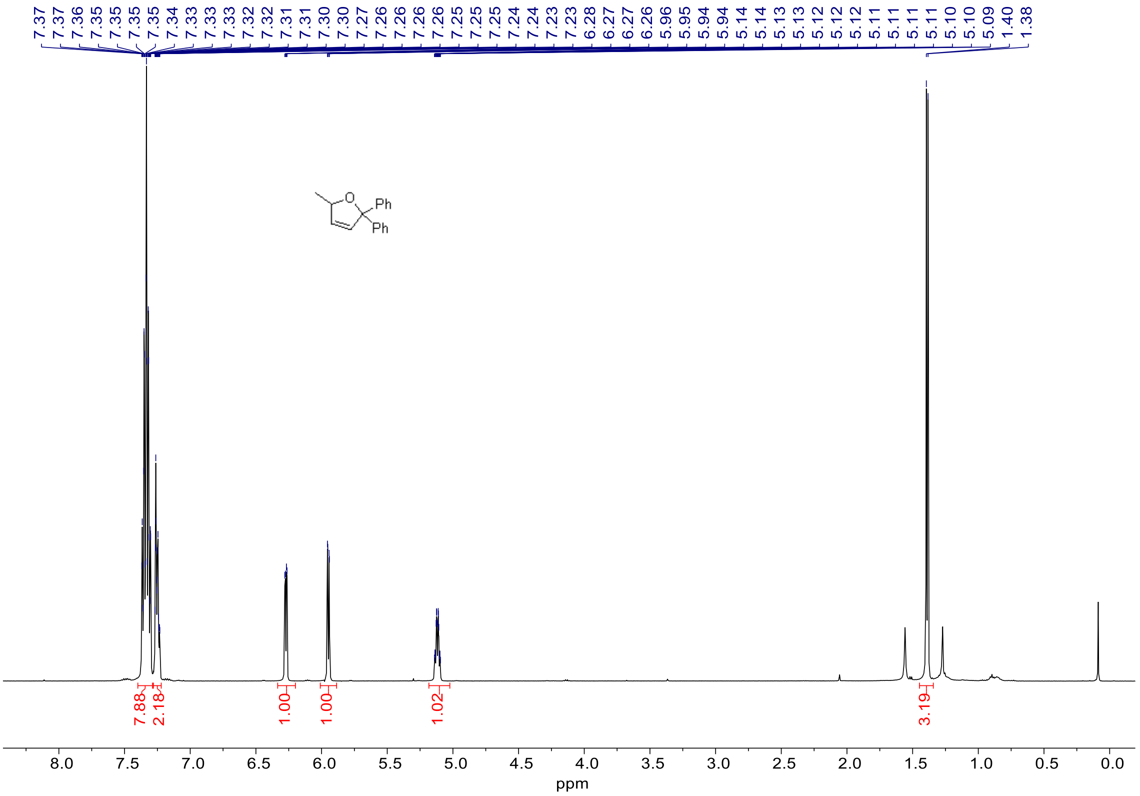

Figure S92. ^{13}C{^{1}H} NMR spectrum (126 MHz, $CDCl_3$) of dihydrofuran **4c**.

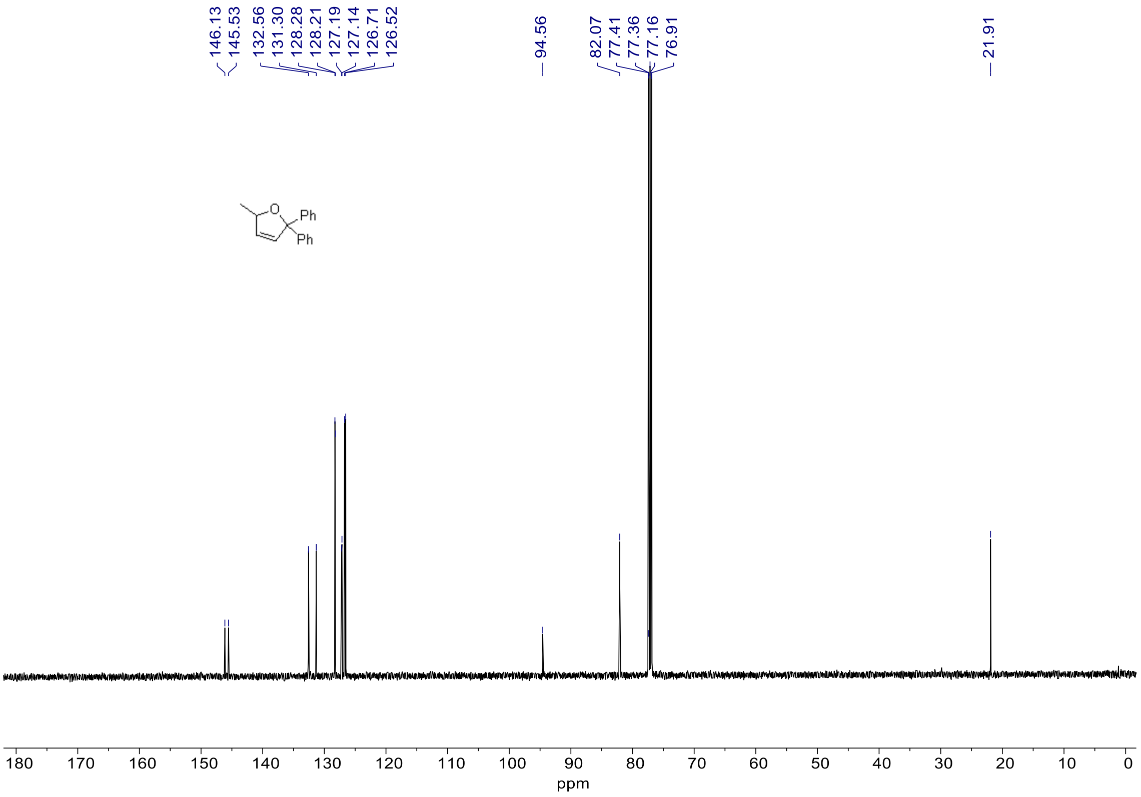

Figure S93. ^{1}H NMR spectrum (499.4 MHz, toluene-d_8) of the decay products of **3f** with 0.2 equiv PPh_3 after 60 min.

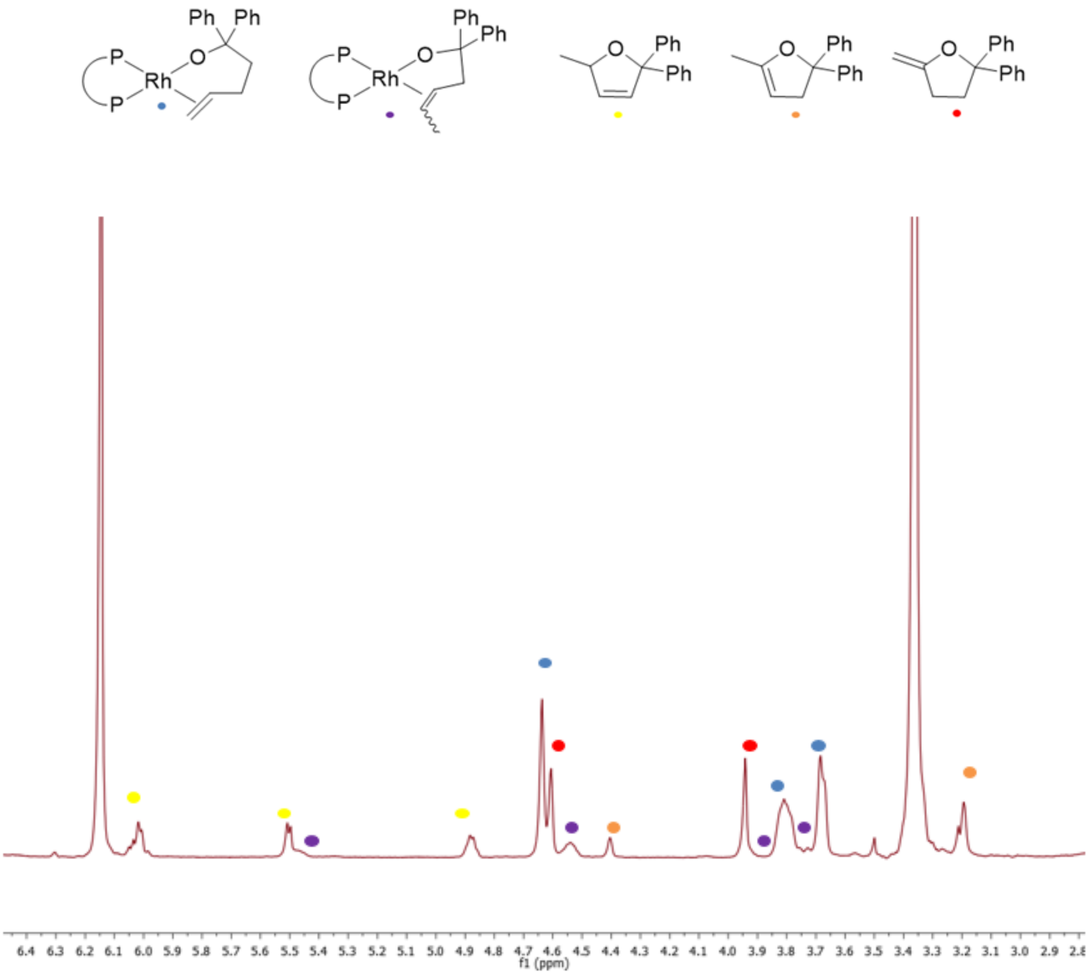

Figure S94. $^{31}P\{^1H\}$ NMR spectrum (162 MHz, toluene-d_8) observed during the decay of **3f** in the absence of PPh_3 at 35 °C, which shows the two intermediates **INT1** and **INT2**.

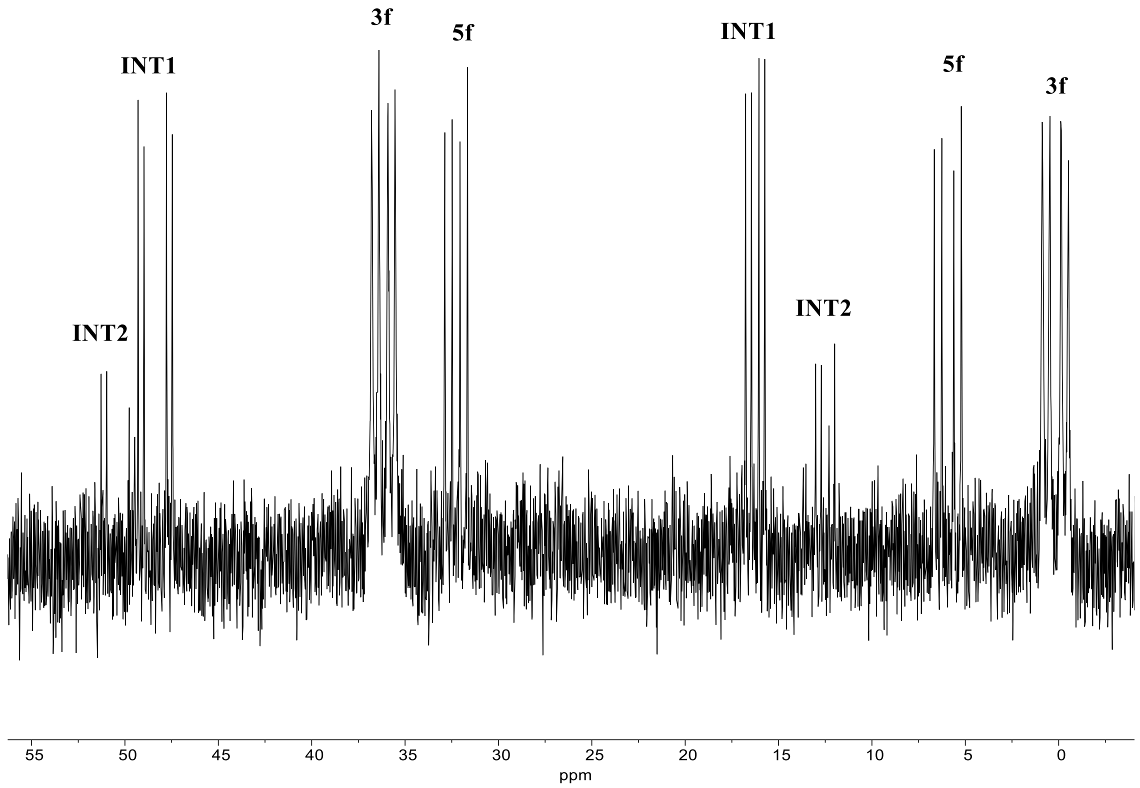

Two unknown species **INT1** und **INT2** are observed besides the starting alkoxo alkene complex **3f** and isomerized complex **5f**.

INT1: δ 48.4 (J_{PRh} = 245.8 Hz, J_{PP} = 50.5 Hz), 16.3 (J_{PRh} = 115.3 Hz, J_{PP} = 50.5 Hz) ppm.

INT2: δ 50.4 (J_{PRh} = 243.2 Hz, J_{PP} = 48.9 Hz), 12.5 (J_{PRh} = 115.9 Hz, J_{PP} = 49.1 Hz) ppm.

Because of the nearly identical coupling constants of **INT1** and **INT2**, we propose that they are either diastereomers or constitutional isomers. Both species fully decayed when the reaction was run to completion at 70 °C.

Figure S95. $^{31}P\{^{1}H\}$ NMR spectrum (202 MHz, toluene-d_8) observed during the decay of **3h** in the absence of PPh_3 at 35 °C, which shows four intermediates **INT3-6**.

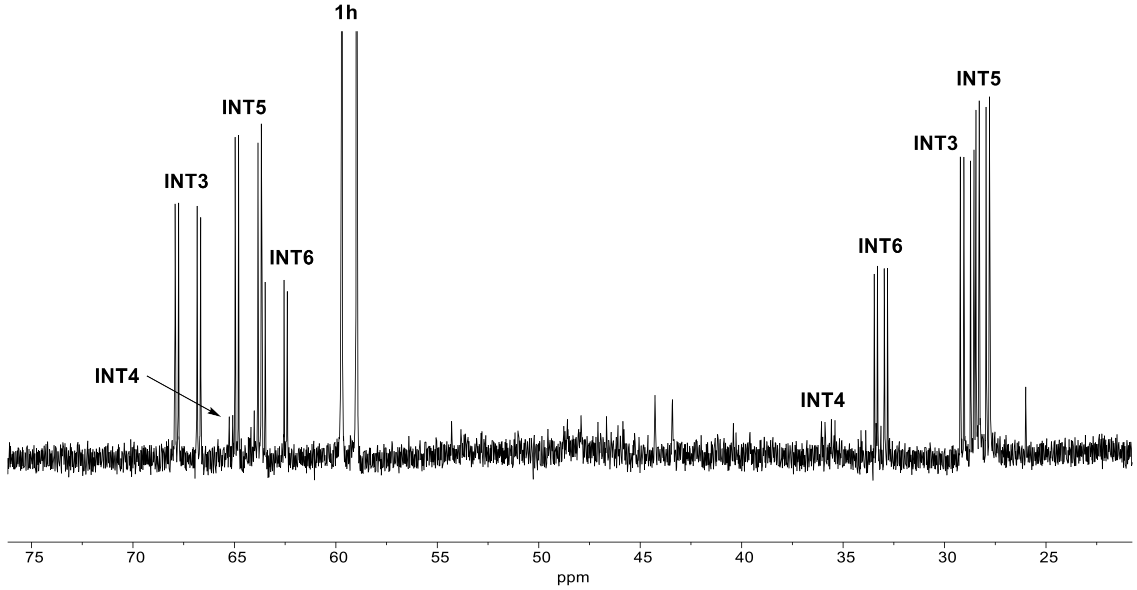

Four intermediate species **INT3-6** were observed as sets of two doublets of doublets. The $^{31}P\{^{1}H\}$ chemical shifts, ^{31}P-^{31}P coupling constants, and the ^{31}P-^{103}Rh coupling constants of all the intermediates were similar. These values are also similar, although slightly higher, to those of **INT1-2** that were observed in the reaction of complex **3f**. Like **INT1-2**, the species **INT3-6** were fully decayed when the reaction was run to completion at 70 °C. We therefore assume that **INT3-6** are the same species as **INT1-2**, but with a different ancillary ligand. Twice as many intermediates are visible in the reaction of **3h** than in the reaction of **3f** because of additional diastereomers, due to the chirality of the BINAP ligand in **3h**.

INT3: δ 67.3 (J_{PRh} = 263.9 Hz, J_{PP} = 41.3 Hz), 28.9 (J_{PRh} = 121.8 Hz, J_{PP} = 41.1 Hz) ppm.

INT4: δ 64.6 (J_{PRh} = 258.0 Hz, J_{PP} = 40.9 Hz), 35.7 (J_{PRh} = 118.3 Hz, J_{PP} = 41.9 Hz) ppm.

INT5: δ 64.3 (J_{PRh} = 272.3 Hz, J_{PP} = 40.6z Hz), 28.1 (J_{PRh} = 123.0 Hz, J_{PP} = 40.6 Hz) ppm.

INT6: δ 63.0 (J_{PRh} = 265.1 Hz, J_{PP} = 38.1 Hz), 33.1 (J_{PRh} = 119.9 Hz, J_{PP} = 38.1 Hz) ppm.

Figure S96. $^{31}P\{^1H\}$ NMR (202.2 MHz, toluene-d_8) spectrum of the reaction of alkoxo alkene complex **3f** after 2 h at 35 °C (green) and 5 minutes after the addition of 5 equiv of PPh_3 (red).

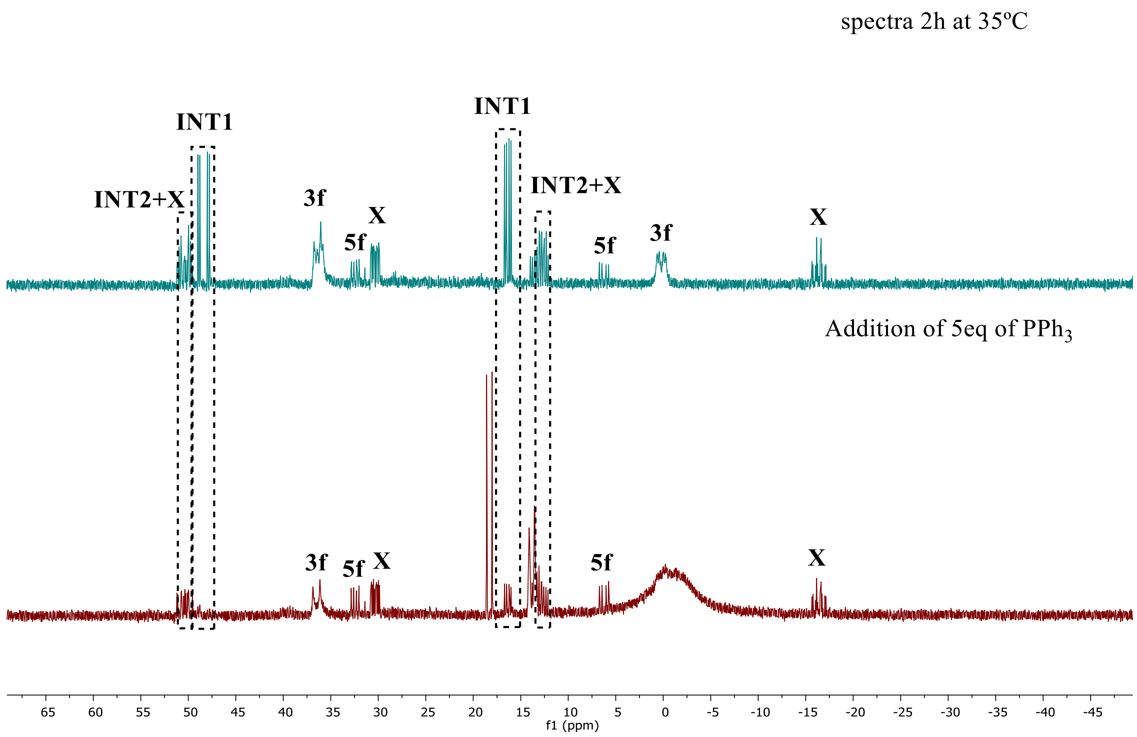

The intermediates **INT1** and **INT2** are almost fully consumed 5 minutes after the addition of 5 equiv of PPh_3. The amount of complex **3f** also decreases noticeably after the addition of PPh_3 because of its increased rate of reaction in the presence of PPh_3. Compound **X** is a degradation product of the ligand, we suspect through a C-H activation reaction. This same side-product is obtained when [Rh(DPPP)$_2$] is treated with a base.

Figure S97. $^{31}P\{^{1}H\}$ NMR (202.2 MHz, toluene-d_8) of [Rh(DPPP)(PCy_3)Cl] evolution in time after the addition of the potassium salt of the alkene.

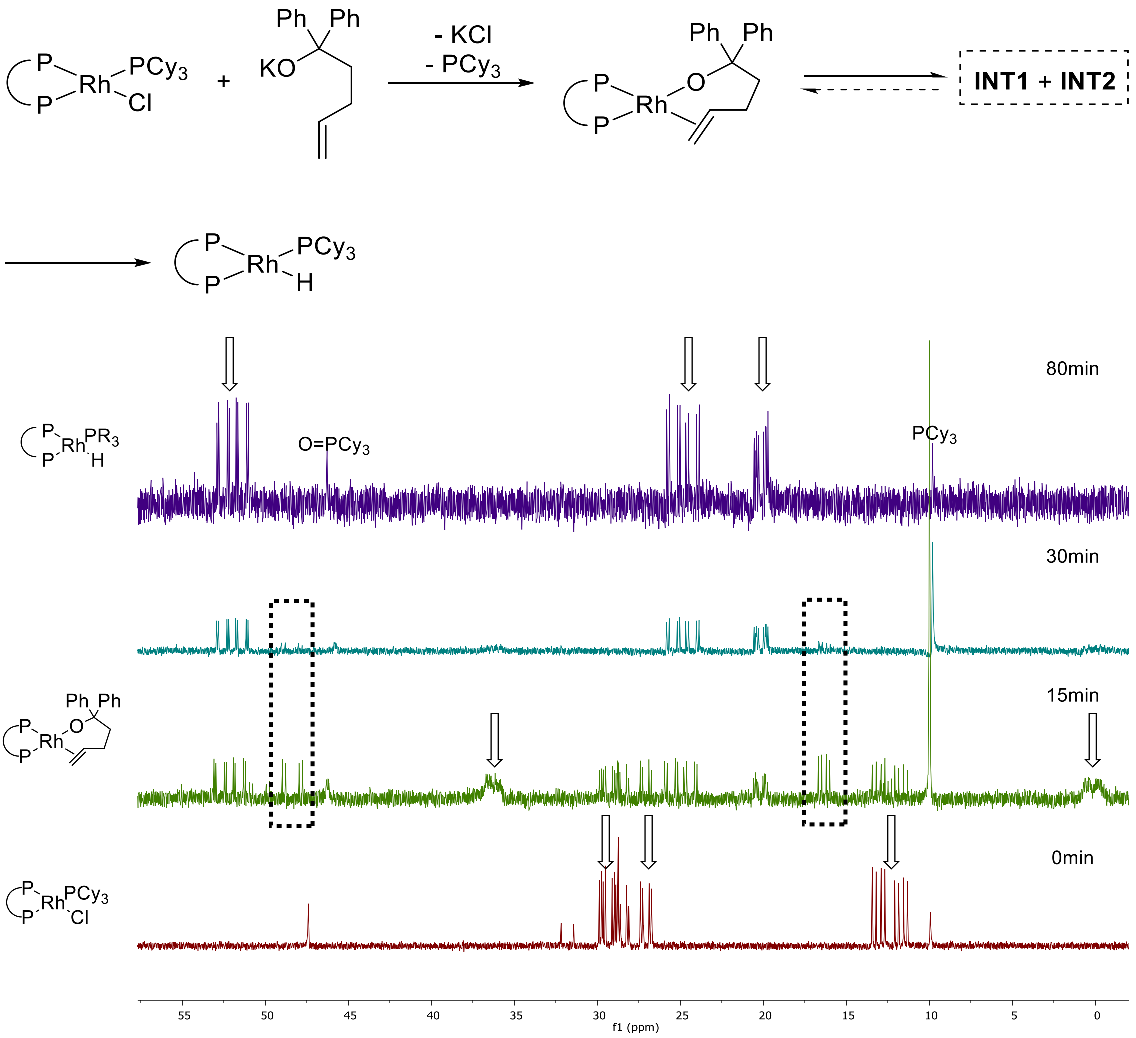

We used PCy_3 instead of PPh_3 in this experiment because its behavior was similar, but it formed a rhodium hydride that could be identified by $^{31}P\{^{1}H\}$ NMR spectroscopy. The starting complex [Rh(DPPP)(PCy_3)Cl] (arrows in the bottom spectrum) is fully consumed after 30 minutes. Alkoxo alkene complex **3f** (arrows in the second spectrum from the bottom) and **INT2** (doted box) appear. Small amounts of **INT1** are visible as well. After prolonged reaction time, complexes **3f**, **INT1**, and **INT2** are consumed, and [Rh(DPPP)(PCy_3)H] is observed as the final reaction product.

Additionally, no adducts between alkoxo alkene complex **3f** and PPh_3 were observed when they were exposed to each other at low temperature.

These experiments demonstrate that added phosphine ligand does not interact with the alkoxo alkene complexes **3a-m** prior to insertion of the alkenes into the rhodium-alkoxo bond.

Figure S98. $^{13}C\{^{1}H\}$ NMR spectrum (242 MHz, toluene-d_8) after the mixture of **2-^{13}C** and **1f** was warmed to 35 °C for 1 h.

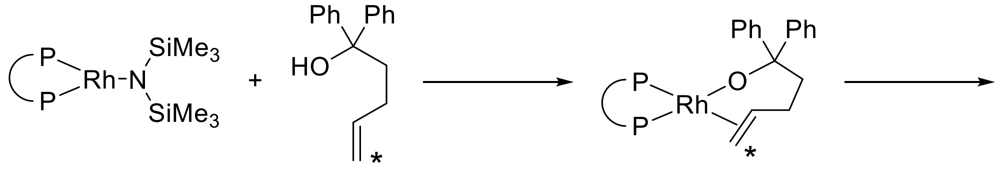

1f **2-^{13}C** **3f-^{13}C**

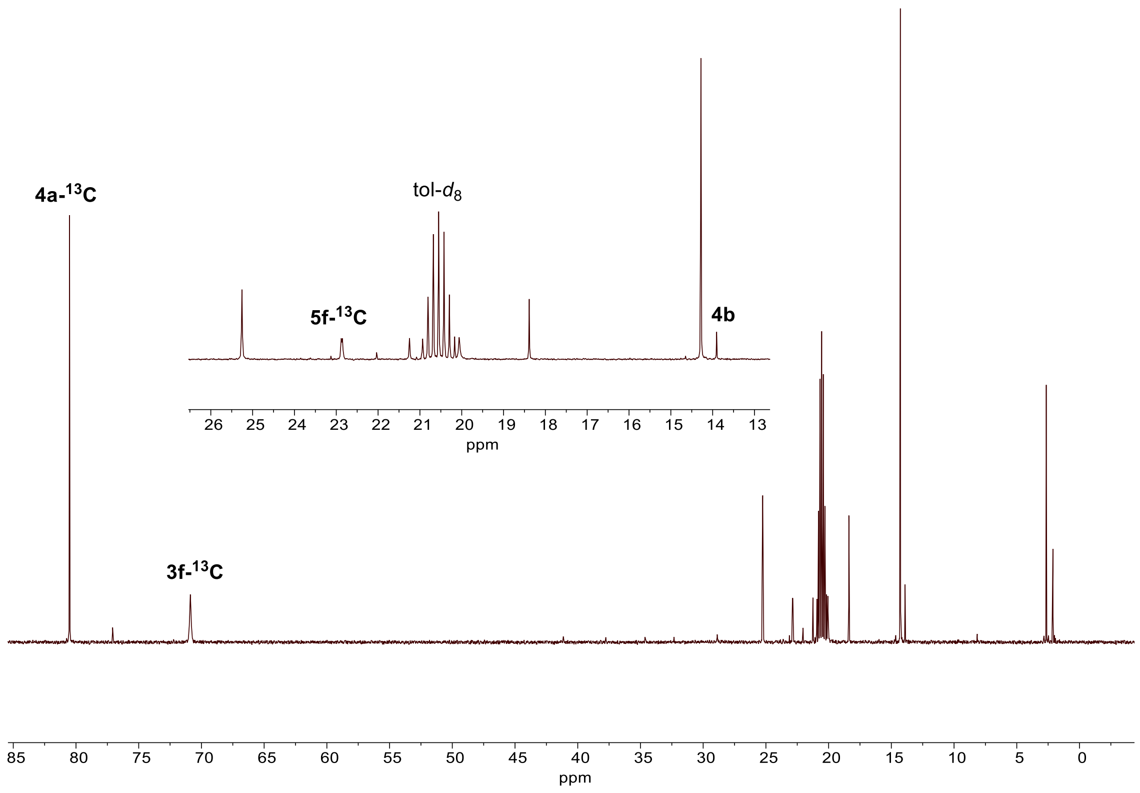

None of the peaks observed contain coupling to rhodium. This lack of coupling shows that **INT1** and **INT2** are not **INT-A** or **INT-B**.

Figure S99. $^{31}P\{^{1}H\}$ DOSY NMR spectrum (242 MHz, toluene-d_8) of **3f** after warming to 35 °C for 1 h.

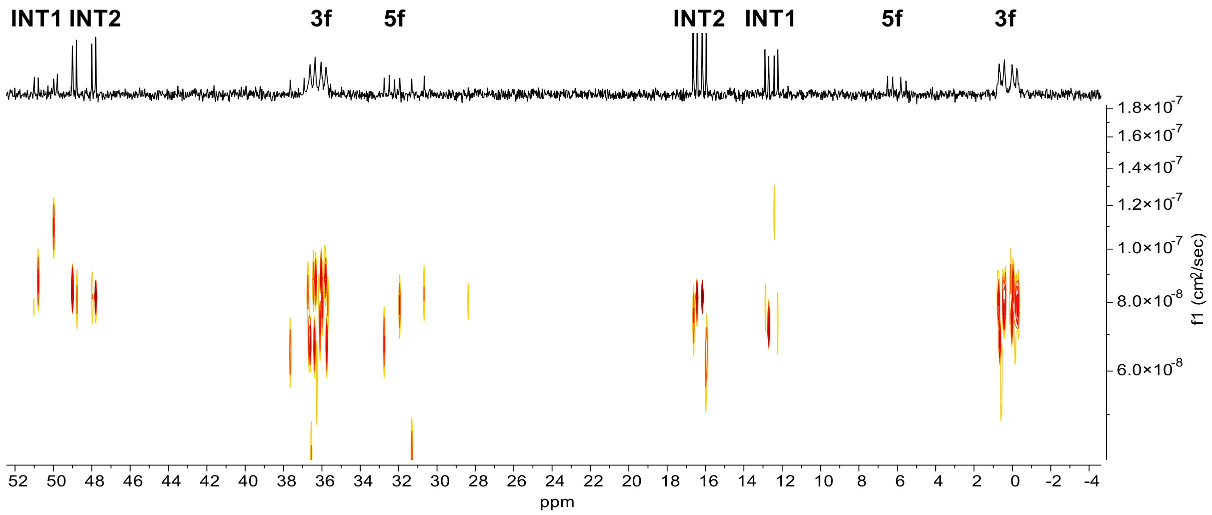

	D (cm^2/s)	$\log_{10}$(D)
**INT1**	$5.17 \cdot 10^{-8}$	-7.29
**INT2**	$5.22 \cdot 10^{-8}$	-7.28
**Xf**	$5.01 \cdot 10^{-8}$	-7.30
**Xf**	$4.64 \cdot 10^{-8}$	-7.33

Diffusion constants were calculated from the spectrum by averaging all signals for each species and multiplying them with an experimentally determined gradient correction factor of $5.35^2/6.70^2$. The determined diffusion constants for **INT1** and **INT2** are nearly the same. These data show that none of the observed species are dinuclear forms of a monomeric precursor. The diffusion constants for the isomeric alkoxo alkene complexes **3f** and **5f** are found to be slightly different from each other, although they are known to have the same molecular weight. **3f** and **5f** are both diffusing slower than the reaction intermediates **INT1** and **INT2**. This slower diffusion indicates a higher molecular weight for **3f** and **5f** than for **INT1** and **INT2** and therefore contradicts the hypothesis that **INT1** and **INT2** might be dimers of the rhodium alkyl complexes that can be formed in this reaction. Nonetheless, the determined values for all four compounds are very close to each other. The sizes of the intermediates, therefore, are similar to those of the complexes **3f** and **5f** and they are likely monomeric.

Figure S100. $^{31}P\{^{1}H\}$-^{1}H-HMBC NMR spectrum (202 MHz, toluene-d_8) after heating of **3f** at 35 °C for 1 h.

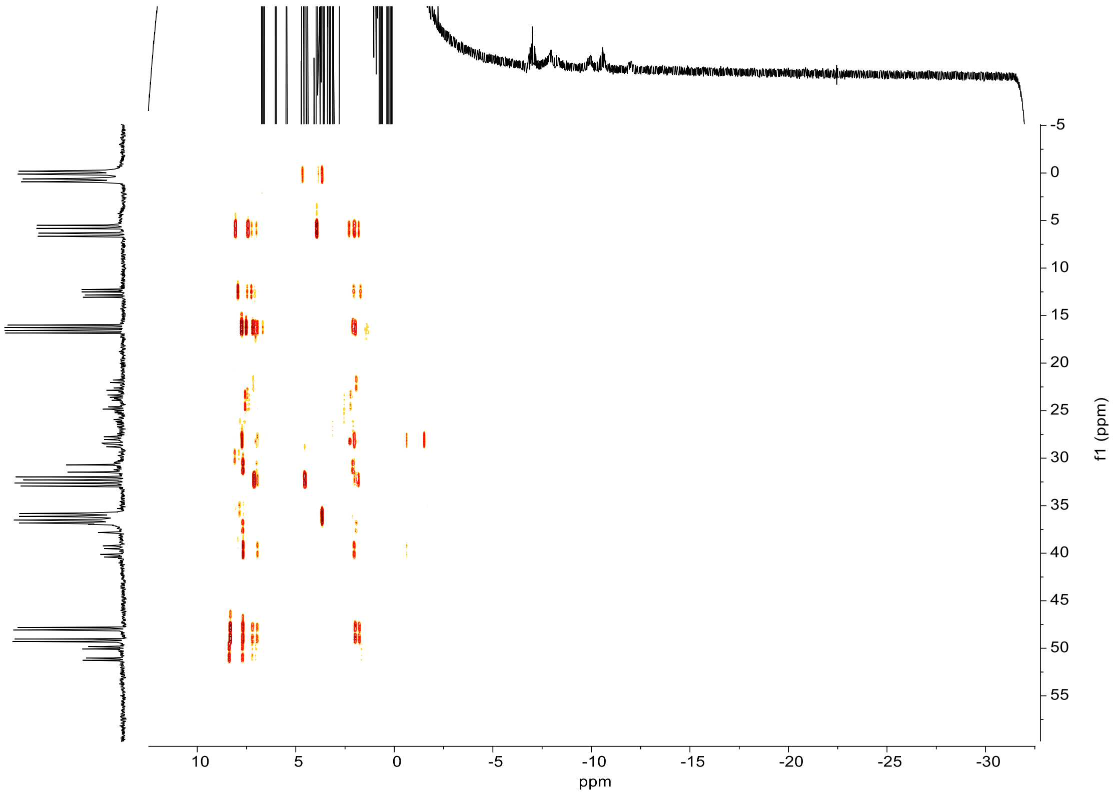

No correlation between the small hydride peaks and the phosphorous atoms of the intermediates was observed. This shows that the intermediates do not contain a hydrido ligand.

Figure S101. Stacked ^{2}H NMR spectra (92 MHz, toluene-d_8) of **2-d_{10}**, **3f-d_{10}**, and of **3-d_{10}** after reaction for 30 minutes at 35 °C.

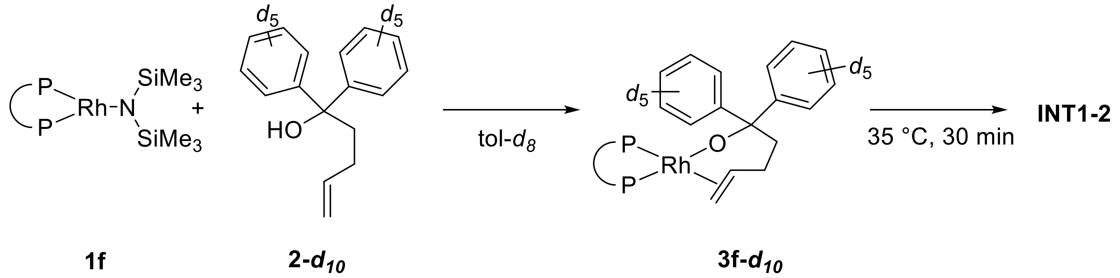

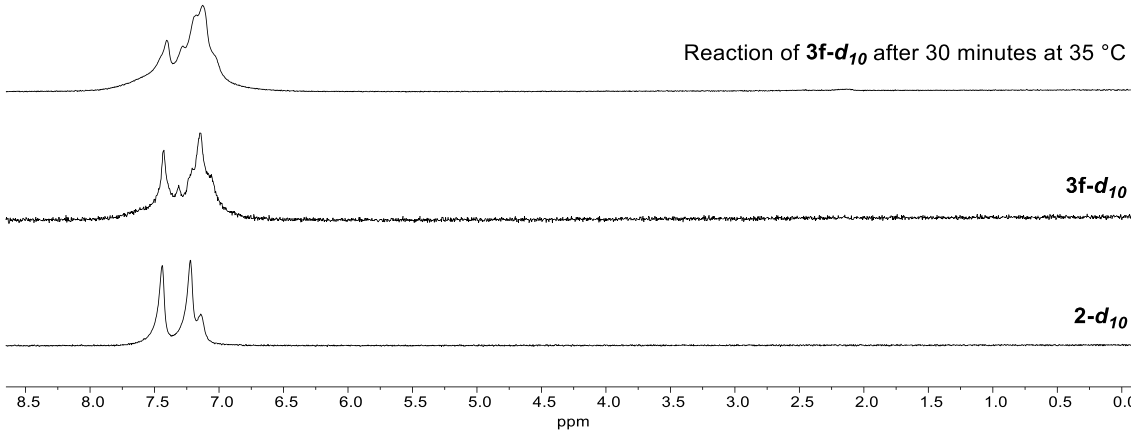

To investigate potential interactions between the rhodium center and the aryl groups of the substrate in the intermediates **INT1** and **INT2**, alkoxo alkene complex **3-d_{10}** was generated in situ from **1f** and alcohol **2-d_{10}**. The ^{2}H NMR peaks of the deuterated arene would be expected to shift to higher field if an arene were bound to rhodium. After 30 minutes at 35 °C, the intermediates had formed, but no upfield shift of the signals was observed.

In addition, no change of the chemical shifts of the ^{31}P{^{1}H} NMR spectrum of the intermediates was observed when the analogous reaction was conducted with alcohol **S1** containing a fluorine substituent on each phenyl group. This result further confirms the absence of interactions with the aryl groups with rhodium in the intermediates.

Discussion of the proposed structure of the complexes observed in the absence of PPh_3:

After accumulation of the observed complexes from complex **3f**, a large excess of concentrated hydrochloric acid was added. The species were fully consumed and 5-Methyl-2,2-diphenyltetrahydrofuran (**6**) was formed in 9% yield, as determined by ^{1}H NMR spectroscopy using TMB as internal standard. This result suggests that protodemetallation of a rhodium-alkyl complex occurred. We attempted to determine the position at which the protodemetallation occurred by adding deuterated acid instead of protic acid, but the ^{2}H NMR spectrum of the crude reaction mixture was too complex to determine the site of deuteration of the organic product. GC/HRMS analysis of the crude

reaction mixture showed a product with the expected mass of **6-*d*1** which further confirms the hypothesis that the observed complexes are rhodium alkyl complexes that undergo demetallation in the presence of strong acid.

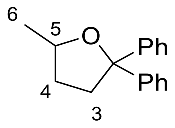

A ^{31}P DOSY NMR experiment (Figure S99) showed that the species have a similar diffusion constant to the alkoxo-alkene complexes **3f** and **5f**, suggesting that they are monomeric rhodium alkyl complexes.

Carbon-labeling experiments (Figure S98) established that rhodium is not bound at C-6 on the carbon scaffold of **6**. If rhodium were bound to C-3, it would be stabilized by rhodium-aryl interactions. No evidence was found for such interactions (Figure S101). Therefore, rhodium must be bound to C-4 or C-5. If rhodium were bound to C-5, no mixture of diastereomeric complexes would form that would give rise to two observable species. Therefore, the rhodium alkyl complex in which rhodium is bound to C-4 of the carbon scaffold of **6** is the only structure that is consistent with all experimental data.

Computational data

Full reference 42:

Gaussian 16, Revision A.03, Frisch, M. J.; Trucks, G. W.; Schlegel, H. B.; Scuseria, G. E.; Robb, M. A.; Cheeseman, J. R.; Scalmani, G.; Barone, V.; Petersson, G. A.; Nakatsuji, H.; Li, X.; Caricato, M.; Marenich, A. V.; Bloino, J.; Janesko, B. G.; Gomperts, R.; Mennucci, B.; Hratchian, H. P.; Ortiz, J. V.; Izmaylov, A. F.; Sonnenberg, J. L.; Williams-Young, D.; Ding, F.; Lipparini, F.; Egidi, F.; Goings, J.; Peng, B.; Petrone, A.; Henderson, T.; Ranasinghe, D.; Zakrzewski, V. G.; Gao, J.; Rega, N.; Zheng, G.; Liang, W.; Hada, M.; Ehara, M.; Toyota, K.; Fukuda, R.; Hasegawa, J.; Ishida, M.; Nakajima, T.; Honda, Y.; Kitao, O.; Nakai, H.; Vreven, T.; Throssell, K.; Montgomery, J. A., Jr.; Peralta, J. E.; Ogliaro, F.; Bearpark, M. J.; Heyd, J. J.; Brothers, E. N.; Kudin, K. N.; Staroverov, V. N.; Keith, T. A.; Kobayashi, R.; Normand, J.; Raghavachari, K.; Rendell, A. P.; Burant, J. C.; Iyengar, S. S.; Tomasi, J.; Cossi, M.; Millam, J. M.; Klene, M.; Adamo, C.; Cammi, R.; Ochterski, J. W.; Martin, R. L.; Morokuma, K.; Farkas, O.; Foresman, J. B.; Fox, D. J. Gaussian, Inc., Wallingford CT, 2016.

Computational details:

All calculations were conducted in Gaussian16 revision A.03. Geometries were optimized at the PBE0 level of theory^{13–16}^{13–16}[13–16] including Grimme dispersion (D3) with Becke-Johnson damping and the SMD solvent model for toluene. A mixed def2 basis set was employed. The carbon atoms of the alkoxo ligand and tetrahydrofuryl moieties, excluding the diphenylmethylene fragment, and the hydrogen atoms bound thereto were modelled with def2-SVPD. Phosphorous atoms, oxygen atoms, and hydrido ligands were also modelled with def2-SVPD. Rhodium was modelled with the def2-TZVP basis set and remaining atoms were optimized with def2-SVP. A thermal correction to 308 K was employed in the frequency calculation. Additional single point calculations were performed with the M06 functional including Grimme dispersion (D3), the SMD solvent model for toluene, and thermal correction to 308 K. Rhodium was modelled with the SDD basis set and ECP. Remaining atoms were modelled with 6-311+G(d,p). This combination of functional and basis sets has been previously used to study a rhodium(I)-catalyzed reaction including a migratory insertion step by Liu, Dong, and coworkers.[17]

All possible conformers were evaluated for the calculated compounds, except for **INT-C** whose conformers were difficult to optimize. Only one conformer was calculated for this compound and it was refrained from trying to optimize the remaining conformers because a clear trend in energy compared to the other compounds was already visible.

Geometries and energy values for calculated structures:

3f

PBE0 SCF energy: -2567.542107 a. u.

PBE0 free energy: -2566.873546 a. u.

M06 SCF energy: -2570.2400499 a. u.

M06 free energy: -2569.579395 a. u.

C	-3.3661966	0.5154370	2.1040570
C	-2.6456746	1.7907620	2.5327990
H	-3.2233296	-0.2651610	2.8673580
H	-3.2176516	2.2410110	3.3597450
H	-2.6624016	2.5362330	1.7191350
C	-3.4470306	0.9169340	-0.7685480
C	-4.5024106	1.8100820	-0.5537010
C	-2.8215646	0.8976450	-2.0239860
C	-4.9151936	2.6729320	-1.5684930
H	-5.0122256	1.8433700	0.4110060
C	-3.2412116	1.7514870	-3.0417440
H	-1.9880086	0.2067960	-2.1859450
C	-4.2867296	2.6461170	-2.8127750
H	-5.7345016	3.3723770	-1.3842350
H	-2.7437146	1.7223800	-4.0142890
H	-4.6124026	3.3242520	-3.6054880
P	-0.0565216	1.1773660	1.6340360
C	-1.2140146	1.5686280	3.0123940
H	-0.8363506	2.4578870	3.5400910
H	-1.1851266	0.7296150	3.7245900
C	0.1713964	2.8265910	0.8706550
C	0.8117904	3.8757060	1.5432870
C	-0.3444496	3.0507530	-0.4096280
C	0.9386924	5.1228510	0.9373640
H	1.2284004	3.7121770	2.5408060
C	-0.2251236	4.3021530	-1.0134910
H	-0.8431136	2.2325420	-0.9340960
C	0.4207504	5.3379990	-0.3416430
H	1.4477474	5.9334850	1.4647880
H	-0.6356416	4.4615040	-2.0135400
H	0.5236154	6.3181010	-0.8142680
C	1.5324464	0.8351290	2.4626660
C	1.5731784	0.0519890	3.6227050
C	2.7359224	1.2222450	1.8588850
C	2.7954254	-0.3218060	4.1796500
H	0.6476354	-0.2891470	4.0919750
C	3.9547314	0.8436780	2.4149570
H	2.7216474	1.8031280	0.9364550
C	3.9892044	0.0730040	3.5769230
H	2.8127344	-0.9317500	5.0863730
H	4.8838594	1.1412360	1.9230910
H	4.9472744	-0.2280180	4.0079330
P	-2.7913956	-0.2266260	0.5152660
C	-3.9198386	-1.6729080	0.3931050
C	-3.9504656	-2.6055420	1.4400890
C	-4.6913176	-1.9145500	-0.7494260
C	-4.7433866	-3.7469530	1.3498350
H	-3.3448706	-2.4461050	2.3360670
C	-5.4796906	-3.0614270	-0.8408680
H	-4.6819066	-1.1991700	-1.5743660
C	-5.5093006	-3.9788570	0.2073460
H	-4.7585056	-4.4625470	2.1756370
H	-6.0771146	-3.2357640	-1.7392200
H	-6.1279336	-4.8766930	0.1347600
Rh	-0.6167816	-0.5193160	0.1655250
O	1.3526864	-0.3978930	-0.2428750
C	1.1440084	-1.3289720	-2.4605110
H	1.7515864	-1.5889290	-3.3410930
H	0.6314454	-0.3857930	-2.7073910
C	0.1201074	-2.4354350	-2.2283180
H	-0.4299726	-2.5743500	-3.1716750
H	0.6549234	-3.3813920	-2.0521630
C	-0.9031596	-2.2399280	-1.1258510
H	-1.9423666	-2.2598490	-1.4685920
C	-0.6880766	-2.6248960	0.2068180
H	0.3001294	-2.9794030	0.5128870
H	-1.5181976	-2.9852110	0.8157930

C	2.0593294	-1.0005900	-1.2580610
C	3.0929354	0.0365330	-1.7541870
C	4.4068264	-0.2644960	-2.1216200
C	2.6725374	1.3703400	-1.8403530
C	5.2742044	0.7378550	-2.5637540
H	4.7725614	-1.2902670	-2.0473500
C	3.5315804	2.3720730	-2.2826180
H	1.6581394	1.6122830	-1.5216380
C	4.8426864	2.0593700	-2.6471650
H	6.3011184	0.4790130	-2.8362360
H	3.1768264	3.4054920	-2.3319450
H	5.5247854	2.8429620	-2.9875170
C	2.7773254	-2.2529370	-0.7373710
C	3.2550284	-3.2680000	-1.5756430
C	2.9727274	-2.3855180	0.6407590
C	3.9054864	-4.3852220	-1.0504380
H	3.1210584	-3.1914680	-2.6573020
C	3.6276234	-3.4963930	1.1690020
H	2.5881134	-1.5954680	1.2869980
C	4.0949714	-4.5045280	0.3257410
H	4.2645234	-5.1689060	-1.7231060
H	3.7716694	-3.5735350	2.2503290
H	4.6036244	-5.3800150	0.7378390
H	-4.4521516	0.6802330	2.0370000

TS1

PBE0 SCF energy: -2567.5115597 a. u.

PBE0 free energy: -2566.840815 a. u.

M06 SCF energy: -2570.215806 a. u.

M06 free energy: -2569.552652 a. u.

C	0.0715498	-1.5706627	2.6112689
O	-1.2204072	-0.5665607	1.4335979
C	-2.3650242	-1.3469647	1.3796279
C	-2.2806462	-2.3183057	2.5917969
C	-0.8365122	-2.7506707	2.7125349
H	-0.0840292	-0.7898047	3.3592019
H	-2.5733272	-1.7623567	3.4947119
H	-2.9561792	-3.1778037	2.4929009
H	-0.6551382	-3.2177197	3.6947409
H	-0.5652732	-3.4953917	1.9512699
C	-2.4708492	-2.1834867	0.0873309
C	-3.6982882	-2.4848317	-0.5149151
C	-1.3134122	-2.7026587	-0.5059051
C	-3.7672932	-3.2662597	-1.6688391
H	-4.6218342	-2.0832677	-0.0958891
C	-1.3756552	-3.4834087	-1.6589441
H	-0.3305642	-2.4494427	-0.0896571
C	-2.6052382	-3.7691497	-2.2508031
H	-4.7412532	-3.4718857	-2.1211511
H	-0.4505732	-3.8659037	-2.0988691
H	-2.6570522	-4.3764557	-3.1581371
C	-3.5950382	-0.4484427	1.5425749
C	-4.7974612	-0.9193637	2.0867619
C	-3.5325662	0.8868063	1.1404949
C	-5.9059172	-0.0817527	2.2090249
H	-4.8799002	-1.9565297	2.4190709
C	-4.6365572	1.7278973	1.2604999
H	-2.5977962	1.2603793	0.7303759
C	-5.8313992	1.2469883	1.7936349
H	-6.8337292	-0.4719267	2.6359449
H	-4.5583272	2.7675843	0.9324959
H	-6.6992802	1.9044883	1.8900639
C	1.3223988	-1.6416327	1.9880409
H	2.1304448	-1.0339727	2.4054819
H	1.6245678	-2.6085957	1.5758429
C	2.8939148	-0.0214127	-2.3087941
C	2.3769288	1.3497953	-2.7230411
H	3.9684018	-0.1138497	-2.5272921
H	2.3787638	-0.8136427	-2.8743721
H	2.7421878	1.5688423	-3.7398081
H	2.8175628	2.1233883	-2.0720711
P	2.6379768	-0.3783467	-0.5157741
C	3.4862578	-2.0005597	-0.3537431

C	4.4585008	-2.2452767	0.6216159
C	3.0522148	-3.0621357	-1.1602171
C	4.9927488	-3.5243537	0.7809529
H	4.7985308	-1.4336707	1.2683619
C	3.5910408	-4.3364017	-1.0049321
H	2.2701488	-2.8949037	-1.9057561
C	4.5637768	-4.5714657	-0.0319051
H	5.7494088	-3.7016097	1.5494799
H	3.2445328	-5.1536187	-1.6426761
H	4.9828308	-5.5727877	0.0945209
C	3.8122598	0.8114503	0.2486629
C	5.1514588	0.9106953	-0.1539831
C	3.3292108	1.6821793	1.2304609
C	5.9878848	1.8665513	0.4167359
H	5.5485008	0.2307063	-0.9128071
C	4.1654968	2.6430673	1.7990209
H	2.2797678	1.5990463	1.5270279
C	5.4947858	2.7360743	1.3923399
H	7.0313468	1.9357173	0.0987039
H	3.7707128	3.3236533	2.5572709
H	6.1525538	3.4886823	1.8347369
P	0.0062968	1.2419133	-1.1081731
C	0.8578988	1.4605463	-2.7323191
H	0.5314048	2.4176653	-3.1685951
H	0.4269158	0.6732233	-3.3727091
C	-1.7241732	1.4210323	-1.7084901
C	-2.3963682	2.6495703	-1.7113781
C	-2.3690312	0.2873503	-2.2155441
C	-3.6962462	2.7381903	-2.2059731
H	-1.9064502	3.5407143	-1.3124601
C	-3.6658512	0.3808163	-2.7167231
H	-1.8591002	-0.6787937	-2.1986001
C	-4.3338432	1.6042043	-2.7082651
H	-4.2160502	3.6997633	-2.1948841
H	-4.1600942	-0.5148317	-3.0994441
H	-5.3565102	1.6731993	-3.0874281
C	0.2793398	2.8278023	-0.2200471
C	0.9204718	3.9420893	-0.7693661
C	-0.1499642	2.8806533	1.1162239
C	1.1389808	5.0847973	0.0029649
H	1.2611418	3.9300083	-1.8062811
C	0.0503068	4.0276423	1.8783749
H	-0.6279702	2.0002603	1.5573799
C	0.7036208	5.1314943	1.3250219
H	1.6531958	5.9438003	-0.4356401
H	-0.2958212	4.0560403	2.9146399
H	0.8741758	6.0275173	1.9271989
Rh	0.6115798	-0.3914137	0.3016149

INT-A

PBE0 SCF energy: -2567.5331001 a. u.

PBE0 free energy: -2566.861287 a. u.

M06 SCF energy: -2570.23442 a. u.

M06 free energy: -2569.570286 a. u.

C	0.1712426	-1.9377548	-2.3419074
O	1.0173106	-1.0877308	-1.4947534
C	2.3954966	-1.4334458	-1.5453474
C	2.4510176	-2.5223128	-2.6515714
C	1.0614586	-3.1383218	-2.6145884
H	0.0123296	-1.3695928	-3.2741264
H	2.6275856	-2.0409368	-3.6237434
H	3.2585106	-3.2422978	-2.4738104
H	0.7922556	-3.6421198	-3.5515404
H	0.9677136	-3.8656878	-1.7955654
C	2.8207376	-2.0468778	-0.2068324
C	4.1603806	-2.0859748	0.1941416
C	1.8692006	-2.6843368	0.5972466
C	4.5431266	-2.7758378	1.3427956
H	4.9214276	-1.5660768	-0.3888874
C	2.2509246	-3.3739668	1.7471576
H	0.8077826	-2.6165268	0.3388636
C	3.5916336	-3.4345368	2.1197106

H	5.5960936	-2.7891998	1.6354566
H	1.4865726	-3.8640688	2.3561976
H	3.8928046	-3.9787238	3.0185206
C	3.1853816	-0.1926408	-1.9309184
C	4.4695226	-0.2882758	-2.4812304
C	2.6163226	1.0739612	-1.7777164
C	5.1774236	0.8573352	-2.8407144
H	4.9226216	-1.2687608	-2.6460524
C	3.3177716	2.2198072	-2.1482334
H	1.6059316	1.1551042	-1.3765074
C	4.6047766	2.1177512	-2.6725444
H	6.1796126	0.7620442	-3.2663594
H	2.8488056	3.1996892	-2.0239284
H	5.1565406	3.0158512	-2.9613624
C	-1.1118414	-2.0988768	-1.5672634
H	-2.0025224	-1.9620768	-2.1961864
H	-1.1859094	-3.0925998	-1.0961184
C	-3.0627504	0.0126282	2.2821436
C	-2.3294514	1.2312502	2.8320836
H	-4.1516764	0.1140782	2.4125076
H	-2.7718814	-0.8899188	2.8441776
H	-2.7304764	1.4455682	3.8365986
H	-2.5552714	2.1230362	2.2232936
P	-2.7159894	-0.4027398	0.5158146
C	-3.8051074	-1.8784738	0.3495256
C	-5.0725594	-1.8361518	-0.2389864
C	-3.3288314	-3.0959028	0.8543716
C	-5.8501324	-2.9924498	-0.3211244
H	-5.4556504	-0.8959588	-0.6420634
C	-4.1102744	-4.2457388	0.7839546
H	-2.3259554	-3.1361318	1.2897996
C	-5.3736434	-4.1969688	0.1916616
H	-6.8363314	-2.9488718	-0.7906334
H	-3.7276464	-5.1887378	1.1828816
H	-5.9836824	-5.1014378	0.1257166
C	-3.6317954	0.8865752	-0.4283024
C	-4.5263534	1.7918162	0.1525676
C	-3.3850674	0.9742352	-1.8063434
C	-5.1494314	2.7708302	-0.6229924
H	-4.7459524	1.7451012	1.2205456
C	-4.0218454	1.9368582	-2.5840144
H	-2.6675014	0.2828072	-2.2559684
C	-4.9006194	2.8454132	-1.9913444
H	-5.8350004	3.4793362	-0.1511204
H	-3.8212724	1.9869762	-3.6572844
H	-5.3895004	3.6126582	-2.5970194
P	0.0456456	0.9839072	1.3128606
C	-0.8194774	1.0622022	2.9491156
H	-0.3847604	1.8810552	3.5421576
H	-0.5826664	0.1244352	3.4760046
C	1.7969086	0.9793922	1.8784366
C	2.7442336	1.8912052	1.4000126
C	2.2134846	-0.0153378	2.7744206
C	4.0748246	1.8146312	1.8097036
H	2.4435426	2.6720782	0.7004566
C	3.5372936	-0.0787178	3.1987256
H	1.4996096	-0.7581758	3.1376276
C	4.4742116	0.8350952	2.7154296
H	4.8016486	2.5285452	1.4138956
H	3.8421496	-0.8578458	3.9008256
H	5.5161986	0.7770972	3.0400046
C	-0.1772274	2.7114042	0.7149436
C	0.0752506	3.8352252	1.5137936
C	-0.6485354	2.8978502	-0.5885764
C	-0.1434994	5.1172042	1.0158056
H	0.4564566	3.7105022	2.5308976
C	-0.8680594	4.1813172	-1.0893324
H	-0.8538504	2.0106982	-1.1954924
C	-0.6165724	5.2921042	-0.2867484
H	0.0567486	5.9873012	1.6464926
H	-1.2447694	4.3097652	-2.1070164
H	-0.7903454	6.2995182	-0.6737954

Rh	-0.6668324	-0.6241318	-0.1433734

TS2

PBE0 SCF energy: -2567.522231 a. u.

PBE0 free energy: -2566.85792 a. u.

M06 SCF energy: -2570.222009 a. u.

M06 free energy: -2569.56367 a. u.

C	-3.1387465	1.3066250	0.0954275
O	-1.9027235	1.8516530	0.5797755
C	-1.8472515	2.2877400	-1.6837825
C	-3.0225675	1.3512410	-1.4515245
H	-2.1592575	3.3434740	-1.6548595
H	-1.3098355	2.1028390	-2.6201445
H	-3.9540815	1.6843540	-1.9218865
H	-2.7703315	0.3610850	-1.8485435
C	-1.0059905	2.0103760	-0.4599015
C	0.2530905	2.6014450	-0.1782685
H	0.6715015	3.2791140	-0.9271535
H	0.4570855	2.8716250	0.8616045
C	-4.2698045	2.2151550	0.5662665
C	-5.5978735	1.7843560	0.4624185
C	-4.0108495	3.4945220	1.0616565
C	-6.6457445	2.6198630	0.8379335
H	-5.8116585	0.7785430	0.0915575
C	-5.0619855	4.3304040	1.4429395
H	-2.9762375	3.8277700	1.1525225
C	-6.3811685	3.8985500	1.3315045
H	-7.6768925	2.2676790	0.7518855
H	-4.8434295	5.3280460	1.8326185
H	-7.2030015	4.5533710	1.6317905
C	-3.3036655	-0.0875900	0.6928625
C	-3.6200965	-1.2210850	-0.0574515
C	-3.1594985	-0.2202350	2.0811265
C	-3.7853555	-2.4618620	0.5626395
H	-3.7378985	-1.1564770	-1.1397875
C	-3.3262985	-1.4539680	2.7004305
H	-2.9154965	0.6620430	2.6766745
C	-3.6404425	-2.5833540	1.9407975
H	-4.0220485	-3.3377440	-0.0458275
H	-3.2120055	-1.5355520	3.7844665
H	-3.7667415	-3.5552240	2.4241495
Rh	0.7146455	0.5852870	-0.6058215
H	-0.8736165	0.4191970	-0.8103045
P	2.9537755	0.7948480	-0.4508915
P	0.8591715	-1.5930530	-1.0966995
C	2.3011095	-2.1367380	-2.1179425
C	3.9177045	-0.1654360	-1.7030585
C	3.6705155	-1.6708090	-1.6265545
H	4.9893625	0.0496000	-1.5694265
H	3.6286955	0.2118350	-2.6965675
H	4.4352415	-2.1697680	-2.2442755
H	3.8473415	-2.0191140	-0.5952155
H	2.2765465	-3.2328580	-2.2275815
H	2.1010625	-1.7253230	-3.1210585
C	0.8980555	-2.6959860	0.3710535
C	1.8223685	-3.7300260	0.5534655
C	-0.0352385	-2.4330250	1.3850925
C	1.8242245	-4.4789370	1.7313075
H	2.5561135	-3.9608820	-0.2207235
C	-0.0422165	-3.1911070	2.5520925
H	-0.7544235	-1.6192590	1.2546475
C	0.8935365	-4.2115770	2.7325365
H	2.5600785	-5.2761090	1.8645895
H	-0.7793935	-2.9759710	3.3289795
H	0.8965285	-4.7975320	3.6552015
C	-0.5154425	-2.3099050	-2.0923215
C	-1.0789395	-1.5138660	-3.0976195
C	-1.0151865	-3.5995850	-1.8865855
C	-2.1223515	-1.9972730	-3.8838805
H	-0.6977175	-0.4983040	-3.2424125
C	-2.0684715	-4.0796490	-2.6658345
H	-0.5923485	-4.2283140	-1.0994655

C	-2.6249345	-3.2810360	-3.6640095
H	-2.5526275	-1.3655920	-4.6653765
H	-2.4592995	-5.0849160	-2.4877185
H	-3.4519035	-3.6579570	-4.2711405
C	3.7153705	0.2120270	1.1156365
C	5.0978405	0.2581750	1.3397065
C	2.8800565	-0.3291780	2.0977665
C	5.6333455	-0.2407990	2.5241565
H	5.7601805	0.6948270	0.5867835
C	3.4167355	-0.8315050	3.2835295
H	1.8025065	-0.3533910	1.9107735
C	4.7930615	-0.7898170	3.4959055
H	6.7125845	-0.2014400	2.6932425
H	2.7539665	-1.2591600	4.0396595
H	5.2166795	-1.1835940	4.4234955
C	3.6449685	2.4863420	-0.6188845
C	3.9799135	3.0163020	-1.8708205
C	3.7263555	3.3141600	0.5090855
C	4.3997155	4.3408390	-1.9899875
H	3.9084285	2.3974630	-2.7679675
C	4.1450005	4.6370990	0.3882875
H	3.4617215	2.9172330	1.4925815
C	4.4847825	5.1549470	-0.8618095
H	4.6617945	4.7386640	-2.9738175
H	4.2064595	5.2683970	1.2784395
H	4.8138695	6.1927670	-0.9561635

INT-B

PBE0 SCF energy: -2567.52532 a. u.

PBE0 free energy: -2566.861211 a. u.

M06 SCF energy: -2570.22625 a. u.

M06 free energy: -2569.570188 a. u.

C	-3.0856669	1.2992981	0.0752094
O	-1.8612779	1.8680891	0.5554394
C	-1.7931789	2.2320621	-1.7262516
C	-2.9514809	1.2820351	-1.4695026
H	-2.1401639	3.2781111	-1.7704626
H	-1.2340939	2.0099191	-2.6413636
H	-3.8831729	1.5810651	-1.9627846
H	-2.6715339	0.2821391	-1.8187326
C	-0.9713149	2.0557321	-0.4773456
C	0.2887851	2.5920841	-0.2206336
H	0.7700461	3.1911581	-0.9956256
H	0.5632281	2.8263921	0.8106064
C	-4.2283359	2.2212151	0.4941874
C	-5.5542099	1.7843291	0.3874974
C	-3.9798679	3.5196091	0.9436624
C	-6.6090369	2.6311621	0.7153444
H	-5.7616169	0.7647341	0.0526064
C	-5.0377709	4.3673991	1.2767424
H	-2.9474069	3.8588521	1.0373834
C	-6.3544669	3.9286291	1.1629124
H	-7.6381119	2.2732281	0.6278994
H	-4.8265769	5.3799441	1.6304734
H	-7.1818499	4.5925701	1.4256184
C	-3.2601329	-0.0704629	0.7229734
C	-3.6226569	-1.2166359	0.0132824
C	-3.0879839	-0.1665379	2.1101514
C	-3.8026949	-2.4349789	0.6712994
H	-3.7633979	-1.1766939	-1.0676116
C	-3.2718299	-1.3781569	2.7685394
H	-2.8073179	0.7255431	2.6739144
C	-3.6298979	-2.5205699	2.0495104
H	-4.0728619	-3.3224259	0.0939344
H	-3.1353319	-1.4314889	3.8518124
H	-3.7686539	-3.4748809	2.5635434
Rh	0.5999731	0.4961771	-0.6536696
H	-0.8743819	-0.0830779	-0.8000356
P	2.8559321	0.8470341	-0.4559296
P	0.8995361	-1.6694009	-1.0784416
C	2.4052831	-2.1667889	-2.0268606
C	3.9193301	-0.1196569	-1.6209676

C	3.7353731	-1.6301189	-1.5040596
H	4.9721141	0.1488531	-1.4444906
H	3.6653841	0.2140401	-2.6394366
H	4.5415781	-2.1142739	-2.0793466
H	3.8904761	-1.9383829	-0.4567276
H	2.4293771	-3.2654829	-2.1045986
H	2.2239181	-1.7936019	-3.0483296
C	0.9450891	-2.7110539	0.4324114
C	1.9087331	-3.6965189	0.6724414
C	-0.0247429	-2.4553439	1.4132694
C	1.9126431	-4.4050909	1.8748014
H	2.6710921	-3.9206849	-0.0755626
C	-0.0286119	-3.1744039	2.6047144
H	-0.7716529	-1.6764079	1.2366144
C	0.9453851	-4.1458569	2.8429004
H	2.6786851	-5.1640519	2.0531534
H	-0.7941739	-2.9660249	3.3554354
H	0.9498221	-4.7002379	3.7848734
C	-0.3881749	-2.4944439	-2.1023316
C	-0.9608239	-1.7665099	-3.1529196
C	-0.8118999	-3.8063859	-1.8711446
C	-1.9377479	-2.3418989	-3.9618556
H	-0.6432139	-0.7317939	-3.3132176
C	-1.8019829	-4.3770669	-2.6721216
H	-0.3816049	-4.3814599	-1.0478366
C	-2.3661339	-3.6480179	-3.7175806
H	-2.3765959	-1.7641399	-4.7794576
H	-2.1363949	-5.3986929	-2.4737356
H	-3.1432569	-4.0962289	-4.3419276
C	3.5543431	0.3561041	1.1689724
C	4.9274171	0.4002131	1.4468214
C	2.6736571	-0.1213839	2.1451144
C	5.4082321	-0.0360819	2.6785804
H	5.6265041	0.7851171	0.6991264
C	3.1557891	-0.5609189	3.3781604
H	1.6045401	-0.1497919	1.9118294
C	4.5227311	-0.5206229	3.6443304
H	6.4803421	0.0007481	2.8885564
H	2.4581931	-0.9409379	4.1283194
H	4.9037701	-0.8667369	4.6086484
C	3.5001991	2.5485161	-0.6897936
C	3.6355951	3.0695061	-1.9839716
C	3.7346741	3.3902061	0.4043544
C	4.0179871	4.3950831	-2.1782906
H	3.4286531	2.4396191	-2.8527446
C	4.1127181	4.7179281	0.2080634
H	3.6225151	3.0044591	1.4204284
C	4.2594671	5.2237841	-1.0824656
H	4.1244231	4.7845341	-3.1940456
H	4.2945031	5.3611011	1.0729204
H	4.5572471	6.2640791	-1.2351776

INT-C

PBE0 SCF energy: -3602.38217 a. u.

PBE0 free energy: -3601.453841 a. u.

C	-5.1997100	-0.3797581	1.2143418
O	-4.5490260	-0.4472121	2.4890398
C	-2.9132780	0.1736489	0.9363418
C	-4.0427370	-0.5662581	0.2290608
H	-2.9354540	1.2402829	0.6947648
H	-1.9139080	-0.1324051	0.6346538
H	-4.2736310	-0.1678141	-0.7650512
H	-3.7909440	-1.6292551	0.1402148
C	-3.2536360	-0.0590161	2.3831788
C	-2.4879220	0.1097519	3.4651368
H	-1.4528450	0.3990179	3.3519638
H	-2.8770010	-0.0695941	4.4683288
C	-5.8290360	1.0014279	1.0345678
C	-6.5190650	1.3023079	-0.1451772
C	-5.7070570	1.9863529	2.0173128
C	-7.0698820	2.5652299	-0.3418682
H	-6.6362880	0.5364659	-0.9161592

C	-6.2615200	3.2523029	1.8204018
H	-5.1737420	1.7605089	2.9415638
C	-6.9420660	3.5470439	0.6421078
H	-7.6067000	2.7836389	-1.2683932
H	-6.1579230	4.0128619	2.5985228
H	-7.3758720	4.5383349	0.4893888
C	-6.2615270	-1.4587471	1.1623078
C	-6.5030730	-2.1853511	-0.0053762
C	-7.0546010	-1.7021231	2.2892258
C	-7.5176140	-3.1414821	-0.0484032
H	-5.8950690	-2.0120561	-0.8950482
C	-8.0618630	-2.6621861	2.2491468
H	-6.8750170	-1.1340091	3.2038838
C	-8.2982890	-3.3844371	1.0788868
H	-7.6922960	-3.7030181	-0.9694682
H	-8.6692180	-2.8473641	3.1386318
H	-9.0901940	-4.1368411	1.0474088
Rh	1.3961280	-0.5037361	0.2896308
H	0.6597180	-1.8686331	0.5621058
P	2.3662370	1.6475129	0.1385018
P	3.2514670	-1.7005721	0.3553218
P	0.3888880	-0.6091711	-1.6653202
C	1.8062360	-0.7189031	-2.8027162
H	1.4615670	-0.9205191	-3.8371392
H	2.2806810	0.2731219	-2.8198172
C	3.8667290	-1.4968661	-1.3848432
H	4.7614830	-2.0992311	-1.6220712
H	4.1833070	-0.4432031	-1.3999922
C	2.8356230	-1.8211221	-2.4601882
H	2.3316580	-2.7703581	-2.2312392
H	3.3854850	-2.0245021	-3.3984262
C	3.2003430	2.1521699	1.7119038
C	4.5154310	2.5798389	1.8898478
C	2.3514330	2.1101699	2.8292748
C	4.9707900	2.9528429	3.1548468
H	5.2039260	2.6624389	1.0456898
C	2.8055360	2.4812869	4.0891828
H	1.3158710	1.7802679	2.7137648
C	4.1234470	2.9074999	4.2586748
H	6.0046100	3.2877259	3.2657938
H	2.1210490	2.4383739	4.9401628
H	4.4849580	3.2023029	5.2465478
C	3.5560970	1.9161119	-1.2014682
C	3.0761640	2.4386099	-2.4076832
C	4.8770140	1.4607189	-1.1351492
C	3.9169600	2.5361679	-3.5156242
H	2.0297520	2.7557849	-2.4889892
C	5.7108820	1.5524549	-2.2444422
H	5.2469520	0.9739649	-0.2311142
C	5.2400930	2.1029159	-3.4384052
H	3.5260090	2.9501279	-4.4486292
H	6.7371480	1.1823319	-2.1724782
H	5.8962370	2.1802989	-4.3083792
C	1.2496200	3.0840479	-0.0332392
C	1.5984390	4.3520759	-0.4841432
C	-0.0539800	2.8309509	0.4185728
C	0.6434790	5.3646319	-0.4718812
H	2.6269940	4.5657619	-0.8130592
C	-1.0050030	3.8501169	0.4448238
H	-0.3091850	1.8091059	0.7118538
C	-0.6511400	5.1217169	-0.0067552
H	0.9214840	6.3617859	-0.8216152
H	-2.0178350	3.6411679	0.7981728
H	-1.3880030	5.9282359	-0.0033962
C	-0.5380200	0.8715549	-2.2345372
C	-1.6661430	1.3212919	-1.5241712
C	-0.1241020	1.5968129	-3.3635682
C	-2.3666340	2.4415919	-1.9886492
H	-2.1511460	0.8780289	-0.5763662
C	-0.8033560	2.7327389	-3.7836202
H	0.7480860	1.2620809	-3.9283632
C	-1.9429470	3.1581069	-3.1011252

H	-3.2581640	2.7724749	-1.4472122
H	-0.4488250	3.2735509	-4.6647002
H	-2.4974700	4.0380459	-3.4325972
C	-0.6941810	-1.9865221	-2.2716132
C	-0.2050240	-3.1355411	-2.9100042
C	-2.0855380	-1.8964191	-2.1276892
C	-1.0561450	-4.1591121	-3.3233212
H	0.8580780	-3.2674101	-3.0996062
C	-2.9425760	-2.9082801	-2.5443792
H	-2.5096640	-0.9916491	-1.7023062
C	-2.4330560	-4.0622591	-3.1389572
H	-0.6249170	-5.0410521	-3.8041492
H	-4.0204550	-2.7870731	-2.4100562
H	-3.0989820	-4.8629061	-3.4682562
C	4.7503530	-1.4029461	1.3850348
C	6.0442840	-1.3113761	0.8617518
C	4.5751090	-1.2571881	2.7675648
C	7.1367420	-1.0784681	1.6970458
H	6.2078570	-1.4282051	-0.2131302
C	5.6647730	-1.0465941	3.6032578
H	3.5636670	-1.2893351	3.1824368
C	6.9523960	-0.9530261	3.0722758
H	8.1399910	-1.0080751	1.2682308
H	5.5041840	-0.9330231	4.6777218
H	7.8065580	-0.7746681	3.7298628
C	3.1453710	-3.5315471	0.5870928
C	3.5406860	-4.4639801	-0.3786872
C	2.6216560	-4.0159861	1.7946098
C	3.4008710	-5.8344061	-0.1541192
H	3.9704190	-4.1368451	-1.3267142
C	2.4968830	-5.3807891	2.0282608
H	2.2824510	-3.3064191	2.5545048
C	2.8807360	-6.3007871	1.0502328
H	3.7106480	-6.5398161	-0.9297392
H	2.0812210	-5.7293991	2.9771888
H	2.7715490	-7.3732511	1.2286608

[Rh(DPPP)(PPh_3)H]

PBE0 SCF energy: -2871.0254 a. u.

PBE0 free energy: -2870.368056 a. u.

C	0.9526280	-2.0424816	-2.6116648
C	2.4564850	-2.1062646	-2.3681158
H	0.5793480	-3.0236056	-2.9436108
H	0.7168430	-1.3265096	-3.4158518
H	2.9159020	-2.6519136	-3.2091078
H	2.6566410	-2.7175976	-1.4722228
C	0.2742030	-2.9125576	0.0090462
C	0.2023140	-4.2592356	-0.3742158
C	0.6916150	-2.5993246	1.3076882
C	0.5216910	-5.2682126	0.5320432
H	-0.1020420	-4.5274866	-1.3892828
C	1.0155820	-3.6077886	2.2142592
H	0.7768670	-1.5434926	1.5841522
C	0.9268130	-4.9439546	1.8281402
H	0.4597270	-6.3151216	0.2237862
H	1.3486280	-3.3447826	3.2211942
H	1.1820320	-5.7377466	2.5349582
P	2.6615140	0.3033004	-0.8260638
C	3.1571190	-0.7552926	-2.2574738
H	4.2517630	-0.8824986	-2.2500828
H	2.9292780	-0.1396736	-3.1435718
C	3.4993090	-0.4559566	0.6223562
C	4.3672270	-1.5497406	0.5420492
C	3.2003920	0.0811964	1.8842352
C	4.9187160	-2.1024046	1.6994572
H	4.6184900	-1.9882536	-0.4253538
C	3.7616660	-0.4617196	3.0358052
H	2.5045720	0.9222204	1.9500922
C	4.6187040	-1.5608916	2.9469132
H	5.5867620	-2.9640026	1.6219132
H	3.5205270	-0.0304216	4.0107222
H	5.0504720	-1.9955606	3.8520082
C	3.7204230	1.7673964	-1.1756508

C	3.1280700	2.9426104	-1.6476278
C	5.1120880	1.7130954	-1.0292348
C	3.9152820	4.0447204	-1.9795258
H	2.0383840	2.9866384	-1.7303148
C	5.8972030	2.8182774	-1.3509118
H	5.5859560	0.8059654	-0.6445128
C	5.3001680	3.9850094	-1.8300048
H	3.4418280	4.9588864	-2.3467618
H	6.9816730	2.7703484	-1.2224868
H	5.9167090	4.8521804	-2.0803948
P	-0.0381030	-1.5088786	-1.1349978
C	-1.7215080	-1.7886416	-1.8213758
C	-2.1618640	-0.8525726	-2.7683078
C	-2.5717900	-2.8381646	-1.4638918
C	-3.4147220	-0.9752516	-3.3617548
H	-1.5165810	-0.0084856	-3.0271118
C	-3.8297510	-2.9576676	-2.0557898
H	-2.2652170	-3.5583046	-0.7036438
C	-4.2524740	-2.0339386	-3.0088718
H	-3.7423440	-0.2341386	-4.0952898
H	-4.4886850	-3.7770986	-1.7574128
H	-5.2396630	-2.1303396	-3.4678648
Rh	0.4747770	0.5758314	-0.3619478
H	0.8901980	2.0792694	0.0556842
P	-1.5096240	1.2332814	0.5138032
C	-2.3609950	2.5267574	-0.4769048
C	-3.4247630	3.2908854	0.0176772
C	-1.9086900	2.7473644	-1.7822418
C	-4.0365570	4.2470064	-0.7901798
H	-3.7738790	3.1437524	1.0425482
C	-2.5229340	3.7020654	-2.5916088
H	-1.0509760	2.1651004	-2.1366358
C	-3.5897810	4.4509144	-2.0968048
H	-4.8650500	4.8410114	-0.3959008
H	-2.1608150	3.8676544	-3.6095328
H	-4.0701720	5.2037474	-2.7271648
C	-1.4405200	2.0231594	2.1859852
C	-2.4729050	1.8803164	3.1233892
C	-0.3414990	2.8273864	2.5128512
C	-2.4041860	2.5255954	4.3577702
H	-3.3381220	1.2551974	2.8938732
C	-0.2771660	3.4765774	3.7436612
H	0.4677220	2.9286954	1.7851262
C	-1.3069630	3.3255644	4.6719742
H	-3.2165000	2.3996954	5.0783472
H	0.5888070	4.1001964	3.9804182
H	-1.2529040	3.8286094	5.6408852
C	-2.8248430	-0.0209686	0.7971492
C	-4.0766870	0.0037954	0.1773392
C	-2.5197670	-1.0734196	1.6722762
C	-5.0146210	-0.9941646	0.4470882
H	-4.3252730	0.8041094	-0.5220148
C	-3.4576700	-2.0639636	1.9452542
H	-1.5379640	-1.1104946	2.1501702
C	-4.7130930	-2.0232036	1.3360702
H	-5.9880970	-0.9656236	-0.0487698
H	-3.2040470	-2.8743286	2.6334362
H	-5.4513070	-2.8012476	1.5464662

[Rh(DPPP)H]

PBE0 SCF energy: -1836.12705 a. u.

PBE0 free energy: -1835.727641 a. u.

M06 SCF energy: -1837.87343 a. u.

M06 free energy: -1837.47872 a. u.

C	-1.3412935	-0.8357595	1.8226581
C	-0.0735745	-0.1140835	2.2677071
H	-2.2088465	-0.4843715	2.4021501
H	-1.2495135	-1.9197245	1.9953641
H	-0.0292465	-0.1485145	3.3689211
H	-0.1530675	0.9540825	2.0060601
C	-2.0966625	1.1848405	-0.0386299
C	-3.0427715	1.7687275	0.8156941

C	-1.4047035	1.9943295	-0.9457929
C	-3.2879315	3.1383225	0.7607211
H	-3.5987475	1.1490765	1.5248131
C	-1.6476695	3.3669365	-0.9984839
H	-0.6599575	1.5296545	-1.5992709
C	-2.5882965	3.9393625	-0.1449599
H	-4.0281735	3.5860365	1.4288291
H	-1.0921945	3.9885695	-1.7049029
H	-2.7793965	5.0149855	-0.1820369
P	1.5442955	-0.6330215	-0.0887669
C	1.2305395	-0.7017015	1.7362661
H	2.0949945	-0.2406265	2.2413431
H	1.2819085	-1.7771785	1.9735861
C	2.0082405	1.1166295	-0.4169449
C	2.0691405	2.1066775	0.5694131
C	2.2685855	1.4710265	-1.7496459
C	2.3692725	3.4274085	0.2300851
H	1.8794145	1.8611175	1.6154921
C	2.5816805	2.7844465	-2.0843799
H	2.2079335	0.7013545	-2.5240239
C	2.6251265	3.7696995	-1.0949729
H	2.4016345	4.1919175	1.0105361
H	2.7860885	3.0441975	-3.1261919
H	2.8591215	4.8039985	-1.3595449
C	3.1814085	-1.4782135	-0.0981499
C	3.2593815	-2.8205415	-0.4807469
C	4.3374925	-0.8230195	0.3431051
C	4.4733425	-3.5023795	-0.4143919
H	2.3563165	-3.3152635	-0.8490469
C	5.5529685	-1.5027965	0.3991801
H	4.2927885	0.2303965	0.6319051
C	5.6223065	-2.8442985	0.0233451
H	4.5240235	-4.5514835	-0.7168859
H	6.4523795	-0.9797975	0.7344351
H	6.5763485	-3.3760675	0.0656081
P	-1.6678335	-0.5993875	0.0212531
C	-3.2754535	-1.4539055	-0.2138869
C	-3.3613615	-2.8139665	0.1219961
C	-4.3758955	-0.8364345	-0.8212339
C	-4.5314155	-3.5297845	-0.1171869
H	-2.4996625	-3.3214065	0.5649351
C	-5.5435675	-1.5584105	-1.0674339
H	-4.3217485	0.2174465	-1.1036949
C	-5.6274465	-2.9032845	-0.7119329
H	-4.5854895	-4.5867015	0.1559641
H	-6.3948235	-1.0627815	-1.5412679
H	-6.5440665	-3.4663995	-0.9041039
Rh	-0.0144725	-1.3241275	-1.3808349
H	1.1885515	-1.8358105	-2.3520219

TS3

PBE0 SCF energy: -2567.510649 a. u.

PBE0 free energy: -2566.84494 a. u.

M06 SCF energy: -2570.210773 a. u.

M06 free energy: -2569.5527 a. u.

C	3.1101621	0.8682602	0.4982148
O	1.9240251	1.4344522	-0.0480072
C	1.3463021	0.3709232	1.9875288
C	2.5780741	-0.2702198	1.3734818
H	1.6314941	0.9917132	2.8529058
H	0.6060811	-0.3607118	2.3265808
H	3.2989061	-0.6356548	2.1135828
H	2.2862541	-1.1020928	0.7215218
C	0.8485551	1.2451342	0.8514728
C	-0.0375699	2.3391932	1.0234098
H	-0.4806679	2.4879152	2.0128248
H	0.1981331	3.2713412	0.5035438
C	3.8495591	1.9026342	1.3515178
C	4.9931061	1.5343992	2.0699248
C	3.4098731	3.2255452	1.4186098
C	5.6747711	2.4660162	2.8477698
H	5.3644681	0.5081432	2.0069708

C	4.0924761	4.1612602	2.1975158
H	2.5260411	3.5154702	0.8500958
C	5.2243161	3.7854762	2.9167718
H	6.5672221	2.1619732	3.4008928
H	3.7338601	5.1930422	2.2404928
H	5.7585461	4.5180732	3.5269348
C	4.0135811	0.4497072	-0.6461482
C	4.6273801	-0.8029428	-0.6876922
C	4.2778581	1.3667662	-1.6704452
C	5.4869071	-1.1377178	-1.7350932
H	4.4328661	-1.5340488	0.0992178
C	5.1311951	1.0333932	-2.7174482
H	3.8060281	2.3511692	-1.6386642
C	5.7409271	-0.2224458	-2.7530522
H	5.9553341	-2.1249228	-1.7554792
H	5.3255731	1.7592492	-3.5113162
H	6.4135941	-0.4844828	-3.5736482
Rh	-1.0537129	0.7940632	-0.0461112
H	-1.5289099	2.2893672	0.2104378
P	-0.6239509	-1.4233078	-0.4768612
P	-3.1907019	0.5426852	-0.7179552
C	-2.0274759	-2.3623058	-1.2483922
H	-1.6617979	-3.3655088	-1.5109542
H	-2.8030009	-2.5016778	-0.4804772
C	-3.5674759	-0.5138158	-2.1938052
H	-4.5959229	-0.8989848	-2.1064652
H	-3.5844029	0.1889952	-3.0403182
C	-2.5658229	-1.6342488	-2.4787942
H	-1.7155179	-1.2208398	-3.0463292
H	-3.0449779	-2.3623238	-3.1526372
C	-4.2131469	2.0124432	-1.1411932
C	-5.6028709	2.0239182	-0.9739862
C	-3.5826999	3.1297282	-1.6998132
C	-6.3479289	3.1384702	-1.3559612
H	-6.1061769	1.1617802	-0.5289852
C	-4.3298069	4.2392732	-2.0915392

H	-2.4946239	3.1225012	-1.8145082
C	-5.7135939	4.2466522	-1.9171792
H	-7.4313529	3.1423802	-1.2110402
H	-3.8270199	5.1066392	-2.5269132
H	-6.2989209	5.1205802	-2.2142762
C	-4.1563899	-0.2002978	0.6655748
C	-4.9612109	-1.3392138	0.5591968
C	-4.0029519	0.3991822	1.9261908
C	-5.5856629	-1.8774128	1.6868908
H	-5.1051099	-1.8285738	-0.4059482
C	-4.6374989	-0.1273518	3.0467548
H	-3.3593989	1.2777382	2.0205018
C	-5.4253359	-1.2750638	2.9319218
H	-6.2010959	-2.7751118	1.5869548
H	-4.5065719	0.3534062	4.0194298
H	-5.9125599	-1.6981248	3.8139448
C	-0.3348089	-2.4524148	1.0187578
C	0.8113641	-3.2161028	1.2606538
C	-1.3245149	-2.3846398	2.0123418
C	0.9650271	-3.8980638	2.4690968
H	1.5991231	-3.2774688	0.5082458
C	-1.1757629	-3.0751188	3.2111758
H	-2.2103159	-1.7638568	1.8513778
C	-0.0264469	-3.8324038	3.4454178
H	1.8698611	-4.4854518	2.6454998
H	-1.9587999	-3.0102818	3.9708538
H	0.0968371	-4.3668398	4.3906748
C	0.7042181	-1.8360828	-1.6713632
C	1.0483841	-3.1522748	-2.0144402
C	1.3049511	-0.7736148	-2.3553832
C	1.9904961	-3.3967908	-3.0099962
H	0.5727951	-3.9959208	-1.5072212
C	2.2307631	-1.0213438	-3.3685652
H	1.0379351	0.2481072	-2.0737892
C	2.5803081	-2.3297968	-3.6909972
H	2.2585801	-4.4255438	-3.2642872

H	2.6938271	-0.1842498	-3.8945242
H	3.3165451	-2.5215008	-4.4756762

4a

PBE0 SCF energy: -731.340412 a. u.

PBE0 free energy: -731.100822 a. u.

M06 SCF energy: -732.297118 a. u.

M06 free energy: -732.060204 a. u.

C	-0.0188424	0.5253724	0.1963675
O	0.3441826	1.5425974	-0.7470325
C	1.5003826	2.0308774	1.2239155
C	0.2721046	1.1914314	1.5504605
H	2.4195936	1.4301434	1.3056605
H	1.6168416	2.9151354	1.8612795
H	0.4363496	0.4670114	2.3559325
H	-0.5717504	1.8402004	1.8227245
C	1.2611336	2.3952884	-0.2153675
C	1.8093416	3.3775314	-0.9353365
H	2.5225276	4.0557604	-0.4694505
H	1.5538106	3.5187804	-1.9856325
C	0.8711546	-0.7007806	-0.0130025
C	0.6896736	-1.8372756	0.7839235
C	1.8757506	-0.7087356	-0.9828065
C	1.5010606	-2.9557896	0.6199515
H	-0.1058674	-1.8533066	1.5332035
C	2.6888816	-1.8314276	-1.1476475
H	2.0224516	0.1697824	-1.6127125
C	2.5066306	-2.9564236	-0.3480635
H	1.3442626	-3.8357086	1.2488845
H	3.4713836	-1.8221826	-1.9106665
H	3.1433386	-3.8347946	-0.4790735
C	-1.4700684	0.1593604	-0.0438585
C	-2.3362164	-0.1240946	1.0149545
C	-1.9449544	0.0538454	-1.3558745
C	-3.6553774	-0.5059086	0.7684685
H	-1.9863684	-0.0469436	2.0466175
C	-3.2635814	-0.3178426	-1.6012975
H	-1.2721064	0.2684144	-2.1881795
C	-4.1236374	-0.6013086	-0.5395635
H	-4.3210294	-0.7241296	1.6072945
H	-3.6229634	-0.3893546	-2.6308945
H	-5.1580914	-0.8955236	-0.7331785

PPh_3

PBE0 SCF energy: -1034.81488 a. u.

PBE0 free energy: -1034.586998 a. u.

P	0.0002076	0.0018522	-1.4282241
C	1.4752846	0.7372832	-0.6124911
C	2.7280366	0.4243652	-1.1601981
C	1.4147226	1.5831702	0.5015109
C	3.8960336	0.9275202	-0.5925611
H	2.7856876	-0.2223838	-2.0410931
C	2.5845976	2.0972652	1.0604769
H	0.4464946	1.8411552	0.9369079
C	3.8263036	1.7682792	0.5188409
H	4.8657296	0.6701652	-1.0262811
H	2.5238956	2.7583302	1.9289699
H	4.7416866	2.1710372	0.9598429
C	-1.3739564	0.9097522	-0.6107231
C	-2.0738844	0.4333042	0.5042089
C	-1.7336774	2.1492442	-1.1596211
C	-3.1072654	1.1860562	1.0618869
H	-1.8088704	-0.5322728	0.9413509
C	-2.7566264	2.9058382	-0.5934491
H	-1.2031194	2.5232502	-2.0405921
C	-3.4482124	2.4237322	0.5183759
H	-3.6476574	0.8020702	1.9310749
H	-3.0227014	3.8724292	-1.0289251
H	-4.2575134	3.0121202	0.9581209
C	-0.0988574	-1.6434178	-0.6124051
C	-0.9940194	-2.5728698	-1.1616891
C	0.6625656	-2.0128678	0.5029949

C	-1.1420484	-3.8361088	-0.5942581
H	-1.5821464	-2.2996548	-2.0430941
C	0.5235996	-3.2833588	1.0614459
H	1.3688446	-1.3026918	0.9393659
C	-0.3806474	-4.1951108	0.5184639
H	-1.8486424	-4.5478338	-1.0288601
H	1.1250186	-3.5603658	1.9312069
H	-0.4888634	-5.1892838	0.9594209

References

1 M. Ogasawara, K. Takizawa and T. Hayashi, *Organometallics*, 2002, **21**, 4853–4861.

2 C. J. Curtis, A. Miedaner, W. W. Ellis and D. L. DuBois, *J. Am. Chem. Soc.*, 2002, **124**, 1918–1925.

3 K. Tani, E. Tanigawa, Y. Tatsuno and S. Otsuka, *J. Organomet. Chem.*, 1985, **279**, 87–101.

4 F. A. L. Anet and P. M. G. Bavin, *Can. J. Chem.*, 1957, **35**, 1084–1087.

5 B. Andes Hess, Jr., A. S. Bailey, V. Boekelheide, *J. Am. Chem. Soc.*, 1967, **89**, 2746–2747.

6 L. A. van der Veen, P. H. Keeven, G. C. Schoemaker, J. N. H. Reek, P. C. J. Kamer, P. van Leeuwen, M. Lutz and A. L. Spek, *Organometallics*, 2000, **19**, 872–883.

7 C. Aïssa, K. Y. T. Ho, D. J. Tetlow and M. Pin-Nó, *Angew. Chemie Int. Ed.*, 2014, **53**, 4209–4212.

8 P. Zhao, C. D. Incarvito and J. F. Hartwig, *J. Am. Chem. Soc.*, 2006, **128**, 9642–9643.

9 T. Kondo, F. Tsunawaki, R. Sato, Y. Ura, K. Wada and T. Mitsudo, *Chem. Lett.*, 2003, **32**, 24–25.

10 I. M. R. G. Princival, J. G. Ferreira, T. G. Silva, J. S. Aguiar and J. L. Princival, *Bioorg. Med. Chem. Lett.*, 2016, **26**, 2839–2842.

11 K. Kurosawa and T. Tsujita, *Bull. Chem. Soc. Jpn.*, 1981, **54**, 2391–2394.

12 D. Anastasiou and W. R. Jackson, *Aust. J. Chem.*, 1992, **45**, 21–37.

13 J. P. Perdew, K. Burke and M. Ernzerhof, *Phys. Rev. Lett.*, 1996, **77**, 3865–3868.

14 J. P. Perdew, K. Burke and M. Ernzerhof, *Phys. Rev. Lett.*, 1997, **78**, 1396–1396.

15 C. Adamo and V. Barone, *J. Chem. Phys.*, 1999, **110**, 6158–6170.

16 M. Ernzerhof and G. E. Scuseria, *J. Chem. Phys.*, 1999, **110**, 5029–5036.

17 G. Lu, C. Fang, T. Xu, G. Dong and P. Liu, *J. Am. Chem. Soc.*, 2015, **137**, 8274–8283.
